# High expression of the breast cancer susceptibility gene BRCA1 in long-lived termite kings

**DOI:** 10.18632/aging.101578

**Published:** 2018-10-11

**Authors:** Eisuke Tasaki, Yuki Mitaka, Tomonari Nozaki, Kazuya Kobayashi, Kenji Matsuura, Yoshihito Iuchi

**Affiliations:** 1 Laboratory of Insect Ecology, Graduate School of Agriculture, Kyoto University, Kyoto 606-8502, Japan; 2 Department of Applied Bioresources Chemistry, The United Graduate School of Agriculture, Tottori University, Tottori 680-8553, Japan; 3 Hokkaido Forest Research Station, Field Science Education and Research Center, Kyoto University, Hokkaido 088-2339, Japan; 4 Graduate School of Sciences and Technology for Innovation, Yamaguchi University, Yamaguchi 753-8515, Japan; * Equal contribution

**Keywords:** DNA repair, lifespan, social insects, gene expression

## Abstract

Aging is associated with the accumulation of DNA damage. High expression of DNA repair genes has been suggested to contribute to prolonged lifespan in several organisms. However, the crucial DNA repair genes contributing to longevity remain unknown. Termite kings have an extraordinary long lifespan compared with that of non-reproductive individuals such as workers despite being derived from the same genome, thus providing a singular model for identifying longevity-related genes. In this study, we demonstrated that termite kings express higher levels of the breast cancer susceptibility gene BRCA1 than other castes. Using RNA sequencing, we identified 21 king-specific genes among 127 newly annotated DNA repair genes in the termite *Reticulitermes speratus*. Using quantitative PCR, we revealed that some of the highly expressed king-specific genes were significantly upregulated in reproductive tissue (testis) compared to their expression in somatic tissue (fat body). Notably, BRCA1 gene expression in the fat body was more than 4-fold higher in kings than in workers. These results suggest that BRCA1 partly contributes to DNA repair in somatic and reproductive tissues in termite kings. These findings provide important insights into the linkage between BRCA1 gene expression and the extraordinary lifespan of termite kings.

## Introduction

Genomic instability and DNA damage are hallmarks of aging and number of aging-linked diseases in multicellular organisms [1]. Indeed, the frequency of DNA damage increases with age in humans, rodents, and flies [2,3]. Aging-associated DNA damage is normally repaired by various DNA repair proteins [4]. Thus, these proteins are highly conserved in many organisms, and they play important roles in organismal aging and longevity [4]. In mammals, longer-lived species such as humans and naked mole rats exhibit higher expression of DNA repair genes than short-lived mice [5]. Moreover, overexpression of several DNA repair genes contributes to the extension of lifespan in the fly *Drosophila melanogaster* [6]. These studies suggest that efficient DNA repair system contributes to extended longevity. However, the crucial DNA repair genes contributing to extraordinary longevity are unclear.

In eusocial insects, such as ants, honeybees, and termites, the lifespan of queens is more than 10-fold longer than that of non-reproductive individuals (workers and soldiers) possessing the same genome, a fact that has attracted significant attention [7–9]. In contradistinction to the queens, reproductive male ants and honeybees have a relatively short lifespan (2–3 months) [10,11]. Conversely, termite kings exhibit extraordinary longevity similarly as the queens [12]. This illustrates that termite kings have an exceptional anti-aging system. A previous study focusing on somatic tissues reported that long-lived queens displayed higher expression of DNA repair genes than short-lived workers in the ant *Lasius niger* [13]. These findings suggest that highly efficient DNA repair mechanisms can also contribute to the extraordinary longevity of termite kings.

The Japanese subterranean termite *Reticulitermes speratus* is one of the most studied termites due to its reproductive system [14]. A novel parthenogenetic system known as asexual queen succession (AQS) was revealed in *R. speratus* [15]. AQS allows queens to extend their reproductive lifespans because a founding queen (primary queen; PQ) is genetically immortal until a colony dies due to the production of a large number of nest queens (secondary queens; SQs) through parthenogenesis. Conversely, a founding king (primary king; PK) should continue to live even if queens are replaced because mother–son inbreeding depression can occur when the PK dies, reducing the genetic contribution to offspring [14,16,17]. Indeed, a previous study has revealed that this king replacement does not often occur in wild colonies of *R. speratus* [18]. It is thus presumed that PKs in *R. speratus* have been exposed to selection for inbreeding avoidance. As a previous study reported that *Reticulitermes* termite queens surprisingly live more than 11 years [19], the PKs of *R. speratus* may have the potential to live for several decades. Therefore, *R. speratus* provides an ideal model for identifying the critical DNA repair genes contributing to extended longevity.

In this study, we performed transcriptome analysis of DNA repair genes using RNA sequencing (RNA-seq) data obtained from all castes of *R. speratus* (both females and males workers, soldiers, alates, young PQs and PKs, and mature SQs and PKs) [20]. We additionally investigated age-dependent expression changes in male reproductives (male alates and young and mature PKs). Furthermore, we investigated where DNA repair genes were overexpressed in the termite body using quantitative PCR (qPCR) analysis. This study uncovered novel evidence that DNA repair functions as an anti-aging mechanism in termite kings.

## RESULTS

### Digital transcriptome analyses of DNA repair genes in *R. speratus*

**Figure 1:**
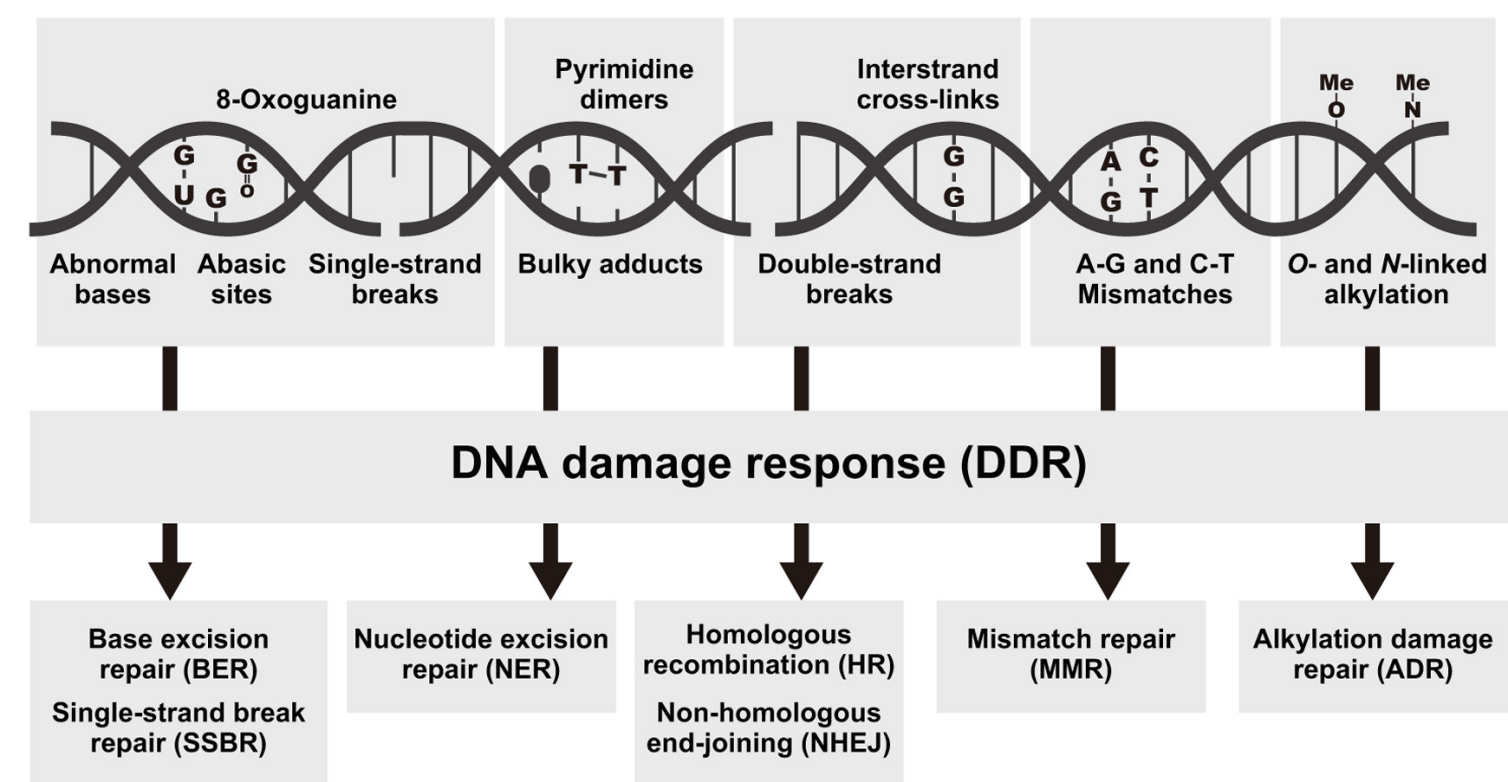
**Types of DNA damage and the associated repair pathways.** Examples of DNA lesions (top), activation of the DNA damage response (DDR, middle), and the most relevant DNA repair pathways responsible for the removal of the lesions (bottom).

Aging-associated DNA damage including abnormal bases, abasic sites, 8-oxoguanine, single-strand breaks (SSBs), bulky adducts, pyrimidine dimers, double-strand breaks (DSBs), interstrand cross-links, and genomic mismatches are generally repaired by various pathways such as base excision repair (BER), SSB repair (SSBR), nucleotide excision repair (NER), homologous recombination (HR), non-homologous end-joining (NHEJ), mismatch repair (MMR), and alkylation damage repair (ADR) [4,21–26] (Fig. 1). Using RNA-seq, we identified 127 putative DNA repair genes including 50 DNA damage response (DDR) genes, 26 HR genes, 16 NER genes, 11 NHEJ genes, 9 ADR genes, 7 MMR genes, 4 genes related to various DNA repair-related pathways, 3 BER genes, and 1 SSBR gene in *R. speratus* (Table S1 and Table S2).

**Figure 2:**
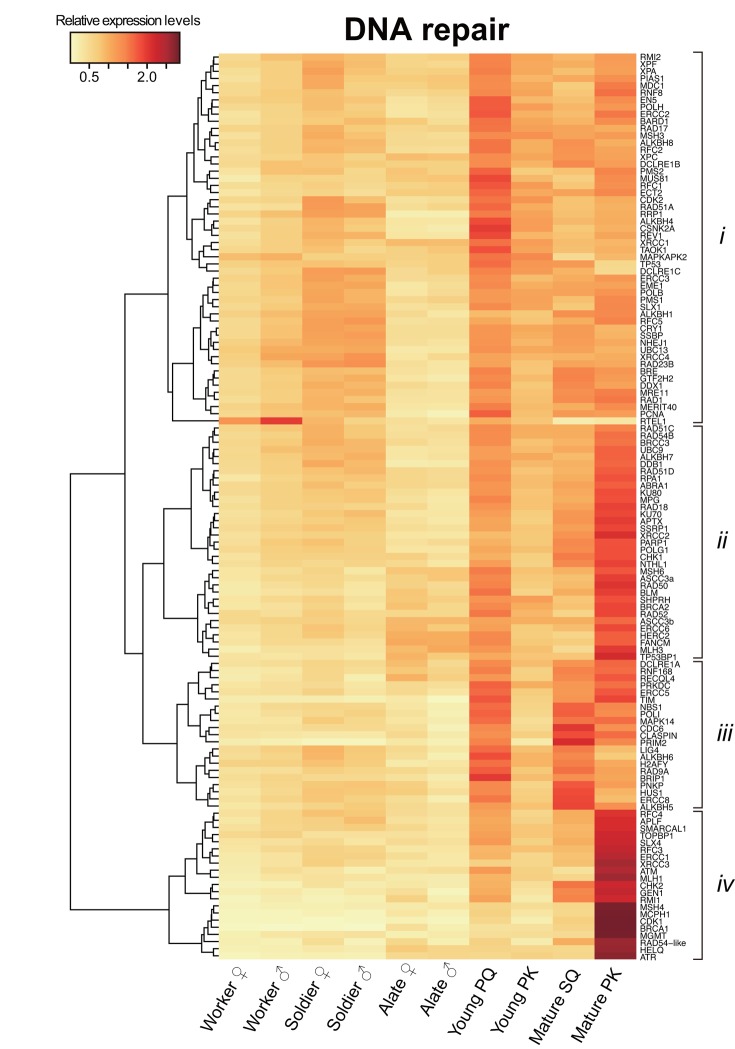
**Differential transcriptome analysis of DNA repair genes among castes.** The heatmap shows the differential expression of 127 DNA repair transcripts among castes in *Reticulitermes speratus*. Workers and soldiers are non-reproductive individuals. Alates, young primary queens (PQs) and primary kings (PKs), and mature secondary queens (SQs) and PKs are reproductives. After nuptial flight, a pair of female and male alates establishes a new colony and starts to produce offspring sexually as a PQ and PK, respectively. The relative expression level is indicated by the mean normalized count per million, ranging from white to red. The tree at the left corresponds to the hierarchical clustering of cluster-averaged expression (clusters *i–iv*).

**Figure 3:**
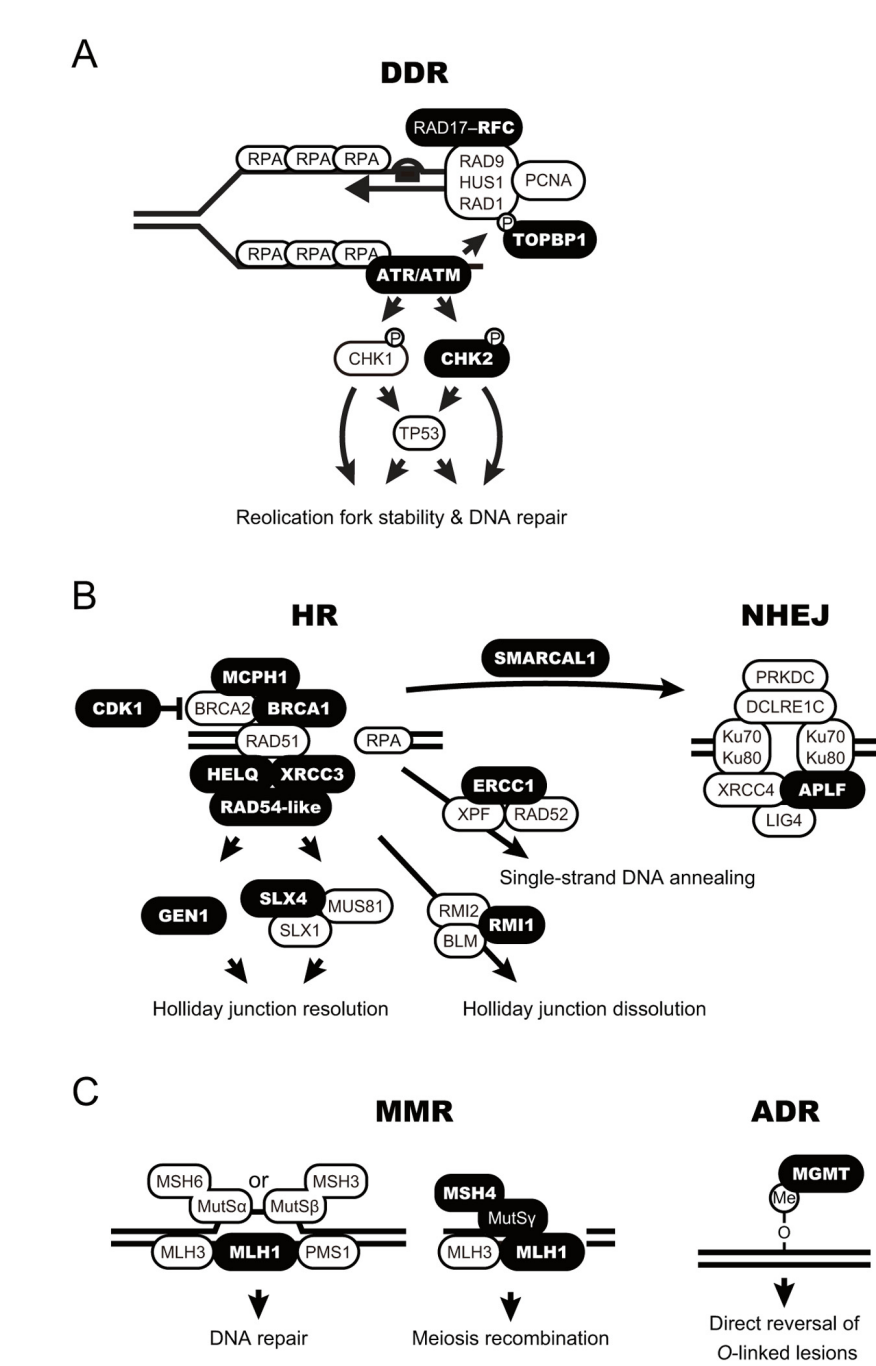
**Schematic model for accelerated DNA repair pathways in termite kings.** (**A**-**C**) DNA damage response (DDR; **A**), homologous recombination and non-homologous end-joining (HR and NHEJ, respectively; **B**), and mismatch repair and alkylation damage repair (MMR and ADR, respectively; **C**) are shown as schematic models. The proteins encoded by the king-specific genes (cluster *iv* in Figure 2) are highlighted in bold.

Digital transcriptome analysis indicated that the expression of 97 genes differs among castes (FDR < 0.05; Fig. 2 and Table S3). We then identified four clusters of genes with significantly varied expression among castes (clusters *i*–*iv*). The clusters had tendencies for high gene expression in young PQs (cluster *i*); young PQs and mature PKs (cluster *ii*); young PQs, SQs and mature PKs (cluster *iii*); or mature PKs (cluster *iv*). Consequently, we identified 21 mature PK-specific genes including nine HR repair genes, six DDR genes, two NHEJ repair genes, two MMR genes, one NER genes, and one ADR gene in cluster *iv* (Table 1). Moreover, principal component analysis (PCA) largely confirmed the results of clustering (Fig. S1 and Table S4). Thus, RNA-seq analysis revealed that mature PKs had superior DDR and HR repair pathways relative to the other castes (Figs. 3A and 3B). MMR and ADR signals are also activated in PKs (Fig. 3C). Moreover, we revealed that the expression of 93 genes (including 40 DDR genes, 19 HR genes, 9 NER genes, 7 NHEJ genes, 6 ADR genes, 5 MMR genes, 4 genes related to various DNA repair-related pathways, and 3 BER genes) increases significantly with age in male reproductives (Table S5). These 93 genes included the aforementioned 21 mature PK-specific genes.

**Table 1:** King-specific gene list.

**Gene symbol**	**Full gene name**	**Signal**
RFC3	Replication factor C subunit 3	DDR
RFC4	Replication factor C subunit 4	DDR
TOPBP1	DNA topoisomerase 2-binding protein 1	DDR
ATR	Serine/threonine-protein kinase ATR	DDR
ATM	Serine-protein kinase ATM	DDR
CHK2	Serine/threonine-protein kinase Chk2	DDR
ERCC1	DNA excision repair protein ERCC-1	NER
**BRCA1**	Breast cancer type 1 susceptibility protein	HR
**MCPH1**	Microcephalin	HR
**XRCC3**	X-ray repair cross-complementing protein 3	HR
**CDK1**	Cyclin-dependent kinase 1	HR
HELQ	Helicase POLQ-like	HR
RMI1	RecQ-mediated genome instability protein 1	HR
GEN1	Flap endonuclease GEN	HR
SLX4	Structure specific endonuclease subunit SLX4	HR
RAD54-like	DNA repair and recombination protein RAD54-like	HR
APLF	Aprataxin and PNK-like factor	NHEJ
SMARCAL1	SWI/SNF-related matrix-associated actin-dependent regulator of chromatin subfamily A-like protein 1	NHEJ
**MLH1**	DNA mismatch repair protein Mlh1	MMR
MSH4	MutS protein homolog 4	MMR
**MGMT**	Methylated-DNA—protein-cysteine methyltransferase	ADR

DDR, DNA damage response; NER, nucleotide excision repair; HR, homologous recombination; NHEJ, non-homologous end-joining; MMR, mismatch repair; ADR, alkylation damage repair. The genes written in bold were used for quantitative PCR analysis in this study.

### Caste-specific expression analysis of DNA repair genes

**Figure 4:**
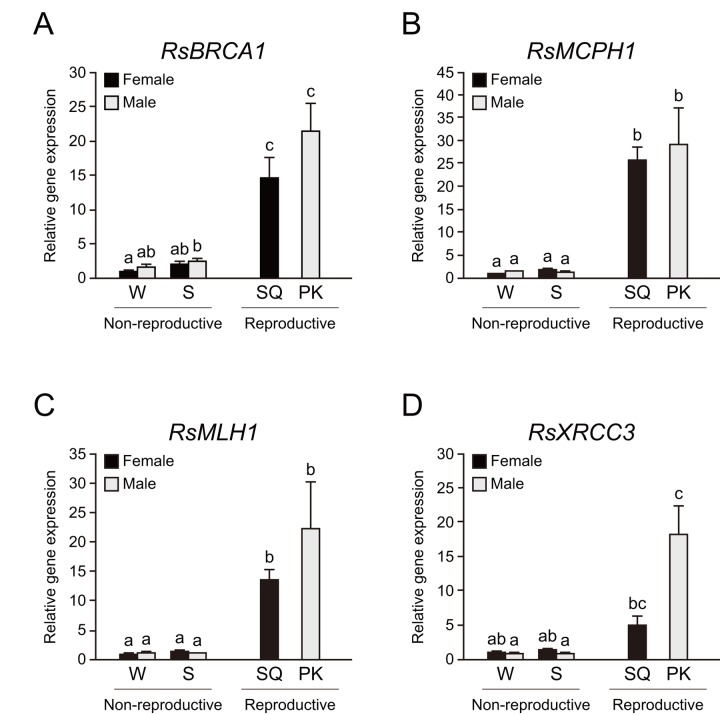
**Higher expression of DNA repair genes in termite reproductives.** (**A**) There were significant differences in *RsBRCA1* expression among castes of *Reticulitermes speratus* (n = 6; *P* < 0.001). *RsBRCA1* expression is higher in reproductives than in non-reproductives (n = 6; *P* < 0.05). (**B**) There were significant differences in *RsMCPH1* expression among castes of *R. speratus* (n = 6; *P* < 0.001). *RsMCPH1* expression is higher in reproductives than in non-reproductives (n = 6; *P* < 0.05). (**C**) There were significant differences in *RsMLH1* expression among castes of *R. speratus* (n = 6; *P* < 0.001). *RsMLH1* expression is higher in reproductives than in non-reproductives (n = 6; *P* < 0.05). (**D**) There were significant differences in *RsXRCC3* expression among castes of *R. speratus* (n = 6; *P* < 0.001). *RsXRCC3* expression is higher in PKs than in non-reproductives (n = 6; *P* < 0.05). Black and gray bars indicate female and male individuals, respectively. Error bars represent the standard error of the mean. Different letters (a–c) over the bars denote significant differences at *P* < 0.05. W, workers; S, soldiers; SQ, mature secondary queens; PK, mature primary kings.

We performed whole-body qPCR analysis to investigate whether termite kings have higher DNA repair gene expression than other castes. We focused on BRCA1, MCPH1, MHL1, and XRCC3 among mature PK-specific genes because these genes had higher expression in cluster *iv* and they play important roles in genome maintenance. Whole-body qPCR revealed that mature PKs and SQs have higher expression of *RsBRCA1*, *RsMCPH1*, and *RsMLH1* than workers and soldiers (n = 6, *P* < 0.05; Figs. 4A–C). *RsXRCC3* was highly expressed only in PKs (n = 6, *P* < 0.05; Fig. 4D).

### Tissue-specific expression analysis in termite workers and reproductives

**Figure 5:**
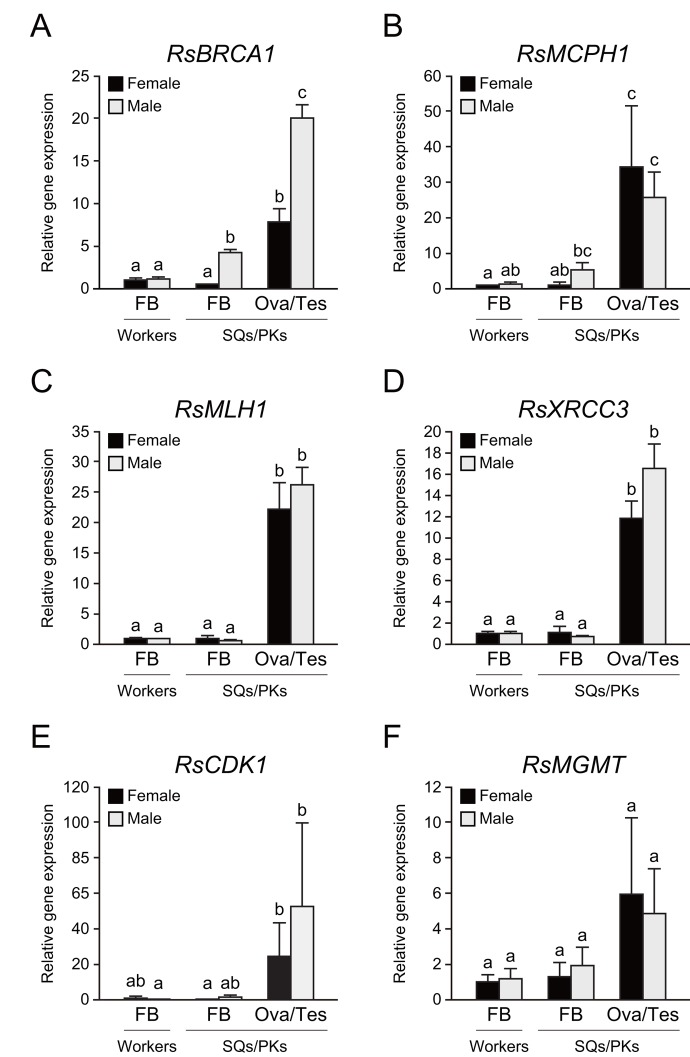
**Tissue-specific expression of several DNA repair genes in termite workers and reproductives.** (**A**) There were significant differences in *RsBRCA1* expression between these tissues (n = 6; *P* < 0.001). *RsBRCA1* expression is higher in testes than in ovaries and the fat body (n = 6; *P* < 0.05). *RsBRCA1* expression is higher in the fat body of PKs than in that of workers and SQs (n = 6; *P* < 0.05). (**B**) There were significant differences in *RsMCPH1* expression between these tissues (n = 6; *P* < 0.001). *RsMCPH1* expression is higher in ovaries and testes than in the fat body of workers and SQs (n = 6; *P* < 0.05). (**C**) There were significant differences in *RsMLH1* expression between these tissues (n = 6; *P* < 0.001). *RsMLH1* expression is higher in the ovaries and testes than in the fat body of workers and reproductives (n = 6; *P* < 0.05). (**D**) There were significant differences in *RsXRCC3* expression between these tissues (n = 6; *P* < 0.001). *RsXRCC3* expression is higher in the ovaries and testes than in the fat body of workers and reproductives (n = 6; *P* < 0.05). (**E**) There were significant differences in *RsCDK1* expression between these tissues (n = 6; *P* < 0.001). *RsCDK1* expression was higher in the ovaries and testes than in the fat body of workers and reproductives (n = 6; *P* < 0.05). (**F**) Although there were significant differences in *RsMGMT* expression between these tissues (n = 6; *P* < 0.05), post-hoc tests revealed no significant differences (n = 6). Black and gray bars indicate females and males, respectively. Error bars represent the standard error of the mean. Different letters (a–c) over the bars denote significant differences at *P* < 0.05. FB, fat body; Ova, ovary; Tes, testis.

To determine whether these repair genes are expressed in the termite body, we performed tissue-specific qPCR analysis in workers and mature reproductives. The testis of PKs displayed the highest *RsBRCA1* expression among all termite tissues (n = 6, *P* < 0.05; Fig. 5A). In addition, the fat body of PKs displayed higher *RsBRCA1* expression than that of workers and SQs (n = 6, *P* < 0.05; Fig. 5A). *RsMCPH1* expression was higher in the ovaries and testes than in the fat body except in all castes excluding PKs (n = 6, *P* < 0.05; Fig. 5B). Meanwhile, *RsMLH1* and *RsXRCC3* expression was higher in the ovaries and testes than in the fat body (n = 6, *P* < 0.05; Figs. 5C and 5D). Furthermore, we assessed the expression levels of *RsCDK1* and *RsMGMT*, which are also considered as mature PK-specific genes, and these tended to be higher in the ovaries and testes than in the fat body, but there were no statistically significant differences (n = 6; Figs. 5E and 5F).

## DISCUSSION

Our analysis demonstrated that mature PKs exhibit higher expression of the BRCA1 gene than other castes in *R. speratus*. BRCA1 is one of the most studied DNA repair genes, particularly in cancer research. Mutation of the gene is linked to familial breast and ovarian cancers [27]. To date, BRCA1 has been implicated in various biological processes, including microRNA biosynthesis [28], antioxidant signaling [29], and homologous DNA repair [30]. However, the role of BRCA1 in organismal aging and longevity has remained mostly unclear. The present study suggests that BRCA1 plays an important role in the extraordinary longevity of termite kings. Our findings suggest that BRCA1 can partly contribute to efficient DNA repair in both somatic and reproductive tissues in termite kings.

BRCA1 is known to have a role of RAD51-mediated HR repair [30]. A previous study reported that the accumulation of RAD51 onto resected DSBs is the cause of HR defects in older flies [3]. Thus, our results indicated that BRCA1 may be a key upstream regulator of the age-related accumulation of RAD51 and the subsequent HR defects. Consistent with this finding, BRCA1 gene overexpression can reduce age-related DSB accumulation and increase cell survival in mouse oocytes [31]. This data provides support for the assertion that BRCA1-regulated HR strongly influences DNA repair efficiency in termite kings with age.

BRCA1, MCPH1, XRCC3, and MLH1 are known to have roles in meiotic progression and the maintenance of genomic stability [32–35]. The germline genome is vulnerable to the accumulation of deleterious mutations during meiotic DNA replication. If those mutations are not eliminated in reproductive cells, errors could be passed to offspring, which is linked to an increased risk of diseases in future generations [36]. Therefore, our findings indicate that strong evolutionary pressures potentially led to the high expression of DNA repair genes in reproductive tissues, especially in the long-lived organisms having the long reproductive periods. Indeed, long-lived humans display lower germline mutation rates than short-lived mice due to high DNA repair gene expression [37]. Additionally, DNA damage levels were lower in long-lived termite queens than in short-lived workers [38]. It is necessary to examine whether mature PKs and SQs have lower levels of DNA damage in reproductive tissues than short-lived species in future research.

The fat body plays major roles in metabolic and reproductive functions in insects [39]. Increased metabolism and reproduction are considered to generate oxidative stress [40–42], which is known to be a cause of DNA damage [43]. The cost of egg production is generally higher than the cost of sperm production [44], suggesting that the fat body of termite queens with long-term reproductive activity may be exposed to strong oxidative stress in comparison to that of the kings. Nevertheless, we found that the fat body of termite kings expressed higher levels of *RsBRCA1* than that of the queens in the present study. Furthermore, we demonstrated that the fat body of the kings does not show high expression levels of *RsCDK1*, which is critical for M-phase entry in mitosis [45]. These findings indicate the possibility that an increased BRCA1 expression is associated with greater longevity and not with cellular stress caused by increased metabolism, reproduction, and mitosis in the fat body of termite kings.

Previous studies described that the long longevity and survival of termites are associated with efficient antioxidant systems [38,46,47]. Thus, although the antioxidant system of termite kings remains unclear, it is estimated that an efficient antioxidant system can prevent the accumulation of DNA damage and contribute to the greater longevity of the kings. Interestingly, a recent study has identified that long-lived reproductives have strong defense systems against transposons as a potential source of DNA damage in comparison to short-lived major workers in the termite *Macrotermes bellicosus* [48]. In addition, long-lived ant queens reportedly display a higher expression of DNA repair genes than short-lived workers [13]. These findings support the hypothesis that the extraordinary longevity of organisms is associated with increased biomolecule homeostasis. However, it is necessary to carefully assess how the reproductives achieve longer lifespans than the non-reproductive individuals in eusocial insects, which has been argued in some studies [49–51]. Therefore, further studies are required to evaluate the biological function of BRCA1 in the longevity of termites using genetic tools, such as CRISPR-Cas9, RNA interference, and transgenic systems. Overall, our study provides new insights into the maintenance mechanisms of biomolecule homeostasis and their links to extraordinary longevity.

## MATERIALS AND METHODS

### Sample collection

Animal ethics committee approval was not required for this study, which used insects. For RNA-seq analysis, alates were extracted from three colonies in secondary forests in Kyoto, Japan, from April to May 2013. One male and one female alate were randomly selected from each colony, and five pairs were created for each colony and kept at 25°C under darkness. They were extracted from each incipient colony after 6 months, and they were regarded as young PQs and PKs. Workers, soldiers, and mature SQs and PKs were collected from four colonies in secondary forests in Kyoto and Shiga, Japan, from July to October 2013. For qPCR analysis, 12 *R. speratus* colonies (workers, soldiers, and mature SQs and PKs) were collected from secondary forests in Yamaguchi and Shiga, Japan, from May to June in 2016 and 2017. Because of the rare appearance of mature PQs in nature [15], we used SQs as mature queens in this study. Whole bodies or separated tissues (reproductive tissues and fat body) of the termites were temporarily preserved at −80°C. Female and male workers and soldiers were separated on the basis of the shape of the caudal sternite using a stereoscope. *R. speratus* is not an endangered or protected species; thus, no specific permits were required for the described field studies, and no specific permissions were required for the locations for termite sampling given that they are public lands.

### *De novo* transcriptome assembly

We analyzed a transcriptomic database of both male and female of workers, soldiers, and alates, young PQs and PKs, and mature SQs and PKs of *R. speratus*, which was constructed via RNA-seq in our previous study [20], to compare the expression levels of DNA repair-related genes among castes. Total RNA was extracted from the whole body of each individual of each reproductive caste (alates, young PQs and PKs, and mature SQs and PKs) using an RNeasy mini kit (Qiagen) following the standardized instructions from the manufacturer. For workers and soldiers, 10 individuals of each sex were pooled to extract a sufficient amount of RNA, and we performed RNA-seq analysis on a total of 60 samples. In accordance with a previously described procedure [52] for first-strand cDNA synthesis, RNA-seq was performed using the Illumina HiSeq 2000 at the Okinawa Institute of Science and Technology Graduate University. After trimming the raw sequencing reads, the remaining reads from all samples were assembled *de novo* using Trinity version trinityrnaseq_r2012–04–27 [53,54], which generates transcriptomic assemblies from short read sequences using the de Bruijn graph algorithm. In a previous study [20], further details of the methods as well as a summary statistics of assembly are provided. Sequence data were deposited in the DNA Data Bank of Japan under the BioProject PRJDB3531, which contains links and access to sample data through the BioSample SAMD00026264–SAMD00026323 and the Sequence Read Archive DRR030795–DRR030854.

### Annotation of DNA repair-related genes

Targets of DNA repair-related genes were selected on the basis of previously reported genes in animals [4]. The peptide sequences of DNA repair-related proteins of *Z. nevadensis*, *Bombyx mori*, and *D. melanogaster* were obtained from the National Center for Biotechnology Information (http://www.ncbi.nlm.nih.gov/) and used as BLAST queries for our peptide database with an e-value cutoff of 1E−60 (Table S1). Analyses of protein families, domains, and motifs were performed using InterProScan in Blast2GO software v5.1.13 [55] (Table S2). Furthermore, to confirm the identity of the BRCA1 gene *RsBRCA1*, we performed multiple amino acid sequence alignments with CLUSTALW and conducted phylogenetic analyses using the molecular evolutionary genetics analysis software MEGA7 [56] (Fig. S2). Gene evolutionary history was inferred using the maximum likelihood method based on the JTT matrix-based model [57], which is the best model based on the Bayesian information criterion. BRCA1 homologs of mammals (*Homo sapiens* and *Mus musculus*), a worm (*Caenorhabditis elegans*), ants (*Harpegnathos saltator*, *Ooceraea biroi*, *Pogonomyrmex barbatus*, and *Camponotus floridanus*), bees (*Apis florea* and *Dufourea novaeangliae*), wasps (*Copidosoma floridanum* and *Nasonia vitripennis*), sawflies (*Neodiprion lecontei* and *Athalia rosae*), termites (*R. speratus* and *Z. nevadensis*), beetles (*Aethina tumida* and *Dendroctonus ponderosae*), and butterflies (*Papilio machaon*, *B. mori*, *Spodoptera litura*, and *Helicoverpa armigera*) were analyzed.

### Abundance estimation and analyses of differential expression

The expression levels of DNA repair genes were estimated using RSEM v1.2.8 [58] separately for the filtered reads from each sample. Raw read counts generated by RSEM were normalized using the trimmed mean of M-value normalization method [59]. Their read counts were then used for differential expression analyses among castes using the “edgeR” package v3.4.2 [60] in R software v3.2.2. Heatmaps and hierarchical clustering (Euclidean distance; Ward’s linkage) were generated using “heatmap.2” in the “gplots” package, and PCA was performed using the “prcomp” function in R software v3.2.2.

### qPCR

We designed primer pairs for each of the DNA repair genes using Primer3 v1.1.4 [61] (Table S6). Using an RNeasy mini kit, total RNA was extracted individually from the whole bodies and separated tissues of workers and soldiers (three pooled individuals) and from the whole bodies and separated tissues of mature SQs and PKs. cDNA was synthesized from RNA using a PrimeScript™ RT reagent kit (Takara) and preserved at −20°C. qPCR was performed using an Applied Biosystems^®^ StepOne™ system (Thermo) with *Power* SYBR™ Green PCR master mix (Thermo). All procedures were performed in accordance with each manufacturer’s protocol. Nicotinamide adenine dinucleotide dehydrogenase subunit 5 (ND5) was selected as the reference gene. Relative expression levels were calculated using a typical ∆∆Ct method. We performed six colony replicates for this experiment.

### Statistical analysis

R software v3.2.2 was used for statistical analyses in qPCR experiments. Normality and homogeneity assumptions for these qPCR data were evaluated by the Shapiro–Wilk test and Bartlett’s test, respectively (*P* < 0.05). We used the Kruskal–Wallis test followed by the Mann–Whitney U test with Holm’s correction for multiple comparisons. All data in graphs are presented as the mean ± standard error of the mean, and all *P* values calculated are provided here. The different letters (a–c) over the bars denote significant differences at *P* < 0.05.

## Supplementary Material

Supplementary Figures

Supplementary Table S1

Supplementary Table S2

Supplementary Table S3

Supplementary Table S4

Supplementary Table S5

Supplementary Table S6

## References

[1] Hoeijmakers JH. DNA damage, aging, and cancer. N Engl J Med. 2009; 361:1475–85. 10.1056/NEJMra0804615 19812404

[2] Moskalev AA, Shaposhnikov MV, Plyusnina EN, Zhavoronkov A, Budovsky A, Yanai H, Fraifeld VE. The role of DNA damage and repair in aging through the prism of Koch-like criteria. Ageing Res Rev. 2013; 12:661–84. 10.1016/j.arr.2012.02.001 22353384

[3] Delabaere L, Ertl HA, Massey DJ, Hofley CM, Sohail F, Bienenstock EJ, Sebastian H, Chiolo I, LaRocque JR. Aging impairs double-strand break repair by homologous recombination in *Drosophila* germ cells. Aging Cell. 2017; 16:320–28. 10.1111/acel.12556 28000382 PMC5334535

[4] Ciccia A, Elledge SJ. The DNA damage response: making it safe to play with knives. Mol Cell. 2010; 40:179–204. 10.1016/j.molcel.2010.09.019 20965415 PMC2988877

[5] MacRae SL, Croken MM, Calder RB, Aliper A, Milholland B, White RR, Zhavoronkov A, Gladyshev VN, Seluanov A, Gorbunova V, Zhang ZD, Vijg J. DNA repair in species with extreme lifespan differences. Aging (Albany NY). 2015; 7:1171–84. 10.18632/aging.100866 26729707 PMC4712340

[6] Shaposhnikov M, Proshkina E, Shilova L, Zhavoronkov A, Moskalev A. Lifespan and Stress Resistance in *Drosophila* with Overexpressed DNA Repair Genes. Sci Rep. 2015; 5:15299. 10.1038/srep15299 26477511 PMC4609912

[7] Jemielity S, Chapuisat M, Parker JD, Keller L. Long live the queen: studying aging in social insects. Age (Dordr). 2005; 27:241–48. 10.1007/s11357-005-2916-z 23598656 PMC3458492

[8] Keller L, Genoud M. Extraordinary lifespans in ants: a test of evolutionary theories of ageing. Nature. 1997; 389:958–60. 10.1038/40130

[9] Page RE Jr, Peng CY. Aging and development in social insects with emphasis on the honey bee, *Apis mellifera* L. Exp Gerontol. 2001; 36:695–711. 10.1016/S0531-5565(00)00236-9 11295509

[10] Jemielity S, Kimura M, Parker KM, Parker JD, Cao X, Aviv A, Keller L. Short telomeres in short-lived males: what are the molecular and evolutionary causes? Aging Cell. 2007; 6:225–33. 10.1111/j.1474-9726.2007.00279.x 17346255 PMC1859884

[11] Rueppell O, Fondrk MK, Page RE Jr. Biodemographic analysis of male honey bee mortality. Aging Cell. 2005; 4:13–19. 10.1111/j.1474-9728.2004.00141.x 15659209 PMC2441913

[12] Boomsma JJ, Baer B, Heinze J. The evolution of male traits in social insects. Annu Rev Entomol. 2005; 50:395–420. 10.1146/annurev.ento.50.071803.130416 15822204

[13] Lucas ER, Privman E, Keller L. Higher expression of somatic repair genes in long-lived ant queens than workers. Aging (Albany NY). 2016; 8:1940–51. 10.18632/aging.101027 27617474 PMC5076446

[14] Matsuura K. Evolution of the asexual queen succession system and its underlying mechanisms in termites. J Exp Biol. 2017; 220:63–72. 10.1242/jeb.142547 28057829

[15] Matsuura K, Vargo EL, Kawatsu K, Labadie PE, Nakano H, Yashiro T, Tsuji K. Queen succession through asexual reproduction in termites. Science. 2009; 323:1687. 10.1126/science.1169702 19325106

[16] Kobayashi K, Hasegawa E, Yamamoto Y, Kawatsu K, Vargo EL, Yoshimura J, Matsuura K. Sex ratio biases in termites provide evidence for kin selection. Nat Commun. 2013; 4:2048. 10.1038/ncomms3048 23807025

[17] Yashiro T, Matsuura K. Termite queens close the sperm gates of eggs to switch from sexual to asexual reproduction. Proc Natl Acad Sci USA. 2014; 111:17212–17. 10.1073/pnas.1412481111 25404335 PMC4260566

[18] Matsuura K, Mizumoto N, Kobayashi K, Nozaki T, Fujita T, Yashiro T, Fuchikawa T, Mitaka Y, Vargo EL. A Genomic Imprinting Model of Termite Caste Determination: Not Genetic but Epigenetic Inheritance Influences Offspring Caste Fate. Am Nat. 2018; 191:677–90. 10.1086/697238 29750562

[19] Vargo EL, Husseneder C. Biology of subterranean termites: insights from molecular studies of *Reticulitermes* and *Coptotermes.* Annu Rev Entomol. 2009; 54:379–403. 10.1146/annurev.ento.54.110807.090443 18793101

[20] Mitaka Y, Kobayashi K, Mikheyev A, Tin MM, Watanabe Y, Matsuura K. Caste-Specific and Sex-Specific Expression of Chemoreceptor Genes in a Termite. PLoS One. 2016; 11:e0146125. 10.1371/journal.pone.0146125 26760975 PMC4712011

[21] Pierce AJ, Johnson RD, Thompson LH, Jasin M. XRCC3 promotes homology-directed repair of DNA damage in mammalian cells. Genes Dev. 1999; 13:2633–38. 10.1101/gad.13.20.2633 10541549 PMC317094

[22] Adelman CA, Lolo RL, Birkbak NJ, Murina O, Matsuzaki K, Horejsi Z, Parmar K, Borel V, Skehel JM, Stamp G, D’Andrea A, Sartori AA, Swanton C, Boulton SJ. HELQ promotes RAD51 paralogue-dependent repair to avert germ cell loss and tumorigenesis. Nature. 2013; 502:381–84. 10.1038/nature12565 24005329 PMC3836231

[23] Mazin AV, Mazina OM, Bugreev DV, Rossi MJ. Rad54, the motor of homologous recombination. DNA Repair (Amst). 2010; 9:286–302. 10.1016/j.dnarep.2009.12.006 20089461 PMC2827677

[24] Keka IS, Mohiuddin, Maede Y, Rahman MM, Sakuma T, Honma M, Yamamoto T, Takeda S, Sasanuma H. Smarcal1 promotes double-strand-break repair by nonhomologous end-joining. Nucleic Acids Res. 2015; 43:6359–72. 10.1093/nar/gkv621 26089390 PMC4513880

[25] Hsieh P, Yamane K. DNA mismatch repair: molecular mechanism, cancer, and ageing. Mech Ageing Dev. 2008; 129:391–407. 10.1016/j.mad.2008.02.012 18406444 PMC2574955

[26] Soll JM, Sobol RW, Mosammaparast N. Regulation of DNA Alkylation Damage Repair: Lessons and Therapeutic Opportunities. Trends Biochem Sci. 2017; 42:206–18. 10.1016/j.tibs.2016.10.001 27816326 PMC5336464

[27] Welcsh PL, King MC. BRCA1 and BRCA2 and the genetics of breast and ovarian cancer. Hum Mol Genet. 2001; 10:705–13. 10.1093/hmg/10.7.705 11257103

[28] Kawai S, Amano A. BRCA1 regulates microRNA biogenesis via the DROSHA microprocessor complex. J Cell Biol. 2012; 197:201–08. 10.1083/jcb.201110008 22492723 PMC3328391

[29] Gorrini C, Baniasadi PS, Harris IS, Silvester J, Inoue S, Snow B, Joshi PA, Wakeham A, Molyneux SD, Martin B, Bouwman P, Cescon DW, Elia AJ, et al. BRCA1 interacts with Nrf2 to regulate antioxidant signaling and cell survival. J Exp Med. 2013; 210:1529–44. 10.1084/jem.20121337 23857982 PMC3727320

[30] Zhao W, Steinfeld JB, Liang F, Chen X, Maranon DG, Jian Ma C, Kwon Y, Rao T, Wang W, Sheng C, Song X, Deng Y, Jimenez-Sainz J, et al. BRCA1-BARD1 promotes RAD51-mediated homologous DNA pairing. Nature. 2017; 550:360–65. 10.1038/nature24060 28976962 PMC5800781

[31] Titus S, Li F, Stobezki R, Akula K, Unsal E, Jeong K, Dickler M, Robson M, Moy F, Goswami S, Oktay K. Impairment of BRCA1-related DNA double-strand break repair leads to ovarian aging in mice and humans. Sci Transl Med. 2013; 5:172ra21. 10.1126/scitranslmed.3004925 23408054 PMC5130338

[32] Broering TJ, Alavattam KG, Sadreyev RI, Ichijima Y, Kato Y, Hasegawa K, Camerini-Otero RD, Lee JT, Andreassen PR, Namekawa SH. BRCA1 establishes DNA damage signaling and pericentric heterochromatin of the X chromosome in male meiosis. J Cell Biol. 2014; 205:663–75. 10.1083/jcb.201311050 24914237 PMC4050732

[33] Liang Y, Gao H, Lin SY, Peng G, Huang X, Zhang P, Goss JA, Brunicardi FC, Multani AS, Chang S, Li K. BRIT1/MCPH1 is essential for mitotic and meiotic recombination DNA repair and maintaining genomic stability in mice. PLoS Genet. 2010; 6:e1000826. 10.1371/journal.pgen.1000826 20107607 PMC2809772

[34] Liu Y, Tarsounas M, O’regan P, West SC. Role of RAD51C and XRCC3 in genetic recombination and DNA repair. J Biol Chem. 2007; 282:1973–79. 10.1074/jbc.M609066200 17114795

[35] Kneitz B, Cohen PE, Avdievich E, Zhu L, Kane MF, Hou H Jr, Kolodner RD, Kucherlapati R, Pollard JW, Edelmann W. MutS homolog 4 localization to meiotic chromosomes is required for chromosome pairing during meiosis in male and female mice. Genes Dev. 2000; 14:1085–97. 10809667 PMC316572

[36] Paul C, Robaire B. Ageing of the male germ line. Nat Rev Urol. 2013; 10:227–34. 10.1038/nrurol.2013.18 23443014

[37] Milholland B, Dong X, Zhang L, Hao X, Suh Y, Vijg J. Differences between germline and somatic mutation rates in humans and mice. Nat Commun. 2017; 8:15183. 10.1038/ncomms15183 28485371 PMC5436103

[38] Tasaki E, Kobayashi K, Matsuura K, Iuchi Y. An Efficient Antioxidant System in a Long-Lived Termite Queen. PLoS One. 2017; 12:e0167412. 10.1371/journal.pone.0167412 28076409 PMC5226355

[39] Arrese EL, Soulages JL. Insect fat body: energy, metabolism, and regulation. Annu Rev Entomol. 2010; 55:207–25. 10.1146/annurev-ento-112408-085356 19725772 PMC3075550

[40] Finkel T, Holbrook NJ. Oxidants, oxidative stress and the biology of ageing. Nature. 2000; 408:239–47. 10.1038/35041687 11089981

[41] Myatt L, Cui X. Oxidative stress in the placenta. Histochem Cell Biol. 2004; 122:369–82. 10.1007/s00418-004-0677-x 15248072

[42] Agarwal A, Saleh RA, Bedaiwy MA. Role of reactive oxygen species in the pathophysiology of human reproduction. Fertil Steril. 2003; 79:829–43. 10.1016/S0015-0282(02)04948-8 12749418

[43] Cooke MS, Evans MD, Dizdaroglu M, Lunec J. Oxidative DNA damage: mechanisms, mutation, and disease. FASEB J. 2003; 17:1195–214. 10.1096/fj.02-0752rev 12832285

[44] Hayward A, Gillooly JF. The cost of sex: quantifying energetic investment in gamete production by males and females. PLoS One. 2011; 6:e16557. 10.1371/journal.pone.0016557 21283632 PMC3026017

[45] Vassilev LT, Tovar C, Chen S, Knezevic D, Zhao X, Sun H, Heimbrook DC, Chen L. Selective small-molecule inhibitor reveals critical mitotic functions of human CDK1. Proc Natl Acad Sci USA. 2006; 103:10660–65. 10.1073/pnas.0600447103 16818887 PMC1502288

[46] Tasaki E, Sakurai H, Nitao M, Matsuura K, Iuchi Y. Uric acid, an important antioxidant contributing to survival in termites. PLoS One. 2017; 12:e0179426. 10.1371/journal.pone.0179426 28609463 PMC5469489

[47] Tasaki E, Kobayashi K, Matsuura K, Iuchi Y. Long-lived termite queens exhibit high Cu/Zn-superoxide dismutase activity. Oxid Med Cell Longev. 2018; 2018:5127251. 10.1155/2018/5127251 29636846 PMC5831368

[48] Elsner D, Meusemann K, Korb J. Longevity and transposon defense, the case of termite reproductives. Proc Natl Acad Sci USA. 2018; 115:5504–09. 10.1073/pnas.1804046115 29735660 PMC6003524

[49] Lucas ER, Keller L. Ageing and somatic maintenance in social insects. Curr Opin Insect Sci. 2014; 5:31–36. 10.1016/j.cois.2014.09.009 32846739

[50] Korb J. Why do social insect queens live so long? Approaches to unravel the sociality-aging puzzle. Curr Opin Insect Sci. 2016; 16:104–07. 10.1016/j.cois.2016.06.004 27720043

[51] Lucas ER, Keller L. New explanation for the longevity of social insect reproductives: transposable element activity. Proc Natl Acad Sci USA. 2018; 115:5317–18. 10.1073/pnas.1806014115 29735706 PMC6003479

[52] Matz M, Shagin D, Bogdanova E, Britanova O, Lukyanov S, Diatchenko L, Chenchik A. Amplification of cDNA ends based on template-switching effect and step-out PCR. Nucleic Acids Res. 1999; 27:1558–60. 10.1093/nar/27.6.1558 10037822 PMC148354

[53] Haas BJ, Papanicolaou A, Yassour M, Grabherr M, Blood PD, Bowden J, Couger MB, Eccles D, Li B, Lieber M, MacManes MD, Ott M, Orvis J, et al. *De novo* transcript sequence reconstruction from RNA-seq using the Trinity platform for reference generation and analysis. Nat Protoc. 2013; 8:1494–512. 10.1038/nprot.2013.084 23845962 PMC3875132

[54] Grabherr MG, Haas BJ, Yassour M, Levin JZ, Thompson DA, Amit I, Adiconis X, Fan L, Raychowdhury R, Zeng Q, Chen Z, Mauceli E, Hacohen N, et al. Full-length transcriptome assembly from RNA-Seq data without a reference genome. Nat Biotechnol. 2011; 29:644–52. 10.1038/nbt.1883 21572440 PMC3571712

[55] Götz S, García-Gómez JM, Terol J, Williams TD, Nagaraj SH, Nueda MJ, Robles M, Talón M, Dopazo J, Conesa A. High-throughput functional annotation and data mining with the Blast2GO suite. Nucleic Acids Res. 2008; 36:3420–35. 10.1093/nar/gkn176 18445632 PMC2425479

[56] Kumar S, Stecher G, Tamura K. MEGA7: Molecular Evolutionary Genetics Analysis Version 7.0 for Bigger Datasets. Mol Biol Evol. 2016; 33:1870–74. 10.1093/molbev/msw054 27004904 PMC8210823

[57] Jones DT, Taylor WR, Thornton JM. The rapid generation of mutation data matrices from protein sequences. Comput Appl Biosci. 1992; 8:275–82. 1633570 10.1093/bioinformatics/8.3.275

[58] Li B, Dewey CN. RSEM: accurate transcript quantification from RNA-Seq data with or without a reference genome. BMC Bioinformatics. 2011; 12:323. 10.1186/1471-2105-12-323 21816040 PMC3163565

[59] Robinson MD, Oshlack A. A scaling normalization method for differential expression analysis of RNA-seq data. Genome Biol. 2010; 11:R25. 10.1186/gb-2010-11-3-r25 20196867 PMC2864565

[60] Robinson MD, McCarthy DJ, Smyth GK. edgeR: a Bioconductor package for differential expression analysis of digital gene expression data. Bioinformatics. 2010; 26:139–40. 10.1093/bioinformatics/btp616 19910308 PMC2796818

[61] Rozen S, Skaletsky H. Primer3 on the WWW for general users and for biologist programmers. Methods Mol Biol. 2000; 132:365–86. 10547847 10.1385/1-59259-192-2:365

