# Supplementary material for: High expression of the breast cancer susceptibility gene BRCA1 in long-lived termite kings
Source: Aging (Albany NY). 2018 Oct 11;10(10):2668–83. doi: 10.18632/aging.101578 (PMC6224230; doi:10.18632/aging.101578)
Supplement: Supplementary Figures [file aging-10-101578-s001.pdf]

SUPPLEMENTARY FIGURES

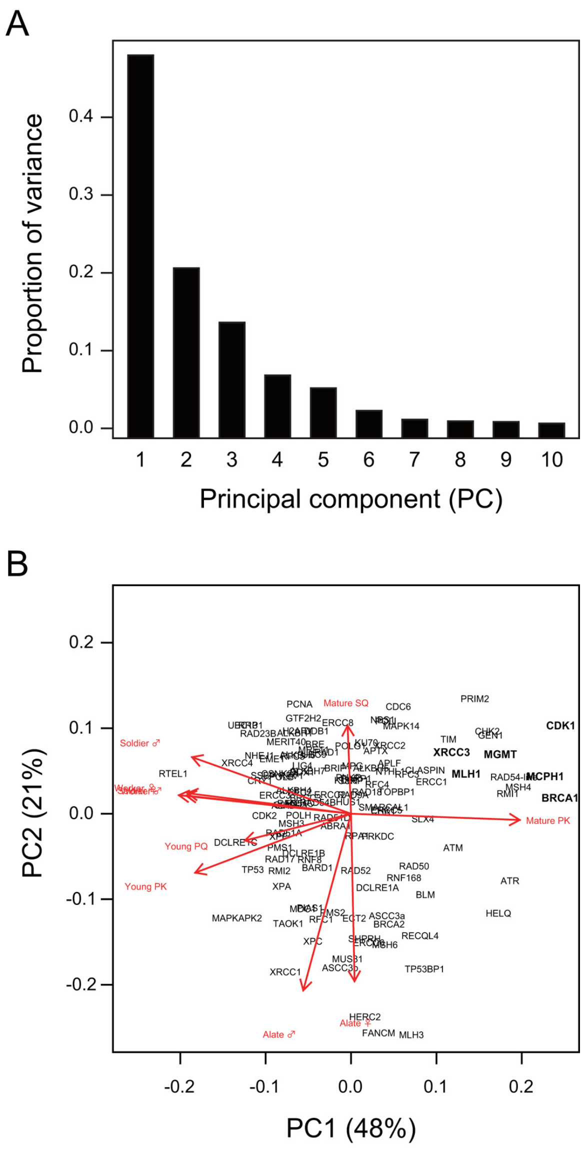

Figure S1. Principal component analysis (PCA) of DNA repair-related gene expression among castes. (**A**) Scree plot illustrating the proportion of variance accounted for by each PC. (**B**) Scores and loading biplots of DNA repair-related genes and termite castes (red arrows) along PCs are shown. PC1 indicates the mature PK-specific genes (PC factor loadings are provided in Table S4). The genes presented in bold were used for qPCR analysis.

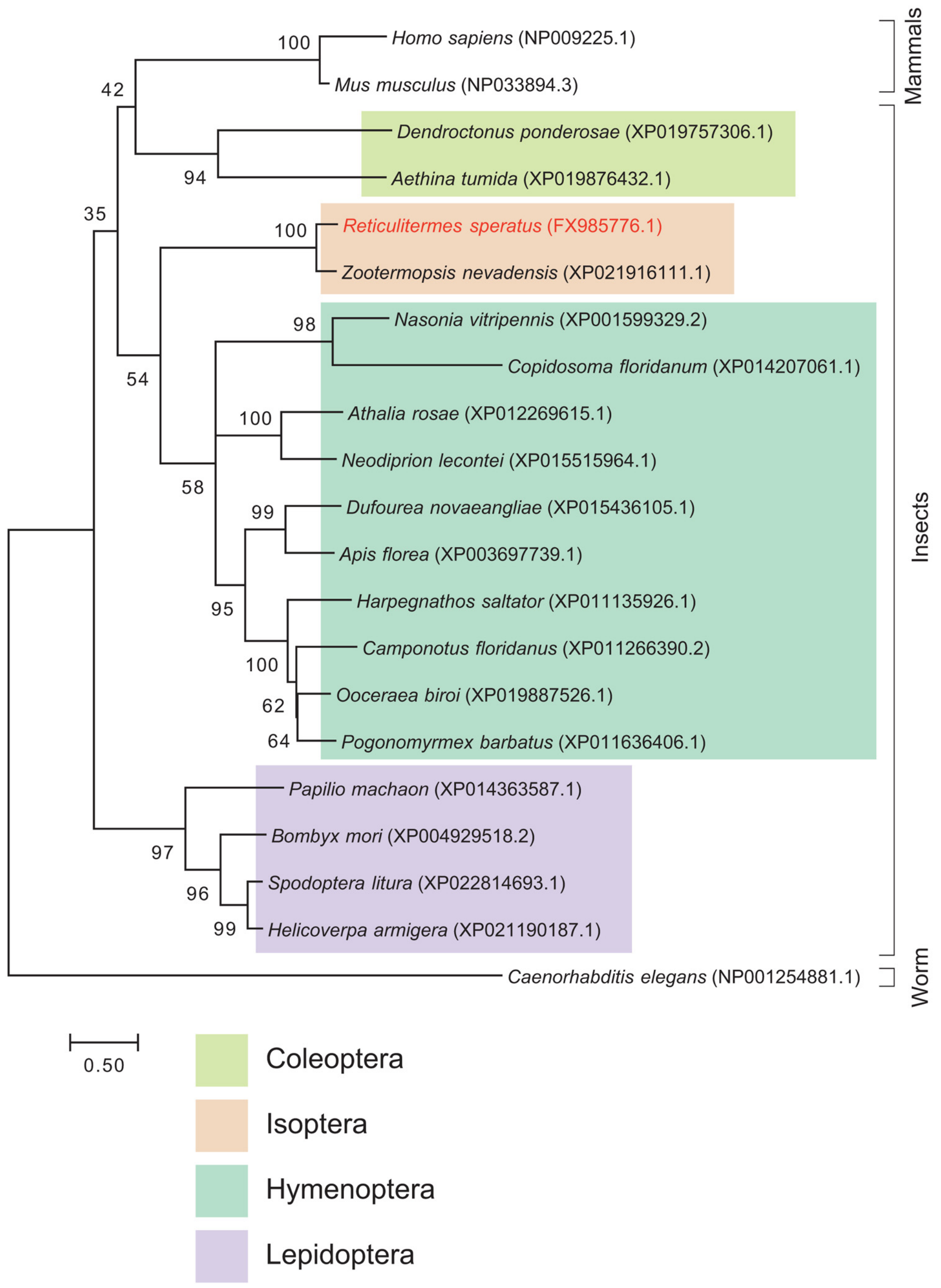

Figure S2. Maximum likelihood molecular phylogenetic tree of BRCA1 homolog sequences. The evolutionary history of the BRCA1 homolog was inferred using the maximum likelihood method based on the JTT matrix-based model. The tree with the highest log likelihood (−9518.81) is shown. The percentage of trees in which the associated taxa clustered together is shown above the branches. Initial tree(s) for the heuristic search were automatically obtained by applying Neighbor-Joining and BioNJ algorithms to a matrix of pairwise distances estimated using a JTT model and then selecting the topology with a superior log likelihood value. A discrete gamma distribution was used to model the evolutionary rate differences among the sites (5 categories [+G, parameter = 1.8985]). The rate variation model allowed for some sites to be evolutionarily invariable ([+I], 1.34% sites). The tree is drawn to scale, with branch lengths measured in the number of substitutions per site. The analysis involved 21 amino acid sequences. All positions containing gaps and missing data were eliminated. There were a total of 231.
