# Supplementary material for: High expression of the breast cancer susceptibility gene BRCA1 in long-lived termite kings
Source: Aging (Albany NY). 2018 Oct 11;10(10):2668–83. doi: 10.18632/aging.101578 (PMC6224230; doi:10.18632/aging.101578)
Supplement: Supplementary Table S1 [file aging-10-101578-s002.docx]

**Table S1. Target gene information.**

| *Reticulitermes speratus* | | | | | | |
| --- | --- | --- | --- | --- | --- | --- |
| Target gene | Target gene ID | Accession no. | Query genes (Accession no.) | Query organisms | E–value | Similarity |
| *RsUBC9* | comp666606_c0_seq2 | FX985741 | SUMO-conjugating enzyme UBC9-B (XP_021938094.1) | *Zootermopsis nevadensis* | 1E−117 | 99% |
|  |  |  | SUMO-conjugating enzyme UBC9-A (KDR09352.1) | *Z. nevadensis* | 5E−100 | 99% |
| *RsRPA1* | comp717937_c4_seq1 | FX985742 | Replication protein A 70 kDa DNA-binding subunit (XP_021932169.1) | *Z. nevadensis* | 0 | 92% |
|  |  |  | Replication protein A 70 kDa DNA-binding subunit (KDR24447.1) | *Z. nevadensis* | 0 | 92% |
| *RsRMI1* | comp728282_c1_seq3 | FX985743 | RecQ-mediated genome instability protein 1-like (XP_021939700.1) | *Z. nevadensis* | 0 | 77% |
|  |  |  | RecQ-mediated genome instability protein 1-like (KDR08209.1) | *Z. nevadensis* | 0 | 77% |
| *RsPARP1* | comp735437_c0_seq1 | FX985744 | LOW QUALITY PROTEIN: poly [ADP-ribose] polymerase-like (XP_021916474.1) | *Z. nevadensis* | 0 | 83% |
|  |  |  | LOW QUALITY PROTEIN: poly [ADP-ribose] polymerase 1-like (XP_021916477.1) | *Z. nevadensis* | 0 | 91% |
|  |  |  | Poly [ADP-ribose] polymerase 1 (KDR21253.1) | *Z. nevadensis* | 8E−103 | 88% |
| *RsCSNK2A* | comp743307_c1_seq4 | FX985745 | Casein kinase II subunit alpha isoform X1 (XP_021914691.1) | *Z. nevadensis* | 0 | 99% |
|  |  |  | Casein kinase II subunit alpha isoform X2 (XP_021914696.1) | *Z. nevadensis* | 0 | 99% |
| *RsRFC3* | comp750891_c1_seq1 | FX985746 | Replication factor C subunit 3 (XP_021942828.1) | *Z. nevadensis* | 0 | 92% |
|  |  |  | Replication factor C subunit 3, partial (KDR22894.1) | *Z. nevadensis* | 0 | 88% |
| *RsRRP1* | comp751690_c0_seq1 | FX985747 | DNA-(apurinic or apyrimidinic site) lyase-like (XP_021917390.1) | *Z. nevadensis* | 0 | 85% |
|  |  |  | Recombination repair protein 1 (KDR20756.1) | *Z. nevadensis* | 0 | 80% |
| *RsRAD23B* | comp764202_c0_seq2 | FX985748 | UV excision repair protein RAD23 homolog B isoform X1 (XP_021927355.1) | *Z. nevadensis* | 0 | 87% |
|  |  |  | UV excision repair protein RAD23-like protein B (KDR15532.1) | *Z. nevadensis* | 0 | 87% |
| *RsAPLF* | comp766890_c0_seq1 | FX985749 | Aprataxin and PNK-like factor isoform X3 (XP_021913415.1) | *Z. nevadensis* | 8E−175 | 74% |
|  |  |  | Aprataxin and PNK-like factor (KDR22686.1) | *Z. nevadensis* | 8E−175 | 74% |
| *RsHUS1* | comp773605_c2_seq2 | FX985750 | Checkpoint protein HUS1 isoform X1 (XP_021921490.1) | *Z. nevadensis* | 3E−168 | 92% |
|  |  |  | Checkpoint protein HUS1 (KDR18613.1) | *Z. nevadensis* | 3E−168 | 92% |
| *RsPOLG1* | comp773670_c4_seq8 | FX985751 | DNA polymerase subunit gamma-1, mitochondrial (XP_021935869.1) | *Z. nevadensis* | 0 | 87% |
|  |  |  | DNA polymerase subunit gamma-1, mitochondrial, partial (KDR10711.1) | *Z. nevadensis* | 0 | 88% |
| *RsNBS1* | comp775837_c0_seq1 | FX985752 | Nibrin (XP_021929811.1) | *Z. nevadensis* | 0 | 68% |
| *RsXRCC3* | comp776406_c0_seq2 | FX985753 | DNA repair protein XRCC3, partial (XP_021927079.1) | *Z. nevadensis* | 7E−177 | 86% |
|  |  |  | DNA repair protein XRCC3 (KDR15679.1) | *Z. nevadensis* | 3E−168 | 77% |
| *RsSSBP* | comp779162_c2_seq3 | FX985754 | Single-strand DNA-binding protein, mitochondrial (XP_021928978.1) | *Z. nevadensis* | 1E−97 | 94% |
|  |  |  | Single-strand DNA-binding protein, mitochondrial (KDR14698.1) | *Z. nevadensis* | 1E−97 | 94% |
| *RsCHK1* | comp781667_c18_seq2 | FX985755 | Serine/threonine-protein kinase grp (XP_021916478.1) | *Z. nevadensis* | 0 | 96% |
|  |  |  | Serine/threonine-protein kinase grp (KDR24204.1) | *Z. nevadensis* | 0 | 96% |
| *RsALKBH1* | comp781939_c1_seq1 | FX985756 | Nucleic acid dioxygenase ALKBH1 (XP_021931764.1) | *Z. nevadensis* | 0 | 80% |
|  |  |  | Alkylated DNA repair protein alkB-like protein 1, partial (KDR13104.1) | *Z. nevadensis* | 9E−135 | 74% |
| *RsMERIT40* | comp782205_c7_seq3 | FX985757 | BRISC and BRCA1-A complex member 1-like (XP_021922426.1) | *Z. nevadensis* | 0 | 91% |
|  |  |  | BRCA1-A complex subunit MERIT40 (KDR18145.1) | *Z. nevadensis* | 0 | 91% |
| *RsMCPH1* | comp782392_c2_seq1 | FX985758 | Microcephalin (KDR08789.1) | *Z. nevadensis* | 0 | 69% |
| *RsALKBH7* | comp784044_c2_seq4 | FX985759 | Alpha-ketoglutarate-dependent dioxygenase alkB homolog 7, mitochondrial (XP_021926268.1) | *Z. nevadensis* | 3E−136 | 87% |
|  |  |  | Alkylated DNA repair protein alkB-like protein 7(KDR16017.1) | *Z. nevadensis* | 3E−136 | 87% |
| *RsTP53* | comp784393_c1_seq4 | FX985760 | Cellular tumor antigen p53 isoform X5 (XP_021937457.1) | *Z. nevadensis* | 1E−81 | 62% |
|  |  |  | Cellular tumor antigen p53 (KDR09760.1) | *Z. nevadensis* | 3E−81 | 62% |
| *RsERCC8* | comp785130_c1_seq2 | FX985761 | DNA excision repair protein ERCC-8-like (XP_021919946.1) | *Z. nevadensis* | 0 | 91% |
|  |  |  | DNA excision repair protein ERCC-8 (KDR19388.1) | *Z. nevadensis* | 0 | 91% |
| *RsXRCC2* | comp785621_c3_seq1 | FX985762 | DNA repair protein XRCC2-like (XP_021922686.1) | *Z. nevadensis* | 1E−169 | 90% |
|  |  |  | DNA repair protein XRCC2 (KDR18012.1) | *Z. nevadensis* | 1E−169 | 90% |
| *RsAPTX* | comp785925_c12_seq3 | FX985763 | Aprataxin isoform X3 (XP_021929429.1) | *Z. nevadensis* | 8E−112 | 89% |
|  |  |  | Aprataxin (KDR14453.1) | *Z. nevadensis* | 5E−111 | 89% |
| *RsMAPK14* | comp786088_c5_seq2 | FX985764 | Mitogen-activating protein kinase 14B-like isoform X1 (XP_021942325.1) | *Z. nevadensis* | 0 | 99% |
|  |  |  | Mitogen-activating protein kinase 14B, partial (KDR24374.1) | *Z. nevadensis* | 0 | 99% |
| *RsNTHL1* | comp786402_c1_seq2 | FX985765 | Endonuclease III-like protein 1 (XP_021931944.1) | *Z. nevadensis* | 0 | 87% |
|  |  |  | Endonuclease III-like protein 1 (KDR13006.1) | *Z. nevadensis* | 0 | 87% |
| *RsSSRP1* | comp786871_c10_seq1 | FX985766 | FACT complex subunit Ssrp1 isoform X1 (XP_021934093.1) | *Z. nevadensis* | 0 | 89% |
|  |  |  | FACT complex subunit Ssrp1 (KDR11732.1) | *Z. nevadensis* | 0 | 89% |
| *RsDCLRE1A* | comp786990_c4_seq9 | FX985767 | DNA cross-link repair 1A protein (KDR15807.1) | *Z. nevadensis* | 0 | 78% |
|  |  |  | DNA cross-link repair 1A protein-like isoform X1 (XP_021926728.1) | *Z. nevadensis* | 3E−153 | 78% |
| *RsMDC1* | comp787453_c10_seq4 | FX985768 | Mediator of DNA damage checkpoint protein 1 (KDR15439.1) | *Z. nevadensis* | 0 | 63% |
|  |  |  | Mediator of DNA damage checkpoint protein 1-like (XP_021927571.1) | *Z. nevadensis* | 1E−128 | 75% |
| *RsEN5* | comp787532_c6_seq3 | FX985769 | Endonuclease V-like (XP_021916327.1) | *Z. nevadensis* | 5E−146 | 89% |
|  |  |  | Putative endonuclease (KDR21324.1) | *Z. nevadensis* | 5E−146 | 89% |
| *RsRTEL1* | comp787593_c7_seq1 | FX985770 | Regulatory of telomere elongation helicase 1-like protein (KDR13998.1) | *Z. nevadensis* | 2E−171 | 81% |
|  |  |  | Regulatory of telomere elongation helicase 1 homolog (XP_021930275.1) | *Z. nevadensis* | 1E−170 | 81% |
| *RsRAD1* | comp787640_c3_seq3 | FX985771 | Cell cycle checkpoint protein RAD1 (XP_021933364.1) | *Z. nevadensis* | 0 | 95% |
|  |  |  | Cell cycle checkpoint protein RAD1 (KDR12148.1) | *Z. nevadensis* | 0 | 95% |
| *RsRAD9A* | comp787726_c9_seq1 | FX985772 | Cell cycle checkpoint control protein RAD9A isoform X1 (XP_021916805.1) | *Z. nevadensis* | 0 | 84% |
|  |  |  | Cell cycle checkpoint control protein RAD9A (KDR21121.1) | *Z. nevadensis* | 0 | 84% |
| *RsRAD50* | comp787877_c7_seq2 | FX985773 | DNA repair protein RAD50 (XP_021933974.1) | *Z. nevadensis* | 0 | 89% |
|  |  |  | DNA repair protein RAD50 (KDR11819.1) | *Z. nevadensis* | 0 | 89% |
| *RsCDC6* | comp787998_c1_seq1 | FX985774 | Cell division control protein 6-like protein (KDR09135.1) | *Z. nevadensis* | 0 | 93% |
|  |  |  | Cell division control protein 6 homolog (XP_021938335.1) | *Z. nevadensis* | 0 | 93% |
| *RsGTF2H2* | comp788477_c6_seq1 | FX985775 | General transcription factor IIH subunit 2 isoform X1 (XP_021941433.1) | *Z. nevadensis* | 0 | 96% |
|  |  |  | General transcription factor IIH subunit 2 (KDR23206.1) | *Z. nevadensis* | 0 | 96% |
| *RsBRCA1* | comp789886_c1_seq2 | FX985776 | Breast cancer type 1 susceptibility protein homolog (XP_021916111.1) | *Z. nevadensis* | 0 | 77% |
|  |  |  | Breast cancer type 1 susceptibility protein-like protein (KDR21368.1) | *Z. nevadensis* | 0 | 77% |
| *RsABRA* | comp789953_c6_seq3 | FX985777 | BRCA1-A complex subunit Abraxas (KDR11421.1) | *Z. nevadensis* | 0 | 80% |
|  |  |  | BRISC complex subunit FAM175B-like isoform X1 (XP_021934569.1) | *Z. nevadensis* | 0 | 80% |
| *RsGEN1* | comp790374_c2_seq3 | FX985778 | Flap endonuclease GEN (XP_021922983.1) | *Z. nevadensis* | 0 | 81% |
|  |  |  | Flap endonuclease GEN (KDR17866.1) | *Z. nevadensis* | 0 | 81% |
| *RsXRCC4* | comp790391_c9_seq4 | FX985779 | XRCC4 coded uncharacterized protein LOC110837903 isoform X1 (XP_021936233.1) | *Z. nevadensis* | 2E−88 | 71% |
| *RsCDK1* | comp790855_c6_seq1 | FX985780 | Cyclin-dependent kinase 1-like isoform X1 (XP_021933362.1) | *Z. nevadensis* | 0 | 97% |
|  |  |  | Cell division control protein 2-like protein (KDR12146.1) | *Z. nevadensis* | 0 | 97% |
| *RsALKBH8* | comp791052_c9_seq9 | FX985781 | Alkylated DNA repair protein alkB homolog 8 isoform X1 (XP_021928884.1) | *Z. nevadensis* | 0 | 88% |
|  |  |  | Alkylated DNA repair protein alkB-like protein 8 (KDR14734.1) | *Z. nevadensis* | 0 | 88% |
| *RsDDB1* | comp791525_c3_seq1 | FX985782 | DNA damage-binding protein 1 isoform X1 (XP_021936210.1) | *Z. nevadensis* | 0 | 99% |
|  |  |  | DNA damage-binding protein 1 (KDR10581.1) | *Z. nevadensis* | 0 | 99% |
| *RsDCLRE1C* | comp791588_c5_seq8 | FX985783 | Protein artemis-lik isoform X1 (XP_021932270.1) | *Z. nevadensis* | 0 | 85% |
|  |  |  | Protein artemis (KDR12848.1) | *Z. nevadensis* | 0 | 85% |
| *RsTIM* | comp792422_c0_seq4 | FX985784 | Protein timeless homolog (XP_021923761.1) | *Z. nevadensis* | 0 | 90% |
|  |  |  | Timeless-like protein, partial (KDR17447.1) | *Z. nevadensis* | 0 | 90% |
| *RsMPG* | comp792462_c1_seq4 | FX985785 | Probable DNA-3-methyladenine glycosylase isoform X2 (XP_021942413.1) | *Z. nevadensis* | 1E−157 | 82% |
|  |  |  | DNA-3-methyladenine glycosylase (KDR23034.1) | *Z. nevadensis* | 1E−157 | 82% |
| *RsPOLI* | comp793382_c2_seq1 | FX985786 | DNA polymerase iota (XP_021932721.1) | *Z. nevadensis* | 0 | 85% |
|  |  |  | DNA polymerase iota (KDR12521.1) | *Z. nevadensis* | 0 | 85% |
| *RsRFC5* | comp793589_c2_seq37 | FX985787 | Replication factor C subunit 5 isoform X1 (XP_021922629.1) | *Z. nevadensis* | 0 | 94% |
|  |  |  | Replication factor C subunit 5 (KDR18066.1) | *Z. nevadensis* | 0 | 94% |
| *RsBRE* | comp793952_c6_seq5 | FX985788 | BRISC and BRCA1-A complex member 2-like isoform X1 (XP_021941409.1) | *Z. nevadensis* | 0 | 95% |
|  |  |  | BRCA1-A complex subunit BRE (KDR06501.1) | *Z. nevadensis* | 0 | 95% |
| *RsSLX4* | comp793988_c21_seq1 | FX985789 | Structure specific endonuclease subunit SLX4-like isoform X1 (XP_021915401.1) | *Z. nevadensis* | 1E−172 | 68% |
| *RsRAD51C* | comp794445_c9_seq8 | FX985790 | DNA repair protein RAD51 homolog 3-like isoform X1 (XP_021935804.1) | *Z. nevadensis* | 0 | 90% |
|  |  |  | DNA repair protein RAD51-like protein 3 (KDR23329.1) | *Z. nevadensis* | 0 | 90% |
| *RsMRE11* | comp794472_c2_seq2 | FX985791 | Double-strand break repair protein MRE11 (XP_021921400.1) | *Z. nevadensis* | 0 | 80% |
|  |  |  | Double-strand break repair protein MRE11 (KDR18710.1) | *Z. nevadensis* | 2E−91 | 91% |
| *RsRAD18* | comp794630_c0_seq3 | FX985792 | E3 ubiquitin-protein ligase RAD18-like isoform X1 (XP_021927432.1) | *Z. nevadensis* | 0 | 70% |
|  |  |  | E3 ubiquitin-protein ligase RAD18 (KDR15487.1) | *Z. nevadensis* | 0 | 70% |
| *RsPCNA* | comp795159_c0_seq1 | FX985793 | Proliferating cell nuclear antigen (XP_021934966.1) | *Z. nevadensis* | 0 | 99% |
|  |  |  | Proliferating cell nuclear antigen (KDR11193.1) | *Z. nevadensis* | 0 | 99% |
| *RsPIAS1* | comp795195_c5_seq2 | FX985794 | E3 SUMO-protein ligase PIAS2 isoform X2 (XP_021928824.1) | *Z. nevadensis* | 0 | 97% |
|  |  |  | E3 SUMO-protein ligase PIAS1 (KDR14768.1) | *Z. nevadensis* | 0 | 97% |
| *RsTAOK1* | comp795349_c9_seq2 | FX985795 | Serine/Threonine-protein kinase Tao isoform X1 (XP_021939254.1) | *Z. nevadensis* | 0 | 99% |
|  |  |  | Serine/Threonine-protein kinase TAO1 (KDR08565.1) | *Z. nevadensis* | 0 | 99% |
| *RsALKBH4* | comp795454_c11_seq2 | FX985796 | Alpha-ketoglutarate-dependent dioxygenase alkB homolog 4 (XP_021922596.1) | *Z. nevadensis* | 0 | 87% |
|  |  |  | Alkylated DNA repair protein alkB-like protein 4 (KDR18060.1) | *Z. nevadensis* | 0 | 87% |
| *RsXPC* | comp797317_c5_seq2 | FX985797 | DNA repair protein complementing XP-C cells homolog isoform X1 (XP_021913565.1) | *Z. nevadensis* | 0 | 82% |
|  |  |  | DNA repair protein complementing XP-C cells-like protein (KDR24336.1) | *Z. nevadensis* | 0 | 82% |
| *RsKU80* | comp797549_c1_seq1 | FX985798 | X-ray repair cross-complementing protein 5 (XP_021931194.1) | *Z. nevadensis* | 0 | 88% |
|  |  |  | ATP-dependent DNA helicase 2 subunit 2 (KDR13452.1) | *Z. nevadensis* | 0 | 88% |
| *RsRFC4* | comp797733_c3_seq7 | FX985799 | Replication factor C subunit 4 (XP_021914470.1) | *Z. nevadensis* | 0 | 95% |
|  |  |  | Replication factor C subunit 4 (KDR22128.1) | *Z. nevadensis* | 0 | 95% |
| *RsRNF168* | comp797745_c0_seq3 | FX985800 | E3 ubiquitin-protein ligase RNF168 (KDR18957.1) | *Z. nevadensis* | 0 | 76% |
|  |  |  | E3 ubiquitin-protein ligase RNF168-like (XP_021920746.1) | *Z. nevadensis* | 3E−142 | 73% |
| *RsCDK2* | comp797989_c2_seq5 | FX985801 | Cyclin-dependent kinase 2-like isoform X1 (XP_021923663.1) | *Z. nevadensis* | 0 | 97% |
|  |  |  | Cell division protein kinase 2, partial (KDR17500.1) | *Z. nevadensis* | 4E−180 | 97% |
| *RsRECQL4* | comp798549_c12_seq1 | FX985802 | ATP-dependent DNA helicase Q4 isoform X2 (XP_021938933.1) | *Z. nevadensis* | 0 | 83% |
|  |  |  | ATP-dependent DNA helicase Q4 (KDR08798.1) | *Z. nevadensis* | 0 | 83% |
| *RsRAD51D* | comp798876_c0_seq5 | FX985803 | DNA repair protein RAD51 homolog 4 isoform X3 (XP_021916043.1) | *Z. nevadensis* | 0 | 91% |
|  |  |  | DNA repair protein RAD51-like protein 4 (KDR21422.1) | *Z. nevadensis* | 0 | 83% |
| *RsLIG4* | comp799329_c0_seq41 | FX985804 | DNA ligase 4 (XP_021923135.1) | *Z. nevadensis* | 0 | 89% |
|  |  |  | DNA ligase 4 (KDR17778.1) | *Z. nevadensis* | 0 | 89% |
| *RsBLM* | comp799738_c5_seq4 | FX985805 | Bloom syndrome protein homolog isoform X1 (XP_021933095.1) | *Z. nevadensis* | 0 | 71% |
| *RsHELQ* | comp800076_c5_seq8 | FX985806 | Helicase POLQ-like isoform X1 (XP_021924763.1) | *Z. nevadensis* | 0 | 85% |
|  |  |  | Helicase POLQ-like (KDR16820.1) | *Z. nevadensis* | 0 | 85% |
| *RsTP53BP1* | comp800398_c17_seq7 | FX985807 | Tumor suppressor p53-binding protein 1 (KDR14143.1) | *Z. nevadensis* | 0 | 69% |
| *RsSHPRH* | comp800688_c4_seq2 | FX985808 | E3 ubiquitin-protein ligase SHPRH isoform X2 (XP_021928095.1) | *Z. nevadensis* | 0 | 97% |
|  |  |  | E3 ubiquitin-protein ligase SHPRH (KDR15144.1) | *Z. nevadensis* | 0 | 97% |
| *RsALKBH6* | comp801409_c0_seq2 | FX985809 | Alpha-ketoglutarate-dependent dioxygenase alkB homolog 6 (XP_021926950.1) | *Z. nevadensis* | 4E−121 | 91% |
|  |  |  | Alkylated DNA repair protein alkB-like protein 6 (KDR15661.1) | *Z. nevadensis* | 4E−121 | 91% |
| *RsPRIM2* | comp801464_c0_seq2 | FX985810 | DNA primase large subunit-like isoform X2 (XP_021940458.1) | *Z. nevadensis* | 0 | 86% |
|  |  |  | DNA primase large subunit (KDR24534.1) | *Z. nevadensis* | 0 | 86% |
| *RsRAD52* | comp801992_c4_seq3 | FX985811 | DNA repair protein RAD52 homolog isoform X1 (XP_021914709.1) | *Z. nevadensis* | 2E−107 | 72% |
|  |  |  | DNA repair protein RAD52-like protein (KDR22011.1) | *Z. nevadensis* | 2E−107 | 72% |
| *RsTOPBP1* | comp802625_c1_seq2 | FX985812 | DNA topoisomerase 2-binding protein 1-B isoform X1 (XP_021933158.1) | *Z. nevadensis* | 0 | 80% |
|  |  |  | DNA topoisomerase 2-binding protein 1 (KDR12289.1) | *Z. nevadensis* | 0 | 80% |
| *RsERCC1* | comp802855_c2_seq4 | FX985813 | DNA excision repair protein ERCC-1 (XP_021934855.1) | *Z. nevadensis* | 2E−164 | 93% |
| *RsRFC2* | comp802916_c1_seq5 | FX985814 | Replication factor C subunit 2 (XP_021914774.1) | *Z. nevadensis* | 0 | 94% |
|  |  |  | Replication factor C subunit 2 (KDR21944.1) | *Z. nevadensis* | 0 | 94% |
| *RsERCC5* | comp803100_c33_seq1 | FX985815 | DNA repair protein complementing XP-G cells homolog (XP_021935303.1) | *Z. nevadensis* | 0 | 82% |
|  |  |  | DNA repair protein complementing XP-G cells (KDR10988.1) | *Z. nevadensis* | 0 | 82% |
| *RsERCC6* | comp803429_c11_seq1 | FX985816 | DNA excision repair protein ERCC-6-like isoform X2 (XP_021935614.1) | *Z. nevadensis* | 0 | 83% |
|  |  |  | DNA excision repair protein ERCC-6 (KDR10852.1) | *Z. nevadensis* | 0 | 83% |
| *RsDCLRE1B* | comp803678_c2_seq5 | FX985817 | DNA cross-link repair 1B protein (KDR20698.1) | *Z. nevadensis* | 0 | 56% |
| *RsRAD54-like* | comp804401_c7_seq1 | FX985818 | DNA repair and recombination protein RAD54-like (XP_021926677.1) | *Z. nevadensis* | 0 | 98% |
|  |  |  | DNA repair and recombination protein RAD54-like (KDR15866.1) | *Z. nevadensis* | 0 | 98% |
| *RsMLH1* | comp804658_c3_seq9 | FX985819 | DNA mismatch repair protein Mlh1 isoform X1 (XP_021924724.1) | *Z. nevadensis* | 0 | 89% |
|  |  |  | DNA mismatch repair protein Mlh1 (KDR23882.1) | *Z. nevadensis* | 0 | 89% |
| *RsBRCA2* | comp805012_c1_seq2 | FX985820 | Breast cancer type 2 susceptibility protein (KDR12341.1) | *Z. nevadensis* | 0 | 49% |
| *RsCHK2* | comp805120_c1_seq2 | FX985821 | Serine/threonine-protein kinase Chk2 (XP_021941758.1) | *Z. nevadensis* | 0 | 86% |
|  |  |  | Serine/threonine-protein kinase Chk2 (KDR97283.1) | *Z. nevadensis* | 0 | 86% |
| *RsBRCC3* | comp805267_c3_seq6 | FX985822 | Lys-63-specific deubiquitinase BRCC36-like isoform X1 (XP_021931518.1) | *Z. nevadensis* | 0 | 98% |
|  |  |  | Lys-63-specific deubiquitinase BRCC36 (KDR13285.1) | *Z. nevadensis* | 0 | 98% |
| *RsSLX1* | comp805527_c0_seq8 | FX985823 | Structure-specific endonuclease subunit slx1 (XP_021939501.1) | *Z. nevadensis* | 3E−145 | 86% |
|  |  |  | Structure-specific endonuclease subunit slx1, partial (KDR08427.1) | *Z. nevadensis* | 4E−145 | 86% |
| *RsPMS1* | comp805573_c9_seq8 | FX985824 | PMS1 protein homolog 1-like (XP_021941278.1) | *Z. nevadensis* | 4E−142 | 88% |
| *RsASCC3b* | comp805680_c4_seq1 | FX985825 | Activating signal cointegrator 1 complex subunit 3 isoform X1 (XP_021913276.1) | *Z. nevadensis* | 0 | 92% |
|  |  |  | Activating signal cointegrator 1 complex subunit 3 (KDR22605.1) | *Z. nevadensis* | 0 | 92% |
| *RsMSH4* | comp805758_c4_seq7 | FX985826 | MutS protein homolog 4-like (XP_021932660.1) | *Z. nevadensis* | 0 | 85% |
|  |  |  | MutS protein homolog 4, partial (KDR12561.1) | *Z. nevadensis* | 0 | 95% |
| *RsPOLB* | comp805922_c7_seq2 | FX985827 | DNA polymerase beta (KDR16351.1) | *Z. nevadensis* | 0 | 93% |
|  |  |  | DNA polymerase beta isoform X4 (XP_021925591.1) | *Z. nevadensis* | 0 | 93% |
| *RsHERC2* | comp806452_c5_seq3 | FX985828 | E3 ubiquitin-protein ligase HERC2 (XP_021924003.1) | *Z. nevadensis* | 0 | 96% |
|  |  |  | E3 ubiquitin-protein ligase HERC2 (KDR17278.1) | *Z. nevadensis* | 0 | 95% |
| *RsSMARCAL1* | comp806472_c3_seq1 | FX985829 | SWI/SNF-related matrix-associated actin-dependent regulator of chromatin subfamily A-like protein 1 isoform X2 (XP_021921060.1) | *Z. nevadensis* | 0 | 88% |
|  |  |  | SWI/SNF-related matrix-associated actin-dependent regulator of chromatin subfamily A-like protein 1 (KDR24079.1) | *Z. nevadensis* | 0 | 88% |
| *RsASCC3a* | comp806992_c5_seq1 | FX985830 | Activating signal cointegrator 1 complex subunit 3-like isoform X1 (XP_021940871.1) | *Z. nevadensis* | 0 | 80% |
| *RsPNKP* | comp807061_c4_seq1 | FX985831 | Bifunctional polynucleotide phosphatase/kinase (KDR16388.1) | *Z. nevadensis* | 3E−126 | 92% |
| *RsRMI2* | comp807085_c7_seq11 | FX985832 | RecQ-mediated genome instability protein 2 (KDR12928.1) | *Z. nevadensis* | 3E−70 | 84% |
| *RsALKBH5* | comp807107_c1_seq1 | FX985833 | RNA demethylase ALKBH5-like (XP_021919973.1) | *Z. nevadensis* | 0 | 97% |
|  |  |  | Alkylated DNA repair protein alkB-like protein 5 (KDR19390.1) | *Z. nevadensis* | 0 | 97% |
| *RsXPF* | comp807337_c7_seq8 | FX985834 | DNA repair endonuclease XPF (XP_021927384.1) | *Z. nevadensis* | 0 | 90% |
|  |  |  | DNA repair endonuclease XPF (KDR15512.1) | *Z. nevadensis* | 0 | 90% |
| *RsCLASPIN* | comp807524_c3_seq6 | FX985835 | Claspin isoform X4 (XP_021936177.1) | *Z. nevadensis* | 0 | 81% |
|  |  |  | Claspin (KDR10568.1) | *Z. nevadensis* | 0 | 81% |
| *RsERCC2* | comp807682_c0_seq3 | FX985836 | TFIIH nasal transcription factor complex helicase XPD subunit (XP_021931504.1) | *Z. nevadensis* | 0 | 99% |
|  |  |  | TFIIH nasal transcription factor complex helicase subunit (KDR13283.1) | *Z. nevadensis* | 0 | 99% |
| *RsMGMT* | comp808363_c6_seq4 | FX985837 | Methylated-DNA—protein-cysteine methyltransferase (XP_021926750.1) | *Z. nevadensis* | 2E−112 | 86% |
|  |  |  | Methylated-DNA—protein-cysteine methyltransferase (KDR15816.1) | *Z. nevadensis* | 2E−96 | 86% |
| *RsUBC13* | comp809027_c2_seq1 | FX985838 | Ubiquitin-conjugating enzyme E2 N (XP_021930699.1) | *Z. nevadensis* | 8E−96 | 100% |
|  |  |  | Ubiquitin-conjugating enzyme E2 N (KDR13781.1) | *Z. nevadensis* | 8E−96 | 100% |
| *RsATR* | comp809151_c13_seq2 | FX985839 | Serine/threonine-protein kinase ATR-like isoform X1 (XP_021924124.1) | *Z. nevadensis* | 0 | 94% |
|  |  |  | Serine/threonine-protein kinase ATR (KDR17199.1) | *Z. nevadensis* | 0 | 94% |
| *RsATM* | comp809623_c5_seq28 | FX985840 | Serine-protein kinase ATM (KDR18215.1) | *Z. nevadensis* | 1E−103 | 83% |
|  |  |  | Serine-protein kinase ATM (XP_021922404.1) | *Z. nevadensis* | 3E−95 | 84% |
| *RsMSH6* | comp809722_c6_seq2 | FX985841 | DNA mismatch repair protein Msh6 isoform X1 (XP_021924017.1) | *Z. nevadensis* | 0 | 90% |
|  |  |  | DNA mismatch repair protein Msh6 (KDR17272.1) | *Z. nevadensis* | 0 | 90% |
| *RsMSH3* | comp810129_c1_seq6 | FX985842 | DNA mismatch repair protein Msh3-like isoform X1 (XP_021932913.1) | *Z. nevadensis* | 0 | 86% |
|  |  |  | DNA mismatch repair protein Msh3 (KDR23480.1) | *Z. nevadensis* | 0 | 86% |
| *RsKU70* | comp810226_c8_seq1 | FX985843 | X-ray repair cross-complementing protein 6 (XP_021923433.1) | *Z. nevadensis* | 0 | 86% |
|  |  |  | ATP-dependent DNA helicase 2 subunit 1 (KDR17626.1) | *Z. nevadensis* | 0 | 86% |
| *RsRNF8* | comp810296_c4_seq10 | FX985844 | E3 ubiquitin-protein ligase rnf8-like isoform X1 (XP_021917531.1) | *Z. nevadensis* | 5E−163 | 84% |
|  |  |  | E3 ubiquitin-protein ligase RNF8 A (KDR24178.1) | *Z. nevadensis* | 5E−163 | 84% |
| *RsH2AFY* | comp810338_c6_seq2 | FX985845 | Core histone macro-H2A.1-like (XP_021942865.1) | *Z. nevadensis* | 0 | 100% |
|  |  |  | Core histone macro-H2A.1 (KDR22855.1) | *Z. nevadensis* | 0 | 100% |
| *RsECT2* | comp810387_c4_seq14 | FX985846 | Protein ECT2 (XP_021939334.1) | *Z. nevadensis* | 0 | 94% |
|  |  |  | Protein ECT2 (KDR08513.1) | *Z. nevadensis* | 0 | 94% |
| *RsERCC3* | comp810587_c6_seq3 | FX985847 | DNA excision repair protein haywire (XP_021936446.1) | *Z. nevadensis* | 0 | 97% |
|  |  |  | DNA excision repair protein haywire (KDR10417.1) | *Z. nevadensis* | 0 | 97% |
| *RsFANCM* | comp810844_c0_seq74 | FX985848 | Fanconi anemia group M protein (KDR10199.1) | *Z. nevadensis* | 0 | 87% |
| *RsCRY1* | comp810921_c1_seq2 | FX985849 | Cryptochrome-1-like (XP_021920747.1) | *Z. nevadensis* | 0 | 91% |
|  |  |  | Cryptochrome-1, partial (KDR18958.1) | *Z. nevadensis* | 0 | 92% |
| *RsPMS2* | comp811054_c10_seq4 | FX985850 | Mismatch repair endonuclease PMS2 isoform X1 (XP_021926703.1) | *Z. nevadensis* | 0 | 85% |
|  |  |  | Mismatch repair endonuclease PMS2 (KDR15822.1) | *Z. nevadensis* | 0 | 85% |
| *RsDDX1* | comp811166_c0_seq6 | FX985851 | ATP-dependent RNA helicase Ddx1 (XP_021921830.1) | *Z. nevadensis* | 0 | 96% |
|  |  |  | ATP-dependent RNA helicase Ddx1 (KDR18468.1) | *Z. nevadensis* | 0 | 96% |
| *RsXPA* | comp811436_c4_seq9 | FX985852 | DNA repair protein complementing XP-A cells homolog (XP_021930690.1) | *Z. nevadensis* | 2E−149 | 88% |
|  |  |  | DNA repair protein complementing XP-A cells-like protein (KDR13782.1) | *Z. nevadensis* | 2E−149 | 88% |
| *RsRAD17* | comp811532_c6_seq58 | FX985853 | Cell cycle checkpoint protein RAD17 isoform X5 (XP_021915134.1) | *Z. nevadensis* | 0 | 80% |
|  |  |  | Cell cycle checkpoint protein RAD17 (KDR21796.1) | *Z. nevadensis* | 0 | 80% |
| *RsRAD51A* | comp811580_c2_seq12 | FX985854 | DNA repair protein RAD51 homolog 1 (XP_021936186.1) | *Z. nevadensis* | 0 | 98% |
|  |  |  | DNA repair protein RAD51-like protein 1 (KDR10565.1) | *Z. nevadensis* | 0 | 98% |
| *RsREV1* | comp811581_c4_seq1 | FX985855 | DNA repair protein REV1 isoform X1 (XP_021918563.1) | *Z. nevadensis* | 0 | 87% |
|  |  |  | DNA repair protein REV1 (KDR20130.1) | *Z. nevadensis* | 0 | 87% |
| *RsBRIP* | comp812334_c7_seq22 | FX985856 | Fanconi anemia group J protein homolog isoform X1 (XP_021939260.1) | *Z. nevadensis* | 0 | 86% |
|  |  |  | Fanconi anemia group J protein-like protein (KDR08569.1) | *Z. nevadensis* | 0 | 86% |
| *RsNHEJ1* | comp812599_c3_seq8 | FX985857 | Non-homologous end-joining factor 1-like isoform X1 (XP_021940864.1) | *Z. nevadensis* | 4E−113 | 79% |
|  |  |  | Non-homologous end-joining factor 1 (KDR07256.1) | *Z. nevadensis* | 4E−113 | 79% |
| *RsMAPKAPK2* | comp812747_c3_seq1 | FX985858 | MAP kinase-activated protein kinase 2 (XP_021929591.1) | *Z. nevadensis* | 0 | 99% |
|  |  |  | MAP kinase-activated protein kinase 2 (KDR14327.1) | *Z. nevadensis* | 0 | 99% |
| *RsRFC1* | comp813084_c0_seq5 | FX985859 | Replication factor C subunit 1 isoform X1 (XP_021910424.1) | *Z. nevadensis* | 0 | 82% |
|  |  |  | Replication factor C subunit 1 (KDR19656.1) | *Z. nevadensis* | 0 | 82% |
| *RsXRCC1* | comp813233_c7_seq4 | FX985860 | DNA repair protein XRCC1 (KDR15428.1) | *Z. nevadensis* | 0 | 70% |
| *RsPOLH* | comp813266_c1_seq37 | FX985861 | DNA polymerase eta isoform X1 (XP_021932263.1) | *Z. nevadensis* | 0 | 80% |
|  |  |  | DNA polymerase eta (KDR12852.1) | *Z. nevadensis* | 0 | 80% |
| *RsMUS81* | comp813274_c0_seq21 | FX985862 | Crossover junction endonuclease MUS81 (XP_021927108.1) | *Z. nevadensis* | 2E−115 | 84% |
|  |  |  | Crossover junction endonuclease MUS81 (KDR15724.1) | *Z. nevadensis* | 2E−115 | 84% |
| *RsRAD54B* | comp813467_c0_seq2 | FX985863 | DNA repair and recombination protein RAD54B-like isoform X4 (XP_021937771.1) | *Z. nevadensis* | 0 | 90% |
|  |  |  | DNA repair and recombination protein RAD54B (KDR09493.1) | *Z. nevadensis* | 0 | 90% |
| *RsBARD1* | comp814729_c12_seq2 | FX985864 | BRCA1-associated RING domain protein 1-like isoform X1 (XP_021937711.1) | *Z. nevadensis* | 0 | 84% |
|  |  |  | BRCA1-associated RING domain protein 1 (KDR23353.1) | *Z. nevadensis* | 0 | 84% |
| *RsEME1* | comp814817_c11_seq1 | FX985865 | Crossover junction endonuclease EME1 (XP_021917979.1) | *Z. nevadensis* | 0 | 80% |
|  |  |  | Crossover junction endonuclease EME1 (KDR20403.1) | *Z. nevadensis* | 0 | 80% |
| *RsMLH3* | comp815779_c3_seq4 | FX985866 | DNA mismatch repair protein Mlh3 (KDR22057.1) | *Z. nevadensis* | 0 | 73% |
| *RsPRKDC* | comp816285_c4_seq18 | FX985867 | DNA-dependent protein kinase catalytic subunit-like isoform X1 (XP_021932883.1) | *Z. nevadensis* | 0 | 84% |
|  |  |  | DNA-dependent protein kinase catalytic subunit (KDR23479.1) | *Z. nevadensis* | 0 | 84% |
