# Supplementary material for: High expression of the breast cancer susceptibility gene BRCA1 in long-lived termite kings
Source: Aging (Albany NY). 2018 Oct 11;10(10):2668–83. doi: 10.18632/aging.101578 (PMC6224230; doi:10.18632/aging.101578)
Supplement: Supplementary Table S2 [file aging-10-101578-s003.docx]

**Table S2. Predicted protein families, domains, and motifs.**

| **Gene name** | **AC** | **Type** | **Name** | **GO IDs** | **Library** |
| --- | --- | --- | --- | --- | --- |
| *RsALKBH5* | [IPR027450](http://www.ebi.ac.uk/interpro/entry/IPR027450) | DOMAIN | Alpha-ketoglutarate-dependent dioxygenase AlkB-like |  | [PF13532 (PFAM)](http://www.ebi.ac.uk/interpro/signature/PF13532) |
|  | [IPR032860](http://www.ebi.ac.uk/interpro/entry/IPR032860) | FAMILY | RNA demethylase ALKBH5 | GO:0035515, GO:0035553, GO:0006397 | [PTHR32074(PANTHER)](http://www.ebi.ac.uk/interpro/signature/PTHR32074) |
|  | [IPR037151](http://www.ebi.ac.uk/interpro/entry/IPR037151) | HOMOLOGOUS_SUPERFAMILY | Alpha-ketoglutarate-dependent dioxygenase AlkB-like superfamily |  | [G3DSA:2.60.120.590(GENE3D)](http://www.ebi.ac.uk/interpro/signature/G3DSA:2.60.120.590) |
|  | no IPR | DOMAIN | Alpha-ketoglutarate-dependent dioxygenase AlkB-like |  | mobidb-lite (MOBIDB_LITE) |
|  | no IPR | FAMILY | RNA demethylase ALKBH5 |  | [SSF51197(SUPERFAMILY)](http://www.ebi.ac.uk/interpro/signature/SSF51197) |
| *RsALKBH6* | [IPR005123](http://www.ebi.ac.uk/interpro/entry/IPR005123) | DOMAIN | Oxoglutarate/iron-dependent dioxygenase | GO:0016491, GO:0055114 | [PS51471(PROSITE_PROFILES)](http://www.ebi.ac.uk/interpro/signature/PS51471) |
|  | [IPR027450](http://www.ebi.ac.uk/interpro/entry/IPR027450) | DOMAIN | Alpha-ketoglutarate-dependent dioxygenase AlkB-like |  | [PF13532 (PFAM)](http://www.ebi.ac.uk/interpro/signature/PF13532) |
|  | [IPR032862](http://www.ebi.ac.uk/interpro/entry/IPR032862) | FAMILY | Alpha-ketoglutarate-dependent dioxygenase alkB homologue 6 |  | [PTHR13069:SF11 (PANTHER)](http://www.ebi.ac.uk/interpro/signature/PTHR13069) |
|  | [IPR037151](http://www.ebi.ac.uk/interpro/entry/IPR037151) | HOMOLOGOUS_SUPERFAMILY | Alpha-ketoglutarate-dependent dioxygenase AlkB-like superfamily |  | [G3DSA:2.60.120.590(GENE3D)](http://www.ebi.ac.uk/interpro/signature/G3DSA:2.60.120.590) |
|  | no IPR | HOMOLOGOUS_SUPERFAMILY | Alpha-ketoglutarate-dependent dioxygenase AlkB-like superfamily |  | [PTHR13069(PANTHER)](http://www.ebi.ac.uk/interpro/signature/PTHR13069) |
|  | no IPR | DOMAIN | Oxoglutarate/iron-dependent dioxygenase |  | [SSF51197(SUPERFAMILY)](http://www.ebi.ac.uk/interpro/signature/SSF51197) |
| *RsABRA1* | [IPR023238](http://www.ebi.ac.uk/interpro/entry/IPR023238) | FAMILY | FAM175 family |  | [PR02051 (PRINTS)](http://www.ebi.ac.uk/interpro/signature/PR02051) |
|  |  |  |  |  | [PTHR31728 (PANTHER)](http://www.ebi.ac.uk/interpro/signature/PTHR31728) |
|  | no IPR | FAMILY | FAM175 family |  | mobidb-lite (MOBIDB_LITE) |
|  |  |  |  |  | mobidb-lite (MOBIDB_LITE) |
|  | no IPR |  |  |  | Coil (COILS) |
| *RsALKBH1* | [IPR004574](http://www.ebi.ac.uk/interpro/entry/IPR004574) | FAMILY | Alkylated DNA repair protein AlkB | GO:0006281, GO:0055114 | [PTHR16557 (PANTHER)](http://www.ebi.ac.uk/interpro/signature/PTHR16557) |
|  | [IPR005123](http://www.ebi.ac.uk/interpro/entry/IPR005123) | DOMAIN | Oxoglutarate/iron-dependent dioxygenase | GO:0016491, GO:0055114 | [PS51471(PROSITE_PROFILES)](http://www.ebi.ac.uk/interpro/signature/PS51471) |
|  | [IPR027450](http://www.ebi.ac.uk/interpro/entry/IPR027450) | DOMAIN | Alpha-ketoglutarate-dependent dioxygenase AlkB-like |  | [PF13532 (PFAM)](http://www.ebi.ac.uk/interpro/signature/PF13532) |
|  | no IPR | DOMAIN | Alpha-ketoglutarate-dependent dioxygenase AlkB-like |  | [PTHR16557:SF2 (PANTHER)](http://www.ebi.ac.uk/interpro/signature/PTHR16557) |
|  | no IPR | DOMAIN | Oxoglutarate/iron-dependent dioxygenase |  | [SSF51197(SUPERFAMILY)](http://www.ebi.ac.uk/interpro/signature/SSF51197) |
| *RsALKBH4* | [IPR005123](http://www.ebi.ac.uk/interpro/entry/IPR005123) | DOMAIN | Oxoglutarate/iron-dependent dioxygenase | GO:0055114, GO:0016491 | [PF03171 (PFAM)](http://www.ebi.ac.uk/interpro/signature/PF03171) |
|  | [IPR032857](http://www.ebi.ac.uk/interpro/entry/IPR032857) | FAMILY | Alpha-ketoglutarate-dependent dioxygenase alkB homologue 4 | GO:0070988 | [PTHR12463(PANTHER)](http://www.ebi.ac.uk/interpro/signature/PTHR12463) |
|  | no IPR | FAMILY | Alpha-ketoglutarate-dependent dioxygenase alkB homologue 4 |  | [SSF51197(SUPERFAMILY)](http://www.ebi.ac.uk/interpro/signature/SSF51197) |
| *RsALKBH7* | [IPR027450](http://www.ebi.ac.uk/interpro/entry/IPR027450) | DOMAIN | Alpha-ketoglutarate-dependent dioxygenase AlkB-like |  | [PF13532 (PFAM)](http://www.ebi.ac.uk/interpro/signature/PF13532) |
|  | [IPR032870](http://www.ebi.ac.uk/interpro/entry/IPR032870) | FAMILY | Alpha-ketoglutarate-dependent dioxygenase alkB homologue 7 | GO:1902445, GO:0006974, GO:0005759 | [PTHR21052(PANTHER)](http://www.ebi.ac.uk/interpro/signature/PTHR21052) |
|  | [IPR037151](http://www.ebi.ac.uk/interpro/entry/IPR037151) | HOMOLOGOUS_SUPERFAMILY | Alpha-ketoglutarate-dependent dioxygenase AlkB-like superfamily |  | [G3DSA:2.60.120.590(GENE3D)](http://www.ebi.ac.uk/interpro/signature/G3DSA:2.60.120.590) |
|  | no IPR | FAMILY | Alpha-ketoglutarate-dependent dioxygenase alkB homologue 7 |  | [SSF51197(SUPERFAMILY)](http://www.ebi.ac.uk/interpro/signature/SSF51197) |
| *RsALKBH8* | [IPR000504](http://www.ebi.ac.uk/interpro/entry/IPR000504) | DOMAIN | RNA recognition motif domain | GO:0003676 | [PS50102(PROSITE_PROFILES)](http://www.ebi.ac.uk/interpro/signature/PS50102) |
|  | [IPR005123](http://www.ebi.ac.uk/interpro/entry/IPR005123) | DOMAIN | Oxoglutarate/iron-dependent dioxygenase | GO:0016491, GO:0055114 | [PS51471(PROSITE_PROFILES)](http://www.ebi.ac.uk/interpro/signature/PS51471) |
|  | [IPR012677](http://www.ebi.ac.uk/interpro/entry/IPR012677) | HOMOLOGOUS_SUPERFAMILY | Nucleotide-binding alpha-beta plait domain superfamily |  | [G3DSA:3.30.70.330(GENE3D)](http://www.ebi.ac.uk/interpro/signature/G3DSA:3.30.70.330) |
|  | [IPR013216](http://www.ebi.ac.uk/interpro/entry/IPR013216) | DOMAIN | Methyltransferase type 11 | GO:0008168, GO:0008152 | [PF08241 (PFAM)](http://www.ebi.ac.uk/interpro/signature/PF08241) |
|  | [IPR027450](http://www.ebi.ac.uk/interpro/entry/IPR027450) | DOMAIN | Alpha-ketoglutarate-dependent dioxygenase AlkB-like |  | [PF13532 (PFAM)](http://www.ebi.ac.uk/interpro/signature/PF13532) |
|  | [IPR029063](http://www.ebi.ac.uk/interpro/entry/IPR029063) | HOMOLOGOUS_SUPERFAMILY | S-adenosyl-L-methionine-dependent methyltransferase |  | [SSF53335(SUPERFAMILY)](http://www.ebi.ac.uk/interpro/signature/SSF53335) |
|  | [IPR032863](http://www.ebi.ac.uk/interpro/entry/IPR032863) | FAMILY | Alkylated DNA repair protein alkB homologue 8 | GO:0002098, GO:0016300, GO:0030488 | [PTHR13069:SF28 (PANTHER)](http://www.ebi.ac.uk/interpro/signature/PTHR13069) |
|  | [IPR034256](http://www.ebi.ac.uk/interpro/entry/IPR034256) | DOMAIN | ALKBH8, RNA recognition motif |  | cd12431 (CDD) |
|  | [IPR035979](http://www.ebi.ac.uk/interpro/entry/IPR035979) | HOMOLOGOUS_SUPERFAMILY | RNA-binding domain superfamily | GO:0003676 | [SSF54928(SUPERFAMILY)](http://www.ebi.ac.uk/interpro/signature/SSF54928) |
|  | [IPR037151](http://www.ebi.ac.uk/interpro/entry/IPR037151) | HOMOLOGOUS_SUPERFAMILY | Alpha-ketoglutarate-dependent dioxygenase AlkB-like superfamily |  | [G3DSA:2.60.120.590(GENE3D)](http://www.ebi.ac.uk/interpro/signature/G3DSA:2.60.120.590) |
|  | no IPR | DOMAIN | ALKBH8, RNA recognition motif |  | cd02440 (CDD) |
|  | no IPR | DOMAIN | Methyltransferase type 11 |  | [PTHR13069(PANTHER)](http://www.ebi.ac.uk/interpro/signature/PTHR13069) |
|  | no IPR | HOMOLOGOUS_SUPERFAMILY | S-adenosyl-L-methionine-dependent methyltransferase |  | [SSF51197(SUPERFAMILY)](http://www.ebi.ac.uk/interpro/signature/SSF51197) |
| *RsAPLF* | [IPR000253](http://www.ebi.ac.uk/interpro/entry/IPR000253) | DOMAIN | Forkhead-associated (FHA) domain | GO:0005515 | cd00060 (CDD) |
|  | [IPR008984](http://www.ebi.ac.uk/interpro/entry/IPR008984) | HOMOLOGOUS_SUPERFAMILY | SMAD/FHA domain superfamily | GO:0005515 | [SSF49879 (SUPERFAMILY)](http://www.ebi.ac.uk/interpro/signature/SSF49879) |
|  | [IPR019406](http://www.ebi.ac.uk/interpro/entry/IPR019406) | DOMAIN | Zinc finger, C2H2, APLF-like |  | [PF10283 (PFAM)](http://www.ebi.ac.uk/interpro/signature/PF10283) |
|  | no IPR | DOMAIN | Zinc finger, C2H2, APLF-like |  | mobidb-lite (MOBIDB_LITE) |
|  |  |  |  |  | mobidb-lite (MOBIDB_LITE) |
|  |  |  |  |  | mobidb-lite (MOBIDB_LITE) |
|  |  |  |  |  | [PTHR21315:SF2 (PANTHER)](http://www.ebi.ac.uk/interpro/signature/PTHR21315) |
|  |  |  |  |  | [PTHR21315 (PANTHER)](http://www.ebi.ac.uk/interpro/signature/PTHR21315) |
|  |  |  |  |  | [PTHR21315 (PANTHER)](http://www.ebi.ac.uk/interpro/signature/PTHR21315) |
|  |  |  |  |  | [PTHR21315:SF2 (PANTHER)](http://www.ebi.ac.uk/interpro/signature/PTHR21315) |
|  |  |  |  |  | [PTHR21315:SF2 (PANTHER)](http://www.ebi.ac.uk/interpro/signature/PTHR21315) |
|  | no IPR |  |  |  | [G3DSA:2.60.200.20 (GENE3D)](http://www.ebi.ac.uk/interpro/signature/G3DSA:2.60.200.20) |
| *RsAPTX* | [IPR011146](http://www.ebi.ac.uk/interpro/entry/IPR011146) | DOMAIN | HIT-like domain | GO:0003824 | [PS51084(PROSITE_PROFILES)](http://www.ebi.ac.uk/interpro/signature/PS51084) |
|  | [IPR026963](http://www.ebi.ac.uk/interpro/entry/IPR026963) | FAMILY | Aprataxin | GO:0003677, GO:0006281, GO:0033699 | [PTHR12486:SF4 (PANTHER)](http://www.ebi.ac.uk/interpro/signature/PTHR12486) |
|  | [IPR032566](http://www.ebi.ac.uk/interpro/entry/IPR032566) | DOMAIN | Aprataxin, C2HE/C2H2/C2HC zinc finger |  | [PF16278 (PFAM)](http://www.ebi.ac.uk/interpro/signature/PF16278) |
|  | [IPR036265](http://www.ebi.ac.uk/interpro/entry/IPR036265) | HOMOLOGOUS_SUPERFAMILY | HIT-like superfamily |  | [G3DSA:3.30.428.10(GENE3D)](http://www.ebi.ac.uk/interpro/signature/G3DSA:3.30.428.10) |
|  |  |  |  |  | [SSF54197(SUPERFAMILY)](http://www.ebi.ac.uk/interpro/signature/SSF54197) |
|  | no IPR | DOMAIN | Aprataxin, C2HE/C2H2/C2HC zinc finger |  | [PTHR12486 (PANTHER)](http://www.ebi.ac.uk/interpro/signature/PTHR12486) |
|  | no IPR | HOMOLOGOUS_SUPERFAMILY | HIT-like superfamily |  | [PF11969 (PFAM)](http://www.ebi.ac.uk/interpro/signature/PF11969) |
| *RsASCC3a* | [IPR001650](http://www.ebi.ac.uk/interpro/entry/IPR001650) | DOMAIN | Helicase, C-terminal |  | [PS51194(PROSITE_PROFILES)](http://www.ebi.ac.uk/interpro/signature/PS51194) |
|  | [IPR004179](http://www.ebi.ac.uk/interpro/entry/IPR004179) | DOMAIN | Sec63 domain |  | [SM00973 (SMART)](http://www.ebi.ac.uk/interpro/signature/SM00973) |
|  |  |  |  |  | [PF02889 (PFAM)](http://www.ebi.ac.uk/interpro/signature/PF02889) |
|  | [IPR011545](http://www.ebi.ac.uk/interpro/entry/IPR011545) | DOMAIN | DEAD/DEAH box helicase domain | GO:0005524, GO:0003676 | [PF00270 (PFAM)](http://www.ebi.ac.uk/interpro/signature/PF00270) |
|  | [IPR014001](http://www.ebi.ac.uk/interpro/entry/IPR014001) | DOMAIN | Helicase superfamily 1/2, ATP-binding domain |  | [PS51192(PROSITE_PROFILES)](http://www.ebi.ac.uk/interpro/signature/PS51192) |
|  |  |  |  |  | [PS51192(PROSITE_PROFILES)](http://www.ebi.ac.uk/interpro/signature/PS51192) |
|  | [IPR027417](http://www.ebi.ac.uk/interpro/entry/IPR027417) | HOMOLOGOUS_SUPERFAMILY | P-loop containing nucleoside triphosphate hydrolase |  | [SSF52540(SUPERFAMILY)](http://www.ebi.ac.uk/interpro/signature/SSF52540) |
|  |  |  |  |  | [SSF52540(SUPERFAMILY)](http://www.ebi.ac.uk/interpro/signature/SSF52540) |
|  |  |  |  |  | [SSF52540(SUPERFAMILY)](http://www.ebi.ac.uk/interpro/signature/SSF52540) |
|  | [IPR035892](http://www.ebi.ac.uk/interpro/entry/IPR035892) | HOMOLOGOUS_SUPERFAMILY | C2 domain superfamily |  | [G3DSA:2.60.40.150(GENE3D)](http://www.ebi.ac.uk/interpro/signature/G3DSA:2.60.40.150) |
|  |  |  |  |  | [G3DSA:2.60.40.150(GENE3D)](http://www.ebi.ac.uk/interpro/signature/G3DSA:2.60.40.150) |
|  | no IPR | HOMOLOGOUS_SUPERFAMILY | C2 domain superfamily |  | [G3DSA:1.10.3380.10(GENE3D)](http://www.ebi.ac.uk/interpro/signature/G3DSA:1.10.3380.10) |
|  | no IPR | DOMAIN | DEAD/DEAH box helicase domain |  | [G3DSA:3.40.50.300(GENE3D)](http://www.ebi.ac.uk/interpro/signature/G3DSA:3.40.50.300) |
|  | no IPR | DOMAIN | Helicase, C-terminal |  | cd00046 (CDD) |
|  |  |  |  |  | [SSF158702(SUPERFAMILY)](http://www.ebi.ac.uk/interpro/signature/SSF158702) |
|  | no IPR | HOMOLOGOUS_SUPERFAMILY | P-loop containing nucleoside triphosphate hydrolase |  | [SSF158702(SUPERFAMILY)](http://www.ebi.ac.uk/interpro/signature/SSF158702) |
|  | no IPR | DOMAIN | Sec63 domain |  | [G3DSA:1.10.10.2530(GENE3D)](http://www.ebi.ac.uk/interpro/signature/G3DSA:1.10.10.2530) |
|  |  |  |  |  | [G3DSA:3.40.50.300(GENE3D)](http://www.ebi.ac.uk/interpro/signature/G3DSA:3.40.50.300) |
|  |  |  |  |  | [G3DSA:3.40.50.300(GENE3D)](http://www.ebi.ac.uk/interpro/signature/G3DSA:3.40.50.300) |
|  |  |  |  |  | [G3DSA:1.10.3380.10(GENE3D)](http://www.ebi.ac.uk/interpro/signature/G3DSA:1.10.3380.10) |
|  |  |  |  |  | [G3DSA:3.40.50.300(GENE3D)](http://www.ebi.ac.uk/interpro/signature/G3DSA:3.40.50.300) |
|  |  |  |  |  | [G3DSA:1.10.10.2530(GENE3D)](http://www.ebi.ac.uk/interpro/signature/G3DSA:1.10.10.2530) |
|  |  |  |  |  | [PTHR24075 (PANTHER)](http://www.ebi.ac.uk/interpro/signature/PTHR24075) |
|  |  |  |  |  | [PTHR24075 (PANTHER)](http://www.ebi.ac.uk/interpro/signature/PTHR24075) |
|  |  |  |  |  | [PTHR24075:SF6 (PANTHER)](http://www.ebi.ac.uk/interpro/signature/PTHR24075) |
|  |  |  |  |  | [PTHR24075:SF6 (PANTHER)](http://www.ebi.ac.uk/interpro/signature/PTHR24075) |
| *RsASCC3b* | [IPR001650](http://www.ebi.ac.uk/interpro/entry/IPR001650) | DOMAIN | Helicase, C-terminal |  | [SM00490 (SMART)](http://www.ebi.ac.uk/interpro/signature/SM00490) |
|  |  |  |  |  | [PF00271 (PFAM)](http://www.ebi.ac.uk/interpro/signature/PF00271) |
|  |  |  |  |  | [PS51194(PROSITE_PROFILES)](http://www.ebi.ac.uk/interpro/signature/PS51194) |
|  |  |  |  |  | [PS51194(PROSITE_PROFILES)](http://www.ebi.ac.uk/interpro/signature/PS51194) |
|  |  |  |  |  | cd00079 (CDD) |
|  |  |  |  |  | cd00079 (CDD) |
|  | [IPR003593](http://www.ebi.ac.uk/interpro/entry/IPR003593) | DOMAIN | AAA+ ATPase domain |  | [SM00382 (SMART)](http://www.ebi.ac.uk/interpro/signature/SM00382) |
|  | [IPR004179](http://www.ebi.ac.uk/interpro/entry/IPR004179) | DOMAIN | Sec63 domain |  | [SM00973 (SMART)](http://www.ebi.ac.uk/interpro/signature/SM00973) |
|  |  |  |  |  | [PF02889 (PFAM)](http://www.ebi.ac.uk/interpro/signature/PF02889) |
|  | [IPR011545](http://www.ebi.ac.uk/interpro/entry/IPR011545) | DOMAIN | DEAD/DEAH box helicase domain | GO:0003676, GO:0005524 | [PF00270 (PFAM)](http://www.ebi.ac.uk/interpro/signature/PF00270) |
|  | [IPR014001](http://www.ebi.ac.uk/interpro/entry/IPR014001) | DOMAIN | Helicase superfamily 1/2, ATP-binding domain |  | [SM00487 (SMART)](http://www.ebi.ac.uk/interpro/signature/SM00487) |
|  |  |  |  |  | [PS51192(PROSITE_PROFILES)](http://www.ebi.ac.uk/interpro/signature/PS51192) |
|  |  |  |  |  | [PS51192(PROSITE_PROFILES)](http://www.ebi.ac.uk/interpro/signature/PS51192) |
|  | [IPR014756](http://www.ebi.ac.uk/interpro/entry/IPR014756) | HOMOLOGOUS_SUPERFAMILY | Immunoglobulin E-set |  | [SSF81296(SUPERFAMILY)](http://www.ebi.ac.uk/interpro/signature/SSF81296) |
|  | [IPR027417](http://www.ebi.ac.uk/interpro/entry/IPR027417) | HOMOLOGOUS_SUPERFAMILY | P-loop containing nucleoside triphosphate hydrolase |  | [SSF52540(SUPERFAMILY)](http://www.ebi.ac.uk/interpro/signature/SSF52540) |
|  |  |  |  |  | [SSF52540(SUPERFAMILY)](http://www.ebi.ac.uk/interpro/signature/SSF52540) |
|  |  |  |  |  | [SSF52540(SUPERFAMILY)](http://www.ebi.ac.uk/interpro/signature/SSF52540) |
|  |  |  |  |  | [SSF52540(SUPERFAMILY)](http://www.ebi.ac.uk/interpro/signature/SSF52540) |
|  | [IPR035892](http://www.ebi.ac.uk/interpro/entry/IPR035892) | HOMOLOGOUS_SUPERFAMILY | C2 domain superfamily |  | [G3DSA:2.60.40.150(GENE3D)](http://www.ebi.ac.uk/interpro/signature/G3DSA:2.60.40.150) |
|  |  |  |  |  | [G3DSA:2.60.40.150(GENE3D)](http://www.ebi.ac.uk/interpro/signature/G3DSA:2.60.40.150) |
|  | [IPR036390](http://www.ebi.ac.uk/interpro/entry/IPR036390) | HOMOLOGOUS_SUPERFAMILY | Winged helix DNA-binding domain superfamily |  | [SSF46785(SUPERFAMILY)](http://www.ebi.ac.uk/interpro/signature/SSF46785) |
|  |  |  |  |  | [SSF46785(SUPERFAMILY)](http://www.ebi.ac.uk/interpro/signature/SSF46785) |
|  | no IPR | HOMOLOGOUS_SUPERFAMILY | C2 domain superfamily |  | PIRSF039073 (PIRSF) |
|  |  |  |  |  | [G3DSA:1.10.10.2530(GENE3D)](http://www.ebi.ac.uk/interpro/signature/G3DSA:1.10.10.2530) |
|  |  |  |  |  | [G3DSA:1.10.10.2530(GENE3D)](http://www.ebi.ac.uk/interpro/signature/G3DSA:1.10.10.2530) |
|  |  |  |  |  | [G3DSA:1.10.3380.10(GENE3D)](http://www.ebi.ac.uk/interpro/signature/G3DSA:1.10.3380.10) |
|  | no IPR | DOMAIN | DEAD/DEAH box helicase domain |  | [PTHR24075 (PANTHER)](http://www.ebi.ac.uk/interpro/signature/PTHR24075) |
|  |  |  |  |  | [PTHR24075:SF6 (PANTHER)](http://www.ebi.ac.uk/interpro/signature/PTHR24075) |
|  | no IPR | DOMAIN | Helicase, C-terminal |  | [G3DSA:1.10.150.20(GENE3D)](http://www.ebi.ac.uk/interpro/signature/G3DSA:1.10.150.20) |
|  |  |  |  |  | [G3DSA:3.40.50.300(GENE3D)](http://www.ebi.ac.uk/interpro/signature/G3DSA:3.40.50.300) |
|  |  |  |  |  | [G3DSA:3.40.50.300(GENE3D)](http://www.ebi.ac.uk/interpro/signature/G3DSA:3.40.50.300) |
|  |  |  |  |  | [G3DSA:3.40.50.300(GENE3D)](http://www.ebi.ac.uk/interpro/signature/G3DSA:3.40.50.300) |
|  |  |  |  |  | [G3DSA:1.10.3380.10(GENE3D)](http://www.ebi.ac.uk/interpro/signature/G3DSA:1.10.3380.10) |
|  |  |  |  |  | cd00046 (CDD) |
|  |  |  |  |  | cd00046 (CDD) |
|  | no IPR | HOMOLOGOUS_SUPERFAMILY | P-loop containing nucleoside triphosphate hydrolase |  | [SSF158702(SUPERFAMILY)](http://www.ebi.ac.uk/interpro/signature/SSF158702) |
|  |  |  |  |  | [SSF158702(SUPERFAMILY)](http://www.ebi.ac.uk/interpro/signature/SSF158702) |
|  | no IPR | DOMAIN | Sec63 domain |  | [G3DSA:3.40.50.300(GENE3D)](http://www.ebi.ac.uk/interpro/signature/G3DSA:3.40.50.300) |
|  | no IPR |  |  |  | Coil (COILS) |
| *RsATM* | [IPR015519](http://www.ebi.ac.uk/interpro/entry/IPR015519) | FAMILY | Serine/threonine-protein kinase ATM | GO:0006281, GO:0000077, GO:0000723, GO:0010212, GO:0090399, GO:0016572, GO:0004674, GO:0006974 | [PTHR11139:SF96 (PANTHER)](http://www.ebi.ac.uk/interpro/signature/PTHR11139) |
|  | [IPR021668](http://www.ebi.ac.uk/interpro/entry/IPR021668) | DOMAIN | Telomere-length maintenance and DNA damage repair | GO:0004674 | [SM01342(SMART)](http://www.ebi.ac.uk/interpro/signature/SM01342) |
|  |  |  |  |  | [PF11640 (PFAM)](http://www.ebi.ac.uk/interpro/signature/PF11640) |
|  | no IPR | DOMAIN | Telomere-length maintenance and DNA damage repair |  | [PTHR11139(PANTHER)](http://www.ebi.ac.uk/interpro/signature/PTHR11139) |
|  | no IPR |  |  |  | Coil (COILS) |
| *RsATR* | [IPR000403](http://www.ebi.ac.uk/interpro/entry/IPR000403) | DOMAIN | Phosphatidylinositol 3-/4-kinase, catalytic domain |  | [SM00146 (SMART)](http://www.ebi.ac.uk/interpro/signature/SM00146) |
|  |  |  |  |  | [PF00454 (PFAM)](http://www.ebi.ac.uk/interpro/signature/PF00454) |
|  |  |  |  |  | [PS50290(PROSITE_PROFILES)](http://www.ebi.ac.uk/interpro/signature/PS50290) |
|  | [IPR003151](http://www.ebi.ac.uk/interpro/entry/IPR003151) | DOMAIN | PIK-related kinase, FAT | GO:0005515 | [PF02259 (PFAM)](http://www.ebi.ac.uk/interpro/signature/PF02259) |
|  | [IPR003152](http://www.ebi.ac.uk/interpro/entry/IPR003152) | DOMAIN | FATC domain | GO:0005515 | [SM01343 (SMART)](http://www.ebi.ac.uk/interpro/signature/SM01343) |
|  |  |  |  |  | [PF02260 (PFAM)](http://www.ebi.ac.uk/interpro/signature/PF02260) |
|  |  |  |  |  | [PS51190(PROSITE_PROFILES)](http://www.ebi.ac.uk/interpro/signature/PS51190) |
|  | [IPR011009](http://www.ebi.ac.uk/interpro/entry/IPR011009) | HOMOLOGOUS_SUPERFAMILY | Protein kinase-like domain superfamily |  | [SSF56112 (SUPERFAMILY)](http://www.ebi.ac.uk/interpro/signature/SSF56112) |
|  | [IPR011990](http://www.ebi.ac.uk/interpro/entry/IPR011990) | HOMOLOGOUS_SUPERFAMILY | Tetratricopeptide-like helical domain superfamily | GO:0005515 | [SSF48452 (SUPERFAMILY)](http://www.ebi.ac.uk/interpro/signature/SSF48452) |
|  | [IPR012993](http://www.ebi.ac.uk/interpro/entry/IPR012993) | DOMAIN | UME domain | GO:0004674 | [SM00802 (SMART)](http://www.ebi.ac.uk/interpro/signature/SM00802) |
|  |  |  |  |  | [PF08064 (PFAM)](http://www.ebi.ac.uk/interpro/signature/PF08064) |
|  | [IPR014009](http://www.ebi.ac.uk/interpro/entry/IPR014009) | DOMAIN | PIK-related kinase |  | [PS51189(PROSITE_PROFILES)](http://www.ebi.ac.uk/interpro/signature/PS51189) |
|  | [IPR016024](http://www.ebi.ac.uk/interpro/entry/IPR016024) | HOMOLOGOUS_SUPERFAMILY | Armadillo-type fold | GO:0005488 | [SSF48371 (SUPERFAMILY)](http://www.ebi.ac.uk/interpro/signature/SSF48371) |
|  | [IPR018936](http://www.ebi.ac.uk/interpro/entry/IPR018936) | CONSERVED_SITE | Phosphatidylinositol 3/4-kinase, conserved site | GO:0016301 | [PS00916(PROSITE_PATTERNS)](http://www.ebi.ac.uk/interpro/signature/PS00916) |
|  | [IPR036940](http://www.ebi.ac.uk/interpro/entry/IPR036940) | HOMOLOGOUS_SUPERFAMILY | Phosphatidylinositol 3-/4-kinase, catalytic domain superfamily | GO:0016301 | [G3DSA:1.10.1070.11 (GENE3D)](http://www.ebi.ac.uk/interpro/signature/G3DSA:1.10.1070.11) |
|  | no IPR | DOMAIN | FATC domain |  | [G3DSA:3.30.1010.10 (GENE3D)](http://www.ebi.ac.uk/interpro/signature/G3DSA:3.30.1010.10) |
|  |  |  |  |  | [PTHR11139:SF69 (PANTHER)](http://www.ebi.ac.uk/interpro/signature/PTHR11139) |
|  |  |  |  |  | [PTHR11139 (PANTHER)](http://www.ebi.ac.uk/interpro/signature/PTHR11139) |
|  | no IPR | DOMAIN | Phosphatidylinositol 3-/4-kinase, catalytic domain |  | cd00892 (CDD) |
|  | no IPR | CONSERVED_SITE | Phosphatidylinositol 3/4-kinase, conserved site |  | TRANSMEMBRANE (PHOBIUS) |
|  |  |  |  |  | CYTOPLASMIC_DOMAIN (PHOBIUS) |
|  |  |  |  |  | NON_CYTOPLASMIC_DOMAIN (PHOBIUS) |
|  | no IPR |  |  |  | Coil (COILS) |
| *RsBARD1* | [IPR001357](http://www.ebi.ac.uk/interpro/entry/IPR001357) | DOMAIN | BRCT domain |  | [SM00292 (SMART)](http://www.ebi.ac.uk/interpro/signature/SM00292) |
|  |  |  |  |  | [PS50172(PROSITE_PROFILES)](http://www.ebi.ac.uk/interpro/signature/PS50172) |
|  |  |  |  |  | [PS50172(PROSITE_PROFILES)](http://www.ebi.ac.uk/interpro/signature/PS50172) |
|  |  |  |  |  | cd00027 (CDD) |
|  | [IPR001841](http://www.ebi.ac.uk/interpro/entry/IPR001841) | DOMAIN | Zinc finger, RING-type |  | [SM00184 (SMART)](http://www.ebi.ac.uk/interpro/signature/SM00184) |
|  |  |  |  |  | [PS50089(PROSITE_PROFILES)](http://www.ebi.ac.uk/interpro/signature/PS50089) |
|  | [IPR002110](http://www.ebi.ac.uk/interpro/entry/IPR002110) | REPEAT | Ankyrin repeat | GO:0005515 | [SM00248 (SMART)](http://www.ebi.ac.uk/interpro/signature/SM00248) |
|  |  |  |  |  | [PS50088(PROSITE_PROFILES)](http://www.ebi.ac.uk/interpro/signature/PS50088) |
|  |  |  |  |  | [PS50088(PROSITE_PROFILES)](http://www.ebi.ac.uk/interpro/signature/PS50088) |
|  |  |  |  |  | [PS50088(PROSITE_PROFILES)](http://www.ebi.ac.uk/interpro/signature/PS50088) |
|  | [IPR013083](http://www.ebi.ac.uk/interpro/entry/IPR013083) | HOMOLOGOUS_SUPERFAMILY | Zinc finger, RING/FYVE/PHD-type |  | [G3DSA:3.30.40.10(GENE3D)](http://www.ebi.ac.uk/interpro/signature/G3DSA:3.30.40.10) |
|  | [IPR017907](http://www.ebi.ac.uk/interpro/entry/IPR017907) | CONSERVED_SITE | Zinc finger, RING-type, conserved site |  | [PS00518(PROSITE_PATTERNS)](http://www.ebi.ac.uk/interpro/signature/PS00518) |
|  | [IPR020683](http://www.ebi.ac.uk/interpro/entry/IPR020683) | DOMAIN | Ankyrin repeat-containing domain |  | [PF12796 (PFAM)](http://www.ebi.ac.uk/interpro/signature/PF12796) |
|  |  |  |  |  | [PS50297(PROSITE_PROFILES)](http://www.ebi.ac.uk/interpro/signature/PS50297) |
|  |  |  |  |  | cd00204 (CDD) |
|  | [IPR033097](http://www.ebi.ac.uk/interpro/entry/IPR033097) | FAMILY | BRCA1-associated RING domain protein 1 | GO:0031436, GO:0043065, GO:0016567 | [PTHR24171:SF8 (PANTHER)](http://www.ebi.ac.uk/interpro/signature/PTHR24171) |
|  |  |  |  |  | [PTHR24171:SF8 (PANTHER)](http://www.ebi.ac.uk/interpro/signature/PTHR24171) |
|  | [IPR036420](http://www.ebi.ac.uk/interpro/entry/IPR036420) | HOMOLOGOUS_SUPERFAMILY | BRCT domain superfamily |  | [G3DSA:3.40.50.10190 (GENE3D)](http://www.ebi.ac.uk/interpro/signature/G3DSA:3.40.50.1019) |
|  |  |  |  |  | [G3DSA:3.40.50.10190 (GENE3D)](http://www.ebi.ac.uk/interpro/signature/G3DSA:3.40.50.1019) |
|  |  |  |  |  | [SSF52113(SUPERFAMILY)](http://www.ebi.ac.uk/interpro/signature/SSF52113) |
|  |  |  |  |  | [SSF52113(SUPERFAMILY)](http://www.ebi.ac.uk/interpro/signature/SSF52113) |
|  | [IPR036770](http://www.ebi.ac.uk/interpro/entry/IPR036770) | HOMOLOGOUS_SUPERFAMILY | Ankyrin repeat-containing domain superfamily |  | [G3DSA:1.25.40.20(GENE3D)](http://www.ebi.ac.uk/interpro/signature/G3DSA:1.25.40.20) |
|  |  |  |  |  | [SSF48403(SUPERFAMILY)](http://www.ebi.ac.uk/interpro/signature/SSF48403) |
|  | no IPR | HOMOLOGOUS_SUPERFAMILY | Ankyrin repeat-containing domain superfamily |  | mobidb-lite (MOBIDB_LITE) |
|  |  |  |  |  | [PTHR24171 (PANTHER)](http://www.ebi.ac.uk/interpro/signature/PTHR24171) |
|  |  |  |  |  | [PTHR24171 (PANTHER)](http://www.ebi.ac.uk/interpro/signature/PTHR24171) |
|  |  |  |  |  | [SSF57850(SUPERFAMILY)](http://www.ebi.ac.uk/interpro/signature/SSF57850) |
|  | no IPR |  |  |  | Coil (COILS) |
| *RsBLM* | [IPR004273](http://www.ebi.ac.uk/interpro/entry/IPR004273) | DOMAIN | Dynein heavy chain domain | GO:0003777, GO:0007018, GO:0030286 | [PF03028 (PFAM)](http://www.ebi.ac.uk/interpro/signature/PF03028) |
|  | no IPR | DOMAIN | Dynein heavy chain domain |  | [PTHR45129 (PANTHER)](http://www.ebi.ac.uk/interpro/signature/PTHR45129) |
|  | no IPR |  |  |  | [G3DSA:3.10.490.20 (GENE3D)](http://www.ebi.ac.uk/interpro/signature/G3DSA:3.10.490.20) |
| *RsBRCA1* | [IPR001357](http://www.ebi.ac.uk/interpro/entry/IPR001357) | DOMAIN | BRCT domain |  | [SM00292 (SMART)](http://www.ebi.ac.uk/interpro/signature/SM00292) |
|  |  |  |  |  | [PS50172 (PROSITE_PROFILES)](http://www.ebi.ac.uk/interpro/signature/PS50172) |
|  |  |  |  |  | [PS50172 (PROSITE_PROFILES)](http://www.ebi.ac.uk/interpro/signature/PS50172) |
|  |  |  |  |  | cd00027 (CDD) |
|  | [IPR031099](http://www.ebi.ac.uk/interpro/entry/IPR031099) | FAMILY | BRCA1-associated | GO:0006974, GO:0006281 | [PTHR13763 (PANTHER)](http://www.ebi.ac.uk/interpro/signature/PTHR13763) |
|  | [IPR036420](http://www.ebi.ac.uk/interpro/entry/IPR036420) | HOMOLOGOUS_SUPERFAMILY | BRCT domain superfamily |  | [G3DSA:3.40.50.10190 (GENE3D)](http://www.ebi.ac.uk/interpro/signature/G3DSA:3.40.50.1019) |
|  |  |  |  |  | [G3DSA:3.40.50.10190 (GENE3D)](http://www.ebi.ac.uk/interpro/signature/G3DSA:3.40.50.1019) |
|  |  |  |  |  | [SSF52113 (SUPERFAMILY)](http://www.ebi.ac.uk/interpro/signature/SSF52113) |
|  |  |  |  |  | [SSF52113 (SUPERFAMILY)](http://www.ebi.ac.uk/interpro/signature/SSF52113) |
|  | no IPR | HOMOLOGOUS_SUPERFAMILY | BRCT domain superfamily |  | mobidb-lite (MOBIDB_LITE) |
|  |  |  |  |  | mobidb-lite (MOBIDB_LITE) |
|  |  |  |  |  | mobidb-lite (MOBIDB_LITE) |
|  |  |  |  |  | mobidb-lite (MOBIDB_LITE) |
|  | no IPR |  |  |  | Coil (COILS) |
| *RsBRCA2* | no IPS match | |  |  |  |
| *RsBRCC3* | [IPR000555](http://www.ebi.ac.uk/interpro/entry/IPR000555) | DOMAIN | JAB1/MPN/MOV34 metalloenzyme domain | GO:0005515 | [SM00232 (SMART)](http://www.ebi.ac.uk/interpro/signature/SM00232) |
|  |  |  |  |  | [PF01398 (PFAM)](http://www.ebi.ac.uk/interpro/signature/PF01398) |
|  | [IPR033860](http://www.ebi.ac.uk/interpro/entry/IPR033860) | FAMILY | Brcc36 isopeptidase | GO:0004843, GO:0006281, GO:0070552, GO:0070531, GO:0070536 | cd08068 (CDD) |
|  | [IPR037518](http://www.ebi.ac.uk/interpro/entry/IPR037518) | DOMAIN | MPN domain |  | [PS50249(PROSITE_PROFILES)](http://www.ebi.ac.uk/interpro/signature/PS50249) |
|  | no IPR | FAMILY | Brcc36 isopeptidase |  | [SSF102712(SUPERFAMILY)](http://www.ebi.ac.uk/interpro/signature/SSF102712) |
|  | no IPR | DOMAIN | JAB1/MPN/MOV34 metalloenzyme domain |  | [G3DSA:3.40.140.10(GENE3D)](http://www.ebi.ac.uk/interpro/signature/G3DSA:3.40.140.10) |
| *RsBRE* | [IPR010358](http://www.ebi.ac.uk/interpro/entry/IPR010358) | FAMILY | BRCA1-A complex subunit BRE | GO:0070531, GO:0070552 | [PF06113 (PFAM)](http://www.ebi.ac.uk/interpro/signature/PF06113) |
|  |  |  |  |  | [PTHR15189 (PANTHER)](http://www.ebi.ac.uk/interpro/signature/PTHR15189) |
| *RsBRIP1* | [IPR006554](http://www.ebi.ac.uk/interpro/entry/IPR006554) | DOMAIN | Helicase-like, DEXD box c2 type | GO:0004003, GO:0016818 | [SM00488 (SMART)](http://www.ebi.ac.uk/interpro/signature/SM00488) |
|  | [IPR006555](http://www.ebi.ac.uk/interpro/entry/IPR006555) | DOMAIN | ATP-dependent helicase, C-terminal | GO:0006139, GO:0005524, GO:0016818, GO:0008026, GO:0003676 | [SM00491 (SMART)](http://www.ebi.ac.uk/interpro/signature/SM00491) |
|  |  |  |  |  | [PF13307 (PFAM)](http://www.ebi.ac.uk/interpro/signature/PF13307) |
|  | [IPR010614](http://www.ebi.ac.uk/interpro/entry/IPR010614) | DOMAIN | DEAD2 | GO:0003677, GO:0005524, GO:0004003 | [PF06733 (PFAM)](http://www.ebi.ac.uk/interpro/signature/PF06733) |
|  | [IPR013020](http://www.ebi.ac.uk/interpro/entry/IPR013020) | FAMILY | ATP-dependent helicase Rad3/Chl1-like | GO:0004003 | [TIGR00604(TIGRFAM)](http://www.ebi.ac.uk/interpro/signature/TIGR00604) |
|  | [IPR014013](http://www.ebi.ac.uk/interpro/entry/IPR014013) | DOMAIN | Helicase superfamily 1/2, ATP-binding domain, DinG/Rad3-type | GO:0005524 | [PS51193(PROSITE_PROFILES)](http://www.ebi.ac.uk/interpro/signature/PS51193) |
|  | [IPR027417](http://www.ebi.ac.uk/interpro/entry/IPR027417) | HOMOLOGOUS_SUPERFAMILY | P-loop containing nucleoside triphosphate hydrolase |  | [SSF52540(SUPERFAMILY)](http://www.ebi.ac.uk/interpro/signature/SSF52540) |
|  | no IPR | FAMILY | ATP-dependent helicase Rad3/Chl1-like |  | [G3DSA:3.40.50.300(GENE3D)](http://www.ebi.ac.uk/interpro/signature/G3DSA:3.40.50.300) |
|  |  |  |  |  | mobidb-lite (MOBIDB_LITE) |
|  |  |  |  |  | mobidb-lite (MOBIDB_LITE) |
|  |  |  |  |  | [PTHR11472(PANTHER)](http://www.ebi.ac.uk/interpro/signature/PTHR11472) |
|  |  |  |  |  | [PTHR11472:SF47 (PANTHER)](http://www.ebi.ac.uk/interpro/signature/PTHR11472) |
|  | no IPR | DOMAIN | ATP-dependent helicase, C-terminal |  | [G3DSA:3.40.50.300(GENE3D)](http://www.ebi.ac.uk/interpro/signature/G3DSA:3.40.50.300) |
|  |  |  |  |  | [G3DSA:3.40.50.300(GENE3D)](http://www.ebi.ac.uk/interpro/signature/G3DSA:3.40.50.300) |
|  | no IPR |  |  |  | Coil (COILS) |
| *RsCDC6* | [IPR003593](http://www.ebi.ac.uk/interpro/entry/IPR003593) | DOMAIN | AAA+ ATPase domain |  | [SM00382 (SMART)](http://www.ebi.ac.uk/interpro/signature/SM00382) |
|  |  |  |  |  | [PF13401 (PFAM)](http://www.ebi.ac.uk/interpro/signature/PF13401) |
|  | [IPR015163](http://www.ebi.ac.uk/interpro/entry/IPR015163) | DOMAIN | Cdc6, C-terminal |  | [SM01074 (SMART)](http://www.ebi.ac.uk/interpro/signature/SM01074) |
|  |  |  |  |  | [PF09079 (PFAM)](http://www.ebi.ac.uk/interpro/signature/PF09079) |
|  |  |  |  |  | cd08768 (CDD) |
|  | [IPR016314](http://www.ebi.ac.uk/interpro/entry/IPR016314) | FAMILY | Cell division protein Cdc6/18 | GO:0051301, GO:0006270 | PIRSF001767 (PIRSF) |
|  | [IPR027417](http://www.ebi.ac.uk/interpro/entry/IPR027417) | HOMOLOGOUS_SUPERFAMILY | P-loop containing nucleoside triphosphate hydrolase |  | [SSF52540 (SUPERFAMILY)](http://www.ebi.ac.uk/interpro/signature/SSF52540) |
|  | [IPR036388](http://www.ebi.ac.uk/interpro/entry/IPR036388) | HOMOLOGOUS_SUPERFAMILY | Winged helix-like DNA-binding domain superfamily |  | [G3DSA:1.10.10.10 (GENE3D)](http://www.ebi.ac.uk/interpro/signature/G3DSA:1.10.10.10) |
|  | [IPR036390](http://www.ebi.ac.uk/interpro/entry/IPR036390) | HOMOLOGOUS_SUPERFAMILY | Winged helix DNA-binding domain superfamily |  | [SSF46785 (SUPERFAMILY)](http://www.ebi.ac.uk/interpro/signature/SSF46785) |
|  | no IPR | DOMAIN | AAA+ ATPase domain |  | [G3DSA:1.10.8.60 (GENE3D)](http://www.ebi.ac.uk/interpro/signature/G3DSA:1.10.8.60) |
|  | no IPR | DOMAIN | Cdc6, C-terminal |  | mobidb-lite (MOBIDB_LITE) |
|  |  |  |  |  | mobidb-lite (MOBIDB_LITE) |
|  |  |  |  |  | [PTHR10763 (PANTHER)](http://www.ebi.ac.uk/interpro/signature/PTHR10763) |
|  |  |  |  |  | [PTHR10763:SF26 (PANTHER)](http://www.ebi.ac.uk/interpro/signature/PTHR10763) |
|  |  |  |  |  | NON_CYTOPLASMIC_DOMAIN (PHOBIUS) |
|  |  |  |  |  | SIGNAL_PEPTIDE_H_REGION (PHOBIUS) |
|  |  |  |  |  | SIGNAL_PEPTIDE_C_REGION (PHOBIUS) |
|  |  |  |  |  | SIGNAL_PEPTIDE_N_REGION (PHOBIUS) |
|  |  |  |  |  | SIGNAL_PEPTIDE (PHOBIUS) |
|  |  |  |  |  | cd00009 (CDD) |
|  | no IPR | FAMILY | Cell division protein Cdc6/18 |  | [G3DSA:3.40.50.300 (GENE3D)](http://www.ebi.ac.uk/interpro/signature/G3DSA:3.40.50.300) |
| *RsCDK1* | [IPR000719](http://www.ebi.ac.uk/interpro/entry/IPR000719) | DOMAIN | Protein kinase domain | GO:0005524, GO:0004672, GO:0006468 | [SM00220 (SMART)](http://www.ebi.ac.uk/interpro/signature/SM00220) |
|  |  |  |  |  | [PF00069 (PFAM)](http://www.ebi.ac.uk/interpro/signature/PF00069) |
|  |  |  |  |  | [PS50011(PROSITE_PROFILES)](http://www.ebi.ac.uk/interpro/signature/PS50011) |
|  | [IPR008271](http://www.ebi.ac.uk/interpro/entry/IPR008271) | ACTIVE_SITE | Serine/threonine-protein kinase, active site | GO:0006468, GO:0004672 | [PS00108(PROSITE_PATTERNS)](http://www.ebi.ac.uk/interpro/signature/PS00108) |
|  | [IPR011009](http://www.ebi.ac.uk/interpro/entry/IPR011009) | HOMOLOGOUS_SUPERFAMILY | Protein kinase-like domain superfamily |  | [SSF56112(SUPERFAMILY)](http://www.ebi.ac.uk/interpro/signature/SSF56112) |
|  | [IPR017441](http://www.ebi.ac.uk/interpro/entry/IPR017441) | BINDING_SITE | Protein kinase, ATP binding site | GO:0005524 | [PS00107(PROSITE_PATTERNS)](http://www.ebi.ac.uk/interpro/signature/PS00107) |
|  | no IPR | DOMAIN | Protein kinase domain |  | [G3DSA:1.10.510.10(GENE3D)](http://www.ebi.ac.uk/interpro/signature/G3DSA:1.10.510.10) |
|  |  |  |  |  | PIRSF000654 (PIRSF) |
|  |  |  |  |  | [G3DSA:3.30.200.20(GENE3D)](http://www.ebi.ac.uk/interpro/signature/G3DSA:3.30.200.20) |
|  |  |  |  |  | [PTHR24056:SF334 (PANTHER)](http://www.ebi.ac.uk/interpro/signature/PTHR24056) |
|  |  |  |  |  | [PTHR24056 (PANTHER)](http://www.ebi.ac.uk/interpro/signature/PTHR24056) |
|  |  |  |  |  | cd07861 (CDD) |
| *RsCDK2* | [IPR000719](http://www.ebi.ac.uk/interpro/entry/IPR000719) | DOMAIN | Protein kinase domain | GO:0004672, GO:0006468, GO:0005524 | [SM00220 (SMART)](http://www.ebi.ac.uk/interpro/signature/SM00220) |
|  |  |  |  |  | [PF00069 (PFAM)](http://www.ebi.ac.uk/interpro/signature/PF00069) |
|  |  |  |  |  | [PS50011(PROSITE_PROFILES)](http://www.ebi.ac.uk/interpro/signature/PS50011) |
|  | [IPR008271](http://www.ebi.ac.uk/interpro/entry/IPR008271) | ACTIVE_SITE | Serine/threonine-protein kinase, active site | GO:0006468, GO:0004672 | [PS00108(PROSITE_PATTERNS)](http://www.ebi.ac.uk/interpro/signature/PS00108) |
|  | [IPR011009](http://www.ebi.ac.uk/interpro/entry/IPR011009) | HOMOLOGOUS_SUPERFAMILY | Protein kinase-like domain superfamily |  | [SSF56112(SUPERFAMILY)](http://www.ebi.ac.uk/interpro/signature/SSF56112) |
|  | [IPR017441](http://www.ebi.ac.uk/interpro/entry/IPR017441) | BINDING_SITE | Protein kinase, ATP binding site | GO:0005524 | [PS00107(PROSITE_PATTERNS)](http://www.ebi.ac.uk/interpro/signature/PS00107) |
|  | no IPR | DOMAIN | Protein kinase domain |  | [G3DSA:1.10.510.10(GENE3D)](http://www.ebi.ac.uk/interpro/signature/G3DSA:1.10.510.10) |
|  |  |  |  |  | PIRSF000654 (PIRSF) |
|  |  |  |  |  | [G3DSA:3.30.200.20(GENE3D)](http://www.ebi.ac.uk/interpro/signature/G3DSA:3.30.200.20) |
|  |  |  |  |  | [PTHR24056:SF185 (PANTHER)](http://www.ebi.ac.uk/interpro/signature/PTHR24056) |
|  |  |  |  |  | [PTHR24056 (PANTHER)](http://www.ebi.ac.uk/interpro/signature/PTHR24056) |
|  |  |  |  |  | cd07835 (CDD) |
| *RsCHK1* | [IPR000719](http://www.ebi.ac.uk/interpro/entry/IPR000719) | DOMAIN | Protein kinase domain | GO:0005524, GO:0006468, GO:0004672 | [SM00220 (SMART)](http://www.ebi.ac.uk/interpro/signature/SM00220) |
|  |  |  |  |  | [PF00069 (PFAM)](http://www.ebi.ac.uk/interpro/signature/PF00069) |
|  |  |  |  |  | [PS50011(PROSITE_PROFILES)](http://www.ebi.ac.uk/interpro/signature/PS50011) |
|  | [IPR008271](http://www.ebi.ac.uk/interpro/entry/IPR008271) | ACTIVE_SITE | Serine/threonine-protein kinase, active site | GO:0006468, GO:0004672 | [PS00108(PROSITE_PATTERNS)](http://www.ebi.ac.uk/interpro/signature/PS00108) |
|  | [IPR011009](http://www.ebi.ac.uk/interpro/entry/IPR011009) | HOMOLOGOUS_SUPERFAMILY | Protein kinase-like domain superfamily |  | [SSF56112(SUPERFAMILY)](http://www.ebi.ac.uk/interpro/signature/SSF56112) |
|  | [IPR017441](http://www.ebi.ac.uk/interpro/entry/IPR017441) | BINDING_SITE | Protein kinase, ATP binding site | GO:0005524 | [PS00107(PROSITE_PATTERNS)](http://www.ebi.ac.uk/interpro/signature/PS00107) |
|  | [IPR034670](http://www.ebi.ac.uk/interpro/entry/IPR034670) | DOMAIN | Checkpoint kinase 1, catalytic domain | GO:0004674, GO:0000077, GO:0006468 | cd14069 (CDD) |
|  | no IPR | DOMAIN | Protein kinase domain |  | [G3DSA:3.30.200.20(GENE3D)](http://www.ebi.ac.uk/interpro/signature/G3DSA:3.30.200.20) |
|  |  |  |  |  | [G3DSA:1.10.510.10(GENE3D)](http://www.ebi.ac.uk/interpro/signature/G3DSA:1.10.510.10) |
|  |  |  |  |  | mobidb-lite (MOBIDB_LITE) |
|  |  |  |  |  | [PTHR45524 (PANTHER)](http://www.ebi.ac.uk/interpro/signature/PTHR45524) |
| *RsCHK2* | [IPR000253](http://www.ebi.ac.uk/interpro/entry/IPR000253) | DOMAIN | Forkhead-associated (FHA) domain | GO:0005515 | [SM00240 (SMART)](http://www.ebi.ac.uk/interpro/signature/SM00240) |
|  |  |  |  |  | [PF00498 (PFAM)](http://www.ebi.ac.uk/interpro/signature/PF00498) |
|  |  |  |  |  | [PS50006(PROSITE_PROFILES)](http://www.ebi.ac.uk/interpro/signature/PS50006) |
|  |  |  |  |  | cd00060 (CDD) |
|  | [IPR000719](http://www.ebi.ac.uk/interpro/entry/IPR000719) | DOMAIN | Protein kinase domain | GO:0004672, GO:0006468, GO:0005524 | [SM00220 (SMART)](http://www.ebi.ac.uk/interpro/signature/SM00220) |
|  |  |  |  |  | [PF00069 (PFAM)](http://www.ebi.ac.uk/interpro/signature/PF00069) |
|  |  |  |  |  | [PS50011(PROSITE_PROFILES)](http://www.ebi.ac.uk/interpro/signature/PS50011) |
|  | [IPR008271](http://www.ebi.ac.uk/interpro/entry/IPR008271) | ACTIVE_SITE | Serine/threonine-protein kinase, active site | GO:0004672, GO:0006468 | [PS00108(PROSITE_PATTERNS)](http://www.ebi.ac.uk/interpro/signature/PS00108) |
|  | [IPR008984](http://www.ebi.ac.uk/interpro/entry/IPR008984) | HOMOLOGOUS_SUPERFAMILY | SMAD/FHA domain superfamily | GO:0005515 | [SSF49879 (SUPERFAMILY)](http://www.ebi.ac.uk/interpro/signature/SSF49879) |
|  | [IPR011009](http://www.ebi.ac.uk/interpro/entry/IPR011009) | HOMOLOGOUS_SUPERFAMILY | Protein kinase-like domain superfamily |  | [SSF56112 (SUPERFAMILY)](http://www.ebi.ac.uk/interpro/signature/SSF56112) |
|  | [IPR017441](http://www.ebi.ac.uk/interpro/entry/IPR017441) | BINDING_SITE | Protein kinase, ATP binding site | GO:0005524 | [PS00107(PROSITE_PATTERNS)](http://www.ebi.ac.uk/interpro/signature/PS00107) |
|  | no IPR | DOMAIN | Protein kinase domain |  | [G3DSA:1.10.510.10 (GENE3D)](http://www.ebi.ac.uk/interpro/signature/G3DSA:1.10.510.10) |
|  |  |  |  |  | [G3DSA:2.60.200.20 (GENE3D)](http://www.ebi.ac.uk/interpro/signature/G3DSA:2.60.200.20) |
|  |  |  |  |  | mobidb-lite (MOBIDB_LITE) |
|  |  |  |  |  | [PTHR44167 (PANTHER)](http://www.ebi.ac.uk/interpro/signature/PTHR44167) |
|  | no IPR | BINDING_SITE | Protein kinase, ATP binding site |  | NON_CYTOPLASMIC_DOMAIN (PHOBIUS) |
|  |  |  |  |  | CYTOPLASMIC_DOMAIN (PHOBIUS) |
|  |  |  |  |  | TRANSMEMBRANE (PHOBIUS) |
| *RsCLASPIN* | no IPR |  |  |  | Coil (COILS) |
|  |  |  |  |  | Coil (COILS) |
|  |  |  |  |  | mobidb-lite (MOBIDB_LITE) |
|  |  |  |  |  | mobidb-lite (MOBIDB_LITE) |
|  |  |  |  |  | mobidb-lite (MOBIDB_LITE) |
|  |  |  |  |  | mobidb-lite (MOBIDB_LITE) |
| *RsCRY1* | [IPR005101](http://www.ebi.ac.uk/interpro/entry/IPR005101) | DOMAIN | Cryptochrome/DNA photolyase, FAD-binding domain |  | [PF03441 (PFAM)](http://www.ebi.ac.uk/interpro/signature/PF03441) |
|  | [IPR006050](http://www.ebi.ac.uk/interpro/entry/IPR006050) | DOMAIN | DNA photolyase, N-terminal |  | [PF00875 (PFAM)](http://www.ebi.ac.uk/interpro/signature/PF00875) |
|  |  |  |  |  | [PS51645(PROSITE_PROFILES)](http://www.ebi.ac.uk/interpro/signature/PS51645) |
|  | [IPR014729](http://www.ebi.ac.uk/interpro/entry/IPR014729) | HOMOLOGOUS_SUPERFAMILY | Rossmann-like alpha/beta/alpha sandwich fold |  | [G3DSA:3.40.50.620(GENE3D)](http://www.ebi.ac.uk/interpro/signature/G3DSA:3.40.50.620) |
|  | [IPR036134](http://www.ebi.ac.uk/interpro/entry/IPR036134) | HOMOLOGOUS_SUPERFAMILY | Cryptochrome/DNA photolyase, FAD-binding domain-like superfamily |  | [SSF48173 (SUPERFAMILY)](http://www.ebi.ac.uk/interpro/signature/SSF48173) |
|  | [IPR036155](http://www.ebi.ac.uk/interpro/entry/IPR036155) | HOMOLOGOUS_SUPERFAMILY | Cryptochrome/photolyase, N-terminal domain superfamily |  | [SSF52425 (SUPERFAMILY)](http://www.ebi.ac.uk/interpro/signature/SSF52425) |
|  | no IPR | DOMAIN | DNA photolyase, N-terminal |  | [G3DSA:1.10.579.10(GENE3D)](http://www.ebi.ac.uk/interpro/signature/G3DSA:1.10.579.10) |
|  | no IPR | HOMOLOGOUS_SUPERFAMILY | Rossmann-like alpha/beta/alpha sandwich fold |  | [PTHR11455:SF30 (PANTHER)](http://www.ebi.ac.uk/interpro/signature/PTHR11455) |
| *RsCSNK2A* | [IPR000719](http://www.ebi.ac.uk/interpro/entry/IPR000719) | DOMAIN | Protein kinase domain | GO:0006468, GO:0005524, GO:0004672 | [SM00220 (SMART)](http://www.ebi.ac.uk/interpro/signature/SM00220) |
|  |  |  |  |  | [PF00069 (PFAM)](http://www.ebi.ac.uk/interpro/signature/PF00069) |
|  |  |  |  |  | [PS50011(PROSITE_PROFILES)](http://www.ebi.ac.uk/interpro/signature/PS50011) |
|  | [IPR008271](http://www.ebi.ac.uk/interpro/entry/IPR008271) | ACTIVE_SITE | Serine/threonine-protein kinase, active site | GO:0004672, GO:0006468 | [PS00108(PROSITE_PATTERNS)](http://www.ebi.ac.uk/interpro/signature/PS00108) |
|  | [IPR011009](http://www.ebi.ac.uk/interpro/entry/IPR011009) | HOMOLOGOUS_SUPERFAMILY | Protein kinase-like domain superfamily |  | [SSF56112(SUPERFAMILY)](http://www.ebi.ac.uk/interpro/signature/SSF56112) |
|  | [IPR017441](http://www.ebi.ac.uk/interpro/entry/IPR017441) | BINDING_SITE | Protein kinase, ATP binding site | GO:0005524 | [PS00107(PROSITE_PATTERNS)](http://www.ebi.ac.uk/interpro/signature/PS00107) |
|  | no IPR | DOMAIN | Protein kinase domain |  | [G3DSA:1.10.510.10(GENE3D)](http://www.ebi.ac.uk/interpro/signature/G3DSA:1.10.510.10) |
|  |  |  |  |  | [G3DSA:3.30.200.20(GENE3D)](http://www.ebi.ac.uk/interpro/signature/G3DSA:3.30.200.20) |
|  |  |  |  |  | [PTHR24054 (PANTHER)](http://www.ebi.ac.uk/interpro/signature/PTHR24054) |
|  |  |  |  |  | [PTHR24054:SF28 (PANTHER)](http://www.ebi.ac.uk/interpro/signature/PTHR24054) |
|  |  |  |  |  | cd14132 (CDD) |
| *RsDCLRE1A* | [IPR001279](http://www.ebi.ac.uk/interpro/entry/IPR001279) | DOMAIN | Metallo-beta-lactamase |  | [SM00849 (SMART)](http://www.ebi.ac.uk/interpro/signature/SM00849) |
|  | [IPR011084](http://www.ebi.ac.uk/interpro/entry/IPR011084) | DOMAIN | DNA repair metallo-beta-lactamase |  | [PF07522 (PFAM)](http://www.ebi.ac.uk/interpro/signature/PF07522) |
|  | [IPR036866](http://www.ebi.ac.uk/interpro/entry/IPR036866) | HOMOLOGOUS_SUPERFAMILY | Ribonuclease Z/Hydroxyacylglutathione hydrolase-like |  | [G3DSA:3.60.15.10 (GENE3D)](http://www.ebi.ac.uk/interpro/signature/G3DSA:3.60.15.10) |
|  |  |  |  |  | [SSF56281 (SUPERFAMILY)](http://www.ebi.ac.uk/interpro/signature/SSF56281) |
|  | no IPR | DOMAIN | DNA repair metallo-beta-lactamase |  | [G3DSA:3.40.50.12650 (GENE3D)](http://www.ebi.ac.uk/interpro/signature/G3DSA:3.40.50.1265) |
|  | no IPR | HOMOLOGOUS_SUPERFAMILY | Ribonuclease Z/Hydroxyacylglutathione hydrolase-like |  | [PTHR23240:SF6 (PANTHER)](http://www.ebi.ac.uk/interpro/signature/PTHR23240) |
|  |  |  |  |  | [PTHR23240 (PANTHER)](http://www.ebi.ac.uk/interpro/signature/PTHR23240) |
|  |  |  |  |  | cd16273 (CDD) |
| *RsDCLRE1B* | [IPR001279](http://www.ebi.ac.uk/interpro/entry/IPR001279) | DOMAIN | Metallo-beta-lactamase |  | [SM00849 (SMART)](http://www.ebi.ac.uk/interpro/signature/SM00849) |
|  | [IPR011084](http://www.ebi.ac.uk/interpro/entry/IPR011084) | DOMAIN | DNA repair metallo-beta-lactamase |  | [PF07522 (PFAM)](http://www.ebi.ac.uk/interpro/signature/PF07522) |
|  | [IPR036866](http://www.ebi.ac.uk/interpro/entry/IPR036866) | HOMOLOGOUS_SUPERFAMILY | Ribonuclease Z/Hydroxyacylglutathione hydrolase-like |  | [G3DSA:3.60.15.10 (GENE3D)](http://www.ebi.ac.uk/interpro/signature/G3DSA:3.60.15.10) |
|  |  |  |  |  | [SSF56281 (SUPERFAMILY)](http://www.ebi.ac.uk/interpro/signature/SSF56281) |
|  | no IPR | DOMAIN | Metallo-beta-lactamase |  | [G3DSA:3.40.50.12650 (GENE3D)](http://www.ebi.ac.uk/interpro/signature/G3DSA:3.40.50.1265) |
|  | no IPR | HOMOLOGOUS_SUPERFAMILY | Ribonuclease Z/Hydroxyacylglutathione hydrolase-like |  | mobidb-lite (MOBIDB_LITE) |
|  |  |  |  |  | mobidb-lite (MOBIDB_LITE) |
|  |  |  |  |  | mobidb-lite (MOBIDB_LITE) |
|  |  |  |  |  | mobidb-lite (MOBIDB_LITE) |
|  |  |  |  |  | mobidb-lite (MOBIDB_LITE) |
|  |  |  |  |  | [PTHR23240 (PANTHER)](http://www.ebi.ac.uk/interpro/signature/PTHR23240) |
|  |  |  |  |  | [PTHR23240:SF8 (PANTHER)](http://www.ebi.ac.uk/interpro/signature/PTHR23240) |
| *RsDCLRE1C* | [IPR001279](http://www.ebi.ac.uk/interpro/entry/IPR001279) | DOMAIN | Metallo-beta-lactamase |  | [PF12706 (PFAM)](http://www.ebi.ac.uk/interpro/signature/PF12706) |
|  | [IPR011084](http://www.ebi.ac.uk/interpro/entry/IPR011084) | DOMAIN | DNA repair metallo-beta-lactamase |  | [PF07522 (PFAM)](http://www.ebi.ac.uk/interpro/signature/PF07522) |
|  | [IPR036866](http://www.ebi.ac.uk/interpro/entry/IPR036866) | HOMOLOGOUS_SUPERFAMILY | Ribonuclease Z/Hydroxyacylglutathione hydrolase-like |  | [G3DSA:3.60.15.10 (GENE3D)](http://www.ebi.ac.uk/interpro/signature/G3DSA:3.60.15.10) |
|  |  |  |  |  | [SSF56281 (SUPERFAMILY)](http://www.ebi.ac.uk/interpro/signature/SSF56281) |
|  | no IPR | DOMAIN | Metallo-beta-lactamase |  | [PTHR23240:SF21 (PANTHER)](http://www.ebi.ac.uk/interpro/signature/PTHR23240) |
|  |  |  |  |  | [PTHR23240 (PANTHER)](http://www.ebi.ac.uk/interpro/signature/PTHR23240) |
|  | no IPR |  |  |  | [G3DSA:3.40.50.12650 (GENE3D)](http://www.ebi.ac.uk/interpro/signature/G3DSA:3.40.50.1265) |
| *RsDDB1* | [IPR004871](http://www.ebi.ac.uk/interpro/entry/IPR004871) | DOMAIN | Cleavage/polyadenylation specificity factor, A subunit, C-terminal | GO:0003676, GO:0005634 | [PF03178 (PFAM)](http://www.ebi.ac.uk/interpro/signature/PF03178) |
|  | [IPR015943](http://www.ebi.ac.uk/interpro/entry/IPR015943) | HOMOLOGOUS_SUPERFAMILY | WD40/YVTN repeat-like-containing domain superfamily | GO:0005515 | [G3DSA:2.130.10.10(GENE3D)](http://www.ebi.ac.uk/interpro/signature/G3DSA:2.130.10.10) |
|  |  |  |  |  | [G3DSA:2.130.10.10(GENE3D)](http://www.ebi.ac.uk/interpro/signature/G3DSA:2.130.10.10) |
|  |  |  |  |  | [G3DSA:2.130.10.10(GENE3D)](http://www.ebi.ac.uk/interpro/signature/G3DSA:2.130.10.10) |
|  | [IPR031297](http://www.ebi.ac.uk/interpro/entry/IPR031297) | FAMILY | DNA damage-binding protein 1 | GO:0006281 | [PTHR10644:SF3 (PANTHER)](http://www.ebi.ac.uk/interpro/signature/PTHR10644) |
|  | no IPR | FAMILY | DNA damage-binding protein 1 |  | [PTHR10644(PANTHER)](http://www.ebi.ac.uk/interpro/signature/PTHR10644) |
|  | no IPR | HOMOLOGOUS_SUPERFAMILY | WD40/YVTN repeat-like-containing domain superfamily |  | [PF10433 (PFAM)](http://www.ebi.ac.uk/interpro/signature/PF10433) |
|  |  |  |  |  | [G3DSA:3.30.980.30(GENE3D)](http://www.ebi.ac.uk/interpro/signature/G3DSA:3.30.980.30) |
|  |  |  |  |  | mobidb-lite (MOBIDB_LITE) |
|  | no IPR |  |  |  | Coil (COILS) |
| *RsDDX1* | [IPR001650](http://www.ebi.ac.uk/interpro/entry/IPR001650) | DOMAIN | Helicase, C-terminal |  | [SM00490 (SMART)](http://www.ebi.ac.uk/interpro/signature/SM00490) |
|  |  |  |  |  | [PF00271 (PFAM)](http://www.ebi.ac.uk/interpro/signature/PF00271) |
|  |  |  |  |  | [PS51194(PROSITE_PROFILES)](http://www.ebi.ac.uk/interpro/signature/PS51194) |
|  |  |  |  |  | cd00079 (CDD) |
|  | [IPR001870](http://www.ebi.ac.uk/interpro/entry/IPR001870) | DOMAIN | B30.2/SPRY domain |  | [PS50188(PROSITE_PROFILES)](http://www.ebi.ac.uk/interpro/signature/PS50188) |
|  | [IPR003877](http://www.ebi.ac.uk/interpro/entry/IPR003877) | DOMAIN | SPRY domain | GO:0005515 | [SM00449 (SMART)](http://www.ebi.ac.uk/interpro/signature/SM00449) |
|  |  |  |  |  | [PF00622 (PFAM)](http://www.ebi.ac.uk/interpro/signature/PF00622) |
|  | [IPR011545](http://www.ebi.ac.uk/interpro/entry/IPR011545) | DOMAIN | DEAD/DEAH box helicase domain | GO:0005524, GO:0003676 | [PF00270 (PFAM)](http://www.ebi.ac.uk/interpro/signature/PF00270) |
|  | [IPR013320](http://www.ebi.ac.uk/interpro/entry/IPR013320) | HOMOLOGOUS_SUPERFAMILY | Concanavalin A-like lectin/glucanase domain superfamily |  | [SSF49899(SUPERFAMILY)](http://www.ebi.ac.uk/interpro/signature/SSF49899) |
|  | [IPR014001](http://www.ebi.ac.uk/interpro/entry/IPR014001) | DOMAIN | Helicase superfamily 1/2, ATP-binding domain |  | [SM00487 (SMART)](http://www.ebi.ac.uk/interpro/signature/SM00487) |
|  |  |  |  |  | [PS51192(PROSITE_PROFILES)](http://www.ebi.ac.uk/interpro/signature/PS51192) |
|  |  |  |  |  | [PS51192(PROSITE_PROFILES)](http://www.ebi.ac.uk/interpro/signature/PS51192) |
|  | [IPR014014](http://www.ebi.ac.uk/interpro/entry/IPR014014) | DOMAIN | RNA helicase, DEAD-box type, Q motif |  | [PS51195(PROSITE_PROFILES)](http://www.ebi.ac.uk/interpro/signature/PS51195) |
|  | [IPR027417](http://www.ebi.ac.uk/interpro/entry/IPR027417) | HOMOLOGOUS_SUPERFAMILY | P-loop containing nucleoside triphosphate hydrolase |  | [SSF52540(SUPERFAMILY)](http://www.ebi.ac.uk/interpro/signature/SSF52540) |
|  |  |  |  |  | [SSF52540(SUPERFAMILY)](http://www.ebi.ac.uk/interpro/signature/SSF52540) |
|  | no IPR | DOMAIN | DEAD/DEAH box helicase domain |  | [G3DSA:3.40.50.300(GENE3D)](http://www.ebi.ac.uk/interpro/signature/G3DSA:3.40.50.300) |
|  |  |  |  |  | [G3DSA:2.60.120.920(GENE3D)](http://www.ebi.ac.uk/interpro/signature/G3DSA:2.60.120.920) |
|  |  |  |  |  | [G3DSA:3.40.50.300(GENE3D)](http://www.ebi.ac.uk/interpro/signature/G3DSA:3.40.50.300) |
|  | no IPR | DOMAIN | Helicase superfamily 1/2, ATP-binding domain |  | cd00046 (CDD) |
|  | no IPR | DOMAIN | Helicase, C-terminal |  | [G3DSA:3.40.50.300(GENE3D)](http://www.ebi.ac.uk/interpro/signature/G3DSA:3.40.50.300) |
|  |  |  |  |  | cd12873 (CDD) |
|  | no IPR | DOMAIN | SPRY domain |  | [PTHR24031:SF307 (PANTHER)](http://www.ebi.ac.uk/interpro/signature/PTHR24031) |
| *RsECT2* | [IPR000219](http://www.ebi.ac.uk/interpro/entry/IPR000219) | DOMAIN | Dbl homology (DH) domain | GO:0005089, GO:0035023 | [SM00325 (SMART)](http://www.ebi.ac.uk/interpro/signature/SM00325) |
|  |  |  |  |  | [PF00621 (PFAM)](http://www.ebi.ac.uk/interpro/signature/PF00621) |
|  |  |  |  |  | [PS50010(PROSITE_PROFILES)](http://www.ebi.ac.uk/interpro/signature/PS50010) |
|  |  |  |  |  | cd00160 (CDD) |
|  | [IPR001331](http://www.ebi.ac.uk/interpro/entry/IPR001331) | CONSERVED_SITE | Guanine-nucleotide dissociation stimulator, CDC24, conserved site | GO:0035556, GO:0005085 | [PS00741(PROSITE_PATTERNS)](http://www.ebi.ac.uk/interpro/signature/PS00741) |
|  | [IPR001357](http://www.ebi.ac.uk/interpro/entry/IPR001357) | DOMAIN | BRCT domain |  | [SM00292 (SMART)](http://www.ebi.ac.uk/interpro/signature/SM00292) |
|  |  |  |  |  | [PF12738 (PFAM)](http://www.ebi.ac.uk/interpro/signature/PF12738) |
|  |  |  |  |  | [PF00533 (PFAM)](http://www.ebi.ac.uk/interpro/signature/PF00533) |
|  |  |  |  |  | [PS50172(PROSITE_PROFILES)](http://www.ebi.ac.uk/interpro/signature/PS50172) |
|  |  |  |  |  | [PS50172(PROSITE_PROFILES)](http://www.ebi.ac.uk/interpro/signature/PS50172) |
|  |  |  |  |  | cd00027 (CDD) |
|  |  |  |  |  | cd00027 (CDD) |
|  | [IPR011993](http://www.ebi.ac.uk/interpro/entry/IPR011993) | HOMOLOGOUS_SUPERFAMILY | PH-like domain superfamily |  | [G3DSA:2.30.29.30(GENE3D)](http://www.ebi.ac.uk/interpro/signature/G3DSA:2.30.29.30) |
|  | [IPR026817](http://www.ebi.ac.uk/interpro/entry/IPR026817) | FAMILY | Guanine nucleotide exchange factor Ect2 | GO:0000902, GO:0045666, GO:0005634, GO:0005085, GO:0005737, GO:0005096, GO:0000910 | [PTHR16777(PANTHER)](http://www.ebi.ac.uk/interpro/signature/PTHR16777) |
|  | [IPR035899](http://www.ebi.ac.uk/interpro/entry/IPR035899) | HOMOLOGOUS_SUPERFAMILY | Dbl homology (DH) domain superfamily |  | [G3DSA:1.20.900.10(GENE3D)](http://www.ebi.ac.uk/interpro/signature/G3DSA:1.20.900.10) |
|  |  |  |  |  | [SSF48065(SUPERFAMILY)](http://www.ebi.ac.uk/interpro/signature/SSF48065) |
|  | [IPR036420](http://www.ebi.ac.uk/interpro/entry/IPR036420) | HOMOLOGOUS_SUPERFAMILY | BRCT domain superfamily |  | [G3DSA:3.40.50.10190 (GENE3D)](http://www.ebi.ac.uk/interpro/signature/G3DSA:3.40.50.1019) |
|  |  |  |  |  | [G3DSA:3.40.50.10190 (GENE3D)](http://www.ebi.ac.uk/interpro/signature/G3DSA:3.40.50.1019) |
|  |  |  |  |  | [G3DSA:3.40.50.10190 (GENE3D)](http://www.ebi.ac.uk/interpro/signature/G3DSA:3.40.50.1019) |
|  |  |  |  |  | [SSF52113(SUPERFAMILY)](http://www.ebi.ac.uk/interpro/signature/SSF52113) |
|  |  |  |  |  | [SSF52113(SUPERFAMILY)](http://www.ebi.ac.uk/interpro/signature/SSF52113) |
|  | no IPR | DOMAIN | BRCT domain |  | cd01229 (CDD) |
| *RsEME1* | [IPR006166](http://www.ebi.ac.uk/interpro/entry/IPR006166) | DOMAIN | ERCC4 domain | GO:0004518, GO:0003677 | [SM00891 (SMART)](http://www.ebi.ac.uk/interpro/signature/SM00891) |
|  |  |  |  |  | [PF02732 (PFAM)](http://www.ebi.ac.uk/interpro/signature/PF02732) |
|  | [IPR033310](http://www.ebi.ac.uk/interpro/entry/IPR033310) | FAMILY | Mms4/EME1/EME2 | GO:0006281, GO:0005634, GO:0048476 | [PTHR21077 (PANTHER)](http://www.ebi.ac.uk/interpro/signature/PTHR21077) |
|  | no IPR | DOMAIN | ERCC4 domain |  | [G3DSA:1.10.150.670 (GENE3D)](http://www.ebi.ac.uk/interpro/signature/G3DSA:1.10.150.670) |
|  |  |  |  |  | mobidb-lite (MOBIDB_LITE) |
|  | no IPR |  |  |  | Coil (COILS) |
| *RsEN5* | [IPR007581](http://www.ebi.ac.uk/interpro/entry/IPR007581) | FAMILY | Endonuclease V | GO:0004519, GO:0006281 | [PF04493 (PFAM)](http://www.ebi.ac.uk/interpro/signature/PF04493) |
|  |  |  |  |  | [PTHR28511 (PANTHER)](http://www.ebi.ac.uk/interpro/signature/PTHR28511) |
|  |  |  |  |  | MF_00801 (HAMAP) |
|  |  |  |  |  | cd06559 (CDD) |
|  | no IPR | FAMILY | Endonuclease V |  | [PTHR28511:SF1 (PANTHER)](http://www.ebi.ac.uk/interpro/signature/PTHR28511) |
|  | no IPR |  |  |  | [G3DSA:3.30.2170.10 (GENE3D)](http://www.ebi.ac.uk/interpro/signature/G3DSA:3.30.2170.10) |
| *RsERCC1* | [IPR004579](http://www.ebi.ac.uk/interpro/entry/IPR004579) | FAMILY | ERCC1/RAD10/SWI10 family | GO:0003684, GO:0006281, GO:0004519, GO:0005634 | [PF03834 (PFAM)](http://www.ebi.ac.uk/interpro/signature/PF03834) |
|  |  |  |  |  | [TIGR00597 (TIGRFAM)](http://www.ebi.ac.uk/interpro/signature/TIGR00597) |
|  |  |  |  |  | [PTHR12749 (PANTHER)](http://www.ebi.ac.uk/interpro/signature/PTHR12749) |
|  |  |  |  |  | PD013585 (PRODOM) |
|  | [IPR010994](http://www.ebi.ac.uk/interpro/entry/IPR010994) | HOMOLOGOUS_SUPERFAMILY | RuvA domain 2-like |  | [SSF47781(SUPERFAMILY)](http://www.ebi.ac.uk/interpro/signature/SSF47781) |
|  | [IPR011335](http://www.ebi.ac.uk/interpro/entry/IPR011335) | HOMOLOGOUS_SUPERFAMILY | Restriction endonuclease type II-like |  | [SSF52980(SUPERFAMILY)](http://www.ebi.ac.uk/interpro/signature/SSF52980) |
|  | no IPR | FAMILY | ERCC1/RAD10/SWI10 family |  | [PF14520 (PFAM)](http://www.ebi.ac.uk/interpro/signature/PF14520) |
|  |  |  |  |  | [G3DSA:1.10.150.20(GENE3D)](http://www.ebi.ac.uk/interpro/signature/G3DSA:1.10.150.20) |
|  |  |  |  |  | [G3DSA:3.40.50.10130 (GENE3D)](http://www.ebi.ac.uk/interpro/signature/G3DSA:3.40.50.1013) |
|  |  |  |  |  | mobidb-lite (MOBIDB_LITE) |
| *RsERCC2* | [IPR001945](http://www.ebi.ac.uk/interpro/entry/IPR001945) | FAMILY | RAD3/XPD family | GO:0005524, GO:0006289, GO:0004003, GO:0005634, GO:0003677, GO:0016818 | [PR00852 (PRINTS)](http://www.ebi.ac.uk/interpro/signature/PR00852) |
|  |  |  |  |  | [PTHR11472:SF1 (PANTHER)](http://www.ebi.ac.uk/interpro/signature/PTHR11472) |
|  | [IPR002464](http://www.ebi.ac.uk/interpro/entry/IPR002464) | CONSERVED_SITE | DNA/RNA helicase, ATP-dependent, DEAH-box type, conserved site |  | [PS00690(PROSITE_PATTERNS)](http://www.ebi.ac.uk/interpro/signature/PS00690) |
|  | [IPR006554](http://www.ebi.ac.uk/interpro/entry/IPR006554) | DOMAIN | Helicase-like, DEXD box c2 type | GO:0016818, GO:0004003 | [SM00488 (SMART)](http://www.ebi.ac.uk/interpro/signature/SM00488) |
|  | [IPR006555](http://www.ebi.ac.uk/interpro/entry/IPR006555) | DOMAIN | ATP-dependent helicase, C-terminal | GO:0003676, GO:0016818, GO:0008026, GO:0006139, GO:0005524 | [SM00491 (SMART)](http://www.ebi.ac.uk/interpro/signature/SM00491) |
|  |  |  |  |  | [PF13307 (PFAM)](http://www.ebi.ac.uk/interpro/signature/PF13307) |
|  | [IPR010614](http://www.ebi.ac.uk/interpro/entry/IPR010614) | DOMAIN | DEAD2 | GO:0003677, GO:0004003, GO:0005524 | [PF06733 (PFAM)](http://www.ebi.ac.uk/interpro/signature/PF06733) |
|  | [IPR010643](http://www.ebi.ac.uk/interpro/entry/IPR010643) | DOMAIN | Helical and beta-bridge domain |  | [PF06777 (PFAM)](http://www.ebi.ac.uk/interpro/signature/PF06777) |
|  | [IPR013020](http://www.ebi.ac.uk/interpro/entry/IPR013020) | FAMILY | ATP-dependent helicase Rad3/Chl1-like | GO:0004003 | [TIGR00604 (TIGRFAM)](http://www.ebi.ac.uk/interpro/signature/TIGR00604) |
|  | [IPR014013](http://www.ebi.ac.uk/interpro/entry/IPR014013) | DOMAIN | Helicase superfamily 1/2, ATP-binding domain, DinG/Rad3-type | GO:0005524 | [PS51193(PROSITE_PROFILES)](http://www.ebi.ac.uk/interpro/signature/PS51193) |
|  | [IPR027417](http://www.ebi.ac.uk/interpro/entry/IPR027417) | HOMOLOGOUS_SUPERFAMILY | P-loop containing nucleoside triphosphate hydrolase |  | [SSF52540(SUPERFAMILY)](http://www.ebi.ac.uk/interpro/signature/SSF52540) |
|  |  |  |  |  | [SSF52540(SUPERFAMILY)](http://www.ebi.ac.uk/interpro/signature/SSF52540) |
|  | no IPR | DOMAIN | ATP-dependent helicase, C-terminal |  | [G3DSA:3.40.50.300(GENE3D)](http://www.ebi.ac.uk/interpro/signature/G3DSA:3.40.50.300) |
|  | no IPR | DOMAIN | DEAD2 |  | [G3DSA:3.40.50.300(GENE3D)](http://www.ebi.ac.uk/interpro/signature/G3DSA:3.40.50.300) |
|  | no IPR | FAMILY | RAD3/XPD family |  | [PTHR11472(PANTHER)](http://www.ebi.ac.uk/interpro/signature/PTHR11472) |
|  | no IPR |  |  |  | Coil (COILS) |
|  |  |  |  |  | Coil (COILS) |
| *RsERCC3* | [IPR001161](http://www.ebi.ac.uk/interpro/entry/IPR001161) | FAMILY | Helicase XPB/Ssl2 | GO:0004003, GO:0006367, GO:0006289 | [TIGR00603 (TIGRFAM)](http://www.ebi.ac.uk/interpro/signature/TIGR00603) |
|  | [IPR001650](http://www.ebi.ac.uk/interpro/entry/IPR001650) | DOMAIN | Helicase, C-terminal |  | [SM00490 (SMART)](http://www.ebi.ac.uk/interpro/signature/SM00490) |
|  |  |  |  |  | [PS51194(PROSITE_PROFILES)](http://www.ebi.ac.uk/interpro/signature/PS51194) |
|  |  |  |  |  | cd00079 (CDD) |
|  | [IPR006935](http://www.ebi.ac.uk/interpro/entry/IPR006935) | DOMAIN | Helicase/UvrB, N-terminal | GO:0016787, GO:0003677, GO:0005524 | [PF04851 (PFAM)](http://www.ebi.ac.uk/interpro/signature/PF04851) |
|  | [IPR014001](http://www.ebi.ac.uk/interpro/entry/IPR014001) | DOMAIN | Helicase superfamily 1/2, ATP-binding domain |  | [SM00487 (SMART)](http://www.ebi.ac.uk/interpro/signature/SM00487) |
|  |  |  |  |  | [PS51192(PROSITE_PROFILES)](http://www.ebi.ac.uk/interpro/signature/PS51192) |
|  | [IPR027417](http://www.ebi.ac.uk/interpro/entry/IPR027417) | HOMOLOGOUS_SUPERFAMILY | P-loop containing nucleoside triphosphate hydrolase |  | [SSF52540(SUPERFAMILY)](http://www.ebi.ac.uk/interpro/signature/SSF52540) |
|  |  |  |  |  | [SSF52540(SUPERFAMILY)](http://www.ebi.ac.uk/interpro/signature/SSF52540) |
|  | [IPR032438](http://www.ebi.ac.uk/interpro/entry/IPR032438) | DOMAIN | ERCC3/RAD25/XPB helicase, C-terminal domain |  | [PF16203 (PFAM)](http://www.ebi.ac.uk/interpro/signature/PF16203) |
|  | [IPR032830](http://www.ebi.ac.uk/interpro/entry/IPR032830) | DOMAIN | Helicase XPB/Ssl2, N-terminal domain |  | [PF13625 (PFAM)](http://www.ebi.ac.uk/interpro/signature/PF13625) |
|  | no IPR | DOMAIN | ERCC3/RAD25/XPB helicase, C-terminal domain |  | mobidb-lite (MOBIDB_LITE) |
|  |  |  |  |  | mobidb-lite (MOBIDB_LITE) |
|  |  |  |  |  | mobidb-lite (MOBIDB_LITE) |
|  |  |  |  |  | [PTHR11274:SF0 (PANTHER)](http://www.ebi.ac.uk/interpro/signature/PTHR11274) |
|  |  |  |  |  | [PTHR11274 (PANTHER)](http://www.ebi.ac.uk/interpro/signature/PTHR11274) |
|  | no IPR | DOMAIN | Helicase XPB/Ssl2, N-terminal domain |  | [G3DSA:3.40.50.300(GENE3D)](http://www.ebi.ac.uk/interpro/signature/G3DSA:3.40.50.300) |
|  | no IPR | DOMAIN | Helicase, C-terminal |  | cd00046 (CDD) |
|  | no IPR | DOMAIN | Helicase/UvrB, N-terminal |  | [G3DSA:3.40.50.300(GENE3D)](http://www.ebi.ac.uk/interpro/signature/G3DSA:3.40.50.300) |
|  | no IPR |  |  |  | [PR00851 (PRINTS)](http://www.ebi.ac.uk/interpro/signature/PR00851) |
| *RsERCC5* | [IPR001044](http://www.ebi.ac.uk/interpro/entry/IPR001044) | FAMILY | XPG/Rad2 endonuclease, eukaryotes | GO:0003697, GO:0005634, GO:0004519, GO:0006289 | [PR00066 (PRINTS)](http://www.ebi.ac.uk/interpro/signature/PR00066) |
|  | [IPR006084](http://www.ebi.ac.uk/interpro/entry/IPR006084) | FAMILY | XPG/Rad2 endonuclease | GO:0004518, GO:0006281 | [PR00853 (PRINTS)](http://www.ebi.ac.uk/interpro/signature/PR00853) |
|  | [IPR006085](http://www.ebi.ac.uk/interpro/entry/IPR006085) | DOMAIN | XPG N-terminal | GO:0006281, GO:0004518 | [SM00485 (SMART)](http://www.ebi.ac.uk/interpro/signature/SM00485) |
|  |  |  |  |  | [PF00752 (PFAM)](http://www.ebi.ac.uk/interpro/signature/PF00752) |
|  | [IPR006086](http://www.ebi.ac.uk/interpro/entry/IPR006086) | DOMAIN | XPG-I domain | GO:0004518 | [SM00484 (SMART)](http://www.ebi.ac.uk/interpro/signature/SM00484) |
|  |  |  |  |  | [PF00867 (PFAM)](http://www.ebi.ac.uk/interpro/signature/PF00867) |
|  | [IPR008918](http://www.ebi.ac.uk/interpro/entry/IPR008918) | CONSERVED_SITE | Helix-hairpin-helix motif, class 2 | GO:0003824, GO:0003677 | [SM00279 (SMART)](http://www.ebi.ac.uk/interpro/signature/SM00279) |
|  | [IPR019974](http://www.ebi.ac.uk/interpro/entry/IPR019974) | CONSERVED_SITE | XPG conserved site | GO:0016788 | [PS00841(PROSITE_PATTERNS)](http://www.ebi.ac.uk/interpro/signature/PS00841) |
|  |  |  |  |  | [PS00842(PROSITE_PATTERNS)](http://www.ebi.ac.uk/interpro/signature/PS00842) |
|  | [IPR029060](http://www.ebi.ac.uk/interpro/entry/IPR029060) | HOMOLOGOUS_SUPERFAMILY | PIN-like domain superfamily |  | [SSF88723(SUPERFAMILY)](http://www.ebi.ac.uk/interpro/signature/SSF88723) |
|  | [IPR036279](http://www.ebi.ac.uk/interpro/entry/IPR036279) | HOMOLOGOUS_SUPERFAMILY | 5'-3' exonuclease, C-terminal domain superfamily |  | [SSF47807(SUPERFAMILY)](http://www.ebi.ac.uk/interpro/signature/SSF47807) |
|  | no IPR | DOMAIN | XPG N-terminal |  | [G3DSA:3.40.50.1010(GENE3D)](http://www.ebi.ac.uk/interpro/signature/G3DSA:3.40.50.1010) |
|  |  |  |  |  | [G3DSA:3.40.50.1010(GENE3D)](http://www.ebi.ac.uk/interpro/signature/G3DSA:3.40.50.1010) |
|  | no IPR | CONSERVED_SITE | XPG conserved site |  | cd09868 (CDD) |
|  |  |  |  |  | cd09868 (CDD) |
|  |  |  |  |  | cd09904 (CDD) |
|  | no IPR | DOMAIN | XPG-I domain |  | mobidb-lite (MOBIDB_LITE) |
|  |  |  |  |  | mobidb-lite (MOBIDB_LITE) |
|  |  |  |  |  | mobidb-lite (MOBIDB_LITE) |
|  |  |  |  |  | mobidb-lite (MOBIDB_LITE) |
|  |  |  |  |  | mobidb-lite (MOBIDB_LITE) |
|  |  |  |  |  | [PTHR16171(PANTHER)](http://www.ebi.ac.uk/interpro/signature/PTHR16171) |
|  |  |  |  |  | [PTHR16171:SF7 (PANTHER)](http://www.ebi.ac.uk/interpro/signature/PTHR16171) |
|  | no IPR |  |  |  | Coil (COILS) |
| *RsERCC6* | [IPR000330](http://www.ebi.ac.uk/interpro/entry/IPR000330) | DOMAIN | SNF2-related, N-terminal domain | GO:0005524 | [PF00176 (PFAM)](http://www.ebi.ac.uk/interpro/signature/PF00176) |
|  | [IPR001650](http://www.ebi.ac.uk/interpro/entry/IPR001650) | DOMAIN | Helicase, C-terminal |  | [SM00490 (SMART)](http://www.ebi.ac.uk/interpro/signature/SM00490) |
|  |  |  |  |  | [PF00271 (PFAM)](http://www.ebi.ac.uk/interpro/signature/PF00271) |
|  |  |  |  |  | [PS51194(PROSITE_PROFILES)](http://www.ebi.ac.uk/interpro/signature/PS51194) |
|  |  |  |  |  | cd00079 (CDD) |
|  | [IPR014001](http://www.ebi.ac.uk/interpro/entry/IPR014001) | DOMAIN | Helicase superfamily 1/2, ATP-binding domain |  | [SM00487 (SMART)](http://www.ebi.ac.uk/interpro/signature/SM00487) |
|  |  |  |  |  | [PS51192(PROSITE_PROFILES)](http://www.ebi.ac.uk/interpro/signature/PS51192) |
|  | [IPR027417](http://www.ebi.ac.uk/interpro/entry/IPR027417) | HOMOLOGOUS_SUPERFAMILY | P-loop containing nucleoside triphosphate hydrolase |  | [SSF52540 (SUPERFAMILY)](http://www.ebi.ac.uk/interpro/signature/SSF52540) |
|  |  |  |  |  | [SSF52540 (SUPERFAMILY)](http://www.ebi.ac.uk/interpro/signature/SSF52540) |
|  | [IPR038718](http://www.ebi.ac.uk/interpro/entry/IPR038718) | HOMOLOGOUS_SUPERFAMILY | SNF2-like, N-terminal domain superfamily |  | [G3DSA:3.40.50.10810 (GENE3D)](http://www.ebi.ac.uk/interpro/signature/G3DSA:3.40.50.1081) |
|  | no IPR | DOMAIN | Helicase, C-terminal |  | cd00046 (CDD) |
|  | no IPR | HOMOLOGOUS_SUPERFAMILY | SNF2-like, N-terminal domain superfamily |  | [G3DSA:3.40.50.300 (GENE3D)](http://www.ebi.ac.uk/interpro/signature/G3DSA:3.40.50.300) |
|  |  |  |  |  | mobidb-lite (MOBIDB_LITE) |
|  |  |  |  |  | mobidb-lite (MOBIDB_LITE) |
|  |  |  |  |  | mobidb-lite (MOBIDB_LITE) |
|  |  |  |  |  | mobidb-lite (MOBIDB_LITE) |
|  |  |  |  |  | mobidb-lite (MOBIDB_LITE) |
|  |  |  |  |  | mobidb-lite (MOBIDB_LITE) |
|  |  |  |  |  | mobidb-lite (MOBIDB_LITE) |
|  |  |  |  |  | mobidb-lite (MOBIDB_LITE) |
|  |  |  |  |  | [PTHR10799 (PANTHER)](http://www.ebi.ac.uk/interpro/signature/PTHR10799) |
|  |  |  |  |  | [PTHR10799:SF837 (PANTHER)](http://www.ebi.ac.uk/interpro/signature/PTHR10799) |
|  | no IPR |  |  |  | Coil (COILS) |
|  |  |  |  |  | Coil (COILS) |
| *RsERCC8* | [IPR001680](http://www.ebi.ac.uk/interpro/entry/IPR001680) | REPEAT | WD40 repeat | GO:0005515 | [SM00320 (SMART)](http://www.ebi.ac.uk/interpro/signature/SM00320) |
|  |  |  |  |  | [PF00400 (PFAM)](http://www.ebi.ac.uk/interpro/signature/PF00400) |
|  |  |  |  |  | [PS50082(PROSITE_PROFILES)](http://www.ebi.ac.uk/interpro/signature/PS50082) |
|  |  |  |  |  | [PS50082(PROSITE_PROFILES)](http://www.ebi.ac.uk/interpro/signature/PS50082) |
|  |  |  |  |  | [PS50082(PROSITE_PROFILES)](http://www.ebi.ac.uk/interpro/signature/PS50082) |
|  |  |  |  |  | [PS50082(PROSITE_PROFILES)](http://www.ebi.ac.uk/interpro/signature/PS50082) |
|  | [IPR015943](http://www.ebi.ac.uk/interpro/entry/IPR015943) | HOMOLOGOUS_SUPERFAMILY | WD40/YVTN repeat-like-containing domain superfamily | GO:0005515 | [G3DSA:2.130.10.10(GENE3D)](http://www.ebi.ac.uk/interpro/signature/G3DSA:2.130.10.10) |
|  | [IPR017986](http://www.ebi.ac.uk/interpro/entry/IPR017986) | DOMAIN | WD40-repeat-containing domain | GO:0005515 | [PS50294(PROSITE_PROFILES)](http://www.ebi.ac.uk/interpro/signature/PS50294) |
|  | [IPR019775](http://www.ebi.ac.uk/interpro/entry/IPR019775) | CONSERVED_SITE | WD40 repeat, conserved site |  | [PS00678(PROSITE_PATTERNS)](http://www.ebi.ac.uk/interpro/signature/PS00678) |
|  | [IPR020472](http://www.ebi.ac.uk/interpro/entry/IPR020472) | REPEAT | G-protein beta WD-40 repeat |  | [PR00320 (PRINTS)](http://www.ebi.ac.uk/interpro/signature/PR00320) |
|  | [IPR036322](http://www.ebi.ac.uk/interpro/entry/IPR036322) | HOMOLOGOUS_SUPERFAMILY | WD40-repeat-containing domain superfamily | GO:0005515 | [SSF50978 (SUPERFAMILY)](http://www.ebi.ac.uk/interpro/signature/SSF50978) |
|  | no IPR | REPEAT | WD40 repeat |  | mobidb-lite (MOBIDB_LITE) |
| *RsFANCM* | [IPR001650](http://www.ebi.ac.uk/interpro/entry/IPR001650) | DOMAIN | Helicase, C-terminal |  | [SM00490 (SMART)](http://www.ebi.ac.uk/interpro/signature/SM00490) |
|  |  |  |  |  | [PF00271 (PFAM)](http://www.ebi.ac.uk/interpro/signature/PF00271) |
|  |  |  |  |  | [PS51194(PROSITE_PROFILES)](http://www.ebi.ac.uk/interpro/signature/PS51194) |
|  |  |  |  |  | cd00079 (CDD) |
|  | [IPR003903](http://www.ebi.ac.uk/interpro/entry/IPR003903) | CONSERVED_SITE | Ubiquitin interacting motif |  | [PS50330(PROSITE_PROFILES)](http://www.ebi.ac.uk/interpro/signature/PS50330) |
|  | [IPR006935](http://www.ebi.ac.uk/interpro/entry/IPR006935) | DOMAIN | Helicase/UvrB, N-terminal | GO:0016787, GO:0003677, GO:0005524 | [PF04851 (PFAM)](http://www.ebi.ac.uk/interpro/signature/PF04851) |
|  | [IPR014001](http://www.ebi.ac.uk/interpro/entry/IPR014001) | DOMAIN | Helicase superfamily 1/2, ATP-binding domain |  | [SM00487 (SMART)](http://www.ebi.ac.uk/interpro/signature/SM00487) |
|  |  |  |  |  | [PS51192(PROSITE_PROFILES)](http://www.ebi.ac.uk/interpro/signature/PS51192) |
|  | [IPR027417](http://www.ebi.ac.uk/interpro/entry/IPR027417) | HOMOLOGOUS_SUPERFAMILY | P-loop containing nucleoside triphosphate hydrolase |  | [SSF52540(SUPERFAMILY)](http://www.ebi.ac.uk/interpro/signature/SSF52540) |
|  | no IPR | DOMAIN | Helicase, C-terminal |  | [G3DSA:3.40.50.300(GENE3D)](http://www.ebi.ac.uk/interpro/signature/G3DSA:3.40.50.300) |
|  |  |  |  |  | mobidb-lite (MOBIDB_LITE) |
|  |  |  |  |  | mobidb-lite (MOBIDB_LITE) |
|  |  |  |  |  | mobidb-lite (MOBIDB_LITE) |
|  |  |  |  |  | mobidb-lite (MOBIDB_LITE) |
|  |  |  |  |  | mobidb-lite (MOBIDB_LITE) |
|  |  |  |  |  | mobidb-lite (MOBIDB_LITE) |
|  |  |  |  |  | mobidb-lite (MOBIDB_LITE) |
|  |  |  |  |  | mobidb-lite (MOBIDB_LITE) |
|  |  |  |  |  | mobidb-lite (MOBIDB_LITE) |
|  |  |  |  |  | [PTHR14025:SF20 (PANTHER)](http://www.ebi.ac.uk/interpro/signature/PTHR14025) |
|  |  |  |  |  | [PTHR14025 (PANTHER)](http://www.ebi.ac.uk/interpro/signature/PTHR14025) |
|  | no IPR | DOMAIN | Helicase/UvrB, N-terminal |  | [G3DSA:3.40.50.300(GENE3D)](http://www.ebi.ac.uk/interpro/signature/G3DSA:3.40.50.300) |
|  |  |  |  |  | [G3DSA:1.20.1320.20(GENE3D)](http://www.ebi.ac.uk/interpro/signature/G3DSA:1.20.1320.20) |
|  | no IPR | CONSERVED_SITE | Ubiquitin interacting motif |  | cd12091 (CDD) |
|  |  |  |  |  | cd00046 (CDD) |
|  | no IPR |  |  |  | Coil (COILS) |
| *RsGEN1* | [IPR006084](http://www.ebi.ac.uk/interpro/entry/IPR006084) | FAMILY | XPG/Rad2 endonuclease | GO:0004518, GO:0006281 | [PR00853 (PRINTS)](http://www.ebi.ac.uk/interpro/signature/PR00853) |
|  |  |  |  |  | [PTHR11081 (PANTHER)](http://www.ebi.ac.uk/interpro/signature/PTHR11081) |
|  | [IPR006085](http://www.ebi.ac.uk/interpro/entry/IPR006085) | DOMAIN | XPG N-terminal | GO:0006281, GO:0004518 | [SM00485 (SMART)](http://www.ebi.ac.uk/interpro/signature/SM00485) |
|  |  |  |  |  | [PF00752 (PFAM)](http://www.ebi.ac.uk/interpro/signature/PF00752) |
|  | [IPR006086](http://www.ebi.ac.uk/interpro/entry/IPR006086) | DOMAIN | XPG-I domain | GO:0004518 | [SM00484 (SMART)](http://www.ebi.ac.uk/interpro/signature/SM00484) |
|  |  |  |  |  | [PF00867 (PFAM)](http://www.ebi.ac.uk/interpro/signature/PF00867) |
|  | [IPR008918](http://www.ebi.ac.uk/interpro/entry/IPR008918) | CONSERVED_SITE | Helix-hairpin-helix motif, class 2 | GO:0003677, GO:0003824 | [SM00279 (SMART)](http://www.ebi.ac.uk/interpro/signature/SM00279) |
|  | [IPR029060](http://www.ebi.ac.uk/interpro/entry/IPR029060) | HOMOLOGOUS_SUPERFAMILY | PIN-like domain superfamily |  | [SSF88723 (SUPERFAMILY)](http://www.ebi.ac.uk/interpro/signature/SSF88723) |
|  | [IPR036279](http://www.ebi.ac.uk/interpro/entry/IPR036279) | HOMOLOGOUS_SUPERFAMILY | 5'-3' exonuclease, C-terminal domain superfamily |  | [SSF47807 (SUPERFAMILY)](http://www.ebi.ac.uk/interpro/signature/SSF47807) |
|  | no IPR | DOMAIN | XPG-I domain |  | mobidb-lite (MOBIDB_LITE) |
|  |  |  |  |  | mobidb-lite (MOBIDB_LITE) |
|  |  |  |  |  | [PTHR11081:SF23 (PANTHER)](http://www.ebi.ac.uk/interpro/signature/PTHR11081) |
|  | no IPR | FAMILY | XPG/Rad2 endonuclease |  | CYTOPLASMIC_DOMAIN (PHOBIUS) |
|  |  |  |  |  | NON_CYTOPLASMIC_DOMAIN (PHOBIUS) |
|  |  |  |  |  | TRANSMEMBRANE (PHOBIUS) |
|  | no IPR |  |  |  | Coil (COILS) |
| *RsGTF2H2* | [IPR002035](http://www.ebi.ac.uk/interpro/entry/IPR002035) | DOMAIN | von Willebrand factor, type A |  | [SM00327 (SMART)](http://www.ebi.ac.uk/interpro/signature/SM00327) |
|  |  |  |  |  | [PS50234(PROSITE_PROFILES)](http://www.ebi.ac.uk/interpro/signature/PS50234) |
|  | [IPR004595](http://www.ebi.ac.uk/interpro/entry/IPR004595) | DOMAIN | TFIIH C1-like domain | GO:0006281, GO:0008270 | [SM01047 (SMART)](http://www.ebi.ac.uk/interpro/signature/SM01047) |
|  |  |  |  |  | [PF07975 (PFAM)](http://www.ebi.ac.uk/interpro/signature/PF07975) |
|  | [IPR007198](http://www.ebi.ac.uk/interpro/entry/IPR007198) | DOMAIN | Ssl1-like |  | [PF04056 (PFAM)](http://www.ebi.ac.uk/interpro/signature/PF04056) |
|  |  |  |  |  | cd01453 (CDD) |
|  | [IPR012170](http://www.ebi.ac.uk/interpro/entry/IPR012170) | FAMILY | TFIIH subunit Ssl1/p44 | GO:0006351, GO:0000439, GO:0006289 | PIRSF015919 (PIRSF) |
|  |  |  |  |  | [TIGR00622 (TIGRFAM)](http://www.ebi.ac.uk/interpro/signature/TIGR00622) |
|  |  |  |  |  | [PTHR12695 (PANTHER)](http://www.ebi.ac.uk/interpro/signature/PTHR12695) |
|  | [IPR013083](http://www.ebi.ac.uk/interpro/entry/IPR013083) | HOMOLOGOUS_SUPERFAMILY | Zinc finger, RING/FYVE/PHD-type |  | [G3DSA:3.30.40.10(GENE3D)](http://www.ebi.ac.uk/interpro/signature/G3DSA:3.30.40.10) |
|  | [IPR013087](http://www.ebi.ac.uk/interpro/entry/IPR013087) | DOMAIN | Zinc finger C2H2-type | GO:0003676 | [PS00028(PROSITE_PATTERNS)](http://www.ebi.ac.uk/interpro/signature/PS00028) |
|  |  |  |  |  | [PS50157(PROSITE_PROFILES)](http://www.ebi.ac.uk/interpro/signature/PS50157) |
|  | [IPR036465](http://www.ebi.ac.uk/interpro/entry/IPR036465) | HOMOLOGOUS_SUPERFAMILY | von Willebrand factor A-like domain superfamily |  | [G3DSA:3.40.50.410(GENE3D)](http://www.ebi.ac.uk/interpro/signature/G3DSA:3.40.50.410) |
|  |  |  |  |  | [SSF53300(SUPERFAMILY)](http://www.ebi.ac.uk/interpro/signature/SSF53300) |
|  | no IPR | DOMAIN | Ssl1-like |  | [SSF57889(SUPERFAMILY)](http://www.ebi.ac.uk/interpro/signature/SSF57889) |
| *RsH2AFY* | [IPR002119](http://www.ebi.ac.uk/interpro/entry/IPR002119) | FAMILY | Histone H2A | GO:0005634, GO:0000786, GO:0003677 | [PR00620 (PRINTS)](http://www.ebi.ac.uk/interpro/signature/PR00620) |
|  |  |  |  |  | [SM00414 (SMART)](http://www.ebi.ac.uk/interpro/signature/SM00414) |
|  |  |  |  |  | cd00074 (CDD) |
|  | [IPR002589](http://www.ebi.ac.uk/interpro/entry/IPR002589) | DOMAIN | Macro domain |  | [SM00506 (SMART)](http://www.ebi.ac.uk/interpro/signature/SM00506) |
|  |  |  |  |  | [PF01661 (PFAM)](http://www.ebi.ac.uk/interpro/signature/PF01661) |
|  |  |  |  |  | [PS51154(PROSITE_PROFILES)](http://www.ebi.ac.uk/interpro/signature/PS51154) |
|  | [IPR007125](http://www.ebi.ac.uk/interpro/entry/IPR007125) | DOMAIN | Histone H2A/H2B/H3 | GO:0003677 | [PF00125 (PFAM)](http://www.ebi.ac.uk/interpro/signature/PF00125) |
|  | [IPR009072](http://www.ebi.ac.uk/interpro/entry/IPR009072) | HOMOLOGOUS_SUPERFAMILY | Histone-fold | GO:0046982 | [G3DSA:1.10.20.10(GENE3D)](http://www.ebi.ac.uk/interpro/signature/G3DSA:1.10.20.10) |
|  |  |  |  |  | [SSF47113 (SUPERFAMILY)](http://www.ebi.ac.uk/interpro/signature/SSF47113) |
|  | [IPR032454](http://www.ebi.ac.uk/interpro/entry/IPR032454) | DOMAIN | Histone H2A, C-terminal domain |  | [PF16211 (PFAM)](http://www.ebi.ac.uk/interpro/signature/PF16211) |
|  | no IPR | HOMOLOGOUS_SUPERFAMILY | Histone-fold |  | [SSF52949 (SUPERFAMILY)](http://www.ebi.ac.uk/interpro/signature/SSF52949) |
|  | no IPR | DOMAIN | Macro domain |  | [G3DSA:3.40.220.10(GENE3D)](http://www.ebi.ac.uk/interpro/signature/G3DSA:3.40.220.10) |
|  |  |  |  |  | mobidb-lite (MOBIDB_LITE) |
|  |  |  |  |  | [PTHR23430 (PANTHER)](http://www.ebi.ac.uk/interpro/signature/PTHR23430) |
|  |  |  |  |  | [PTHR23430:SF69 (PANTHER)](http://www.ebi.ac.uk/interpro/signature/PTHR23430) |
| *RsHELQ* | [IPR001650](http://www.ebi.ac.uk/interpro/entry/IPR001650) | DOMAIN | Helicase, C-terminal |  | [SM00490 (SMART)](http://www.ebi.ac.uk/interpro/signature/SM00490) |
|  |  |  |  |  | [PF00271 (PFAM)](http://www.ebi.ac.uk/interpro/signature/PF00271) |
|  |  |  |  |  | [PS51194(PROSITE_PROFILES)](http://www.ebi.ac.uk/interpro/signature/PS51194) |
|  |  |  |  |  | cd00079 (CDD) |
|  | [IPR011545](http://www.ebi.ac.uk/interpro/entry/IPR011545) | DOMAIN | DEAD/DEAH box helicase domain | GO:0003676, GO:0005524 | [PF00270 (PFAM)](http://www.ebi.ac.uk/interpro/signature/PF00270) |
|  | [IPR014001](http://www.ebi.ac.uk/interpro/entry/IPR014001) | DOMAIN | Helicase superfamily 1/2, ATP-binding domain |  | [SM00487 (SMART)](http://www.ebi.ac.uk/interpro/signature/SM00487) |
|  |  |  |  |  | [PS51192(PROSITE_PROFILES)](http://www.ebi.ac.uk/interpro/signature/PS51192) |
|  | [IPR027417](http://www.ebi.ac.uk/interpro/entry/IPR027417) | HOMOLOGOUS_SUPERFAMILY | P-loop containing nucleoside triphosphate hydrolase |  | [SSF52540(SUPERFAMILY)](http://www.ebi.ac.uk/interpro/signature/SSF52540) |
|  | no IPR | DOMAIN | Helicase, C-terminal |  | [G3DSA:1.10.150.20(GENE3D)](http://www.ebi.ac.uk/interpro/signature/G3DSA:1.10.150.20) |
|  |  |  |  |  | [G3DSA:3.40.50.300(GENE3D)](http://www.ebi.ac.uk/interpro/signature/G3DSA:3.40.50.300) |
|  |  |  |  |  | [G3DSA:1.10.3380.20(GENE3D)](http://www.ebi.ac.uk/interpro/signature/G3DSA:1.10.3380.20) |
|  |  |  |  |  | [G3DSA:3.40.50.300(GENE3D)](http://www.ebi.ac.uk/interpro/signature/G3DSA:3.40.50.300) |
|  |  |  |  |  | mobidb-lite (MOBIDB_LITE) |
|  |  |  |  |  | [PTHR24031:SF357 (PANTHER)](http://www.ebi.ac.uk/interpro/signature/PTHR24031) |
|  |  |  |  |  | [PTHR24031 (PANTHER)](http://www.ebi.ac.uk/interpro/signature/PTHR24031) |
|  |  |  |  |  | cd00046 (CDD) |
|  |  |  |  |  | [SSF158702(SUPERFAMILY)](http://www.ebi.ac.uk/interpro/signature/SSF158702) |
| *RsHERC2* | [IPR000408](http://www.ebi.ac.uk/interpro/entry/IPR000408) | REPEAT | Regulator of chromosome condensation, RCC1 |  | [PR00633 (PRINTS)](http://www.ebi.ac.uk/interpro/signature/PR00633) |
|  |  |  |  |  | [PF00415 (PFAM)](http://www.ebi.ac.uk/interpro/signature/PF00415) |
|  |  |  |  |  | [PS00626(PROSITE_PATTERNS)](http://www.ebi.ac.uk/interpro/signature/PS00626) |
|  |  |  |  |  | [PS50012(PROSITE_PROFILES)](http://www.ebi.ac.uk/interpro/signature/PS50012) |
|  |  |  |  |  | [PS50012(PROSITE_PROFILES)](http://www.ebi.ac.uk/interpro/signature/PS50012) |
|  |  |  |  |  | [PS50012(PROSITE_PROFILES)](http://www.ebi.ac.uk/interpro/signature/PS50012) |
|  |  |  |  |  | [PS50012(PROSITE_PROFILES)](http://www.ebi.ac.uk/interpro/signature/PS50012) |
|  |  |  |  |  | [PS50012(PROSITE_PROFILES)](http://www.ebi.ac.uk/interpro/signature/PS50012) |
|  |  |  |  |  | [PS50012(PROSITE_PROFILES)](http://www.ebi.ac.uk/interpro/signature/PS50012) |
|  |  |  |  |  | [PS50012(PROSITE_PROFILES)](http://www.ebi.ac.uk/interpro/signature/PS50012) |
|  |  |  |  |  | [PS50012(PROSITE_PROFILES)](http://www.ebi.ac.uk/interpro/signature/PS50012) |
|  |  |  |  |  | [PS50012(PROSITE_PROFILES)](http://www.ebi.ac.uk/interpro/signature/PS50012) |
|  |  |  |  |  | [PS50012(PROSITE_PROFILES)](http://www.ebi.ac.uk/interpro/signature/PS50012) |
|  |  |  |  |  | [PS50012(PROSITE_PROFILES)](http://www.ebi.ac.uk/interpro/signature/PS50012) |
|  |  |  |  |  | [PS50012(PROSITE_PROFILES)](http://www.ebi.ac.uk/interpro/signature/PS50012) |
|  |  |  |  |  | [PS50012(PROSITE_PROFILES)](http://www.ebi.ac.uk/interpro/signature/PS50012) |
|  |  |  |  |  | [PS50012(PROSITE_PROFILES)](http://www.ebi.ac.uk/interpro/signature/PS50012) |
|  | [IPR000433](http://www.ebi.ac.uk/interpro/entry/IPR000433) | DOMAIN | Zinc finger, ZZ-type | GO:0008270 | [SM00291 (SMART)](http://www.ebi.ac.uk/interpro/signature/SM00291) |
|  |  |  |  |  | [PF00569 (PFAM)](http://www.ebi.ac.uk/interpro/signature/PF00569) |
|  |  |  |  |  | [PS50135(PROSITE_PROFILES)](http://www.ebi.ac.uk/interpro/signature/PS50135) |
|  | [IPR000569](http://www.ebi.ac.uk/interpro/entry/IPR000569) | DOMAIN | HECT domain | GO:0004842 | [SM00119 (SMART)](http://www.ebi.ac.uk/interpro/signature/SM00119) |
|  |  |  |  |  | [PF00632 (PFAM)](http://www.ebi.ac.uk/interpro/signature/PF00632) |
|  |  |  |  |  | [PS50237(PROSITE_PROFILES)](http://www.ebi.ac.uk/interpro/signature/PS50237) |
|  |  |  |  |  | cd00078 (CDD) |
|  | [IPR004939](http://www.ebi.ac.uk/interpro/entry/IPR004939) | DOMAIN | APC10/DOC domain |  | [SM01337 (SMART)](http://www.ebi.ac.uk/interpro/signature/SM01337) |
|  |  |  |  |  | [PF03256 (PFAM)](http://www.ebi.ac.uk/interpro/signature/PF03256) |
|  |  |  |  |  | [PS51284(PROSITE_PROFILES)](http://www.ebi.ac.uk/interpro/signature/PS51284) |
|  | [IPR008979](http://www.ebi.ac.uk/interpro/entry/IPR008979) | HOMOLOGOUS_SUPERFAMILY | Galactose-binding-like domain superfamily |  | [G3DSA:2.60.120.260(GENE3D)](http://www.ebi.ac.uk/interpro/signature/G3DSA:2.60.120.260) |
|  |  |  |  |  | [SSF49785(SUPERFAMILY)](http://www.ebi.ac.uk/interpro/signature/SSF49785) |
|  | [IPR009091](http://www.ebi.ac.uk/interpro/entry/IPR009091) | HOMOLOGOUS_SUPERFAMILY | Regulator of chromosome condensation 1/beta-lactamase-inhibitor protein II |  | [G3DSA:2.130.10.30(GENE3D)](http://www.ebi.ac.uk/interpro/signature/G3DSA:2.130.10.30) |
|  |  |  |  |  | [SSF50985(SUPERFAMILY)](http://www.ebi.ac.uk/interpro/signature/SSF50985) |
|  |  |  |  |  | [SSF50985(SUPERFAMILY)](http://www.ebi.ac.uk/interpro/signature/SSF50985) |
|  | [IPR010606](http://www.ebi.ac.uk/interpro/entry/IPR010606) | DOMAIN | Mib-herc2 | GO:0016567, GO:0046872, GO:0004842 | [PF06701 (PFAM)](http://www.ebi.ac.uk/interpro/signature/PF06701) |
|  |  |  |  |  | [PS51416(PROSITE_PROFILES)](http://www.ebi.ac.uk/interpro/signature/PS51416) |
|  | [IPR014722](http://www.ebi.ac.uk/interpro/entry/IPR014722) | HOMOLOGOUS_SUPERFAMILY | Ribosomal protein L2, domain 2 |  | [G3DSA:2.30.30.30(GENE3D)](http://www.ebi.ac.uk/interpro/signature/G3DSA:2.30.30.30) |
|  | [IPR021097](http://www.ebi.ac.uk/interpro/entry/IPR021097) | DOMAIN | CPH domain |  | [PF11515 (PFAM)](http://www.ebi.ac.uk/interpro/signature/PF11515) |
|  | [IPR035983](http://www.ebi.ac.uk/interpro/entry/IPR035983) | HOMOLOGOUS_SUPERFAMILY | HECT, E3 ligase catalytic domain | GO:0004842 | [SSF56204(SUPERFAMILY)](http://www.ebi.ac.uk/interpro/signature/SSF56204) |
|  | [IPR037252](http://www.ebi.ac.uk/interpro/entry/IPR037252) | HOMOLOGOUS_SUPERFAMILY | Mib/herc2 domain superfamily | GO:0004842, GO:0046872 | [G3DSA:2.30.30.920(GENE3D)](http://www.ebi.ac.uk/interpro/signature/G3DSA:2.30.30.920) |
|  |  |  |  |  | [SSF159034(SUPERFAMILY)](http://www.ebi.ac.uk/interpro/signature/SSF159034) |
|  | [IPR037976](http://www.ebi.ac.uk/interpro/entry/IPR037976) | DOMAIN | HERC2, APC10 domain |  | cd08664 (CDD) |
|  | no IPR | HOMOLOGOUS_SUPERFAMILY | Galactose-binding-like domain superfamily |  | [SSF63748(SUPERFAMILY)](http://www.ebi.ac.uk/interpro/signature/SSF63748) |
|  | no IPR | DOMAIN | HECT domain |  | [G3DSA:3.90.1750.10(GENE3D)](http://www.ebi.ac.uk/interpro/signature/G3DSA:3.90.1750.10) |
|  | no IPR | HOMOLOGOUS_SUPERFAMILY | Mib/herc2 domain superfamily |  | [SSF57850(SUPERFAMILY)](http://www.ebi.ac.uk/interpro/signature/SSF57850) |
|  | no IPR | REPEAT | Regulator of chromosome condensation, RCC1 |  | mobidb-lite (MOBIDB_LITE) |
|  |  |  |  |  | mobidb-lite (MOBIDB_LITE) |
|  |  |  |  |  | [PTHR11254(PANTHER)](http://www.ebi.ac.uk/interpro/signature/PTHR11254) |
|  |  |  |  |  | [PTHR11254:SF325 (PANTHER)](http://www.ebi.ac.uk/interpro/signature/PTHR11254) |
|  | no IPR | HOMOLOGOUS_SUPERFAMILY | Ribosomal protein L2, domain 2 |  | [G3DSA:3.30.60.90(GENE3D)](http://www.ebi.ac.uk/interpro/signature/G3DSA:3.30.60.90) |
|  |  |  |  |  | [G3DSA:3.30.2160.10(GENE3D)](http://www.ebi.ac.uk/interpro/signature/G3DSA:3.30.2160.10) |
|  | no IPR | DOMAIN | Zinc finger, ZZ-type |  | [G3DSA:3.30.2410.10(GENE3D)](http://www.ebi.ac.uk/interpro/signature/G3DSA:3.30.2410.10) |
| *RsHUS1* | [IPR007150](http://www.ebi.ac.uk/interpro/entry/IPR007150) | FAMILY | Checkpoint protein Hus1/Mec3 | GO:0030896, GO:0000077 | [PF04005 (PFAM)](http://www.ebi.ac.uk/interpro/signature/PF04005) |
|  | [IPR016580](http://www.ebi.ac.uk/interpro/entry/IPR016580) | FAMILY | Cell cycle checkpoint, Hus1 | GO:0005730, GO:0030896, GO:0000077 | PIRSF011312 (PIRSF) |
|  | no IPR | FAMILY | Cell cycle checkpoint, Hus1 |  | [G3DSA:3.70.10.10 (GENE3D)](http://www.ebi.ac.uk/interpro/signature/G3DSA:3.70.10.10) |
|  |  |  |  |  | [PTHR12900:SF4 (PANTHER)](http://www.ebi.ac.uk/interpro/signature/PTHR12900) |
|  |  |  |  |  | [PTHR12900 (PANTHER)](http://www.ebi.ac.uk/interpro/signature/PTHR12900) |
|  |  |  |  |  | SIGNAL_PEPTIDE_N_REGION (PHOBIUS) |
|  |  |  |  |  | SIGNAL_PEPTIDE_H_REGION (PHOBIUS) |
|  |  |  |  |  | SIGNAL_PEPTIDE (PHOBIUS) |
|  |  |  |  |  | SIGNAL_PEPTIDE_C_REGION (PHOBIUS) |
|  |  |  |  |  | NON_CYTOPLASMIC_DOMAIN (PHOBIUS) |
| *RsKU70* | [IPR005160](http://www.ebi.ac.uk/interpro/entry/IPR005160) | DOMAIN | Ku70/Ku80 C-terminal arm | GO:0006303, GO:0003677, GO:0004003 | [PF03730 (PFAM)](http://www.ebi.ac.uk/interpro/signature/PF03730) |
|  | [IPR005161](http://www.ebi.ac.uk/interpro/entry/IPR005161) | DOMAIN | Ku70/Ku80, N-terminal alpha/beta |  | [PF03731 (PFAM)](http://www.ebi.ac.uk/interpro/signature/PF03731) |
|  | [IPR006164](http://www.ebi.ac.uk/interpro/entry/IPR006164) | DOMAIN | Ku70/Ku80 beta-barrel domain | GO:0003677, GO:0006303 | [SM00559 (SMART)](http://www.ebi.ac.uk/interpro/signature/SM00559) |
|  |  |  |  |  | [PF02735 (PFAM)](http://www.ebi.ac.uk/interpro/signature/PF02735) |
|  | [IPR006165](http://www.ebi.ac.uk/interpro/entry/IPR006165) | FAMILY | Ku70 | GO:0003684, GO:0005634, GO:0006303, GO:0042162, GO:0043564, GO:0000723, GO:0003677 | [TIGR00578(TIGRFAM)](http://www.ebi.ac.uk/interpro/signature/TIGR00578) |
|  |  |  |  |  | PIRSF003033 (PIRSF) |
|  |  |  |  |  | [PTHR12604:SF2 (PANTHER)](http://www.ebi.ac.uk/interpro/signature/PTHR12604) |
|  | [IPR016194](http://www.ebi.ac.uk/interpro/entry/IPR016194) | HOMOLOGOUS_SUPERFAMILY | SPOC-like, C-terminal domain superfamily |  | [G3DSA:2.40.290.10(GENE3D)](http://www.ebi.ac.uk/interpro/signature/G3DSA:2.40.290.10) |
|  |  |  |  |  | [SSF100939(SUPERFAMILY)](http://www.ebi.ac.uk/interpro/signature/SSF100939) |
|  | [IPR027388](http://www.ebi.ac.uk/interpro/entry/IPR027388) | HOMOLOGOUS_SUPERFAMILY | Ku70, bridge and pillars domain superfamily |  | [G3DSA:4.10.970.10(GENE3D)](http://www.ebi.ac.uk/interpro/signature/G3DSA:4.10.970.10) |
|  | [IPR036361](http://www.ebi.ac.uk/interpro/entry/IPR036361) | HOMOLOGOUS_SUPERFAMILY | SAP domain superfamily |  | [SSF68906(SUPERFAMILY)](http://www.ebi.ac.uk/interpro/signature/SSF68906) |
|  | [IPR036465](http://www.ebi.ac.uk/interpro/entry/IPR036465) | HOMOLOGOUS_SUPERFAMILY | von Willebrand factor A-like domain superfamily |  | [G3DSA:3.40.50.410(GENE3D)](http://www.ebi.ac.uk/interpro/signature/G3DSA:3.40.50.410) |
|  |  |  |  |  | [SSF53300(SUPERFAMILY)](http://www.ebi.ac.uk/interpro/signature/SSF53300) |
|  | no IPR | FAMILY | Ku70 |  | mobidb-lite (MOBIDB_LITE) |
|  |  |  |  |  | [PTHR12604(PANTHER)](http://www.ebi.ac.uk/interpro/signature/PTHR12604) |
|  |  |  |  |  | cd01458 (CDD) |
|  |  |  |  |  | cd00788 (CDD) |
|  | no IPR | HOMOLOGOUS_SUPERFAMILY | SPOC-like, C-terminal domain superfamily |  | [G3DSA:1.10.1600.10(GENE3D)](http://www.ebi.ac.uk/interpro/signature/G3DSA:1.10.1600.10) |
|  | no IPR |  |  |  | Coil (COILS) |
| *RsKU80* | [IPR002035](http://www.ebi.ac.uk/interpro/entry/IPR002035) | DOMAIN | von Willebrand factor, type A |  | [PS50234(PROSITE_PROFILES)](http://www.ebi.ac.uk/interpro/signature/PS50234) |
|  | [IPR005161](http://www.ebi.ac.uk/interpro/entry/IPR005161) | DOMAIN | Ku70/Ku80, N-terminal alpha/beta |  | [PF03731 (PFAM)](http://www.ebi.ac.uk/interpro/signature/PF03731) |
|  | [IPR006164](http://www.ebi.ac.uk/interpro/entry/IPR006164) | DOMAIN | Ku70/Ku80 beta-barrel domain | GO:0006303, GO:0003677 | [SM00559 (SMART)](http://www.ebi.ac.uk/interpro/signature/SM00559) |
|  |  |  |  |  | [PF02735 (PFAM)](http://www.ebi.ac.uk/interpro/signature/PF02735) |
|  | [IPR014893](http://www.ebi.ac.uk/interpro/entry/IPR014893) | DOMAIN | Ku, C-terminal | GO:0016817 | [PF08785 (PFAM)](http://www.ebi.ac.uk/interpro/signature/PF08785) |
|  | [IPR016194](http://www.ebi.ac.uk/interpro/entry/IPR016194) | HOMOLOGOUS_SUPERFAMILY | SPOC-like, C-terminal domain superfamily |  | [G3DSA:2.40.290.10(GENE3D)](http://www.ebi.ac.uk/interpro/signature/G3DSA:2.40.290.10) |
|  |  |  |  |  | [SSF100939(SUPERFAMILY)](http://www.ebi.ac.uk/interpro/signature/SSF100939) |
|  | [IPR024193](http://www.ebi.ac.uk/interpro/entry/IPR024193) | FAMILY | Ku80 | GO:0006303, GO:0043564, GO:0003684, GO:0006310, GO:0000723, GO:0003677, GO:0005634, GO:0042162 | PIRSF016570 (PIRSF) |
|  |  |  |  |  | [PTHR12604:SF4 (PANTHER)](http://www.ebi.ac.uk/interpro/signature/PTHR12604) |
|  | [IPR036465](http://www.ebi.ac.uk/interpro/entry/IPR036465) | HOMOLOGOUS_SUPERFAMILY | von Willebrand factor A-like domain superfamily |  | [G3DSA:3.40.50.410(GENE3D)](http://www.ebi.ac.uk/interpro/signature/G3DSA:3.40.50.410) |
|  |  |  |  |  | [SSF53300(SUPERFAMILY)](http://www.ebi.ac.uk/interpro/signature/SSF53300) |
|  | [IPR036494](http://www.ebi.ac.uk/interpro/entry/IPR036494) | HOMOLOGOUS_SUPERFAMILY | Ku, C-terminal domain superfamily |  | [G3DSA:1.25.40.240(GENE3D)](http://www.ebi.ac.uk/interpro/signature/G3DSA:1.25.40.240) |
|  |  |  |  |  | [SSF101420(SUPERFAMILY)](http://www.ebi.ac.uk/interpro/signature/SSF101420) |
|  | no IPR | HOMOLOGOUS_SUPERFAMILY | Ku, C-terminal domain superfamily |  | [PTHR12604(PANTHER)](http://www.ebi.ac.uk/interpro/signature/PTHR12604) |
|  | no IPR | DOMAIN | Ku70/Ku80 beta-barrel domain |  | [G3DSA:1.10.1600.10(GENE3D)](http://www.ebi.ac.uk/interpro/signature/G3DSA:1.10.1600.10) |
|  | no IPR | DOMAIN | von Willebrand factor, type A |  | cd00873 (CDD) |
| *RsLIG4* | [IPR000977](http://www.ebi.ac.uk/interpro/entry/IPR000977) | FAMILY | DNA ligase, ATP-dependent | GO:0006310, GO:0071897, GO:0003910, GO:0006281, GO:0005524 | [TIGR00574 (TIGRFAM)](http://www.ebi.ac.uk/interpro/signature/TIGR00574) |
|  | [IPR001357](http://www.ebi.ac.uk/interpro/entry/IPR001357) | DOMAIN | BRCT domain |  | [SM00292 (SMART)](http://www.ebi.ac.uk/interpro/signature/SM00292) |
|  |  |  |  |  | [PF16589 (PFAM)](http://www.ebi.ac.uk/interpro/signature/PF16589) |
|  |  |  |  |  | [PS50172(PROSITE_PROFILES)](http://www.ebi.ac.uk/interpro/signature/PS50172) |
|  |  |  |  |  | cd00027 (CDD) |
|  | [IPR012308](http://www.ebi.ac.uk/interpro/entry/IPR012308) | DOMAIN | DNA ligase, ATP-dependent, N-terminal | GO:0006281, GO:0003677, GO:0003910, GO:0006310 | [PF04675 (PFAM)](http://www.ebi.ac.uk/interpro/signature/PF04675) |
|  | [IPR012309](http://www.ebi.ac.uk/interpro/entry/IPR012309) | DOMAIN | DNA ligase, ATP-dependent, C-terminal | GO:0006310, GO:0003910, GO:0006281 | [PF04679 (PFAM)](http://www.ebi.ac.uk/interpro/signature/PF04679) |
|  | [IPR012310](http://www.ebi.ac.uk/interpro/entry/IPR012310) | DOMAIN | DNA ligase, ATP-dependent, central | GO:0006281, GO:0003910, GO:0006310, GO:0005524 | [PF01068 (PFAM)](http://www.ebi.ac.uk/interpro/signature/PF01068) |
|  |  |  |  |  | [PS50160(PROSITE_PROFILES)](http://www.ebi.ac.uk/interpro/signature/PS50160) |
|  | [IPR012340](http://www.ebi.ac.uk/interpro/entry/IPR012340) | HOMOLOGOUS_SUPERFAMILY | Nucleic acid-binding, OB-fold |  | [SSF50249(SUPERFAMILY)](http://www.ebi.ac.uk/interpro/signature/SSF50249) |
|  | [IPR016059](http://www.ebi.ac.uk/interpro/entry/IPR016059) | CONSERVED_SITE | DNA ligase, ATP-dependent, conserved site | GO:0051103, GO:0003909 | [PS00333(PROSITE_PATTERNS)](http://www.ebi.ac.uk/interpro/signature/PS00333) |
|  | [IPR021536](http://www.ebi.ac.uk/interpro/entry/IPR021536) | DOMAIN | DNA ligase IV domain | GO:0003910 | [PF11411 (PFAM)](http://www.ebi.ac.uk/interpro/signature/PF11411) |
|  | [IPR029710](http://www.ebi.ac.uk/interpro/entry/IPR029710) | FAMILY | DNA ligase 4 | GO:0003677, GO:0006281, GO:0051103, GO:0005524, GO:0003910 | [PTHR10459:SF7 (PANTHER)](http://www.ebi.ac.uk/interpro/signature/PTHR10459) |
|  | [IPR036420](http://www.ebi.ac.uk/interpro/entry/IPR036420) | HOMOLOGOUS_SUPERFAMILY | BRCT domain superfamily |  | [G3DSA:3.40.50.10190 (GENE3D)](http://www.ebi.ac.uk/interpro/signature/G3DSA:3.40.50.1019) |
|  |  |  |  |  | [SSF52113(SUPERFAMILY)](http://www.ebi.ac.uk/interpro/signature/SSF52113) |
|  | [IPR036599](http://www.ebi.ac.uk/interpro/entry/IPR036599) | HOMOLOGOUS_SUPERFAMILY | DNA ligase, ATP-dependent, N-terminal domain superfamily | GO:0003677, GO:0006281, GO:0003910, GO:0006310 | [G3DSA:1.10.3260.10(GENE3D)](http://www.ebi.ac.uk/interpro/signature/G3DSA:1.10.3260.10) |
|  |  |  |  |  | [SSF117018(SUPERFAMILY)](http://www.ebi.ac.uk/interpro/signature/SSF117018) |
|  | no IPR | DOMAIN | BRCT domain |  | cd07968 (CDD) |
|  |  |  |  |  | cd07903 (CDD) |
|  | no IPR | HOMOLOGOUS_SUPERFAMILY | BRCT domain superfamily |  | [G3DSA:2.40.50.140(GENE3D)](http://www.ebi.ac.uk/interpro/signature/G3DSA:2.40.50.140) |
|  |  |  |  |  | [SSF56091(SUPERFAMILY)](http://www.ebi.ac.uk/interpro/signature/SSF56091) |
|  | no IPR | FAMILY | DNA ligase, ATP-dependent |  | [PTHR10459(PANTHER)](http://www.ebi.ac.uk/interpro/signature/PTHR10459) |
|  | no IPR | DOMAIN | DNA ligase, ATP-dependent, N-terminal |  | [G3DSA:3.30.470.30(GENE3D)](http://www.ebi.ac.uk/interpro/signature/G3DSA:3.30.470.30) |
| *RsMAPK14* | [IPR000719](http://www.ebi.ac.uk/interpro/entry/IPR000719) | DOMAIN | Protein kinase domain | GO:0005524, GO:0004672, GO:0006468 | [SM00220 (SMART)](http://www.ebi.ac.uk/interpro/signature/SM00220) |
|  |  |  |  |  | [PF00069 (PFAM)](http://www.ebi.ac.uk/interpro/signature/PF00069) |
|  |  |  |  |  | [PS50011(PROSITE_PROFILES)](http://www.ebi.ac.uk/interpro/signature/PS50011) |
|  | [IPR003527](http://www.ebi.ac.uk/interpro/entry/IPR003527) | CONSERVED_SITE | Mitogen-activated protein (MAP) kinase, conserved site | GO:0004707, GO:0005524, GO:0006468 | [PS01351(PROSITE_PATTERNS)](http://www.ebi.ac.uk/interpro/signature/PS01351) |
|  | [IPR008352](http://www.ebi.ac.uk/interpro/entry/IPR008352) | FAMILY | Mitogen-activated protein (MAP) kinase, p38 | GO:0006468, GO:0005524, GO:0004707 | [PR01773 (PRINTS)](http://www.ebi.ac.uk/interpro/signature/PR01773) |
|  | [IPR011009](http://www.ebi.ac.uk/interpro/entry/IPR011009) | HOMOLOGOUS_SUPERFAMILY | Protein kinase-like domain superfamily |  | [SSF56112(SUPERFAMILY)](http://www.ebi.ac.uk/interpro/signature/SSF56112) |
|  | [IPR017441](http://www.ebi.ac.uk/interpro/entry/IPR017441) | BINDING_SITE | Protein kinase, ATP binding site | GO:0005524 | [PS00107(PROSITE_PATTERNS)](http://www.ebi.ac.uk/interpro/signature/PS00107) |
|  | no IPR | DOMAIN | Protein kinase domain |  | [G3DSA:3.30.200.20(GENE3D)](http://www.ebi.ac.uk/interpro/signature/G3DSA:3.30.200.20) |
|  |  |  |  |  | [G3DSA:1.10.510.10(GENE3D)](http://www.ebi.ac.uk/interpro/signature/G3DSA:1.10.510.10) |
|  |  |  |  |  | PIRSF000654 (PIRSF) |
|  |  |  |  |  | [PTHR24055(PANTHER)](http://www.ebi.ac.uk/interpro/signature/PTHR24055) |
|  |  |  |  |  | [PTHR24055:SF110 (PANTHER)](http://www.ebi.ac.uk/interpro/signature/PTHR24055) |
|  |  |  |  |  | cd07851 (CDD) |
| *RsMAPKAPK2* | [IPR000719](http://www.ebi.ac.uk/interpro/entry/IPR000719) | DOMAIN | Protein kinase domain | GO:0004672, GO:0006468, GO:0005524 | [SM00220 (SMART)](http://www.ebi.ac.uk/interpro/signature/SM00220) |
|  |  |  |  |  | [PF00069 (PFAM)](http://www.ebi.ac.uk/interpro/signature/PF00069) |
|  |  |  |  |  | [PS50011(PROSITE_PROFILES)](http://www.ebi.ac.uk/interpro/signature/PS50011) |
|  | [IPR008271](http://www.ebi.ac.uk/interpro/entry/IPR008271) | ACTIVE_SITE | Serine/threonine-protein kinase, active site | GO:0006468, GO:0004672 | [PS00108(PROSITE_PATTERNS)](http://www.ebi.ac.uk/interpro/signature/PS00108) |
|  | [IPR011009](http://www.ebi.ac.uk/interpro/entry/IPR011009) | HOMOLOGOUS_SUPERFAMILY | Protein kinase-like domain superfamily |  | [SSF56112 (SUPERFAMILY)](http://www.ebi.ac.uk/interpro/signature/SSF56112) |
|  | [IPR027442](http://www.ebi.ac.uk/interpro/entry/IPR027442) | HOMOLOGOUS_SUPERFAMILY | MAP kinase activated protein kinase, C-terminal |  | [G3DSA:4.10.1170.10(GENE3D)](http://www.ebi.ac.uk/interpro/signature/G3DSA:4.10.1170.10) |
|  | no IPR | HOMOLOGOUS_SUPERFAMILY | MAP kinase activated protein kinase, C-terminal |  | [G3DSA:1.10.510.10 (GENE3D)](http://www.ebi.ac.uk/interpro/signature/G3DSA:1.10.510.10) |
|  |  |  |  |  | PIRSF000654 (PIRSF) |
|  | no IPR | DOMAIN | Protein kinase domain |  | [G3DSA:3.30.200.20 (GENE3D)](http://www.ebi.ac.uk/interpro/signature/G3DSA:3.30.200.20) |
|  |  |  |  |  | mobidb-lite (MOBIDB_LITE) |
|  |  |  |  |  | [PTHR24349:SF64 (PANTHER)](http://www.ebi.ac.uk/interpro/signature/PTHR24349) |
|  |  |  |  |  | [PTHR24349 (PANTHER)](http://www.ebi.ac.uk/interpro/signature/PTHR24349) |
|  |  |  |  |  | cd14089 (CDD) |
|  | no IPR | ACTIVE_SITE | Serine/threonine-protein kinase, active site |  | NON_CYTOPLASMIC_DOMAIN (PHOBIUS) |
|  |  |  |  |  | TRANSMEMBRANE (PHOBIUS) |
|  |  |  |  |  | CYTOPLASMIC_DOMAIN (PHOBIUS) |
| *RsMCPH1* | [IPR001357](http://www.ebi.ac.uk/interpro/entry/IPR001357) | DOMAIN | BRCT domain |  | [SM00292 (SMART)](http://www.ebi.ac.uk/interpro/signature/SM00292) |
|  |  |  |  |  | [PF00533 (PFAM)](http://www.ebi.ac.uk/interpro/signature/PF00533) |
|  |  |  |  |  | [PF12738 (PFAM)](http://www.ebi.ac.uk/interpro/signature/PF12738) |
|  |  |  |  |  | [PS50172 (PROSITE_PROFILES)](http://www.ebi.ac.uk/interpro/signature/PS50172) |
|  |  |  |  |  | [PS50172 (PROSITE_PROFILES)](http://www.ebi.ac.uk/interpro/signature/PS50172) |
|  |  |  |  |  | [PS50172 (PROSITE_PROFILES)](http://www.ebi.ac.uk/interpro/signature/PS50172) |
|  |  |  |  |  | cd00027 (CDD) |
|  |  |  |  |  | cd00027 (CDD) |
|  |  |  |  |  | cd00027 (CDD) |
|  | [IPR022047](http://www.ebi.ac.uk/interpro/entry/IPR022047) | FAMILY | Microcephalin-like |  | [PTHR14625 (PANTHER)](http://www.ebi.ac.uk/interpro/signature/PTHR14625) |
|  | [IPR036420](http://www.ebi.ac.uk/interpro/entry/IPR036420) | HOMOLOGOUS_SUPERFAMILY | BRCT domain superfamily |  | [G3DSA:3.40.50.10190 (GENE3D)](http://www.ebi.ac.uk/interpro/signature/G3DSA:3.40.50.1019) |
|  |  |  |  |  | [G3DSA:3.40.50.10190 (GENE3D)](http://www.ebi.ac.uk/interpro/signature/G3DSA:3.40.50.1019) |
|  |  |  |  |  | [G3DSA:3.40.50.10190 (GENE3D)](http://www.ebi.ac.uk/interpro/signature/G3DSA:3.40.50.1019) |
|  |  |  |  |  | [SSF52113 (SUPERFAMILY)](http://www.ebi.ac.uk/interpro/signature/SSF52113) |
|  |  |  |  |  | [SSF52113 (SUPERFAMILY)](http://www.ebi.ac.uk/interpro/signature/SSF52113) |
|  |  |  |  |  | [SSF52113 (SUPERFAMILY)](http://www.ebi.ac.uk/interpro/signature/SSF52113) |
|  | no IPR | DOMAIN | BRCT domain |  | mobidb-lite (MOBIDB_LITE) |
|  |  |  |  |  | mobidb-lite (MOBIDB_LITE) |
|  |  |  |  |  | mobidb-lite (MOBIDB_LITE) |
| *RsMDC1* | [IPR000253](http://www.ebi.ac.uk/interpro/entry/IPR000253) | DOMAIN | Forkhead-associated (FHA) domain | GO:0005515 | [SM00240 (SMART)](http://www.ebi.ac.uk/interpro/signature/SM00240) |
|  |  |  |  |  | [PF00498 (PFAM)](http://www.ebi.ac.uk/interpro/signature/PF00498) |
|  |  |  |  |  | [PS50006 (PROSITE_PROFILES)](http://www.ebi.ac.uk/interpro/signature/PS50006) |
|  |  |  |  |  | cd00060 (CDD) |
|  | [IPR001357](http://www.ebi.ac.uk/interpro/entry/IPR001357) | DOMAIN | BRCT domain |  | [SM00292 (SMART)](http://www.ebi.ac.uk/interpro/signature/SM00292) |
|  |  |  |  |  | [PF16770 (PFAM)](http://www.ebi.ac.uk/interpro/signature/PF16770) |
|  |  |  |  |  | [PS50172 (PROSITE_PROFILES)](http://www.ebi.ac.uk/interpro/signature/PS50172) |
|  |  |  |  |  | cd00027 (CDD) |
|  | [IPR008984](http://www.ebi.ac.uk/interpro/entry/IPR008984) | HOMOLOGOUS_SUPERFAMILY | SMAD/FHA domain superfamily | GO:0005515 | [SSF49879 (SUPERFAMILY)](http://www.ebi.ac.uk/interpro/signature/SSF49879) |
|  | [IPR036420](http://www.ebi.ac.uk/interpro/entry/IPR036420) | HOMOLOGOUS_SUPERFAMILY | BRCT domain superfamily |  | [G3DSA:3.40.50.10190 (GENE3D)](http://www.ebi.ac.uk/interpro/signature/G3DSA:3.40.50.1019) |
|  |  |  |  |  | [G3DSA:3.40.50.10190 (GENE3D)](http://www.ebi.ac.uk/interpro/signature/G3DSA:3.40.50.1019) |
|  |  |  |  |  | [SSF52113 (SUPERFAMILY)](http://www.ebi.ac.uk/interpro/signature/SSF52113) |
|  | no IPR | DOMAIN | BRCT domain |  | [G3DSA:2.60.200.20 (GENE3D)](http://www.ebi.ac.uk/interpro/signature/G3DSA:2.60.200.20) |
|  | no IPR | DOMAIN | Forkhead-associated (FHA) domain |  | mobidb-lite (MOBIDB_LITE) |
|  |  |  |  |  | mobidb-lite (MOBIDB_LITE) |
|  |  |  |  |  | mobidb-lite (MOBIDB_LITE) |
|  |  |  |  |  | mobidb-lite (MOBIDB_LITE) |
|  |  |  |  |  | mobidb-lite (MOBIDB_LITE) |
|  |  |  |  |  | mobidb-lite (MOBIDB_LITE) |
|  |  |  |  |  | mobidb-lite (MOBIDB_LITE) |
|  |  |  |  |  | mobidb-lite (MOBIDB_LITE) |
|  |  |  |  |  | [PTHR23196 (PANTHER)](http://www.ebi.ac.uk/interpro/signature/PTHR23196) |
|  |  |  |  |  | [PTHR23196 (PANTHER)](http://www.ebi.ac.uk/interpro/signature/PTHR23196) |
|  |  |  |  |  | [PTHR23196:SF16 (PANTHER)](http://www.ebi.ac.uk/interpro/signature/PTHR23196) |
|  |  |  |  |  | [PTHR23196:SF16 (PANTHER)](http://www.ebi.ac.uk/interpro/signature/PTHR23196) |
|  |  |  |  |  | [PTHR23196:SF16 (PANTHER)](http://www.ebi.ac.uk/interpro/signature/PTHR23196) |
| *RsMERIT40* | [IPR026126](http://www.ebi.ac.uk/interpro/entry/IPR026126) | FAMILY | BRISC and BRCA1-A complex member 1 | GO:0070531, GO:0070552, GO:0045739 | [PTHR15660 (PANTHER)](http://www.ebi.ac.uk/interpro/signature/PTHR15660) |
|  | [IPR036465](http://www.ebi.ac.uk/interpro/entry/IPR036465) | HOMOLOGOUS_SUPERFAMILY | von Willebrand factor A-like domain superfamily |  | [G3DSA:3.40.50.410(GENE3D)](http://www.ebi.ac.uk/interpro/signature/G3DSA:3.40.50.410) |
|  | no IPR | HOMOLOGOUS_SUPERFAMILY | von Willebrand factor A-like domain superfamily |  | mobidb-lite (MOBIDB_LITE) |
| *RsMGMT* | [IPR001497](http://www.ebi.ac.uk/interpro/entry/IPR001497) | ACTIVE_SITE | Methylated-DNA-[protein]-cysteine S-methyltransferase, active site | GO:0006281, GO:0003908 | [PS00374(PROSITE_PATTERNS)](http://www.ebi.ac.uk/interpro/signature/PS00374) |
|  | [IPR014048](http://www.ebi.ac.uk/interpro/entry/IPR014048) | DOMAIN | Methylated-DNA-[protein]-cysteine S-methyltransferase, DNA binding | GO:0006281, GO:0003824 | [PF01035 (PFAM)](http://www.ebi.ac.uk/interpro/signature/PF01035) |
|  |  |  |  |  | [TIGR00589 (TIGRFAM)](http://www.ebi.ac.uk/interpro/signature/TIGR00589) |
|  |  |  |  |  | cd06445 (CDD) |
|  | [IPR036217](http://www.ebi.ac.uk/interpro/entry/IPR036217) | HOMOLOGOUS_SUPERFAMILY | Methylated DNA-protein cysteine methyltransferase, DNA binding domain |  | [SSF46767(SUPERFAMILY)](http://www.ebi.ac.uk/interpro/signature/SSF46767) |
|  | [IPR036388](http://www.ebi.ac.uk/interpro/entry/IPR036388) | HOMOLOGOUS_SUPERFAMILY | Winged helix-like DNA-binding domain superfamily |  | [G3DSA:1.10.10.10(GENE3D)](http://www.ebi.ac.uk/interpro/signature/G3DSA:1.10.10.10) |
|  | [IPR036631](http://www.ebi.ac.uk/interpro/entry/IPR036631) | HOMOLOGOUS_SUPERFAMILY | Methylated DNA-protein cysteine methyltransferase domain superfamily | GO:0006281, GO:0003908 | [SSF53155(SUPERFAMILY)](http://www.ebi.ac.uk/interpro/signature/SSF53155) |
|  | no IPR | DOMAIN | Methylated-DNA-[protein]-cysteine S-methyltransferase, DNA binding |  | [PTHR10815(PANTHER)](http://www.ebi.ac.uk/interpro/signature/PTHR10815) |
|  |  |  |  |  | [PTHR10815:SF5 (PANTHER)](http://www.ebi.ac.uk/interpro/signature/PTHR10815) |
|  | no IPR |  |  |  | [G3DSA:3.30.160.70(GENE3D)](http://www.ebi.ac.uk/interpro/signature/G3DSA:3.30.160.70) |
| *RsMLH1* | [IPR002099](http://www.ebi.ac.uk/interpro/entry/IPR002099) | FAMILY | DNA mismatch repair protein family | GO:0030983, GO:0006298, GO:0005524 | [TIGR00585 (TIGRFAM)](http://www.ebi.ac.uk/interpro/signature/TIGR00585) |
|  | [IPR003594](http://www.ebi.ac.uk/interpro/entry/IPR003594) | DOMAIN | Histidine kinase/HSP90-like ATPase |  | cd00075 (CDD) |
|  | [IPR011186](http://www.ebi.ac.uk/interpro/entry/IPR011186) | FAMILY | DNA mismatch repair protein Mlh1 | GO:0006298, GO:0032300 | [PTHR10073:SF40 (PANTHER)](http://www.ebi.ac.uk/interpro/signature/PTHR10073) |
|  | [IPR013507](http://www.ebi.ac.uk/interpro/entry/IPR013507) | DOMAIN | DNA mismatch repair protein, C-terminal | GO:0030983, GO:0005524, GO:0006298 | [SM01340 (SMART)](http://www.ebi.ac.uk/interpro/signature/SM01340) |
|  |  |  |  |  | [PF01119 (PFAM)](http://www.ebi.ac.uk/interpro/signature/PF01119) |
|  | [IPR014721](http://www.ebi.ac.uk/interpro/entry/IPR014721) | HOMOLOGOUS_SUPERFAMILY | Ribosomal protein S5 domain 2-type fold, subgroup |  | [G3DSA:3.30.230.10(GENE3D)](http://www.ebi.ac.uk/interpro/signature/G3DSA:3.30.230.10) |
|  | [IPR014762](http://www.ebi.ac.uk/interpro/entry/IPR014762) | CONSERVED_SITE | DNA mismatch repair, conserved site |  | [PS00058(PROSITE_PATTERNS)](http://www.ebi.ac.uk/interpro/signature/PS00058) |
|  | [IPR020568](http://www.ebi.ac.uk/interpro/entry/IPR020568) | HOMOLOGOUS_SUPERFAMILY | Ribosomal protein S5 domain 2-type fold |  | [SSF54211(SUPERFAMILY)](http://www.ebi.ac.uk/interpro/signature/SSF54211) |
|  | [IPR032189](http://www.ebi.ac.uk/interpro/entry/IPR032189) | DOMAIN | DNA mismatch repair protein Mlh1, C-terminal |  | [PF16413 (PFAM)](http://www.ebi.ac.uk/interpro/signature/PF16413) |
|  | [IPR036890](http://www.ebi.ac.uk/interpro/entry/IPR036890) | HOMOLOGOUS_SUPERFAMILY | Histidine kinase/HSP90-like ATPase superfamily |  | [G3DSA:3.30.565.10(GENE3D)](http://www.ebi.ac.uk/interpro/signature/G3DSA:3.30.565.10) |
|  |  |  |  |  | [SSF55874(SUPERFAMILY)](http://www.ebi.ac.uk/interpro/signature/SSF55874) |
|  | no IPR | FAMILY | DNA mismatch repair protein family |  | mobidb-lite (MOBIDB_LITE) |
|  |  |  |  |  | [PTHR10073 (PANTHER)](http://www.ebi.ac.uk/interpro/signature/PTHR10073) |
|  |  |  |  |  | [PTHR10073 (PANTHER)](http://www.ebi.ac.uk/interpro/signature/PTHR10073) |
|  | no IPR | DOMAIN | DNA mismatch repair protein, C-terminal |  | [PF13589 (PFAM)](http://www.ebi.ac.uk/interpro/signature/PF13589) |
|  | no IPR | CONSERVED_SITE | DNA mismatch repair, conserved site |  | cd03483 (CDD) |
| *RsMLH3* | [IPR001202](http://www.ebi.ac.uk/interpro/entry/IPR001202) | DOMAIN | WW domain | GO:0005515 | [SM00456 (SMART)](http://www.ebi.ac.uk/interpro/signature/SM00456) |
|  |  |  |  |  | [PS01159(PROSITE_PATTERNS)](http://www.ebi.ac.uk/interpro/signature/PS01159) |
|  |  |  |  |  | [PS50020(PROSITE_PROFILES)](http://www.ebi.ac.uk/interpro/signature/PS50020) |
|  |  |  |  |  | cd00201 (CDD) |
|  | [IPR003594](http://www.ebi.ac.uk/interpro/entry/IPR003594) | DOMAIN | Histidine kinase/HSP90-like ATPase |  | [PF02518 (PFAM)](http://www.ebi.ac.uk/interpro/signature/PF02518) |
|  |  |  |  |  | cd00075 (CDD) |
|  | [IPR013507](http://www.ebi.ac.uk/interpro/entry/IPR013507) | DOMAIN | DNA mismatch repair protein, C-terminal | GO:0006298, GO:0005524, GO:0030983 | [SM01340 (SMART)](http://www.ebi.ac.uk/interpro/signature/SM01340) |
|  |  |  |  |  | [PF01119 (PFAM)](http://www.ebi.ac.uk/interpro/signature/PF01119) |
|  | [IPR014721](http://www.ebi.ac.uk/interpro/entry/IPR014721) | HOMOLOGOUS_SUPERFAMILY | Ribosomal protein S5 domain 2-type fold, subgroup |  | [G3DSA:3.30.230.10(GENE3D)](http://www.ebi.ac.uk/interpro/signature/G3DSA:3.30.230.10) |
|  | [IPR014762](http://www.ebi.ac.uk/interpro/entry/IPR014762) | CONSERVED_SITE | DNA mismatch repair, conserved site |  | [PS00058(PROSITE_PATTERNS)](http://www.ebi.ac.uk/interpro/signature/PS00058) |
|  | [IPR014790](http://www.ebi.ac.uk/interpro/entry/IPR014790) | DOMAIN | MutL, C-terminal, dimerisation | GO:0005524, GO:0006298 | [SM00853 (SMART)](http://www.ebi.ac.uk/interpro/signature/SM00853) |
|  |  |  |  |  | [PF08676 (PFAM)](http://www.ebi.ac.uk/interpro/signature/PF08676) |
|  | [IPR020568](http://www.ebi.ac.uk/interpro/entry/IPR020568) | HOMOLOGOUS_SUPERFAMILY | Ribosomal protein S5 domain 2-type fold |  | [SSF54211(SUPERFAMILY)](http://www.ebi.ac.uk/interpro/signature/SSF54211) |
|  | [IPR028830](http://www.ebi.ac.uk/interpro/entry/IPR028830) | FAMILY | DNA mismatch repair protein Mlh3 | GO:0007131, GO:0032300, GO:0006298 | [PTHR10073:SF7 (PANTHER)](http://www.ebi.ac.uk/interpro/signature/PTHR10073) |
|  | [IPR036020](http://www.ebi.ac.uk/interpro/entry/IPR036020) | HOMOLOGOUS_SUPERFAMILY | WW domain superfamily | GO:0005515 | [SSF51045(SUPERFAMILY)](http://www.ebi.ac.uk/interpro/signature/SSF51045) |
|  | [IPR036890](http://www.ebi.ac.uk/interpro/entry/IPR036890) | HOMOLOGOUS_SUPERFAMILY | Histidine kinase/HSP90-like ATPase superfamily |  | [G3DSA:3.30.565.10(GENE3D)](http://www.ebi.ac.uk/interpro/signature/G3DSA:3.30.565.10) |
|  |  |  |  |  | [SSF55874(SUPERFAMILY)](http://www.ebi.ac.uk/interpro/signature/SSF55874) |
|  | [IPR037198](http://www.ebi.ac.uk/interpro/entry/IPR037198) | HOMOLOGOUS_SUPERFAMILY | MutL, C-terminal domain superfamily |  | [SSF118116(SUPERFAMILY)](http://www.ebi.ac.uk/interpro/signature/SSF118116) |
|  | no IPR | DOMAIN | DNA mismatch repair protein, C-terminal |  | [G3DSA:3.30.1370.100(GENE3D)](http://www.ebi.ac.uk/interpro/signature/G3DSA:3.30.1370.100) |
|  |  |  |  |  | [G3DSA:2.30.42.20(GENE3D)](http://www.ebi.ac.uk/interpro/signature/G3DSA:2.30.42.20) |
|  | no IPR | HOMOLOGOUS_SUPERFAMILY | Histidine kinase/HSP90-like ATPase superfamily |  | mobidb-lite (MOBIDB_LITE) |
|  |  |  |  |  | mobidb-lite (MOBIDB_LITE) |
|  |  |  |  |  | [PTHR10073 (PANTHER)](http://www.ebi.ac.uk/interpro/signature/PTHR10073) |
| *RsMPG* | [IPR003180](http://www.ebi.ac.uk/interpro/entry/IPR003180) | FAMILY | Methylpurine-DNA glycosylase | GO:0003677, GO:0003905, GO:0006284 | [PF02245 (PFAM)](http://www.ebi.ac.uk/interpro/signature/PF02245) |
|  |  |  |  |  | [TIGR00567(TIGRFAM)](http://www.ebi.ac.uk/interpro/signature/TIGR00567) |
|  |  |  |  |  | [PTHR10429(PANTHER)](http://www.ebi.ac.uk/interpro/signature/PTHR10429) |
|  |  |  |  |  | MF_00527 (HAMAP) |
|  |  |  |  |  | cd00540 (CDD) |
|  | [IPR011034](http://www.ebi.ac.uk/interpro/entry/IPR011034) | HOMOLOGOUS_SUPERFAMILY | Formyl transferase-like, C-terminal domain superfamily | GO:0003824 | [SSF50486(SUPERFAMILY)](http://www.ebi.ac.uk/interpro/signature/SSF50486) |
|  | [IPR036995](http://www.ebi.ac.uk/interpro/entry/IPR036995) | HOMOLOGOUS_SUPERFAMILY | Methylpurine-DNA glycosylase superfamily | GO:0003677, GO:0006284, GO:0003905 | [G3DSA:3.10.300.10(GENE3D)](http://www.ebi.ac.uk/interpro/signature/G3DSA:3.10.300.10) |
|  | no IPR | HOMOLOGOUS_SUPERFAMILY | Methylpurine-DNA glycosylase superfamily |  | mobidb-lite (MOBIDB_LITE) |
| *RsMRE11* | [IPR003701](http://www.ebi.ac.uk/interpro/entry/IPR003701) | FAMILY | DNA double-strand break repair protein Mre11 | GO:0008408, GO:0030870, GO:0004519, GO:0006302 | [TIGR00583(TIGRFAM)](http://www.ebi.ac.uk/interpro/signature/TIGR00583) |
|  |  |  |  |  | PIRSF000882 (PIRSF) |
|  | [IPR004843](http://www.ebi.ac.uk/interpro/entry/IPR004843) | DOMAIN | Calcineurin-like phosphoesterase domain, ApaH type | GO:0016787 | [PF00149 (PFAM)](http://www.ebi.ac.uk/interpro/signature/PF00149) |
|  | [IPR007281](http://www.ebi.ac.uk/interpro/entry/IPR007281) | DOMAIN | Mre11, DNA-binding | GO:0005634, GO:0030145, GO:0004519, GO:0006302 | [SM01347 (SMART)](http://www.ebi.ac.uk/interpro/signature/SM01347) |
|  |  |  |  |  | [PF04152 (PFAM)](http://www.ebi.ac.uk/interpro/signature/PF04152) |
|  | [IPR029052](http://www.ebi.ac.uk/interpro/entry/IPR029052) | HOMOLOGOUS_SUPERFAMILY | Metallo-dependent phosphatase-like |  | [G3DSA:3.60.21.10(GENE3D)](http://www.ebi.ac.uk/interpro/signature/G3DSA:3.60.21.10) |
|  | [IPR038487](http://www.ebi.ac.uk/interpro/entry/IPR038487) | HOMOLOGOUS_SUPERFAMILY | Mre11, DNA-binding domain superfamily |  | [G3DSA:3.30.110.110(GENE3D)](http://www.ebi.ac.uk/interpro/signature/G3DSA:3.30.110.110) |
|  | no IPR | FAMILY | DNA double-strand break repair protein Mre11 |  | mobidb-lite (MOBIDB_LITE) |
|  |  |  |  |  | [PTHR10139(PANTHER)](http://www.ebi.ac.uk/interpro/signature/PTHR10139) |
|  |  |  |  |  | cd00840 (CDD) |
|  |  |  |  |  | [SSF56300(SUPERFAMILY)](http://www.ebi.ac.uk/interpro/signature/SSF56300) |
|  | no IPR |  |  |  | Coil (COILS) |
| *RsMSH3* | [IPR000432](http://www.ebi.ac.uk/interpro/entry/IPR000432) | DOMAIN | DNA mismatch repair protein MutS, C-terminal | GO:0030983, GO:0006298, GO:0005524 | [SM00534 (SMART)](http://www.ebi.ac.uk/interpro/signature/SM00534) |
|  |  |  |  |  | [PF00488 (PFAM)](http://www.ebi.ac.uk/interpro/signature/PF00488) |
|  |  |  |  |  | [PS00486(PROSITE_PATTERNS)](http://www.ebi.ac.uk/interpro/signature/PS00486) |
|  | [IPR007695](http://www.ebi.ac.uk/interpro/entry/IPR007695) | DOMAIN | DNA mismatch repair protein MutS-like, N-terminal | GO:0006298, GO:0005524, GO:0030983 | [PF01624 (PFAM)](http://www.ebi.ac.uk/interpro/signature/PF01624) |
|  | [IPR007696](http://www.ebi.ac.uk/interpro/entry/IPR007696) | DOMAIN | DNA mismatch repair protein MutS, core | GO:0030983, GO:0005524, GO:0006298 | [SM00533 (SMART)](http://www.ebi.ac.uk/interpro/signature/SM00533) |
|  |  |  |  |  | [PF05192 (PFAM)](http://www.ebi.ac.uk/interpro/signature/PF05192) |
|  | [IPR007860](http://www.ebi.ac.uk/interpro/entry/IPR007860) | DOMAIN | DNA mismatch repair protein MutS, connector domain | GO:0006298, GO:0030983, GO:0005524 | [PF05188 (PFAM)](http://www.ebi.ac.uk/interpro/signature/PF05188) |
|  | [IPR016151](http://www.ebi.ac.uk/interpro/entry/IPR016151) | HOMOLOGOUS_SUPERFAMILY | DNA mismatch repair protein MutS, N-terminal | GO:0006298, GO:0005524, GO:0030983 | [G3DSA:3.40.1170.10(GENE3D)](http://www.ebi.ac.uk/interpro/signature/G3DSA:3.40.1170.10) |
|  |  |  |  |  | [SSF55271(SUPERFAMILY)](http://www.ebi.ac.uk/interpro/signature/SSF55271) |
|  | [IPR017261](http://www.ebi.ac.uk/interpro/entry/IPR017261) | FAMILY | DNA mismatch repair protein MutS/MSH | GO:0006298, GO:0030983 | PIRSF037677 (PIRSF) |
|  | [IPR027417](http://www.ebi.ac.uk/interpro/entry/IPR027417) | HOMOLOGOUS_SUPERFAMILY | P-loop containing nucleoside triphosphate hydrolase |  | [SSF52540(SUPERFAMILY)](http://www.ebi.ac.uk/interpro/signature/SSF52540) |
|  | [IPR036187](http://www.ebi.ac.uk/interpro/entry/IPR036187) | HOMOLOGOUS_SUPERFAMILY | DNA mismatch repair protein MutS, core domain superfamily |  | [SSF48334(SUPERFAMILY)](http://www.ebi.ac.uk/interpro/signature/SSF48334) |
|  | [IPR036678](http://www.ebi.ac.uk/interpro/entry/IPR036678) | HOMOLOGOUS_SUPERFAMILY | MutS, connector domain superfamily | GO:0005524, GO:0030983, GO:0006298 | [G3DSA:3.30.420.110(GENE3D)](http://www.ebi.ac.uk/interpro/signature/G3DSA:3.30.420.110) |
|  |  |  |  |  | [SSF53150(SUPERFAMILY)](http://www.ebi.ac.uk/interpro/signature/SSF53150) |
|  | no IPR | HOMOLOGOUS_SUPERFAMILY | DNA mismatch repair protein MutS, N-terminal |  | [G3DSA:1.10.1420.10(GENE3D)](http://www.ebi.ac.uk/interpro/signature/G3DSA:1.10.1420.10) |
|  | no IPR | DOMAIN | DNA mismatch repair protein MutS-like, N-terminal |  | mobidb-lite (MOBIDB_LITE) |
|  |  |  |  |  | [PTHR11361:SF122 (PANTHER)](http://www.ebi.ac.uk/interpro/signature/PTHR11361) |
|  |  |  |  |  | [PTHR11361(PANTHER)](http://www.ebi.ac.uk/interpro/signature/PTHR11361) |
|  | no IPR | FAMILY | DNA mismatch repair protein MutS/MSH |  | [G3DSA:1.10.1420.10(GENE3D)](http://www.ebi.ac.uk/interpro/signature/G3DSA:1.10.1420.10) |
|  |  |  |  |  | [G3DSA:3.40.50.300(GENE3D)](http://www.ebi.ac.uk/interpro/signature/G3DSA:3.40.50.300) |
| *RsMSH6* | [IPR000432](http://www.ebi.ac.uk/interpro/entry/IPR000432) | DOMAIN | DNA mismatch repair protein MutS, C-terminal | GO:0030983, GO:0006298, GO:0005524 | [SM00534 (SMART)](http://www.ebi.ac.uk/interpro/signature/SM00534) |
|  |  |  |  |  | [PF00488 (PFAM)](http://www.ebi.ac.uk/interpro/signature/PF00488) |
|  |  |  |  |  | [PS00486(PROSITE_PATTERNS)](http://www.ebi.ac.uk/interpro/signature/PS00486) |
|  | [IPR007695](http://www.ebi.ac.uk/interpro/entry/IPR007695) | DOMAIN | DNA mismatch repair protein MutS-like, N-terminal | GO:0005524, GO:0030983, GO:0006298 | [PF01624 (PFAM)](http://www.ebi.ac.uk/interpro/signature/PF01624) |
|  | [IPR007696](http://www.ebi.ac.uk/interpro/entry/IPR007696) | DOMAIN | DNA mismatch repair protein MutS, core | GO:0030983, GO:0005524, GO:0006298 | [SM00533 (SMART)](http://www.ebi.ac.uk/interpro/signature/SM00533) |
|  |  |  |  |  | [PF05192 (PFAM)](http://www.ebi.ac.uk/interpro/signature/PF05192) |
|  | [IPR007860](http://www.ebi.ac.uk/interpro/entry/IPR007860) | DOMAIN | DNA mismatch repair protein MutS, connector domain | GO:0006298, GO:0030983, GO:0005524 | [PF05188 (PFAM)](http://www.ebi.ac.uk/interpro/signature/PF05188) |
|  | [IPR007861](http://www.ebi.ac.uk/interpro/entry/IPR007861) | DOMAIN | DNA mismatch repair protein MutS, clamp | GO:0005524, GO:0030983, GO:0006298 | [PF05190 (PFAM)](http://www.ebi.ac.uk/interpro/signature/PF05190) |
|  | [IPR015536](http://www.ebi.ac.uk/interpro/entry/IPR015536) | FAMILY | DNA mismatch repair protein MutS-homologue MSH6 | GO:0006298 | [PTHR11361:SF31 (PANTHER)](http://www.ebi.ac.uk/interpro/signature/PTHR11361) |
|  | [IPR016151](http://www.ebi.ac.uk/interpro/entry/IPR016151) | HOMOLOGOUS_SUPERFAMILY | DNA mismatch repair protein MutS, N-terminal | GO:0030983, GO:0005524, GO:0006298 | [G3DSA:3.40.1170.10(GENE3D)](http://www.ebi.ac.uk/interpro/signature/G3DSA:3.40.1170.10) |
|  |  |  |  |  | [SSF55271(SUPERFAMILY)](http://www.ebi.ac.uk/interpro/signature/SSF55271) |
|  | [IPR017261](http://www.ebi.ac.uk/interpro/entry/IPR017261) | FAMILY | DNA mismatch repair protein MutS/MSH | GO:0030983, GO:0006298 | PIRSF037677 (PIRSF) |
|  | [IPR027417](http://www.ebi.ac.uk/interpro/entry/IPR027417) | HOMOLOGOUS_SUPERFAMILY | P-loop containing nucleoside triphosphate hydrolase |  | [SSF52540(SUPERFAMILY)](http://www.ebi.ac.uk/interpro/signature/SSF52540) |
|  | [IPR036187](http://www.ebi.ac.uk/interpro/entry/IPR036187) | HOMOLOGOUS_SUPERFAMILY | DNA mismatch repair protein MutS, core domain superfamily |  | [SSF48334(SUPERFAMILY)](http://www.ebi.ac.uk/interpro/signature/SSF48334) |
|  | [IPR036678](http://www.ebi.ac.uk/interpro/entry/IPR036678) | HOMOLOGOUS_SUPERFAMILY | MutS, connector domain superfamily | GO:0006298, GO:0030983, GO:0005524 | [G3DSA:3.30.420.110(GENE3D)](http://www.ebi.ac.uk/interpro/signature/G3DSA:3.30.420.110) |
|  |  |  |  |  | [SSF53150(SUPERFAMILY)](http://www.ebi.ac.uk/interpro/signature/SSF53150) |
|  | no IPR | DOMAIN | DNA mismatch repair protein MutS, C-terminal |  | [G3DSA:1.10.1420.10(GENE3D)](http://www.ebi.ac.uk/interpro/signature/G3DSA:1.10.1420.10) |
|  |  |  |  |  | cd03286 (CDD) |
|  | no IPR | HOMOLOGOUS_SUPERFAMILY | DNA mismatch repair protein MutS, N-terminal |  | [G3DSA:1.10.1420.10(GENE3D)](http://www.ebi.ac.uk/interpro/signature/G3DSA:1.10.1420.10) |
|  | no IPR | FAMILY | DNA mismatch repair protein MutS-homologue MSH6 |  | [PTHR11361(PANTHER)](http://www.ebi.ac.uk/interpro/signature/PTHR11361) |
|  | no IPR | DOMAIN | DNA mismatch repair protein MutS-like, N-terminal |  | [G3DSA:3.40.50.300(GENE3D)](http://www.ebi.ac.uk/interpro/signature/G3DSA:3.40.50.300) |
|  | no IPR |  |  |  | Coil (COILS) |
| *RsMUS81* | [IPR027421](http://www.ebi.ac.uk/interpro/entry/IPR027421) | HOMOLOGOUS_SUPERFAMILY | DNA polymerase lambda lyase domain superfamily |  | [G3DSA:1.10.150.110 (GENE3D)](http://www.ebi.ac.uk/interpro/signature/G3DSA:1.10.150.110) |
|  |  |  |  |  | [SSF47802 (SUPERFAMILY)](http://www.ebi.ac.uk/interpro/signature/SSF47802) |
|  | [IPR036388](http://www.ebi.ac.uk/interpro/entry/IPR036388) | HOMOLOGOUS_SUPERFAMILY | Winged helix-like DNA-binding domain superfamily |  | [G3DSA:1.10.10.10 (GENE3D)](http://www.ebi.ac.uk/interpro/signature/G3DSA:1.10.10.10) |
|  | no IPR | HOMOLOGOUS_SUPERFAMILY | DNA polymerase lambda lyase domain superfamily |  | mobidb-lite (MOBIDB_LITE) |
|  |  |  |  |  | [PTHR13451 (PANTHER)](http://www.ebi.ac.uk/interpro/signature/PTHR13451) |
|  |  |  |  |  | [PTHR13451:SF0 (PANTHER)](http://www.ebi.ac.uk/interpro/signature/PTHR13451) |
| *RsMutS4* | [IPR000432](http://www.ebi.ac.uk/interpro/entry/IPR000432) | DOMAIN | DNA mismatch repair protein MutS, C-terminal | GO:0005524, GO:0006298, GO:0030983 | [SM00534 (SMART)](http://www.ebi.ac.uk/interpro/signature/SM00534) |
|  |  |  |  |  | [PF00488 (PFAM)](http://www.ebi.ac.uk/interpro/signature/PF00488) |
|  |  |  |  |  | [PS00486(PROSITE_PATTERNS)](http://www.ebi.ac.uk/interpro/signature/PS00486) |
|  | [IPR007696](http://www.ebi.ac.uk/interpro/entry/IPR007696) | DOMAIN | DNA mismatch repair protein MutS, core | GO:0005524, GO:0030983, GO:0006298 | [SM00533 (SMART)](http://www.ebi.ac.uk/interpro/signature/SM00533) |
|  |  |  |  |  | [PF05192 (PFAM)](http://www.ebi.ac.uk/interpro/signature/PF05192) |
|  | [IPR007860](http://www.ebi.ac.uk/interpro/entry/IPR007860) | DOMAIN | DNA mismatch repair protein MutS, connector domain | GO:0005524, GO:0006298, GO:0030983 | [PF05188 (PFAM)](http://www.ebi.ac.uk/interpro/signature/PF05188) |
|  | [IPR007861](http://www.ebi.ac.uk/interpro/entry/IPR007861) | DOMAIN | DNA mismatch repair protein MutS, clamp | GO:0005524, GO:0030983, GO:0006298 | [PF05190 (PFAM)](http://www.ebi.ac.uk/interpro/signature/PF05190) |
|  | [IPR017261](http://www.ebi.ac.uk/interpro/entry/IPR017261) | FAMILY | DNA mismatch repair protein MutS/MSH | GO:0030983, GO:0006298 | PIRSF037677 (PIRSF) |
|  | [IPR027417](http://www.ebi.ac.uk/interpro/entry/IPR027417) | HOMOLOGOUS_SUPERFAMILY | P-loop containing nucleoside triphosphate hydrolase |  | [SSF52540(SUPERFAMILY)](http://www.ebi.ac.uk/interpro/signature/SSF52540) |
|  | [IPR036187](http://www.ebi.ac.uk/interpro/entry/IPR036187) | HOMOLOGOUS_SUPERFAMILY | DNA mismatch repair protein MutS, core domain superfamily |  | [SSF48334(SUPERFAMILY)](http://www.ebi.ac.uk/interpro/signature/SSF48334) |
|  | [IPR036678](http://www.ebi.ac.uk/interpro/entry/IPR036678) | HOMOLOGOUS_SUPERFAMILY | MutS, connector domain superfamily | GO:0030983, GO:0005524, GO:0006298 | [G3DSA:3.30.420.110(GENE3D)](http://www.ebi.ac.uk/interpro/signature/G3DSA:3.30.420.110) |
|  |  |  |  |  | [SSF53150(SUPERFAMILY)](http://www.ebi.ac.uk/interpro/signature/SSF53150) |
|  | no IPR | DOMAIN | DNA mismatch repair protein MutS, C-terminal |  | [G3DSA:3.40.50.300(GENE3D)](http://www.ebi.ac.uk/interpro/signature/G3DSA:3.40.50.300) |
|  |  |  |  |  | [G3DSA:1.10.1420.10(GENE3D)](http://www.ebi.ac.uk/interpro/signature/G3DSA:1.10.1420.10) |
|  |  |  |  |  | mobidb-lite (MOBIDB_LITE) |
|  |  |  |  |  | [PTHR11361:SF21 (PANTHER)](http://www.ebi.ac.uk/interpro/signature/PTHR11361) |
|  |  |  |  |  | [PTHR11361(PANTHER)](http://www.ebi.ac.uk/interpro/signature/PTHR11361) |
|  |  |  |  |  | cd03243 (CDD) |
|  | no IPR | FAMILY | DNA mismatch repair protein MutS/MSH |  | [G3DSA:1.10.1420.10(GENE3D)](http://www.ebi.ac.uk/interpro/signature/G3DSA:1.10.1420.10) |
| *RsNBS1* | [IPR000253](http://www.ebi.ac.uk/interpro/entry/IPR000253) | DOMAIN | Forkhead-associated (FHA) domain | GO:0005515 | [SM00240 (SMART)](http://www.ebi.ac.uk/interpro/signature/SM00240) |
|  |  |  |  |  | [PF00498 (PFAM)](http://www.ebi.ac.uk/interpro/signature/PF00498) |
|  |  |  |  |  | [PS50006 (PROSITE_PROFILES)](http://www.ebi.ac.uk/interpro/signature/PS50006) |
|  |  |  |  |  | cd00060 (CDD) |
|  | [IPR001357](http://www.ebi.ac.uk/interpro/entry/IPR001357) | DOMAIN | BRCT domain |  | cd00027 (CDD) |
|  | [IPR008984](http://www.ebi.ac.uk/interpro/entry/IPR008984) | HOMOLOGOUS_SUPERFAMILY | SMAD/FHA domain superfamily | GO:0005515 | [SSF49879 (SUPERFAMILY)](http://www.ebi.ac.uk/interpro/signature/SSF49879) |
|  | [IPR032429](http://www.ebi.ac.uk/interpro/entry/IPR032429) | DOMAIN | Nibrin, second BRCT domain |  | [PF16508 (PFAM)](http://www.ebi.ac.uk/interpro/signature/PF16508) |
|  | [IPR036420](http://www.ebi.ac.uk/interpro/entry/IPR036420) | HOMOLOGOUS_SUPERFAMILY | BRCT domain superfamily |  | [G3DSA:3.40.50.10190 (GENE3D)](http://www.ebi.ac.uk/interpro/signature/G3DSA:3.40.50.1019) |
|  |  |  |  |  | [SSF52113 (SUPERFAMILY)](http://www.ebi.ac.uk/interpro/signature/SSF52113) |
|  | no IPR | DOMAIN | Forkhead-associated (FHA) domain |  | [G3DSA:3.40.50.10980 (GENE3D)](http://www.ebi.ac.uk/interpro/signature/G3DSA:3.40.50.1098) |
|  | no IPR | DOMAIN | Nibrin, second BRCT domain |  | [G3DSA:2.60.200.20 (GENE3D)](http://www.ebi.ac.uk/interpro/signature/G3DSA:2.60.200.20) |
|  |  |  |  |  | mobidb-lite (MOBIDB_LITE) |
|  |  |  |  |  | [PTHR12162 (PANTHER)](http://www.ebi.ac.uk/interpro/signature/PTHR12162) |
|  | no IPR |  |  |  | Coil (COILS) |
| *RsNHEJ1* | [IPR015381](http://www.ebi.ac.uk/interpro/entry/IPR015381) | FAMILY | XLF family | GO:0005634, GO:0006302 | [PF09302 (PFAM)](http://www.ebi.ac.uk/interpro/signature/PF09302) |
|  | no IPR | FAMILY | XLF family |  | mobidb-lite (MOBIDB_LITE) |
|  |  |  |  |  | [PTHR32235 (PANTHER)](http://www.ebi.ac.uk/interpro/signature/PTHR32235) |
|  | no IPR |  |  |  | Coil (COILS) |
|  |  |  |  |  | [G3DSA:2.170.210.10 (GENE3D)](http://www.ebi.ac.uk/interpro/signature/G3DSA:2.170.210.10) |
|  |  |  |  |  | [G3DSA:1.10.287.450 (GENE3D)](http://www.ebi.ac.uk/interpro/signature/G3DSA:1.10.287.450) |
| *RsNTHL1* | [IPR000445](http://www.ebi.ac.uk/interpro/entry/IPR000445) | CONSERVED_SITE | Helix-hairpin-helix motif | GO:0003677 | [PF00633 (PFAM)](http://www.ebi.ac.uk/interpro/signature/PF00633) |
|  | [IPR003265](http://www.ebi.ac.uk/interpro/entry/IPR003265) | DOMAIN | HhH-GPD domain | GO:0006284 | [SM00478 (SMART)](http://www.ebi.ac.uk/interpro/signature/SM00478) |
|  |  |  |  |  | [PF00730 (PFAM)](http://www.ebi.ac.uk/interpro/signature/PF00730) |
|  |  |  |  |  | cd00056 (CDD) |
|  | [IPR004035](http://www.ebi.ac.uk/interpro/entry/IPR004035) | BINDING_SITE | Endonuclease III, iron-sulphur binding site |  | [PS00764(PROSITE_PATTERNS)](http://www.ebi.ac.uk/interpro/signature/PS00764) |
|  | [IPR004036](http://www.ebi.ac.uk/interpro/entry/IPR004036) | CONSERVED_SITE | Endonuclease III-like, conserved site-2 |  | [PS01155(PROSITE_PATTERNS)](http://www.ebi.ac.uk/interpro/signature/PS01155) |
|  | [IPR011257](http://www.ebi.ac.uk/interpro/entry/IPR011257) | HOMOLOGOUS_SUPERFAMILY | DNA glycosylase | GO:0003824, GO:0006281 | [SSF48150(SUPERFAMILY)](http://www.ebi.ac.uk/interpro/signature/SSF48150) |
|  | [IPR023170](http://www.ebi.ac.uk/interpro/entry/IPR023170) | HOMOLOGOUS_SUPERFAMILY | Helix-turn-helix, base-excision DNA repair, C-terminal |  | [G3DSA:1.10.1670.10(GENE3D)](http://www.ebi.ac.uk/interpro/signature/G3DSA:1.10.1670.10) |
|  | [IPR030841](http://www.ebi.ac.uk/interpro/entry/IPR030841) | FAMILY | Endonuclease III-like protein 1 | GO:0019104, GO:0006285, GO:0006281, GO:0005634, GO:0003906 | MF_03183 (HAMAP) |
|  | no IPR | CONSERVED_SITE | Helix-hairpin-helix motif |  | [G3DSA:1.10.340.30(GENE3D)](http://www.ebi.ac.uk/interpro/signature/G3DSA:1.10.340.30) |
|  | no IPR | HOMOLOGOUS_SUPERFAMILY | Helix-turn-helix, base-excision DNA repair, C-terminal |  | mobidb-lite (MOBIDB_LITE) |
|  |  |  |  |  | [PTHR43286(PANTHER)](http://www.ebi.ac.uk/interpro/signature/PTHR43286) |
| *RsPARP1* | [IPR001357](http://www.ebi.ac.uk/interpro/entry/IPR001357) | DOMAIN | BRCT domain |  | [SM00292 (SMART)](http://www.ebi.ac.uk/interpro/signature/SM00292) |
|  |  |  |  |  | [PF00533 (PFAM)](http://www.ebi.ac.uk/interpro/signature/PF00533) |
|  |  |  |  |  | [PS50172(PROSITE_PROFILES)](http://www.ebi.ac.uk/interpro/signature/PS50172) |
|  |  |  |  |  | cd00027 (CDD) |
|  | [IPR001510](http://www.ebi.ac.uk/interpro/entry/IPR001510) | DOMAIN | Zinc finger, PARP-type | GO:0008270, GO:0003677 | [SM01336 (SMART)](http://www.ebi.ac.uk/interpro/signature/SM01336) |
|  |  |  |  |  | [PF00645 (PFAM)](http://www.ebi.ac.uk/interpro/signature/PF00645) |
|  |  |  |  |  | [PS00347(PROSITE_PATTERNS)](http://www.ebi.ac.uk/interpro/signature/PS00347) |
|  |  |  |  |  | [PS50064(PROSITE_PROFILES)](http://www.ebi.ac.uk/interpro/signature/PS50064) |
|  |  |  |  |  | [PS50064(PROSITE_PROFILES)](http://www.ebi.ac.uk/interpro/signature/PS50064) |
|  | [IPR004102](http://www.ebi.ac.uk/interpro/entry/IPR004102) | DOMAIN | Poly(ADP-ribose) polymerase, regulatory domain | GO:0003950, GO:0006471 | [PF02877 (PFAM)](http://www.ebi.ac.uk/interpro/signature/PF02877) |
|  |  |  |  |  | [PS51060(PROSITE_PROFILES)](http://www.ebi.ac.uk/interpro/signature/PS51060) |
|  | [IPR008288](http://www.ebi.ac.uk/interpro/entry/IPR008288) | FAMILY | Poly [ADP-ribose] polymerase | GO:0003677, GO:0008270, GO:0005634, GO:0051287, GO:0006471, GO:0003950 | PIRSF000489 (PIRSF) |
|  | [IPR008893](http://www.ebi.ac.uk/interpro/entry/IPR008893) | DOMAIN | WGR domain |  | [SM00773 (SMART)](http://www.ebi.ac.uk/interpro/signature/SM00773) |
|  |  |  |  |  | [PF05406 (PFAM)](http://www.ebi.ac.uk/interpro/signature/PF05406) |
|  | [IPR012317](http://www.ebi.ac.uk/interpro/entry/IPR012317) | DOMAIN | Poly(ADP-ribose) polymerase, catalytic domain | GO:0003950 | [PF00644 (PFAM)](http://www.ebi.ac.uk/interpro/signature/PF00644) |
|  |  |  |  |  | [PS51059(PROSITE_PROFILES)](http://www.ebi.ac.uk/interpro/signature/PS51059) |
|  | [IPR012982](http://www.ebi.ac.uk/interpro/entry/IPR012982) | DOMAIN | PADR1 domain |  | [SM01335 (SMART)](http://www.ebi.ac.uk/interpro/signature/SM01335) |
|  |  |  |  |  | [PF08063 (PFAM)](http://www.ebi.ac.uk/interpro/signature/PF08063) |
|  | [IPR036420](http://www.ebi.ac.uk/interpro/entry/IPR036420) | HOMOLOGOUS_SUPERFAMILY | BRCT domain superfamily |  | [G3DSA:3.40.50.10190 (GENE3D)](http://www.ebi.ac.uk/interpro/signature/G3DSA:3.40.50.1019) |
|  |  |  |  |  | [SSF52113(SUPERFAMILY)](http://www.ebi.ac.uk/interpro/signature/SSF52113) |
|  | [IPR036616](http://www.ebi.ac.uk/interpro/entry/IPR036616) | HOMOLOGOUS_SUPERFAMILY | Poly(ADP-ribose) polymerase, regulatory domain superfamily | GO:0003950, GO:0006471 | [G3DSA:1.20.142.10(GENE3D)](http://www.ebi.ac.uk/interpro/signature/G3DSA:1.20.142.10) |
|  |  |  |  |  | [SSF47587(SUPERFAMILY)](http://www.ebi.ac.uk/interpro/signature/SSF47587) |
|  | [IPR036930](http://www.ebi.ac.uk/interpro/entry/IPR036930) | HOMOLOGOUS_SUPERFAMILY | WGR domain superfamily |  | [G3DSA:2.20.140.10(GENE3D)](http://www.ebi.ac.uk/interpro/signature/G3DSA:2.20.140.10) |
|  |  |  |  |  | [SSF142921(SUPERFAMILY)](http://www.ebi.ac.uk/interpro/signature/SSF142921) |
|  | [IPR036957](http://www.ebi.ac.uk/interpro/entry/IPR036957) | HOMOLOGOUS_SUPERFAMILY | Zinc finger, PARP-type superfamily | GO:0008270, GO:0003677 | [G3DSA:3.30.1740.10(GENE3D)](http://www.ebi.ac.uk/interpro/signature/G3DSA:3.30.1740.10) |
|  |  |  |  |  | [G3DSA:3.30.1740.10(GENE3D)](http://www.ebi.ac.uk/interpro/signature/G3DSA:3.30.1740.10) |
|  | [IPR038650](http://www.ebi.ac.uk/interpro/entry/IPR038650) | HOMOLOGOUS_SUPERFAMILY | PADR1 domain superfamily |  | [G3DSA:2.20.25.630(GENE3D)](http://www.ebi.ac.uk/interpro/signature/G3DSA:2.20.25.630) |
|  | no IPR | DOMAIN | BRCT domain |  | cd08001 (CDD) |
|  | no IPR | DOMAIN | PADR1 domain |  | [G3DSA:3.90.228.10(GENE3D)](http://www.ebi.ac.uk/interpro/signature/G3DSA:3.90.228.10) |
|  | no IPR | DOMAIN | Poly(ADP-ribose) polymerase, catalytic domain |  | [G3DSA:1.10.20.130(GENE3D)](http://www.ebi.ac.uk/interpro/signature/G3DSA:1.10.20.130) |
|  |  |  |  |  | cd01437 (CDD) |
|  | no IPR | DOMAIN | Poly(ADP-ribose) polymerase, regulatory domain |  | mobidb-lite (MOBIDB_LITE) |
|  |  |  |  |  | mobidb-lite (MOBIDB_LITE) |
|  |  |  |  |  | mobidb-lite (MOBIDB_LITE) |
|  |  |  |  |  | mobidb-lite (MOBIDB_LITE) |
|  |  |  |  |  | [PTHR10459(PANTHER)](http://www.ebi.ac.uk/interpro/signature/PTHR10459) |
|  |  |  |  |  | [PTHR10459:SF80 (PANTHER)](http://www.ebi.ac.uk/interpro/signature/PTHR10459) |
|  | no IPR | HOMOLOGOUS_SUPERFAMILY | Poly(ADP-ribose) polymerase, regulatory domain superfamily |  | [SSF57716(SUPERFAMILY)](http://www.ebi.ac.uk/interpro/signature/SSF57716) |
|  | no IPR | HOMOLOGOUS_SUPERFAMILY | WGR domain superfamily |  | [SSF57716(SUPERFAMILY)](http://www.ebi.ac.uk/interpro/signature/SSF57716) |
|  |  |  |  |  | [SSF56399(SUPERFAMILY)](http://www.ebi.ac.uk/interpro/signature/SSF56399) |
| *RsPCNA* | [IPR000730](http://www.ebi.ac.uk/interpro/entry/IPR000730) | FAMILY | Proliferating cell nuclear antigen, PCNA | GO:0006275, GO:0030337, GO:0003677 | [PR00339 (PRINTS)](http://www.ebi.ac.uk/interpro/signature/PR00339) |
|  |  |  |  |  | [TIGR00590 (TIGRFAM)](http://www.ebi.ac.uk/interpro/signature/TIGR00590) |
|  |  |  |  |  | [PTHR11352:SF0 (PANTHER)](http://www.ebi.ac.uk/interpro/signature/PTHR11352) |
|  |  |  |  |  | MF_00317 (HAMAP) |
|  | [IPR022648](http://www.ebi.ac.uk/interpro/entry/IPR022648) | DOMAIN | Proliferating cell nuclear antigen, PCNA, N-terminal | GO:0006275, GO:0003677 | [PF00705 (PFAM)](http://www.ebi.ac.uk/interpro/signature/PF00705) |
|  | [IPR022649](http://www.ebi.ac.uk/interpro/entry/IPR022649) | DOMAIN | Proliferating cell nuclear antigen, PCNA, C-terminal | GO:0003677, GO:0006275 | [PF02747 (PFAM)](http://www.ebi.ac.uk/interpro/signature/PF02747) |
|  | [IPR022659](http://www.ebi.ac.uk/interpro/entry/IPR022659) | CONSERVED_SITE | Proliferating cell nuclear antigen, PCNA, conserved site |  | [PS01251(PROSITE_PATTERNS)](http://www.ebi.ac.uk/interpro/signature/PS01251) |
|  |  |  |  |  | [PS00293(PROSITE_PATTERNS)](http://www.ebi.ac.uk/interpro/signature/PS00293) |
|  | no IPR | FAMILY | Proliferating cell nuclear antigen, PCNA |  | [PTHR11352 (PANTHER)](http://www.ebi.ac.uk/interpro/signature/PTHR11352) |
|  |  |  |  |  | cd00577 (CDD) |
|  |  |  |  |  | [SSF55979 (SUPERFAMILY)](http://www.ebi.ac.uk/interpro/signature/SSF55979) |
|  |  |  |  |  | [SSF55979 (SUPERFAMILY)](http://www.ebi.ac.uk/interpro/signature/SSF55979) |
|  | no IPR | DOMAIN | Proliferating cell nuclear antigen, PCNA, N-terminal |  | [G3DSA:3.70.10.10(GENE3D)](http://www.ebi.ac.uk/interpro/signature/G3DSA:3.70.10.10) |
| *RsPIAS1* | [IPR004181](http://www.ebi.ac.uk/interpro/entry/IPR004181) | DOMAIN | Zinc finger, MIZ-type | GO:0008270 | [PF02891 (PFAM)](http://www.ebi.ac.uk/interpro/signature/PF02891) |
|  |  |  |  |  | [PS51044 (PROSITE_PROFILES)](http://www.ebi.ac.uk/interpro/signature/PS51044) |
|  | [IPR013083](http://www.ebi.ac.uk/interpro/entry/IPR013083) | HOMOLOGOUS_SUPERFAMILY | Zinc finger, RING/FYVE/PHD-type |  | [G3DSA:3.30.40.10 (GENE3D)](http://www.ebi.ac.uk/interpro/signature/G3DSA:3.30.40.10) |
|  | [IPR023321](http://www.ebi.ac.uk/interpro/entry/IPR023321) | DOMAIN | PINIT domain |  | [PF14324 (PFAM)](http://www.ebi.ac.uk/interpro/signature/PF14324) |
|  |  |  |  |  | [PS51466 (PROSITE_PROFILES)](http://www.ebi.ac.uk/interpro/signature/PS51466) |
|  | [IPR027228](http://www.ebi.ac.uk/interpro/entry/IPR027228) | FAMILY | E3 SUMO-protein ligase PIAS2 | GO:0019789 | [PTHR10782:SF12 (PANTHER)](http://www.ebi.ac.uk/interpro/signature/PTHR10782) |
|  |  |  |  |  | [PTHR10782:SF12 (PANTHER)](http://www.ebi.ac.uk/interpro/signature/PTHR10782) |
|  | [IPR038654](http://www.ebi.ac.uk/interpro/entry/IPR038654) | HOMOLOGOUS_SUPERFAMILY | PINIT domain superfamily |  | [G3DSA:2.60.120.780 (GENE3D)](http://www.ebi.ac.uk/interpro/signature/G3DSA:2.60.120.780) |
|  | no IPR | FAMILY | E3 SUMO-protein ligase PIAS2 |  | [PTHR10782 (PANTHER)](http://www.ebi.ac.uk/interpro/signature/PTHR10782) |
|  |  |  |  |  | [PTHR10782 (PANTHER)](http://www.ebi.ac.uk/interpro/signature/PTHR10782) |
|  | no IPR | DOMAIN | Zinc finger, MIZ-type |  | cd16790 (CDD) |
|  | no IPR | HOMOLOGOUS_SUPERFAMILY | Zinc finger, RING/FYVE/PHD-type |  | mobidb-lite (MOBIDB_LITE) |
| *RsPMS1* | [IPR003594](http://www.ebi.ac.uk/interpro/entry/IPR003594) | DOMAIN | Histidine kinase/HSP90-like ATPase |  | cd00075 (CDD) |
|  | [IPR014762](http://www.ebi.ac.uk/interpro/entry/IPR014762) | CONSERVED_SITE | DNA mismatch repair, conserved site |  | [PS00058 (PROSITE_PATTERNS)](http://www.ebi.ac.uk/interpro/signature/PS00058) |
|  | [IPR031263](http://www.ebi.ac.uk/interpro/entry/IPR031263) | FAMILY | PMS1 protein homologue 1 |  | [PTHR10073:SF43 (PANTHER)](http://www.ebi.ac.uk/interpro/signature/PTHR10073) |
|  | [IPR036890](http://www.ebi.ac.uk/interpro/entry/IPR036890) | HOMOLOGOUS_SUPERFAMILY | Histidine kinase/HSP90-like ATPase superfamily |  | [G3DSA:3.30.565.10 (GENE3D)](http://www.ebi.ac.uk/interpro/signature/G3DSA:3.30.565.10) |
|  |  |  |  |  | [SSF55874 (SUPERFAMILY)](http://www.ebi.ac.uk/interpro/signature/SSF55874) |
|  | no IPR | FAMILY | PMS1 protein homologue 1 |  | [PTHR10073 (PANTHER)](http://www.ebi.ac.uk/interpro/signature/PTHR10073) |
|  | no IPR |  |  |  | [PF13589 (PFAM)](http://www.ebi.ac.uk/interpro/signature/PF13589) |
| *RsPMS2* | [IPR002099](http://www.ebi.ac.uk/interpro/entry/IPR002099) | FAMILY | DNA mismatch repair protein family | GO:0006298, GO:0030983, GO:0005524 | [TIGR00585 (TIGRFAM)](http://www.ebi.ac.uk/interpro/signature/TIGR00585) |
|  | [IPR003594](http://www.ebi.ac.uk/interpro/entry/IPR003594) | DOMAIN | Histidine kinase/HSP90-like ATPase |  | cd00075 (CDD) |
|  | [IPR013507](http://www.ebi.ac.uk/interpro/entry/IPR013507) | DOMAIN | DNA mismatch repair protein, C-terminal | GO:0005524, GO:0006298, GO:0030983 | [SM01340 (SMART)](http://www.ebi.ac.uk/interpro/signature/SM01340) |
|  |  |  |  |  | [PF01119 (PFAM)](http://www.ebi.ac.uk/interpro/signature/PF01119) |
|  | [IPR014721](http://www.ebi.ac.uk/interpro/entry/IPR014721) | HOMOLOGOUS_SUPERFAMILY | Ribosomal protein S5 domain 2-type fold, subgroup |  | [G3DSA:3.30.230.10(GENE3D)](http://www.ebi.ac.uk/interpro/signature/G3DSA:3.30.230.10) |
|  | [IPR014762](http://www.ebi.ac.uk/interpro/entry/IPR014762) | CONSERVED_SITE | DNA mismatch repair, conserved site |  | [PS00058(PROSITE_PATTERNS)](http://www.ebi.ac.uk/interpro/signature/PS00058) |
|  | [IPR014790](http://www.ebi.ac.uk/interpro/entry/IPR014790) | DOMAIN | MutL, C-terminal, dimerisation | GO:0006298, GO:0005524 | [SM00853 (SMART)](http://www.ebi.ac.uk/interpro/signature/SM00853) |
|  |  |  |  |  | [PF08676 (PFAM)](http://www.ebi.ac.uk/interpro/signature/PF08676) |
|  | [IPR020568](http://www.ebi.ac.uk/interpro/entry/IPR020568) | HOMOLOGOUS_SUPERFAMILY | Ribosomal protein S5 domain 2-type fold |  | [SSF54211(SUPERFAMILY)](http://www.ebi.ac.uk/interpro/signature/SSF54211) |
|  | [IPR036890](http://www.ebi.ac.uk/interpro/entry/IPR036890) | HOMOLOGOUS_SUPERFAMILY | Histidine kinase/HSP90-like ATPase superfamily |  | [G3DSA:3.30.565.10(GENE3D)](http://www.ebi.ac.uk/interpro/signature/G3DSA:3.30.565.10) |
|  |  |  |  |  | [SSF55874(SUPERFAMILY)](http://www.ebi.ac.uk/interpro/signature/SSF55874) |
|  | [IPR037198](http://www.ebi.ac.uk/interpro/entry/IPR037198) | HOMOLOGOUS_SUPERFAMILY | MutL, C-terminal domain superfamily |  | [SSF118116(SUPERFAMILY)](http://www.ebi.ac.uk/interpro/signature/SSF118116) |
|  | no IPR | FAMILY | DNA mismatch repair protein family |  | [PF13589 (PFAM)](http://www.ebi.ac.uk/interpro/signature/PF13589) |
|  |  |  |  |  | [G3DSA:2.30.42.20(GENE3D)](http://www.ebi.ac.uk/interpro/signature/G3DSA:2.30.42.20) |
|  |  |  |  |  | [G3DSA:3.30.1370.100(GENE3D)](http://www.ebi.ac.uk/interpro/signature/G3DSA:3.30.1370.100) |
|  | no IPR | DOMAIN | Histidine kinase/HSP90-like ATPase |  | cd03484 (CDD) |
|  | no IPR | DOMAIN | MutL, C-terminal, dimerisation |  | mobidb-lite (MOBIDB_LITE) |
|  |  |  |  |  | mobidb-lite (MOBIDB_LITE) |
|  |  |  |  |  | [PTHR10073 (PANTHER)](http://www.ebi.ac.uk/interpro/signature/PTHR10073) |
|  |  |  |  |  | [PTHR10073:SF12 (PANTHER)](http://www.ebi.ac.uk/interpro/signature/PTHR10073) |
| *RsPNKP* | [IPR013954](http://www.ebi.ac.uk/interpro/entry/IPR013954) | FAMILY | Polynucleotide kinase 3 phosphatase |  | [PF08645 (PFAM)](http://www.ebi.ac.uk/interpro/signature/PF08645) |
|  | [IPR023214](http://www.ebi.ac.uk/interpro/entry/IPR023214) | HOMOLOGOUS_SUPERFAMILY | HAD superfamily |  | [G3DSA:3.40.50.1000 (GENE3D)](http://www.ebi.ac.uk/interpro/signature/G3DSA:3.40.50.1000) |
|  | [IPR027417](http://www.ebi.ac.uk/interpro/entry/IPR027417) | HOMOLOGOUS_SUPERFAMILY | P-loop containing nucleoside triphosphate hydrolase |  | [SSF52540 (SUPERFAMILY)](http://www.ebi.ac.uk/interpro/signature/SSF52540) |
|  | no IPR | HOMOLOGOUS_SUPERFAMILY | HAD superfamily |  | [G3DSA:3.40.50.300 (GENE3D)](http://www.ebi.ac.uk/interpro/signature/G3DSA:3.40.50.300) |
|  |  |  |  |  | [PF13671 (PFAM)](http://www.ebi.ac.uk/interpro/signature/PF13671) |
|  | no IPR | FAMILY | Polynucleotide kinase 3 phosphatase |  | [PTHR12083 (PANTHER)](http://www.ebi.ac.uk/interpro/signature/PTHR12083) |
|  |  |  |  |  | SIGNAL_PEPTIDE_H_REGION (PHOBIUS) |
|  |  |  |  |  | SIGNAL_PEPTIDE (PHOBIUS) |
|  |  |  |  |  | SIGNAL_PEPTIDE_N_REGION (PHOBIUS) |
|  |  |  |  |  | SIGNAL_PEPTIDE_C_REGION (PHOBIUS) |
|  |  |  |  |  | NON_CYTOPLASMIC_DOMAIN (PHOBIUS) |
|  |  |  |  |  | SignalP-noTM (SIGNALP_EUK) |
| *RsPOLB* | [IPR002008](http://www.ebi.ac.uk/interpro/entry/IPR002008) | FAMILY | DNA polymerase family X, beta-like | GO:0003677, GO:0006281 | [PR00870 (PRINTS)](http://www.ebi.ac.uk/interpro/signature/PR00870) |
|  | [IPR002054](http://www.ebi.ac.uk/interpro/entry/IPR002054) | DOMAIN | DNA-directed DNA polymerase X | GO:0003887, GO:0003677 | [SM00483 (SMART)](http://www.ebi.ac.uk/interpro/signature/SM00483) |
|  |  |  |  |  | cd00141 (CDD) |
|  | [IPR003583](http://www.ebi.ac.uk/interpro/entry/IPR003583) | DOMAIN | Helix-hairpin-helix DNA-binding motif, class 1 | GO:0003677, GO:0006281 | [SM00278 (SMART)](http://www.ebi.ac.uk/interpro/signature/SM00278) |
|  | [IPR010996](http://www.ebi.ac.uk/interpro/entry/IPR010996) | DOMAIN | DNA polymerase beta-like, N-terminal domain |  | [PF14716 (PFAM)](http://www.ebi.ac.uk/interpro/signature/PF14716) |
|  | [IPR018944](http://www.ebi.ac.uk/interpro/entry/IPR018944) | DOMAIN | DNA polymerase lambda, fingers domain | GO:0034061, GO:0003677, GO:0005634 | [PF10391 (PFAM)](http://www.ebi.ac.uk/interpro/signature/PF10391) |
|  | [IPR019843](http://www.ebi.ac.uk/interpro/entry/IPR019843) | BINDING_SITE | DNA polymerase family X, binding site | GO:0016779 | [PS00522(PROSITE_PATTERNS)](http://www.ebi.ac.uk/interpro/signature/PS00522) |
|  | [IPR022312](http://www.ebi.ac.uk/interpro/entry/IPR022312) | FAMILY | DNA polymerase family X | GO:0034061, GO:0003677 | [PR00869 (PRINTS)](http://www.ebi.ac.uk/interpro/signature/PR00869) |
|  | [IPR027421](http://www.ebi.ac.uk/interpro/entry/IPR027421) | HOMOLOGOUS_SUPERFAMILY | DNA polymerase lambda lyase domain superfamily |  | [G3DSA:1.10.150.110(GENE3D)](http://www.ebi.ac.uk/interpro/signature/G3DSA:1.10.150.110) |
|  |  |  |  |  | [SSF47802(SUPERFAMILY)](http://www.ebi.ac.uk/interpro/signature/SSF47802) |
|  | [IPR028207](http://www.ebi.ac.uk/interpro/entry/IPR028207) | DOMAIN | DNA polymerase beta, palm domain |  | [PF14792 (PFAM)](http://www.ebi.ac.uk/interpro/signature/PF14792) |
|  | [IPR029398](http://www.ebi.ac.uk/interpro/entry/IPR029398) | DOMAIN | DNA polymerase beta, thumb domain |  | [PF14791 (PFAM)](http://www.ebi.ac.uk/interpro/signature/PF14791) |
|  | [IPR037160](http://www.ebi.ac.uk/interpro/entry/IPR037160) | HOMOLOGOUS_SUPERFAMILY | DNA polymerase, thumb domain superfamily |  | [G3DSA:3.30.210.10(GENE3D)](http://www.ebi.ac.uk/interpro/signature/G3DSA:3.30.210.10) |
|  | no IPR | HOMOLOGOUS_SUPERFAMILY | DNA polymerase lambda lyase domain superfamily |  | [SSF81301(SUPERFAMILY)](http://www.ebi.ac.uk/interpro/signature/SSF81301) |
|  | no IPR | DOMAIN | DNA polymerase lambda, fingers domain |  | [G3DSA:1.10.150.20(GENE3D)](http://www.ebi.ac.uk/interpro/signature/G3DSA:1.10.150.20) |
|  | no IPR | HOMOLOGOUS_SUPERFAMILY | DNA polymerase, thumb domain superfamily |  | [PTHR11276 (PANTHER)](http://www.ebi.ac.uk/interpro/signature/PTHR11276) |
|  | no IPR | DOMAIN | DNA-directed DNA polymerase X |  | [G3DSA:3.30.460.10(GENE3D)](http://www.ebi.ac.uk/interpro/signature/G3DSA:3.30.460.10) |
|  |  |  |  |  | [SSF81585(SUPERFAMILY)](http://www.ebi.ac.uk/interpro/signature/SSF81585) |
| *RsPOLG1* | [IPR001098](http://www.ebi.ac.uk/interpro/entry/IPR001098) | DOMAIN | DNA-directed DNA polymerase, family A, palm domain | GO:0006260, GO:0003677, GO:0003887 | [SM00482 (SMART)](http://www.ebi.ac.uk/interpro/signature/SM00482) |
|  |  |  |  |  | [PF00476 (PFAM)](http://www.ebi.ac.uk/interpro/signature/PF00476) |
|  | [IPR002297](http://www.ebi.ac.uk/interpro/entry/IPR002297) | FAMILY | DNA-directed DNA-polymerase, family A, mitochondria | GO:0005760, GO:0006260, GO:0003677 | [PR00867 (PRINTS)](http://www.ebi.ac.uk/interpro/signature/PR00867) |
|  |  |  |  |  | [PTHR10267(PANTHER)](http://www.ebi.ac.uk/interpro/signature/PTHR10267) |
|  | [IPR012337](http://www.ebi.ac.uk/interpro/entry/IPR012337) | HOMOLOGOUS_SUPERFAMILY | Ribonuclease H-like superfamily |  | [SSF53098(SUPERFAMILY)](http://www.ebi.ac.uk/interpro/signature/SSF53098) |
|  | [IPR019760](http://www.ebi.ac.uk/interpro/entry/IPR019760) | CONSERVED_SITE | DNA-directed DNA polymerase, family A, conserved site | GO:0003887, GO:0006260 | [PS00447(PROSITE_PATTERNS)](http://www.ebi.ac.uk/interpro/signature/PS00447) |
|  | no IPR | CONSERVED_SITE | DNA-directed DNA polymerase, family A, conserved site |  | [SSF56672(SUPERFAMILY)](http://www.ebi.ac.uk/interpro/signature/SSF56672) |
|  | no IPR | DOMAIN | DNA-directed DNA polymerase, family A, palm domain |  | [G3DSA:1.10.150.20(GENE3D)](http://www.ebi.ac.uk/interpro/signature/G3DSA:1.10.150.20) |
|  |  |  |  |  | [G3DSA:3.30.420.390(GENE3D)](http://www.ebi.ac.uk/interpro/signature/G3DSA:3.30.420.390) |
|  |  |  |  |  | [G3DSA:3.30.70.370(GENE3D)](http://www.ebi.ac.uk/interpro/signature/G3DSA:3.30.70.370) |
|  | no IPR |  |  |  | Coil (COILS) |
| *RsPOLH* | [IPR001126](http://www.ebi.ac.uk/interpro/entry/IPR001126) | DOMAIN | UmuC domain | GO:0006281 | [PF00817 (PFAM)](http://www.ebi.ac.uk/interpro/signature/PF00817) |
|  |  |  |  |  | [PS50173(PROSITE_PROFILES)](http://www.ebi.ac.uk/interpro/signature/PS50173) |
|  | [IPR017061](http://www.ebi.ac.uk/interpro/entry/IPR017061) | FAMILY | DNA polymerase eta/kappa/iota/IV |  | PIRSF036603 (PIRSF) |
|  | [IPR017961](http://www.ebi.ac.uk/interpro/entry/IPR017961) | DOMAIN | DNA polymerase, Y-family, little finger domain | GO:0006281, GO:0003684 | [PF11799 (PFAM)](http://www.ebi.ac.uk/interpro/signature/PF11799) |
|  | [IPR036775](http://www.ebi.ac.uk/interpro/entry/IPR036775) | HOMOLOGOUS_SUPERFAMILY | DNA polymerase, Y-family, little finger domain superfamily | GO:0006281, GO:0003684 | [G3DSA:3.30.1490.100(GENE3D)](http://www.ebi.ac.uk/interpro/signature/G3DSA:3.30.1490.100) |
|  |  |  |  |  | [SSF100879(SUPERFAMILY)](http://www.ebi.ac.uk/interpro/signature/SSF100879) |
|  | no IPR | DOMAIN | DNA polymerase, Y-family, little finger domain |  | mobidb-lite (MOBIDB_LITE) |
|  |  |  |  |  | mobidb-lite (MOBIDB_LITE) |
|  |  |  |  |  | [PTHR11076:SF11 (PANTHER)](http://www.ebi.ac.uk/interpro/signature/PTHR11076) |
|  |  |  |  |  | [PTHR11076 (PANTHER)](http://www.ebi.ac.uk/interpro/signature/PTHR11076) |
|  | no IPR | DOMAIN | UmuC domain |  | [G3DSA:3.30.70.270(GENE3D)](http://www.ebi.ac.uk/interpro/signature/G3DSA:3.30.70.270) |
|  |  |  |  |  | [SSF56672(SUPERFAMILY)](http://www.ebi.ac.uk/interpro/signature/SSF56672) |
|  | no IPR |  |  |  | Coil (COILS) |
|  |  |  |  |  | [G3DSA:2.30.40.20(GENE3D)](http://www.ebi.ac.uk/interpro/signature/G3DSA:2.30.40.20) |
|  |  |  |  |  | [G3DSA:1.10.150.20(GENE3D)](http://www.ebi.ac.uk/interpro/signature/G3DSA:1.10.150.20) |
| *RsPOLI* | [IPR001126](http://www.ebi.ac.uk/interpro/entry/IPR001126) | DOMAIN | UmuC domain | GO:0006281 | [PF00817 (PFAM)](http://www.ebi.ac.uk/interpro/signature/PF00817) |
|  |  |  |  |  | [PS50173(PROSITE_PROFILES)](http://www.ebi.ac.uk/interpro/signature/PS50173) |
|  | [IPR017961](http://www.ebi.ac.uk/interpro/entry/IPR017961) | DOMAIN | DNA polymerase, Y-family, little finger domain | GO:0003684, GO:0006281 | [PF11799 (PFAM)](http://www.ebi.ac.uk/interpro/signature/PF11799) |
|  | [IPR036775](http://www.ebi.ac.uk/interpro/entry/IPR036775) | HOMOLOGOUS_SUPERFAMILY | DNA polymerase, Y-family, little finger domain superfamily | GO:0003684, GO:0006281 | [G3DSA:3.30.1490.100(GENE3D)](http://www.ebi.ac.uk/interpro/signature/G3DSA:3.30.1490.100) |
|  |  |  |  |  | [SSF100879(SUPERFAMILY)](http://www.ebi.ac.uk/interpro/signature/SSF100879) |
|  | no IPR | DOMAIN | DNA polymerase, Y-family, little finger domain |  | [G3DSA:2.30.40.20(GENE3D)](http://www.ebi.ac.uk/interpro/signature/G3DSA:2.30.40.20) |
|  |  |  |  |  | [G3DSA:3.30.70.270(GENE3D)](http://www.ebi.ac.uk/interpro/signature/G3DSA:3.30.70.270) |
|  |  |  |  |  | mobidb-lite (MOBIDB_LITE) |
|  |  |  |  |  | [PTHR11076:SF10 (PANTHER)](http://www.ebi.ac.uk/interpro/signature/PTHR11076) |
|  |  |  |  |  | [PTHR11076 (PANTHER)](http://www.ebi.ac.uk/interpro/signature/PTHR11076) |
|  | no IPR | HOMOLOGOUS_SUPERFAMILY | DNA polymerase, Y-family, little finger domain superfamily |  | [SSF56672(SUPERFAMILY)](http://www.ebi.ac.uk/interpro/signature/SSF56672) |
|  | no IPR |  |  |  | [G3DSA:1.10.150.20(GENE3D)](http://www.ebi.ac.uk/interpro/signature/G3DSA:1.10.150.20) |
| *RsPRIM2* | [IPR007238](http://www.ebi.ac.uk/interpro/entry/IPR007238) | FAMILY | DNA primase large subunit, eukaryotic/archaeal | GO:0003896, GO:0006269 | [PF04104 (PFAM)](http://www.ebi.ac.uk/interpro/signature/PF04104) |
|  |  |  |  |  | [PTHR10537 (PANTHER)](http://www.ebi.ac.uk/interpro/signature/PTHR10537) |
|  | [IPR016558](http://www.ebi.ac.uk/interpro/entry/IPR016558) | FAMILY | DNA primase, large subunit, eukaryotic | GO:0016779 | PIRSF009449 (PIRSF) |
|  |  |  |  |  | cd07322 (CDD) |
|  | no IPR | FAMILY | DNA primase large subunit, eukaryotic/archaeal |  | [PTHR10537:SF3 (PANTHER)](http://www.ebi.ac.uk/interpro/signature/PTHR10537) |
|  | no IPR |  |  |  | [G3DSA:1.20.930.80 (GENE3D)](http://www.ebi.ac.uk/interpro/signature/G3DSA:1.20.930.80) |
| *RsPRKDC* | [IPR000403](http://www.ebi.ac.uk/interpro/entry/IPR000403) | DOMAIN | Phosphatidylinositol 3-/4-kinase, catalytic domain |  | [SM00146 (SMART)](http://www.ebi.ac.uk/interpro/signature/SM00146) |
|  | [PF00454 (PFAM)](http://www.ebi.ac.uk/interpro/signature/PF00454) | |  |  |  |
|  | [PS50290(PROSITE_PROFILES)](http://www.ebi.ac.uk/interpro/signature/PS50290) | |  |  |  |
|  | [IPR003152](http://www.ebi.ac.uk/interpro/entry/IPR003152) | DOMAIN | FATC domain | GO:0005515 | [SM01343 (SMART)](http://www.ebi.ac.uk/interpro/signature/SM01343) |
|  |  |  |  |  | [PF02260 (PFAM)](http://www.ebi.ac.uk/interpro/signature/PF02260) |
|  |  |  |  |  | [PS51190(PROSITE_PROFILES)](http://www.ebi.ac.uk/interpro/signature/PS51190) |
|  | [IPR011009](http://www.ebi.ac.uk/interpro/entry/IPR011009) | HOMOLOGOUS_SUPERFAMILY | Protein kinase-like domain superfamily |  | [SSF56112 (SUPERFAMILY)](http://www.ebi.ac.uk/interpro/signature/SSF56112) |
|  | [IPR014009](http://www.ebi.ac.uk/interpro/entry/IPR014009) | DOMAIN | PIK-related kinase |  | [PS51189(PROSITE_PROFILES)](http://www.ebi.ac.uk/interpro/signature/PS51189) |
|  | [IPR016024](http://www.ebi.ac.uk/interpro/entry/IPR016024) | HOMOLOGOUS_SUPERFAMILY | Armadillo-type fold | GO:0005488 | [SSF48371 (SUPERFAMILY)](http://www.ebi.ac.uk/interpro/signature/SSF48371) |
|  |  |  |  |  | [SSF48371 (SUPERFAMILY)](http://www.ebi.ac.uk/interpro/signature/SSF48371) |
|  | [IPR018936](http://www.ebi.ac.uk/interpro/entry/IPR018936) | CONSERVED_SITE | Phosphatidylinositol 3/4-kinase, conserved site | GO:0016301 | [PS00915(PROSITE_PATTERNS)](http://www.ebi.ac.uk/interpro/signature/PS00915) |
|  |  |  |  |  | [PS00916(PROSITE_PATTERNS)](http://www.ebi.ac.uk/interpro/signature/PS00916) |
|  | [IPR036940](http://www.ebi.ac.uk/interpro/entry/IPR036940) | HOMOLOGOUS_SUPERFAMILY | Phosphatidylinositol 3-/4-kinase, catalytic domain superfamily | GO:0016301 | [G3DSA:1.10.1070.11(GENE3D)](http://www.ebi.ac.uk/interpro/signature/G3DSA:1.10.1070.11) |
|  | [IPR037706](http://www.ebi.ac.uk/interpro/entry/IPR037706) | DOMAIN | DNA-dependent protein kinase catalytic subunit, catalytic domain | GO:0004677, GO:0006281 | cd05172 (CDD) |
|  | no IPR | DOMAIN | DNA-dependent protein kinase catalytic subunit, catalytic domain |  | SignalP-noTM (SIGNALP_EUK) |
|  | no IPR | DOMAIN | Phosphatidylinositol 3-/4-kinase, catalytic domain |  | mobidb-lite (MOBIDB_LITE) |
|  |  |  |  |  | [PTHR11139 (PANTHER)](http://www.ebi.ac.uk/interpro/signature/PTHR11139) |
|  |  |  |  |  | [PTHR11139:SF68 (PANTHER)](http://www.ebi.ac.uk/interpro/signature/PTHR11139) |
|  | no IPR | HOMOLOGOUS_SUPERFAMILY | Phosphatidylinositol 3-/4-kinase, catalytic domain superfamily |  | [G3DSA:3.30.1010.10(GENE3D)](http://www.ebi.ac.uk/interpro/signature/G3DSA:3.30.1010.10) |
|  | no IPR | CONSERVED_SITE | Phosphatidylinositol 3/4-kinase, conserved site |  | SIGNAL_PEPTIDE_C_REGION (PHOBIUS) |
|  |  |  |  |  | NON_CYTOPLASMIC_DOMAIN (PHOBIUS) |
|  |  |  |  |  | SIGNAL_PEPTIDE (PHOBIUS) |
|  |  |  |  |  | SIGNAL_PEPTIDE_N_REGION (PHOBIUS) |
|  |  |  |  |  | SIGNAL_PEPTIDE_H_REGION (PHOBIUS) |
|  | no IPR |  |  |  | Coil (COILS) |
|  |  |  |  |  | Coil (COILS) |
| *RsRAD1* | [IPR003011](http://www.ebi.ac.uk/interpro/entry/IPR003011) | FAMILY | Cell cycle checkpoint protein, Rad1 | GO:0000077, GO:0005634 | [PR01246 (PRINTS)](http://www.ebi.ac.uk/interpro/signature/PR01246) |
|  | [IPR003021](http://www.ebi.ac.uk/interpro/entry/IPR003021) | FAMILY | Rad1/Rec1/Rad17 | GO:0000077, GO:0005634 | [PR01245 (PRINTS)](http://www.ebi.ac.uk/interpro/signature/PR01245) |
|  |  |  |  |  | [PF02144 (PFAM)](http://www.ebi.ac.uk/interpro/signature/PF02144) |
|  |  |  |  |  | [PTHR10870 (PANTHER)](http://www.ebi.ac.uk/interpro/signature/PTHR10870) |
|  | no IPR | FAMILY | Cell cycle checkpoint protein, Rad1 |  | [G3DSA:3.70.10.10 (GENE3D)](http://www.ebi.ac.uk/interpro/signature/G3DSA:3.70.10.10) |
|  | no IPR | FAMILY | Rad1/Rec1/Rad17 |  | cd00577 (CDD) |
|  |  |  |  |  | [SSF55979 (SUPERFAMILY)](http://www.ebi.ac.uk/interpro/signature/SSF55979) |
| *RsRAD17* | [IPR004582](http://www.ebi.ac.uk/interpro/entry/IPR004582) | FAMILY | Checkpoint protein Rad17/Rad24 | GO:0006281, GO:0005634, GO:0007049 | [PTHR12172(PANTHER)](http://www.ebi.ac.uk/interpro/signature/PTHR12172) |
|  | [IPR027417](http://www.ebi.ac.uk/interpro/entry/IPR027417) | HOMOLOGOUS_SUPERFAMILY | P-loop containing nucleoside triphosphate hydrolase |  | [SSF52540(SUPERFAMILY)](http://www.ebi.ac.uk/interpro/signature/SSF52540) |
|  | no IPR | FAMILY | Checkpoint protein Rad17/Rad24 |  | [PTHR12172:SF0 (PANTHER)](http://www.ebi.ac.uk/interpro/signature/PTHR12172) |
|  |  |  |  |  | cd00009 (CDD) |
|  | no IPR |  |  |  | [G3DSA:3.40.50.300(GENE3D)](http://www.ebi.ac.uk/interpro/signature/G3DSA:3.40.50.300) |
|  |  |  |  |  | [PF03215 (PFAM)](http://www.ebi.ac.uk/interpro/signature/PF03215) |
|  |  |  |  |  | mobidb-lite (MOBIDB_LITE) |
| *RsRAD18* | [IPR001841](http://www.ebi.ac.uk/interpro/entry/IPR001841) | DOMAIN | Zinc finger, RING-type |  | [SM00184 (SMART)](http://www.ebi.ac.uk/interpro/signature/SM00184) |
|  |  |  |  |  | [PS50089(PROSITE_PROFILES)](http://www.ebi.ac.uk/interpro/signature/PS50089) |
|  | [IPR003034](http://www.ebi.ac.uk/interpro/entry/IPR003034) | DOMAIN | SAP domain |  | [SM00513 (SMART)](http://www.ebi.ac.uk/interpro/signature/SM00513) |
|  |  |  |  |  | [PS50800(PROSITE_PROFILES)](http://www.ebi.ac.uk/interpro/signature/PS50800) |
|  | [IPR006642](http://www.ebi.ac.uk/interpro/entry/IPR006642) | DOMAIN | Zinc finger, Rad18-type putative | GO:0006281, GO:0003677 | [SM00734 (SMART)](http://www.ebi.ac.uk/interpro/signature/SM00734) |
|  | [IPR013083](http://www.ebi.ac.uk/interpro/entry/IPR013083) | HOMOLOGOUS_SUPERFAMILY | Zinc finger, RING/FYVE/PHD-type |  | [G3DSA:3.30.40.10 (GENE3D)](http://www.ebi.ac.uk/interpro/signature/G3DSA:3.30.40.10) |
|  | [IPR017907](http://www.ebi.ac.uk/interpro/entry/IPR017907) | CONSERVED_SITE | Zinc finger, RING-type, conserved site |  | [PS00518(PROSITE_PATTERNS)](http://www.ebi.ac.uk/interpro/signature/PS00518) |
|  | no IPR | DOMAIN | SAP domain |  | cd16529 (CDD) |
|  |  |  |  |  | [SSF57850 (SUPERFAMILY)](http://www.ebi.ac.uk/interpro/signature/SSF57850) |
|  | no IPR | HOMOLOGOUS_SUPERFAMILY | Zinc finger, RING/FYVE/PHD-type |  | [G3DSA:3.30.160.60 (GENE3D)](http://www.ebi.ac.uk/interpro/signature/G3DSA:3.30.160.60) |
|  |  |  |  |  | mobidb-lite (MOBIDB_LITE) |
|  |  |  |  |  | [PTHR14134 (PANTHER)](http://www.ebi.ac.uk/interpro/signature/PTHR14134) |
|  |  |  |  |  | [PTHR14134:SF2 (PANTHER)](http://www.ebi.ac.uk/interpro/signature/PTHR14134) |
|  | no IPR | DOMAIN | Zinc finger, Rad18-type putative |  | [PF13923 (PFAM)](http://www.ebi.ac.uk/interpro/signature/PF13923) |
| *RsRAD23B* | [IPR000626](http://www.ebi.ac.uk/interpro/entry/IPR000626) | DOMAIN | Ubiquitin domain | GO:0005515 | [SM00213 (SMART)](http://www.ebi.ac.uk/interpro/signature/SM00213) |
|  |  |  |  |  | [PF00240 (PFAM)](http://www.ebi.ac.uk/interpro/signature/PF00240) |
|  |  |  |  |  | [PS50053(PROSITE_PROFILES)](http://www.ebi.ac.uk/interpro/signature/PS50053) |
|  | [IPR004806](http://www.ebi.ac.uk/interpro/entry/IPR004806) | FAMILY | UV excision repair protein Rad23 | GO:0005634, GO:0006289 | [PR01839 (PRINTS)](http://www.ebi.ac.uk/interpro/signature/PR01839) |
|  |  |  |  |  | [TIGR00601 (TIGRFAM)](http://www.ebi.ac.uk/interpro/signature/TIGR00601) |
|  | [IPR006636](http://www.ebi.ac.uk/interpro/entry/IPR006636) | DOMAIN | Heat shock chaperonin-binding |  | [SM00727 (SMART)](http://www.ebi.ac.uk/interpro/signature/SM00727) |
|  | [IPR009060](http://www.ebi.ac.uk/interpro/entry/IPR009060) | HOMOLOGOUS_SUPERFAMILY | UBA-like superfamily | GO:0005515 | [SSF46934 (SUPERFAMILY)](http://www.ebi.ac.uk/interpro/signature/SSF46934) |
|  |  |  |  |  | [SSF46934 (SUPERFAMILY)](http://www.ebi.ac.uk/interpro/signature/SSF46934) |
|  | [IPR015360](http://www.ebi.ac.uk/interpro/entry/IPR015360) | DOMAIN | XPC-binding domain | GO:0043161, GO:0003684, GO:0006289 | [PF09280 (PFAM)](http://www.ebi.ac.uk/interpro/signature/PF09280) |
|  | [IPR015940](http://www.ebi.ac.uk/interpro/entry/IPR015940) | DOMAIN | Ubiquitin-associated domain |  | [SM00165 (SMART)](http://www.ebi.ac.uk/interpro/signature/SM00165) |
|  |  |  |  |  | [PF00627 (PFAM)](http://www.ebi.ac.uk/interpro/signature/PF00627) |
|  |  |  |  |  | [PS50030(PROSITE_PROFILES)](http://www.ebi.ac.uk/interpro/signature/PS50030) |
|  |  |  |  |  | [PS50030(PROSITE_PROFILES)](http://www.ebi.ac.uk/interpro/signature/PS50030) |
|  | [IPR029071](http://www.ebi.ac.uk/interpro/entry/IPR029071) | HOMOLOGOUS_SUPERFAMILY | Ubiquitin-like domain superfamily |  | [SSF54236 (SUPERFAMILY)](http://www.ebi.ac.uk/interpro/signature/SSF54236) |
|  | [IPR036353](http://www.ebi.ac.uk/interpro/entry/IPR036353) | HOMOLOGOUS_SUPERFAMILY | XPC-binding domain superfamily | GO:0003684, GO:0043161, GO:0006289 | [G3DSA:1.10.10.540(GENE3D)](http://www.ebi.ac.uk/interpro/signature/G3DSA:1.10.10.540) |
|  |  |  |  |  | [SSF101238 (SUPERFAMILY)](http://www.ebi.ac.uk/interpro/signature/SSF101238) |
|  | no IPR | DOMAIN | Ubiquitin domain |  | [G3DSA:1.10.8.10 (GENE3D)](http://www.ebi.ac.uk/interpro/signature/G3DSA:1.10.8.10) |
|  |  |  |  |  | cd14378 (CDD) |
|  |  |  |  |  | cd14427 (CDD) |
|  | no IPR | DOMAIN | XPC-binding domain |  | [G3DSA:3.10.20.90(GENE3D)](http://www.ebi.ac.uk/interpro/signature/G3DSA:3.10.20.90) |
|  | no IPR | HOMOLOGOUS_SUPERFAMILY | XPC-binding domain superfamily |  | [G3DSA:1.10.8.10 (GENE3D)](http://www.ebi.ac.uk/interpro/signature/G3DSA:1.10.8.10) |
|  |  |  |  |  | mobidb-lite (MOBIDB_LITE) |
|  |  |  |  |  | mobidb-lite (MOBIDB_LITE) |
|  |  |  |  |  | mobidb-lite (MOBIDB_LITE) |
|  |  |  |  |  | [PTHR10621:SF13 (PANTHER)](http://www.ebi.ac.uk/interpro/signature/PTHR10621) |
|  |  |  |  |  | [PTHR10621 (PANTHER)](http://www.ebi.ac.uk/interpro/signature/PTHR10621) |
|  | no IPR |  |  |  | Coil (COILS) |
| *RsRAD50* | [IPR003959](http://www.ebi.ac.uk/interpro/entry/IPR003959) | DOMAIN | ATPase, AAA-type, core | GO:0005524 | [PF13304 (PFAM)](http://www.ebi.ac.uk/interpro/signature/PF13304) |
|  | [IPR004584](http://www.ebi.ac.uk/interpro/entry/IPR004584) | FAMILY | DNA repair protein Rad50, eukaryotes | GO:0030870, GO:0006281, GO:0000723, GO:0016887, GO:0005634 | [TIGR00606(TIGRFAM)](http://www.ebi.ac.uk/interpro/signature/TIGR00606) |
|  | [IPR013134](http://www.ebi.ac.uk/interpro/entry/IPR013134) | DOMAIN | RAD50, zinc hook |  | [PF04423 (PFAM)](http://www.ebi.ac.uk/interpro/signature/PF04423) |
|  |  |  |  |  | [PS51131(PROSITE_PROFILES)](http://www.ebi.ac.uk/interpro/signature/PS51131) |
|  | [IPR027417](http://www.ebi.ac.uk/interpro/entry/IPR027417) | HOMOLOGOUS_SUPERFAMILY | P-loop containing nucleoside triphosphate hydrolase |  | [SSF52540(SUPERFAMILY)](http://www.ebi.ac.uk/interpro/signature/SSF52540) |
|  |  |  |  |  | [SSF52540(SUPERFAMILY)](http://www.ebi.ac.uk/interpro/signature/SSF52540) |
|  | no IPR | DOMAIN | ATPase, AAA-type, core |  | [PTHR18867(PANTHER)](http://www.ebi.ac.uk/interpro/signature/PTHR18867) |
|  | no IPR | HOMOLOGOUS_SUPERFAMILY | P-loop containing nucleoside triphosphate hydrolase |  | [SSF75712(SUPERFAMILY)](http://www.ebi.ac.uk/interpro/signature/SSF75712) |
|  | no IPR | DOMAIN | RAD50, zinc hook |  | [G3DSA:3.40.50.300(GENE3D)](http://www.ebi.ac.uk/interpro/signature/G3DSA:3.40.50.300) |
|  |  |  |  |  | [G3DSA:3.40.50.300(GENE3D)](http://www.ebi.ac.uk/interpro/signature/G3DSA:3.40.50.300) |
|  |  |  |  |  | [PF13476 (PFAM)](http://www.ebi.ac.uk/interpro/signature/PF13476) |
|  |  |  |  |  | cd03240 (CDD) |
|  |  |  |  |  | cd03240 (CDD) |
|  | no IPR |  |  |  | Coil (COILS) |
|  |  |  |  |  | Coil (COILS) |
|  |  |  |  |  | Coil (COILS) |
|  |  |  |  |  | Coil (COILS) |
|  |  |  |  |  | Coil (COILS) |
|  |  |  |  |  | Coil (COILS) |
|  |  |  |  |  | Coil (COILS) |
|  |  |  |  |  | Coil (COILS) |
|  |  |  |  |  | Coil (COILS) |
|  |  |  |  |  | Coil (COILS) |
|  |  |  |  |  | Coil (COILS) |
|  |  |  |  |  | Coil (COILS) |
|  |  |  |  |  | Coil (COILS) |
|  |  |  |  |  | Coil (COILS) |
|  |  |  |  |  | Coil (COILS) |
|  |  |  |  |  | Coil (COILS) |
| *RsRAD51A* | [IPR003593](http://www.ebi.ac.uk/interpro/entry/IPR003593) | DOMAIN | AAA+ ATPase domain |  | [SM00382 (SMART)](http://www.ebi.ac.uk/interpro/signature/SM00382) |
|  | [IPR010995](http://www.ebi.ac.uk/interpro/entry/IPR010995) | HOMOLOGOUS_SUPERFAMILY | DNA repair Rad51/transcription factor NusA, alpha-helical | GO:0000166 | [SSF47794 (SUPERFAMILY)](http://www.ebi.ac.uk/interpro/signature/SSF47794) |
|  | [IPR011941](http://www.ebi.ac.uk/interpro/entry/IPR011941) | FAMILY | DNA recombination/repair protein Rad51 | GO:0003697, GO:0000724, GO:0000150, GO:0008094, GO:0003690, GO:1990426 | [TIGR02239 (TIGRFAM)](http://www.ebi.ac.uk/interpro/signature/TIGR02239) |
|  | [IPR013632](http://www.ebi.ac.uk/interpro/entry/IPR013632) | DOMAIN | DNA recombination and repair protein Rad51-like, C-terminal |  | [PF08423 (PFAM)](http://www.ebi.ac.uk/interpro/signature/PF08423) |
|  | [IPR016467](http://www.ebi.ac.uk/interpro/entry/IPR016467) | FAMILY | DNA recombination and repair protein, RecA-like |  | PIRSF005856 (PIRSF) |
|  | [IPR020587](http://www.ebi.ac.uk/interpro/entry/IPR020587) | DOMAIN | DNA recombination and repair protein RecA, monomer-monomer interface | GO:0003677, GO:0005524, GO:0008094, GO:0006259 | [PS50163(PROSITE_PROFILES)](http://www.ebi.ac.uk/interpro/signature/PS50163) |
|  | [IPR020588](http://www.ebi.ac.uk/interpro/entry/IPR020588) | DOMAIN | DNA recombination and repair protein RecA-like, ATP-binding domain | GO:0003677, GO:0008094, GO:0005524, GO:0006281 | [PS50162(PROSITE_PROFILES)](http://www.ebi.ac.uk/interpro/signature/PS50162) |
|  | [IPR027417](http://www.ebi.ac.uk/interpro/entry/IPR027417) | HOMOLOGOUS_SUPERFAMILY | P-loop containing nucleoside triphosphate hydrolase |  | [SSF52540 (SUPERFAMILY)](http://www.ebi.ac.uk/interpro/signature/SSF52540) |
|  | [IPR033925](http://www.ebi.ac.uk/interpro/entry/IPR033925) | DOMAIN | Rad51/DMC1/RadA | GO:0003677 | cd01123 (CDD) |
|  | no IPR | DOMAIN | AAA+ ATPase domain |  | [G3DSA:1.10.150.20 (GENE3D)](http://www.ebi.ac.uk/interpro/signature/G3DSA:1.10.150.20) |
|  |  |  |  |  | [G3DSA:3.40.50.300 (GENE3D)](http://www.ebi.ac.uk/interpro/signature/G3DSA:3.40.50.300) |
|  | no IPR | DOMAIN | DNA recombination and repair protein Rad51-like, C-terminal |  | [PTHR22942:SF39 (PANTHER)](http://www.ebi.ac.uk/interpro/signature/PTHR22942) |
|  |  |  |  |  | [PTHR22942 (PANTHER)](http://www.ebi.ac.uk/interpro/signature/PTHR22942) |
|  |  |  |  |  | SIGNAL_PEPTIDE_N_REGION (PHOBIUS) |
|  |  |  |  |  | SIGNAL_PEPTIDE_H_REGION (PHOBIUS) |
|  |  |  |  |  | SIGNAL_PEPTIDE_C_REGION (PHOBIUS) |
|  |  |  |  |  | NON_CYTOPLASMIC_DOMAIN (PHOBIUS) |
|  |  |  |  |  | SIGNAL_PEPTIDE (PHOBIUS) |
|  | no IPR | FAMILY | DNA recombination and repair protein, RecA-like |  | [PF14520 (PFAM)](http://www.ebi.ac.uk/interpro/signature/PF14520) |
| *RsRAD51C* | [IPR013632](http://www.ebi.ac.uk/interpro/entry/IPR013632) | DOMAIN | DNA recombination and repair protein Rad51-like, C-terminal |  | [PF08423 (PFAM)](http://www.ebi.ac.uk/interpro/signature/PF08423) |
|  | [IPR016467](http://www.ebi.ac.uk/interpro/entry/IPR016467) | FAMILY | DNA recombination and repair protein, RecA-like |  | PIRSF005856 (PIRSF) |
|  | [IPR020588](http://www.ebi.ac.uk/interpro/entry/IPR020588) | DOMAIN | DNA recombination and repair protein RecA-like, ATP-binding domain | GO:0008094, GO:0006281, GO:0003677, GO:0005524 | [PS50162(PROSITE_PROFILES)](http://www.ebi.ac.uk/interpro/signature/PS50162) |
|  | [IPR027417](http://www.ebi.ac.uk/interpro/entry/IPR027417) | HOMOLOGOUS_SUPERFAMILY | P-loop containing nucleoside triphosphate hydrolase |  | [SSF52540(SUPERFAMILY)](http://www.ebi.ac.uk/interpro/signature/SSF52540) |
|  | [IPR033925](http://www.ebi.ac.uk/interpro/entry/IPR033925) | DOMAIN | Rad51/DMC1/RadA | GO:0003677 | cd01123 (CDD) |
|  | no IPR | DOMAIN | DNA recombination and repair protein Rad51-like, C-terminal |  | [G3DSA:3.40.50.300(GENE3D)](http://www.ebi.ac.uk/interpro/signature/G3DSA:3.40.50.300) |
|  |  |  |  |  | [PTHR22942(PANTHER)](http://www.ebi.ac.uk/interpro/signature/PTHR22942) |
|  |  |  |  |  | [PTHR22942:SF14 (PANTHER)](http://www.ebi.ac.uk/interpro/signature/PTHR22942) |
| *RsRAD51D* | [IPR013632](http://www.ebi.ac.uk/interpro/entry/IPR013632) | DOMAIN | DNA recombination and repair protein Rad51-like, C-terminal |  | [PF08423 (PFAM)](http://www.ebi.ac.uk/interpro/signature/PF08423) |
|  | [IPR020588](http://www.ebi.ac.uk/interpro/entry/IPR020588) | DOMAIN | DNA recombination and repair protein RecA-like, ATP-binding domain | GO:0005524, GO:0006281, GO:0008094, GO:0003677 | [PS50162(PROSITE_PROFILES)](http://www.ebi.ac.uk/interpro/signature/PS50162) |
|  | [IPR027417](http://www.ebi.ac.uk/interpro/entry/IPR027417) | HOMOLOGOUS_SUPERFAMILY | P-loop containing nucleoside triphosphate hydrolase |  | [SSF52540(SUPERFAMILY)](http://www.ebi.ac.uk/interpro/signature/SSF52540) |
|  | no IPR | DOMAIN | DNA recombination and repair protein Rad51-like, C-terminal |  | [G3DSA:1.10.150.20(GENE3D)](http://www.ebi.ac.uk/interpro/signature/G3DSA:1.10.150.20) |
|  |  |  |  |  | [PTHR22942:SF29 (PANTHER)](http://www.ebi.ac.uk/interpro/signature/PTHR22942) |
|  |  |  |  |  | [PTHR22942(PANTHER)](http://www.ebi.ac.uk/interpro/signature/PTHR22942) |
|  | no IPR |  |  |  | [G3DSA:3.40.50.300(GENE3D)](http://www.ebi.ac.uk/interpro/signature/G3DSA:3.40.50.300) |
| *RsRAD52* | [IPR007232](http://www.ebi.ac.uk/interpro/entry/IPR007232) | FAMILY | DNA repair protein Rad52/59/22 | GO:0006281, GO:0006310 | [PF04098 (PFAM)](http://www.ebi.ac.uk/interpro/signature/PF04098) |
|  |  |  |  |  | [PTHR12132 (PANTHER)](http://www.ebi.ac.uk/interpro/signature/PTHR12132) |
|  |  |  |  |  | [PTHR12132 (PANTHER)](http://www.ebi.ac.uk/interpro/signature/PTHR12132) |
|  | no IPR | FAMILY | DNA repair protein Rad52/59/22 |  | [G3DSA:3.30.390.80 (GENE3D)](http://www.ebi.ac.uk/interpro/signature/G3DSA:3.30.390.80) |
|  |  |  |  |  | mobidb-lite (MOBIDB_LITE) |
|  |  |  |  |  | [PTHR12132:SF1 (PANTHER)](http://www.ebi.ac.uk/interpro/signature/PTHR12132) |
|  |  |  |  |  | [PTHR12132:SF1 (PANTHER)](http://www.ebi.ac.uk/interpro/signature/PTHR12132) |
|  |  |  |  |  | [SSF54768 (SUPERFAMILY)](http://www.ebi.ac.uk/interpro/signature/SSF54768) |
| *RsRAD54B* | [IPR000330](http://www.ebi.ac.uk/interpro/entry/IPR000330) | DOMAIN | SNF2-related, N-terminal domain | [GO:0005524](file:///C:\go_info\GO\0005524$1) | [PF00176 (PFAM)](http://www.ebi.ac.uk/interpro/signature/PF00176) |
|  | [IPR001650](http://www.ebi.ac.uk/interpro/entry/IPR001650) | DOMAIN | Helicase, C-terminal |  | [SM00490 (SMART)](http://www.ebi.ac.uk/interpro/signature/SM00490) |
|  |  |  |  |  | [PF00271 (PFAM)](http://www.ebi.ac.uk/interpro/signature/PF00271) |
|  |  |  |  |  | [PS51194(PROSITE_PROFILES)](http://www.ebi.ac.uk/interpro/signature/PS51194) |
|  |  |  |  |  | cd00079 (CDD) |
|  | [IPR014001](http://www.ebi.ac.uk/interpro/entry/IPR014001) | DOMAIN | Helicase superfamily 1/2, ATP-binding domain |  | [SM00487 (SMART)](http://www.ebi.ac.uk/interpro/signature/SM00487) |
|  |  |  |  |  | [PS51192(PROSITE_PROFILES)](http://www.ebi.ac.uk/interpro/signature/PS51192) |
|  | [IPR027417](http://www.ebi.ac.uk/interpro/entry/IPR027417) | HOMOLOGOUS_SUPERFAMILY | P-loop containing nucleoside triphosphate hydrolase |  | [SSF52540 (SUPERFAMILY)](http://www.ebi.ac.uk/interpro/signature/SSF52540) |
|  |  |  |  |  | [SSF52540 (SUPERFAMILY)](http://www.ebi.ac.uk/interpro/signature/SSF52540) |
|  | [IPR038718](http://www.ebi.ac.uk/interpro/entry/IPR038718) | HOMOLOGOUS_SUPERFAMILY | SNF2-like, N-terminal domain superfamily |  | [G3DSA:3.40.50.10810 (GENE3D)](http://www.ebi.ac.uk/interpro/signature/G3DSA:3.40.50.1081) |
|  | no IPR | DOMAIN | Helicase superfamily 1/2, ATP-binding domain |  | cd00046 (CDD) |
|  | no IPR | DOMAIN | Helicase, C-terminal |  | [G3DSA:1.20.120.850(GENE3D)](http://www.ebi.ac.uk/interpro/signature/G3DSA:1.20.120.850) |
|  |  |  |  |  | [PTHR10799:SF889 (PANTHER)](http://www.ebi.ac.uk/interpro/signature/PTHR10799) |
|  |  |  |  |  | [PTHR10799 (PANTHER)](http://www.ebi.ac.uk/interpro/signature/PTHR10799) |
|  | no IPR | DOMAIN | SNF2-related, N-terminal domain |  | [G3DSA:3.40.50.300 (GENE3D)](http://www.ebi.ac.uk/interpro/signature/G3DSA:3.40.50.300) |
| *RsRAD54-like* | [IPR000330](http://www.ebi.ac.uk/interpro/entry/IPR000330) | DOMAIN | SNF2-related, N-terminal domain | GO:0005524 | [PF00176 (PFAM)](http://www.ebi.ac.uk/interpro/signature/PF00176) |
|  | [IPR001650](http://www.ebi.ac.uk/interpro/entry/IPR001650) | DOMAIN | Helicase, C-terminal |  | [SM00490 (SMART)](http://www.ebi.ac.uk/interpro/signature/SM00490) |
|  |  |  |  |  | [PF00271 (PFAM)](http://www.ebi.ac.uk/interpro/signature/PF00271) |
|  |  |  |  |  | [PS51194(PROSITE_PROFILES)](http://www.ebi.ac.uk/interpro/signature/PS51194) |
|  |  |  |  |  | cd00079 (CDD) |
|  | [IPR014001](http://www.ebi.ac.uk/interpro/entry/IPR014001) | DOMAIN | Helicase superfamily 1/2, ATP-binding domain |  | [SM00487 (SMART)](http://www.ebi.ac.uk/interpro/signature/SM00487) |
|  |  |  |  |  | [PS51192(PROSITE_PROFILES)](http://www.ebi.ac.uk/interpro/signature/PS51192) |
|  | [IPR027417](http://www.ebi.ac.uk/interpro/entry/IPR027417) | HOMOLOGOUS_SUPERFAMILY | P-loop containing nucleoside triphosphate hydrolase |  | [SSF52540 (SUPERFAMILY)](http://www.ebi.ac.uk/interpro/signature/SSF52540) |
|  |  |  |  |  | [SSF52540 (SUPERFAMILY)](http://www.ebi.ac.uk/interpro/signature/SSF52540) |
|  | [IPR038718](http://www.ebi.ac.uk/interpro/entry/IPR038718) | HOMOLOGOUS_SUPERFAMILY | SNF2-like, N-terminal domain superfamily |  | [G3DSA:3.40.50.10810 (GENE3D)](http://www.ebi.ac.uk/interpro/signature/G3DSA:3.40.50.1081) |
|  | no IPR | DOMAIN | Helicase, C-terminal |  | [G3DSA:1.20.120.850(GENE3D)](http://www.ebi.ac.uk/interpro/signature/G3DSA:1.20.120.850) |
|  |  |  |  |  | [G3DSA:3.40.50.300 (GENE3D)](http://www.ebi.ac.uk/interpro/signature/G3DSA:3.40.50.300) |
|  |  |  |  |  | [PTHR10799:SF889 (PANTHER)](http://www.ebi.ac.uk/interpro/signature/PTHR10799) |
|  |  |  |  |  | [PTHR10799 (PANTHER)](http://www.ebi.ac.uk/interpro/signature/PTHR10799) |
|  |  |  |  |  | cd00046 (CDD) |
| *RsRAD9A* | [IPR007268](http://www.ebi.ac.uk/interpro/entry/IPR007268) | FAMILY | Rad9/Ddc1 | GO:0030896, GO:0000077 | [PF04139 (PFAM)](http://www.ebi.ac.uk/interpro/signature/PF04139) |
|  |  |  |  |  | [PTHR15237 (PANTHER)](http://www.ebi.ac.uk/interpro/signature/PTHR15237) |
|  | [IPR026584](http://www.ebi.ac.uk/interpro/entry/IPR026584) | FAMILY | Rad9 | GO:0006281, GO:0030896, GO:0000075, GO:0000077 | PIRSF009303 (PIRSF) |
|  | no IPR | FAMILY | Rad9 |  | mobidb-lite (MOBIDB_LITE) |
|  | no IPR | FAMILY | Rad9/Ddc1 |  | [SSF55979 (SUPERFAMILY)](http://www.ebi.ac.uk/interpro/signature/SSF55979) |
|  | no IPR |  |  |  | [G3DSA:3.70.10.10 (GENE3D)](http://www.ebi.ac.uk/interpro/signature/G3DSA:3.70.10.10) |
| *RsRECQL4* | [IPR001650](http://www.ebi.ac.uk/interpro/entry/IPR001650) | DOMAIN | Helicase, C-terminal |  | [SM00490 (SMART)](http://www.ebi.ac.uk/interpro/signature/SM00490) |
|  |  |  |  |  | [PF00271 (PFAM)](http://www.ebi.ac.uk/interpro/signature/PF00271) |
|  |  |  |  |  | [PS51194(PROSITE_PROFILES)](http://www.ebi.ac.uk/interpro/signature/PS51194) |
|  |  |  |  |  | cd00079 (CDD) |
|  | [IPR001878](http://www.ebi.ac.uk/interpro/entry/IPR001878) | DOMAIN | Zinc finger, CCHC-type | GO:0008270, GO:0003676 | [SM00343 (SMART)](http://www.ebi.ac.uk/interpro/signature/SM00343) |
|  |  |  |  |  | [PF00098 (PFAM)](http://www.ebi.ac.uk/interpro/signature/PF00098) |
|  |  |  |  |  | [PS50158(PROSITE_PROFILES)](http://www.ebi.ac.uk/interpro/signature/PS50158) |
|  | [IPR004589](http://www.ebi.ac.uk/interpro/entry/IPR004589) | FAMILY | DNA helicase, ATP-dependent, RecQ type | GO:0006310, GO:0008026 | [TIGR00614 (TIGRFAM)](http://www.ebi.ac.uk/interpro/signature/TIGR00614) |
|  | [IPR011545](http://www.ebi.ac.uk/interpro/entry/IPR011545) | DOMAIN | DEAD/DEAH box helicase domain | GO:0003676, GO:0005524 | [PF00270 (PFAM)](http://www.ebi.ac.uk/interpro/signature/PF00270) |
|  | [IPR014001](http://www.ebi.ac.uk/interpro/entry/IPR014001) | DOMAIN | Helicase superfamily 1/2, ATP-binding domain |  | [SM00487 (SMART)](http://www.ebi.ac.uk/interpro/signature/SM00487) |
|  |  |  |  |  | [PS51192(PROSITE_PROFILES)](http://www.ebi.ac.uk/interpro/signature/PS51192) |
|  | [IPR021110](http://www.ebi.ac.uk/interpro/entry/IPR021110) | FAMILY | DNA replication/checkpoint protein |  | [PF11719 (PFAM)](http://www.ebi.ac.uk/interpro/signature/PF11719) |
|  | [IPR027417](http://www.ebi.ac.uk/interpro/entry/IPR027417) | HOMOLOGOUS_SUPERFAMILY | P-loop containing nucleoside triphosphate hydrolase |  | [SSF52540(SUPERFAMILY)](http://www.ebi.ac.uk/interpro/signature/SSF52540) |
|  | [IPR036875](http://www.ebi.ac.uk/interpro/entry/IPR036875) | HOMOLOGOUS_SUPERFAMILY | Zinc finger, CCHC-type superfamily | GO:0008270, GO:0003676 | [SSF57756(SUPERFAMILY)](http://www.ebi.ac.uk/interpro/signature/SSF57756) |
|  | no IPR | FAMILY | DNA helicase, ATP-dependent, RecQ type |  | mobidb-lite (MOBIDB_LITE) |
|  |  |  |  |  | [PTHR13710 (PANTHER)](http://www.ebi.ac.uk/interpro/signature/PTHR13710) |
|  |  |  |  |  | [PTHR13710:SF108 (PANTHER)](http://www.ebi.ac.uk/interpro/signature/PTHR13710) |
|  | no IPR | DOMAIN | Helicase, C-terminal |  | cd00046 (CDD) |
|  | no IPR | DOMAIN | Zinc finger, CCHC-type |  | [G3DSA:3.40.50.300(GENE3D)](http://www.ebi.ac.uk/interpro/signature/G3DSA:3.40.50.300) |
|  |  |  |  |  | [G3DSA:1.10.10.1460(GENE3D)](http://www.ebi.ac.uk/interpro/signature/G3DSA:1.10.10.1460) |
|  |  |  |  |  | [G3DSA:3.40.50.300(GENE3D)](http://www.ebi.ac.uk/interpro/signature/G3DSA:3.40.50.300) |
| *RsREV1* | [IPR001126](http://www.ebi.ac.uk/interpro/entry/IPR001126) | DOMAIN | UmuC domain | GO:0006281 | [PF00817 (PFAM)](http://www.ebi.ac.uk/interpro/signature/PF00817) |
|  |  |  |  |  | [PS50173(PROSITE_PROFILES)](http://www.ebi.ac.uk/interpro/signature/PS50173) |
|  | [IPR001357](http://www.ebi.ac.uk/interpro/entry/IPR001357) | DOMAIN | BRCT domain |  | [SM00292 (SMART)](http://www.ebi.ac.uk/interpro/signature/SM00292) |
|  |  |  |  |  | [PF16589 (PFAM)](http://www.ebi.ac.uk/interpro/signature/PF16589) |
|  |  |  |  |  | [PS50172(PROSITE_PROFILES)](http://www.ebi.ac.uk/interpro/signature/PS50172) |
|  |  |  |  |  | cd00027 (CDD) |
|  | [IPR012112](http://www.ebi.ac.uk/interpro/entry/IPR012112) | FAMILY | DNA repair protein Rev1 | GO:0042276, GO:0003684, GO:0006281, GO:0016779 | PIRSF036573 (PIRSF) |
|  | [IPR017961](http://www.ebi.ac.uk/interpro/entry/IPR017961) | DOMAIN | DNA polymerase, Y-family, little finger domain | GO:0006281, GO:0003684 | [PF11799 (PFAM)](http://www.ebi.ac.uk/interpro/signature/PF11799) |
|  | [IPR031991](http://www.ebi.ac.uk/interpro/entry/IPR031991) | DOMAIN | DNA repair protein Rev1, C-terminal |  | [PF16727 (PFAM)](http://www.ebi.ac.uk/interpro/signature/PF16727) |
|  | [IPR036420](http://www.ebi.ac.uk/interpro/entry/IPR036420) | HOMOLOGOUS_SUPERFAMILY | BRCT domain superfamily |  | [G3DSA:3.40.50.10190 (GENE3D)](http://www.ebi.ac.uk/interpro/signature/G3DSA:3.40.50.1019) |
|  |  |  |  |  | [SSF52113(SUPERFAMILY)](http://www.ebi.ac.uk/interpro/signature/SSF52113) |
|  | [IPR036775](http://www.ebi.ac.uk/interpro/entry/IPR036775) | HOMOLOGOUS_SUPERFAMILY | DNA polymerase, Y-family, little finger domain superfamily | GO:0003684, GO:0006281 | [G3DSA:3.30.1490.100(GENE3D)](http://www.ebi.ac.uk/interpro/signature/G3DSA:3.30.1490.100) |
|  |  |  |  |  | [SSF100879(SUPERFAMILY)](http://www.ebi.ac.uk/interpro/signature/SSF100879) |
|  | [IPR038401](http://www.ebi.ac.uk/interpro/entry/IPR038401) | HOMOLOGOUS_SUPERFAMILY | Rev1, C-terminal domain superfamily |  | [G3DSA:1.20.58.1280(GENE3D)](http://www.ebi.ac.uk/interpro/signature/G3DSA:1.20.58.1280) |
|  | no IPR | DOMAIN | BRCT domain |  | cd01701 (CDD) |
|  | no IPR | HOMOLOGOUS_SUPERFAMILY | BRCT domain superfamily |  | [G3DSA:3.30.70.270(GENE3D)](http://www.ebi.ac.uk/interpro/signature/G3DSA:3.30.70.270) |
|  |  |  |  |  | [SSF56672(SUPERFAMILY)](http://www.ebi.ac.uk/interpro/signature/SSF56672) |
|  | no IPR | DOMAIN | DNA polymerase, Y-family, little finger domain |  | [G3DSA:1.10.150.20(GENE3D)](http://www.ebi.ac.uk/interpro/signature/G3DSA:1.10.150.20) |
|  | no IPR | DOMAIN | DNA repair protein Rev1, C-terminal |  | mobidb-lite (MOBIDB_LITE) |
|  |  |  |  |  | mobidb-lite (MOBIDB_LITE) |
|  |  |  |  |  | [PTHR11076:SF12 (PANTHER)](http://www.ebi.ac.uk/interpro/signature/PTHR11076) |
|  |  |  |  |  | [PTHR11076(PANTHER)](http://www.ebi.ac.uk/interpro/signature/PTHR11076) |
|  |  |  |  |  | [PTHR11076:SF12 (PANTHER)](http://www.ebi.ac.uk/interpro/signature/PTHR11076) |
|  |  |  |  |  | [PTHR11076(PANTHER)](http://www.ebi.ac.uk/interpro/signature/PTHR11076) |
|  | no IPR | HOMOLOGOUS_SUPERFAMILY | Rev1, C-terminal domain superfamily |  | [G3DSA:2.30.40.20(GENE3D)](http://www.ebi.ac.uk/interpro/signature/G3DSA:2.30.40.20) |
| *RsRFC1* | [IPR001357](http://www.ebi.ac.uk/interpro/entry/IPR001357) | DOMAIN | BRCT domain |  | [SM00292 (SMART)](http://www.ebi.ac.uk/interpro/signature/SM00292) |
|  |  |  |  |  | [PF00533 (PFAM)](http://www.ebi.ac.uk/interpro/signature/PF00533) |
|  |  |  |  |  | [PS50172(PROSITE_PROFILES)](http://www.ebi.ac.uk/interpro/signature/PS50172) |
|  |  |  |  |  | cd00027 (CDD) |
|  | [IPR003593](http://www.ebi.ac.uk/interpro/entry/IPR003593) | DOMAIN | AAA+ ATPase domain |  | [SM00382 (SMART)](http://www.ebi.ac.uk/interpro/signature/SM00382) |
|  | [IPR003959](http://www.ebi.ac.uk/interpro/entry/IPR003959) | DOMAIN | ATPase, AAA-type, core | GO:0005524 | [PF00004 (PFAM)](http://www.ebi.ac.uk/interpro/signature/PF00004) |
|  | [IPR008921](http://www.ebi.ac.uk/interpro/entry/IPR008921) | HOMOLOGOUS_SUPERFAMILY | DNA polymerase III, clamp loader complex, gamma/delta/delta subunit, C-terminal | GO:0003677, GO:0006260 | [SSF48019(SUPERFAMILY)](http://www.ebi.ac.uk/interpro/signature/SSF48019) |
|  | [IPR012178](http://www.ebi.ac.uk/interpro/entry/IPR012178) | FAMILY | Replication factor C subunit 1 | GO:0005663, GO:0005524, GO:0003677, GO:0003689, GO:0006281, GO:0006260 | PIRSF036578 (PIRSF) |
|  | [IPR013725](http://www.ebi.ac.uk/interpro/entry/IPR013725) | DOMAIN | DNA replication factor RFC1, C-terminal | GO:0005663, GO:0006260, GO:0005524, GO:0003689 | [PF08519 (PFAM)](http://www.ebi.ac.uk/interpro/signature/PF08519) |
|  | [IPR027417](http://www.ebi.ac.uk/interpro/entry/IPR027417) | HOMOLOGOUS_SUPERFAMILY | P-loop containing nucleoside triphosphate hydrolase |  | [SSF52540(SUPERFAMILY)](http://www.ebi.ac.uk/interpro/signature/SSF52540) |
|  | [IPR036420](http://www.ebi.ac.uk/interpro/entry/IPR036420) | HOMOLOGOUS_SUPERFAMILY | BRCT domain superfamily |  | [G3DSA:3.40.50.10190 (GENE3D)](http://www.ebi.ac.uk/interpro/signature/G3DSA:3.40.50.1019) |
|  |  |  |  |  | [SSF52113(SUPERFAMILY)](http://www.ebi.ac.uk/interpro/signature/SSF52113) |
|  | no IPR | DOMAIN | ATPase, AAA-type, core |  | [G3DSA:1.20.272.10(GENE3D)](http://www.ebi.ac.uk/interpro/signature/G3DSA:1.20.272.10) |
|  | no IPR | DOMAIN | BRCT domain |  | cd00009 (CDD) |
|  | no IPR | HOMOLOGOUS_SUPERFAMILY | BRCT domain superfamily |  | [G3DSA:1.10.8.60(GENE3D)](http://www.ebi.ac.uk/interpro/signature/G3DSA:1.10.8.60) |
|  |  |  |  |  | [G3DSA:3.40.50.300(GENE3D)](http://www.ebi.ac.uk/interpro/signature/G3DSA:3.40.50.300) |
|  |  |  |  |  | mobidb-lite (MOBIDB_LITE) |
|  |  |  |  |  | mobidb-lite (MOBIDB_LITE) |
|  |  |  |  |  | mobidb-lite (MOBIDB_LITE) |
|  |  |  |  |  | mobidb-lite (MOBIDB_LITE) |
|  |  |  |  |  | mobidb-lite (MOBIDB_LITE) |
|  |  |  |  |  | mobidb-lite (MOBIDB_LITE) |
|  |  |  |  |  | [PTHR23389(PANTHER)](http://www.ebi.ac.uk/interpro/signature/PTHR23389) |
|  |  |  |  |  | [PTHR23389:SF22 (PANTHER)](http://www.ebi.ac.uk/interpro/signature/PTHR23389) |
|  |  |  |  |  | [PTHR23389:SF22 (PANTHER)](http://www.ebi.ac.uk/interpro/signature/PTHR23389) |
|  |  |  |  |  | [PTHR23389(PANTHER)](http://www.ebi.ac.uk/interpro/signature/PTHR23389) |
|  | no IPR |  |  |  | Coil (COILS) |
|  |  |  |  |  | Coil (COILS) |
| *RsRFC2* | [IPR003593](http://www.ebi.ac.uk/interpro/entry/IPR003593) | DOMAIN | AAA+ ATPase domain |  | [SM00382 (SMART)](http://www.ebi.ac.uk/interpro/signature/SM00382) |
|  | [IPR003959](http://www.ebi.ac.uk/interpro/entry/IPR003959) | DOMAIN | ATPase, AAA-type, core | GO:0005524 | [PF00004 (PFAM)](http://www.ebi.ac.uk/interpro/signature/PF00004) |
|  | [IPR008921](http://www.ebi.ac.uk/interpro/entry/IPR008921) | HOMOLOGOUS_SUPERFAMILY | DNA polymerase III, clamp loader complex, gamma/delta/delta subunit, C-terminal | GO:0003677, GO:0006260 | [SSF48019(SUPERFAMILY)](http://www.ebi.ac.uk/interpro/signature/SSF48019) |
|  | [IPR013748](http://www.ebi.ac.uk/interpro/entry/IPR013748) | DOMAIN | Replication factor C, C-terminal |  | [PF08542 (PFAM)](http://www.ebi.ac.uk/interpro/signature/PF08542) |
|  | [IPR027417](http://www.ebi.ac.uk/interpro/entry/IPR027417) | HOMOLOGOUS_SUPERFAMILY | P-loop containing nucleoside triphosphate hydrolase |  | [SSF52540(SUPERFAMILY)](http://www.ebi.ac.uk/interpro/signature/SSF52540) |
|  | no IPR | DOMAIN | ATPase, AAA-type, core |  | [G3DSA:3.40.50.300(GENE3D)](http://www.ebi.ac.uk/interpro/signature/G3DSA:3.40.50.300) |
|  |  |  |  |  | [PTHR11669(PANTHER)](http://www.ebi.ac.uk/interpro/signature/PTHR11669) |
|  |  |  |  |  | [PTHR11669:SF5 (PANTHER)](http://www.ebi.ac.uk/interpro/signature/PTHR11669) |
|  |  |  |  |  | cd00009 (CDD) |
|  | no IPR | DOMAIN | Replication factor C, C-terminal |  | [G3DSA:1.10.8.60(GENE3D)](http://www.ebi.ac.uk/interpro/signature/G3DSA:1.10.8.60) |
|  |  |  |  |  | [G3DSA:1.20.272.10(GENE3D)](http://www.ebi.ac.uk/interpro/signature/G3DSA:1.20.272.10) |
| *RsRFC3* | [IPR008921](http://www.ebi.ac.uk/interpro/entry/IPR008921) | HOMOLOGOUS_SUPERFAMILY | DNA polymerase III, clamp loader complex, gamma/delta/delta subunit, C-terminal | GO:0003677, GO:0006260 | [SSF48019(SUPERFAMILY)](http://www.ebi.ac.uk/interpro/signature/SSF48019) |
|  | [IPR013748](http://www.ebi.ac.uk/interpro/entry/IPR013748) | DOMAIN | Replication factor C, C-terminal |  | [PF08542 (PFAM)](http://www.ebi.ac.uk/interpro/signature/PF08542) |
|  | [IPR027417](http://www.ebi.ac.uk/interpro/entry/IPR027417) | HOMOLOGOUS_SUPERFAMILY | P-loop containing nucleoside triphosphate hydrolase |  | [SSF52540(SUPERFAMILY)](http://www.ebi.ac.uk/interpro/signature/SSF52540) |
|  | no IPR | DOMAIN | Replication factor C, C-terminal |  | [PF13177 (PFAM)](http://www.ebi.ac.uk/interpro/signature/PF13177) |
|  |  |  |  |  | [G3DSA:1.20.272.10(GENE3D)](http://www.ebi.ac.uk/interpro/signature/G3DSA:1.20.272.10) |
|  |  |  |  |  | [G3DSA:1.10.8.60(GENE3D)](http://www.ebi.ac.uk/interpro/signature/G3DSA:1.10.8.60) |
|  |  |  |  |  | [G3DSA:3.40.50.300(GENE3D)](http://www.ebi.ac.uk/interpro/signature/G3DSA:3.40.50.300) |
|  |  |  |  |  | [PTHR11669(PANTHER)](http://www.ebi.ac.uk/interpro/signature/PTHR11669) |
|  |  |  |  |  | [PTHR11669:SF1 (PANTHER)](http://www.ebi.ac.uk/interpro/signature/PTHR11669) |
|  |  |  |  |  | cd00009 (CDD) |
| *RsRFC4* | [IPR003593](http://www.ebi.ac.uk/interpro/entry/IPR003593) | DOMAIN | AAA+ ATPase domain |  | [SM00382 (SMART)](http://www.ebi.ac.uk/interpro/signature/SM00382) |
|  | [IPR003959](http://www.ebi.ac.uk/interpro/entry/IPR003959) | DOMAIN | ATPase, AAA-type, core | GO:0005524 | [PF00004 (PFAM)](http://www.ebi.ac.uk/interpro/signature/PF00004) |
|  | [IPR008921](http://www.ebi.ac.uk/interpro/entry/IPR008921) | HOMOLOGOUS_SUPERFAMILY | DNA polymerase III, clamp loader complex, gamma/delta/delta subunit, C-terminal | GO:0006260, GO:0003677 | [SSF48019(SUPERFAMILY)](http://www.ebi.ac.uk/interpro/signature/SSF48019) |
|  | [IPR013748](http://www.ebi.ac.uk/interpro/entry/IPR013748) | DOMAIN | Replication factor C, C-terminal |  | [PF08542 (PFAM)](http://www.ebi.ac.uk/interpro/signature/PF08542) |
|  | [IPR027417](http://www.ebi.ac.uk/interpro/entry/IPR027417) | HOMOLOGOUS_SUPERFAMILY | P-loop containing nucleoside triphosphate hydrolase |  | [SSF52540(SUPERFAMILY)](http://www.ebi.ac.uk/interpro/signature/SSF52540) |
|  | no IPR | DOMAIN | AAA+ ATPase domain |  | [G3DSA:1.10.8.60(GENE3D)](http://www.ebi.ac.uk/interpro/signature/G3DSA:1.10.8.60) |
|  |  |  |  |  | [G3DSA:3.40.50.300(GENE3D)](http://www.ebi.ac.uk/interpro/signature/G3DSA:3.40.50.300) |
|  | no IPR | DOMAIN | Replication factor C, C-terminal |  | [G3DSA:1.20.272.10(GENE3D)](http://www.ebi.ac.uk/interpro/signature/G3DSA:1.20.272.10) |
|  |  |  |  |  | mobidb-lite (MOBIDB_LITE) |
|  |  |  |  |  | [PTHR11669(PANTHER)](http://www.ebi.ac.uk/interpro/signature/PTHR11669) |
|  |  |  |  |  | [PTHR11669:SF20 (PANTHER)](http://www.ebi.ac.uk/interpro/signature/PTHR11669) |
|  |  |  |  |  | cd00009 (CDD) |
| *RsRFC5* | [IPR003593](http://www.ebi.ac.uk/interpro/entry/IPR003593) | DOMAIN | AAA+ ATPase domain |  | [SM00382 (SMART)](http://www.ebi.ac.uk/interpro/signature/SM00382) |
|  | [IPR003959](http://www.ebi.ac.uk/interpro/entry/IPR003959) | DOMAIN | ATPase, AAA-type, core | GO:0005524 | [PF00004 (PFAM)](http://www.ebi.ac.uk/interpro/signature/PF00004) |
|  | [IPR008921](http://www.ebi.ac.uk/interpro/entry/IPR008921) | HOMOLOGOUS_SUPERFAMILY | DNA polymerase III, clamp loader complex, gamma/delta/delta subunit, C-terminal | GO:0006260, GO:0003677 | [SSF48019(SUPERFAMILY)](http://www.ebi.ac.uk/interpro/signature/SSF48019) |
|  | [IPR013748](http://www.ebi.ac.uk/interpro/entry/IPR013748) | DOMAIN | Replication factor C, C-terminal |  | [PF08542 (PFAM)](http://www.ebi.ac.uk/interpro/signature/PF08542) |
|  | [IPR027417](http://www.ebi.ac.uk/interpro/entry/IPR027417) | HOMOLOGOUS_SUPERFAMILY | P-loop containing nucleoside triphosphate hydrolase |  | [SSF52540(SUPERFAMILY)](http://www.ebi.ac.uk/interpro/signature/SSF52540) |
|  | no IPR | DOMAIN | AAA+ ATPase domain |  | [G3DSA:1.20.272.10(GENE3D)](http://www.ebi.ac.uk/interpro/signature/G3DSA:1.20.272.10) |
|  |  |  |  |  | [G3DSA:1.10.8.60(GENE3D)](http://www.ebi.ac.uk/interpro/signature/G3DSA:1.10.8.60) |
|  |  |  |  |  | [G3DSA:3.40.50.300(GENE3D)](http://www.ebi.ac.uk/interpro/signature/G3DSA:3.40.50.300) |
|  | no IPR | DOMAIN | ATPase, AAA-type, core |  | [PTHR11669:SF9 (PANTHER)](http://www.ebi.ac.uk/interpro/signature/PTHR11669) |
|  |  |  |  |  | [PTHR11669(PANTHER)](http://www.ebi.ac.uk/interpro/signature/PTHR11669) |
|  |  |  |  |  | cd00009 (CDD) |
| *RsRMI1* | [IPR013894](http://www.ebi.ac.uk/interpro/entry/IPR013894) | DOMAIN | RecQ mediated genome instability protein, N-terminal |  | [PF08585 (PFAM)](http://www.ebi.ac.uk/interpro/signature/PF08585) |
|  | [IPR032199](http://www.ebi.ac.uk/interpro/entry/IPR032199) | DOMAIN | Recq-mediated genome instability protein 1, C-terminal OB-fold domain | GO:0000166 | [PF16099 (PFAM)](http://www.ebi.ac.uk/interpro/signature/PF16099) |
|  | [IPR033472](http://www.ebi.ac.uk/interpro/entry/IPR033472) | DOMAIN | RecQ mediated genome instability protein, DUF1767 |  | [SM01161 (SMART)](http://www.ebi.ac.uk/interpro/signature/SM01161) |
|  | no IPR | DOMAIN | RecQ mediated genome instability protein, DUF1767 |  | [G3DSA:2.40.50.510 (GENE3D)](http://www.ebi.ac.uk/interpro/signature/G3DSA:2.40.50.510) |
|  |  |  |  |  | [G3DSA:2.40.50.770 (GENE3D)](http://www.ebi.ac.uk/interpro/signature/G3DSA:2.40.50.770) |
|  | no IPR | DOMAIN | Recq-mediated genome instability protein 1, C-terminal OB-fold domain |  | [G3DSA:1.10.8.1020 (GENE3D)](http://www.ebi.ac.uk/interpro/signature/G3DSA:1.10.8.1020) |
|  |  |  |  |  | mobidb-lite (MOBIDB_LITE) |
|  |  |  |  |  | mobidb-lite (MOBIDB_LITE) |
|  |  |  |  |  | [PTHR14790:SF15 (PANTHER)](http://www.ebi.ac.uk/interpro/signature/PTHR14790) |
|  |  |  |  |  | [PTHR14790 (PANTHER)](http://www.ebi.ac.uk/interpro/signature/PTHR14790) |
|  | no IPR |  |  |  | Coil (COILS) |
|  |  |  |  |  | Coil (COILS) |
| *RsRMI2* | [IPR032245](http://www.ebi.ac.uk/interpro/entry/IPR032245) | FAMILY | RecQ-mediated genome instability protein 2 |  | [PF16100 (PFAM)](http://www.ebi.ac.uk/interpro/signature/PF16100) |
|  |  |  |  |  | [PTHR33962 (PANTHER)](http://www.ebi.ac.uk/interpro/signature/PTHR33962) |
|  | no IPR | FAMILY | RecQ-mediated genome instability protein 2 |  | [G3DSA:2.40.50.140 (GENE3D)](http://www.ebi.ac.uk/interpro/signature/G3DSA:2.40.50.140) |
| *RsRNF168* | [IPR001841](http://www.ebi.ac.uk/interpro/entry/IPR001841) | DOMAIN | Zinc finger, RING-type |  | [SM00184 (SMART)](http://www.ebi.ac.uk/interpro/signature/SM00184) |
|  |  |  |  |  | [PS50089 (PROSITE_PROFILES)](http://www.ebi.ac.uk/interpro/signature/PS50089) |
|  | [IPR013083](http://www.ebi.ac.uk/interpro/entry/IPR013083) | HOMOLOGOUS_SUPERFAMILY | Zinc finger, RING/FYVE/PHD-type |  | [G3DSA:3.30.40.10 (GENE3D)](http://www.ebi.ac.uk/interpro/signature/G3DSA:3.30.40.10) |
|  | no IPR | DOMAIN | Zinc finger, RING-type |  | [PF13920 (PFAM)](http://www.ebi.ac.uk/interpro/signature/PF13920) |
|  |  |  |  |  | cd16550 (CDD) |
|  |  |  |  |  | [SSF57850 (SUPERFAMILY)](http://www.ebi.ac.uk/interpro/signature/SSF57850) |
|  | no IPR | HOMOLOGOUS_SUPERFAMILY | Zinc finger, RING/FYVE/PHD-type |  | mobidb-lite (MOBIDB_LITE) |
|  |  |  |  |  | mobidb-lite (MOBIDB_LITE) |
|  |  |  |  |  | [PTHR23328 (PANTHER)](http://www.ebi.ac.uk/interpro/signature/PTHR23328) |
|  | no IPR |  |  |  | Coil (COILS) |
| *RsRNF8* | [IPR000253](http://www.ebi.ac.uk/interpro/entry/IPR000253) | DOMAIN | Forkhead-associated (FHA) domain | GO:0005515 | [SM00240 (SMART)](http://www.ebi.ac.uk/interpro/signature/SM00240) |
|  |  |  |  |  | [PF00498 (PFAM)](http://www.ebi.ac.uk/interpro/signature/PF00498) |
|  |  |  |  |  | [PS50006(PROSITE_PROFILES)](http://www.ebi.ac.uk/interpro/signature/PS50006) |
|  |  |  |  |  | cd00060 (CDD) |
|  | [IPR001841](http://www.ebi.ac.uk/interpro/entry/IPR001841) | DOMAIN | Zinc finger, RING-type |  | [SM00184 (SMART)](http://www.ebi.ac.uk/interpro/signature/SM00184) |
|  |  |  |  |  | [PS50089(PROSITE_PROFILES)](http://www.ebi.ac.uk/interpro/signature/PS50089) |
|  | [IPR001878](http://www.ebi.ac.uk/interpro/entry/IPR001878) | DOMAIN | Zinc finger, CCHC-type | GO:0003676, GO:0008270 | [PS50158(PROSITE_PROFILES)](http://www.ebi.ac.uk/interpro/signature/PS50158) |
|  | [IPR008984](http://www.ebi.ac.uk/interpro/entry/IPR008984) | HOMOLOGOUS_SUPERFAMILY | SMAD/FHA domain superfamily | GO:0005515 | [SSF49879 (SUPERFAMILY)](http://www.ebi.ac.uk/interpro/signature/SSF49879) |
|  | [IPR013083](http://www.ebi.ac.uk/interpro/entry/IPR013083) | HOMOLOGOUS_SUPERFAMILY | Zinc finger, RING/FYVE/PHD-type |  | [G3DSA:3.30.40.10 (GENE3D)](http://www.ebi.ac.uk/interpro/signature/G3DSA:3.30.40.10) |
|  | [IPR017907](http://www.ebi.ac.uk/interpro/entry/IPR017907) | CONSERVED_SITE | Zinc finger, RING-type, conserved site |  | [PS00518(PROSITE_PATTERNS)](http://www.ebi.ac.uk/interpro/signature/PS00518) |
|  | [IPR018957](http://www.ebi.ac.uk/interpro/entry/IPR018957) | DOMAIN | Zinc finger, C3HC4 RING-type | GO:0046872 | [PF00097 (PFAM)](http://www.ebi.ac.uk/interpro/signature/PF00097) |
|  | [IPR036875](http://www.ebi.ac.uk/interpro/entry/IPR036875) | HOMOLOGOUS_SUPERFAMILY | Zinc finger, CCHC-type superfamily | GO:0008270, GO:0003676 | [SSF57756 (SUPERFAMILY)](http://www.ebi.ac.uk/interpro/signature/SSF57756) |
|  | no IPR | DOMAIN | Forkhead-associated (FHA) domain |  | [G3DSA:2.60.200.20 (GENE3D)](http://www.ebi.ac.uk/interpro/signature/G3DSA:2.60.200.20) |
|  |  |  |  |  | mobidb-lite (MOBIDB_LITE) |
|  |  |  |  |  | mobidb-lite (MOBIDB_LITE) |
|  |  |  |  |  | [PTHR15067 (PANTHER)](http://www.ebi.ac.uk/interpro/signature/PTHR15067) |
|  | no IPR | HOMOLOGOUS_SUPERFAMILY | SMAD/FHA domain superfamily |  | [SSF57850 (SUPERFAMILY)](http://www.ebi.ac.uk/interpro/signature/SSF57850) |
|  | no IPR |  |  |  | Coil (COILS) |
|  |  |  |  |  | Coil (COILS) |
| *RsRPA1* | [IPR004365](http://www.ebi.ac.uk/interpro/entry/IPR004365) | DOMAIN | OB-fold nucleic acid binding domain, AA-tRNA synthetase-type | GO:0003676 | [PF01336 (PFAM)](http://www.ebi.ac.uk/interpro/signature/PF01336) |
|  | [IPR004591](http://www.ebi.ac.uk/interpro/entry/IPR004591) | FAMILY | Replication factor A protein 1 | GO:0006310, GO:0006260, GO:0003677, GO:0006281, GO:0005634 | [TIGR00617(TIGRFAM)](http://www.ebi.ac.uk/interpro/signature/TIGR00617) |
|  | [IPR007199](http://www.ebi.ac.uk/interpro/entry/IPR007199) | DOMAIN | Replication factor-A protein 1, N-terminal | GO:0003677, GO:0006260, GO:0005634 | [PF04057 (PFAM)](http://www.ebi.ac.uk/interpro/signature/PF04057) |
|  |  |  |  |  | cd04477 (CDD) |
|  | [IPR012340](http://www.ebi.ac.uk/interpro/entry/IPR012340) | HOMOLOGOUS_SUPERFAMILY | Nucleic acid-binding, OB-fold |  | [SSF50249(SUPERFAMILY)](http://www.ebi.ac.uk/interpro/signature/SSF50249) |
|  |  |  |  |  | [SSF50249(SUPERFAMILY)](http://www.ebi.ac.uk/interpro/signature/SSF50249) |
|  |  |  |  |  | [SSF50249(SUPERFAMILY)](http://www.ebi.ac.uk/interpro/signature/SSF50249) |
|  |  |  |  |  | [SSF50249(SUPERFAMILY)](http://www.ebi.ac.uk/interpro/signature/SSF50249) |
|  | [IPR013955](http://www.ebi.ac.uk/interpro/entry/IPR013955) | DOMAIN | Replication factor A, C-terminal |  | [PF08646 (PFAM)](http://www.ebi.ac.uk/interpro/signature/PF08646) |
|  |  |  |  |  | cd04476 (CDD) |
|  | [IPR031657](http://www.ebi.ac.uk/interpro/entry/IPR031657) | DOMAIN | Replication protein A, OB domain |  | [PF16900 (PFAM)](http://www.ebi.ac.uk/interpro/signature/PF16900) |
|  | no IPR | DOMAIN | OB-fold nucleic acid binding domain, AA-tRNA synthetase-type |  | [G3DSA:2.40.50.140(GENE3D)](http://www.ebi.ac.uk/interpro/signature/G3DSA:2.40.50.140) |
|  |  |  |  |  | mobidb-lite (MOBIDB_LITE) |
|  |  |  |  |  | [PTHR23273(PANTHER)](http://www.ebi.ac.uk/interpro/signature/PTHR23273) |
|  |  |  |  |  | [PTHR23273:SF4 (PANTHER)](http://www.ebi.ac.uk/interpro/signature/PTHR23273) |
|  | no IPR | FAMILY | Replication factor A protein 1 |  | [G3DSA:2.40.50.140(GENE3D)](http://www.ebi.ac.uk/interpro/signature/G3DSA:2.40.50.140) |
|  |  |  |  |  | [G3DSA:2.40.50.140(GENE3D)](http://www.ebi.ac.uk/interpro/signature/G3DSA:2.40.50.140) |
|  | no IPR | DOMAIN | Replication factor A, C-terminal |  | [G3DSA:2.40.50.140(GENE3D)](http://www.ebi.ac.uk/interpro/signature/G3DSA:2.40.50.140) |
|  | no IPR | DOMAIN | Replication factor-A protein 1, N-terminal |  | cd04474 (CDD) |
|  |  |  |  |  | cd04475 (CDD) |
| *RsRRP1* | [IPR004808](http://www.ebi.ac.uk/interpro/entry/IPR004808) | FAMILY | AP endonuclease 1 | GO:0004518, GO:0006281 | [TIGR00633 (TIGRFAM)](http://www.ebi.ac.uk/interpro/signature/TIGR00633) |
|  |  |  |  |  | [PTHR22748(PANTHER)](http://www.ebi.ac.uk/interpro/signature/PTHR22748) |
|  |  |  |  |  | [PS51435(PROSITE_PROFILES)](http://www.ebi.ac.uk/interpro/signature/PS51435) |
|  | [IPR005135](http://www.ebi.ac.uk/interpro/entry/IPR005135) | DOMAIN | Endonuclease/exonuclease/phosphatase |  | [PF03372 (PFAM)](http://www.ebi.ac.uk/interpro/signature/PF03372) |
|  | [IPR020847](http://www.ebi.ac.uk/interpro/entry/IPR020847) | BINDING_SITE | AP endonuclease 1, binding site | GO:0006281, GO:0004519, GO:0005622, GO:0003677 | [PS00726(PROSITE_PATTERNS)](http://www.ebi.ac.uk/interpro/signature/PS00726) |
|  | [IPR020848](http://www.ebi.ac.uk/interpro/entry/IPR020848) | CONSERVED_SITE | AP endonuclease 1, conserved site | GO:0004519, GO:0005622, GO:0003677, GO:0006281 | [PS00727(PROSITE_PATTERNS)](http://www.ebi.ac.uk/interpro/signature/PS00727) |
|  | [IPR036691](http://www.ebi.ac.uk/interpro/entry/IPR036691) | HOMOLOGOUS_SUPERFAMILY | Endonuclease/exonuclease/phosphatase superfamily |  | [G3DSA:3.60.10.10(GENE3D)](http://www.ebi.ac.uk/interpro/signature/G3DSA:3.60.10.10) |
|  |  |  |  |  | [SSF56219(SUPERFAMILY)](http://www.ebi.ac.uk/interpro/signature/SSF56219) |
|  | no IPR | FAMILY | AP endonuclease 1 |  | mobidb-lite (MOBIDB_LITE) |
|  |  |  |  |  | [PTHR22748:SF6 (PANTHER)](http://www.ebi.ac.uk/interpro/signature/PTHR22748) |
|  |  |  |  |  | cd09087 (CDD) |
|  | no IPR |  |  |  | [TIGR00195 (TIGRFAM)](http://www.ebi.ac.uk/interpro/signature/TIGR00195) |
| *RsRTEL1* | [IPR006555](http://www.ebi.ac.uk/interpro/entry/IPR006555) | DOMAIN | ATP-dependent helicase, C-terminal | GO:0016818, GO:0006139, GO:0003676, GO:0008026, GO:0005524 | [SM00491 (SMART)](http://www.ebi.ac.uk/interpro/signature/SM00491) |
|  |  |  |  |  | [PF13307 (PFAM)](http://www.ebi.ac.uk/interpro/signature/PF13307) |
|  | no IPR | DOMAIN | ATP-dependent helicase, C-terminal |  | [G3DSA:3.40.50.300(GENE3D)](http://www.ebi.ac.uk/interpro/signature/G3DSA:3.40.50.300) |
|  |  |  |  |  | mobidb-lite (MOBIDB_LITE) |
|  |  |  |  |  | [PTHR11472 (PANTHER)](http://www.ebi.ac.uk/interpro/signature/PTHR11472) |
|  |  |  |  |  | [PTHR11472:SF34 (PANTHER)](http://www.ebi.ac.uk/interpro/signature/PTHR11472) |
| *RsSHPRH* | [IPR001650](http://www.ebi.ac.uk/interpro/entry/IPR001650) | DOMAIN | Helicase, C-terminal |  | [SM00490 (SMART)](http://www.ebi.ac.uk/interpro/signature/SM00490) |
|  |  |  |  |  | [PF00271 (PFAM)](http://www.ebi.ac.uk/interpro/signature/PF00271) |
|  |  |  |  |  | [PS51194 (PROSITE_PROFILES)](http://www.ebi.ac.uk/interpro/signature/PS51194) |
|  |  |  |  |  | cd00079 (CDD) |
|  | [IPR001841](http://www.ebi.ac.uk/interpro/entry/IPR001841) | DOMAIN | Zinc finger, RING-type |  | [SM00184 (SMART)](http://www.ebi.ac.uk/interpro/signature/SM00184) |
|  |  |  |  |  | [PS50089 (PROSITE_PROFILES)](http://www.ebi.ac.uk/interpro/signature/PS50089) |
|  | [IPR013083](http://www.ebi.ac.uk/interpro/entry/IPR013083) | HOMOLOGOUS_SUPERFAMILY | Zinc finger, RING/FYVE/PHD-type |  | [G3DSA:3.30.40.10 (GENE3D)](http://www.ebi.ac.uk/interpro/signature/G3DSA:3.30.40.10) |
|  | [IPR017907](http://www.ebi.ac.uk/interpro/entry/IPR017907) | CONSERVED_SITE | Zinc finger, RING-type, conserved site |  | [PS00518 (PROSITE_PATTERNS)](http://www.ebi.ac.uk/interpro/signature/PS00518) |
|  | [IPR027417](http://www.ebi.ac.uk/interpro/entry/IPR027417) | HOMOLOGOUS_SUPERFAMILY | P-loop containing nucleoside triphosphate hydrolase |  | [SSF52540 (SUPERFAMILY)](http://www.ebi.ac.uk/interpro/signature/SSF52540) |
|  | no IPR | DOMAIN | Helicase, C-terminal |  | [PTHR10799:SF948 (PANTHER)](http://www.ebi.ac.uk/interpro/signature/PTHR10799) |
|  |  |  |  |  | [PTHR10799 (PANTHER)](http://www.ebi.ac.uk/interpro/signature/PTHR10799) |
|  |  |  |  |  | cd16569 (CDD) |
|  | no IPR | HOMOLOGOUS_SUPERFAMILY | P-loop containing nucleoside triphosphate hydrolase |  | [SSF57850 (SUPERFAMILY)](http://www.ebi.ac.uk/interpro/signature/SSF57850) |
|  | no IPR | HOMOLOGOUS_SUPERFAMILY | Zinc finger, RING/FYVE/PHD-type |  | [G3DSA:3.40.50.300 (GENE3D)](http://www.ebi.ac.uk/interpro/signature/G3DSA:3.40.50.300) |
|  |  |  |  |  | [PF13920 (PFAM)](http://www.ebi.ac.uk/interpro/signature/PF13920) |
|  | no IPR |  |  |  | Coil (COILS) |
| *RsSLX1* | [IPR000305](http://www.ebi.ac.uk/interpro/entry/IPR000305) | DOMAIN | GIY-YIG endonuclease |  | [PF01541 (PFAM)](http://www.ebi.ac.uk/interpro/signature/PF01541) |
|  |  |  |  |  | [PS50164(PROSITE_PROFILES)](http://www.ebi.ac.uk/interpro/signature/PS50164) |
|  | [IPR013083](http://www.ebi.ac.uk/interpro/entry/IPR013083) | HOMOLOGOUS_SUPERFAMILY | Zinc finger, RING/FYVE/PHD-type |  | [G3DSA:3.30.40.10(GENE3D)](http://www.ebi.ac.uk/interpro/signature/G3DSA:3.30.40.10) |
|  | [IPR027520](http://www.ebi.ac.uk/interpro/entry/IPR027520) | FAMILY | Structure-specific endonuclease subunit Slx1 | GO:0033557, GO:0017108, GO:0006281 | MF_03100 (HAMAP) |
|  | [IPR035901](http://www.ebi.ac.uk/interpro/entry/IPR035901) | HOMOLOGOUS_SUPERFAMILY | GIY-YIG endonuclease superfamily |  | [G3DSA:3.40.1440.10(GENE3D)](http://www.ebi.ac.uk/interpro/signature/G3DSA:3.40.1440.10) |
|  |  |  |  |  | [SSF82771(SUPERFAMILY)](http://www.ebi.ac.uk/interpro/signature/SSF82771) |
|  | no IPR | FAMILY | Structure-specific endonuclease subunit Slx1 |  | cd10455 (CDD) |
|  | no IPR | HOMOLOGOUS_SUPERFAMILY | Zinc finger, RING/FYVE/PHD-type |  | [PTHR20208:SF10 (PANTHER)](http://www.ebi.ac.uk/interpro/signature/PTHR20208) |
|  |  |  |  |  | [PTHR20208 (PANTHER)](http://www.ebi.ac.uk/interpro/signature/PTHR20208) |
| *RsSLX4* | [IPR000210](http://www.ebi.ac.uk/interpro/entry/IPR000210) | DOMAIN | BTB/POZ domain | GO:0005515 | [PF00651 (PFAM)](http://www.ebi.ac.uk/interpro/signature/PF00651) |
|  |  |  |  |  | [PS50097(PROSITE_PROFILES)](http://www.ebi.ac.uk/interpro/signature/PS50097) |
|  | [IPR011333](http://www.ebi.ac.uk/interpro/entry/IPR011333) | HOMOLOGOUS_SUPERFAMILY | SKP1/BTB/POZ domain superfamily |  | [SSF54695(SUPERFAMILY)](http://www.ebi.ac.uk/interpro/signature/SSF54695) |
|  | [IPR018574](http://www.ebi.ac.uk/interpro/entry/IPR018574) | FAMILY | Structure-specific endonuclease subunit Slx4 | GO:0005634, GO:0006281, GO:0006260, GO:0033557, GO:0017108 | [PF09494 (PFAM)](http://www.ebi.ac.uk/interpro/signature/PF09494) |
|  | no IPR | DOMAIN | BTB/POZ domain |  | mobidb-lite (MOBIDB_LITE) |
|  |  |  |  |  | mobidb-lite (MOBIDB_LITE) |
|  |  |  |  |  | mobidb-lite (MOBIDB_LITE) |
|  |  |  |  |  | mobidb-lite (MOBIDB_LITE) |
|  |  |  |  |  | mobidb-lite (MOBIDB_LITE) |
|  |  |  |  |  | mobidb-lite (MOBIDB_LITE) |
|  |  |  |  |  | mobidb-lite (MOBIDB_LITE) |
|  |  |  |  |  | mobidb-lite (MOBIDB_LITE) |
|  |  |  |  |  | [PTHR21541(PANTHER)](http://www.ebi.ac.uk/interpro/signature/PTHR21541) |
|  | no IPR |  |  |  | [G3DSA:3.30.710.10(GENE3D)](http://www.ebi.ac.uk/interpro/signature/G3DSA:3.30.710.10) |
| *RsSMARCAL1* | [IPR000330](http://www.ebi.ac.uk/interpro/entry/IPR000330) | DOMAIN | SNF2-related, N-terminal domain | GO:0005524 | [PF00176 (PFAM)](http://www.ebi.ac.uk/interpro/signature/PF00176) |
|  | [IPR001650](http://www.ebi.ac.uk/interpro/entry/IPR001650) | DOMAIN | Helicase, C-terminal |  | [SM00490 (SMART)](http://www.ebi.ac.uk/interpro/signature/SM00490) |
|  |  |  |  |  | [PF00271 (PFAM)](http://www.ebi.ac.uk/interpro/signature/PF00271) |
|  |  |  |  |  | [PS51194(PROSITE_PROFILES)](http://www.ebi.ac.uk/interpro/signature/PS51194) |
|  |  |  |  |  | cd00079 (CDD) |
|  | [IPR010003](http://www.ebi.ac.uk/interpro/entry/IPR010003) | DOMAIN | HARP domain |  | [PF07443 (PFAM)](http://www.ebi.ac.uk/interpro/signature/PF07443) |
|  |  |  |  |  | [PS51467(PROSITE_PROFILES)](http://www.ebi.ac.uk/interpro/signature/PS51467) |
|  | [IPR014001](http://www.ebi.ac.uk/interpro/entry/IPR014001) | DOMAIN | Helicase superfamily 1/2, ATP-binding domain |  | [SM00487 (SMART)](http://www.ebi.ac.uk/interpro/signature/SM00487) |
|  |  |  |  |  | [PS51192(PROSITE_PROFILES)](http://www.ebi.ac.uk/interpro/signature/PS51192) |
|  | [IPR027417](http://www.ebi.ac.uk/interpro/entry/IPR027417) | HOMOLOGOUS_SUPERFAMILY | P-loop containing nucleoside triphosphate hydrolase |  | [SSF52540(SUPERFAMILY)](http://www.ebi.ac.uk/interpro/signature/SSF52540) |
|  |  |  |  |  | [SSF52540(SUPERFAMILY)](http://www.ebi.ac.uk/interpro/signature/SSF52540) |
|  | [IPR030101](http://www.ebi.ac.uk/interpro/entry/IPR030101) | FAMILY | SWI/SNF-related matrix-associated actin-dependent regulator of chromatin subfamily A-like protein 1 | GO:0036310, GO:0006281, GO:0031297 | [PTHR10799:SF731 (PANTHER)](http://www.ebi.ac.uk/interpro/signature/PTHR10799) |
|  | [IPR038718](http://www.ebi.ac.uk/interpro/entry/IPR038718) | HOMOLOGOUS_SUPERFAMILY | SNF2-like, N-terminal domain superfamily |  | [G3DSA:3.40.50.10810 (GENE3D)](http://www.ebi.ac.uk/interpro/signature/G3DSA:3.40.50.1081) |
|  | no IPR | DOMAIN | HARP domain |  | [PTHR10799(PANTHER)](http://www.ebi.ac.uk/interpro/signature/PTHR10799) |
|  | no IPR | DOMAIN | Helicase superfamily 1/2, ATP-binding domain |  | cd00046 (CDD) |
|  | no IPR | DOMAIN | SNF2-related, N-terminal domain |  | [G3DSA:3.40.50.300(GENE3D)](http://www.ebi.ac.uk/interpro/signature/G3DSA:3.40.50.300) |
|  | no IPR |  |  |  | Coil (COILS) |
| *RsSSBP* | [IPR000424](http://www.ebi.ac.uk/interpro/entry/IPR000424) | FAMILY | Primosome PriB/single-strand DNA-binding | GO:0003697 | [PF00436 (PFAM)](http://www.ebi.ac.uk/interpro/signature/PF00436) |
|  |  |  |  |  | [PTHR10302 (PANTHER)](http://www.ebi.ac.uk/interpro/signature/PTHR10302) |
|  |  |  |  |  | [PS50935 (PROSITE_PROFILES)](http://www.ebi.ac.uk/interpro/signature/PS50935) |
|  |  |  |  |  | cd04496 (CDD) |
|  | [IPR011344](http://www.ebi.ac.uk/interpro/entry/IPR011344) | FAMILY | Single-stranded DNA-binding protein | GO:0006260, GO:0003697 | [TIGR00621 (TIGRFAM)](http://www.ebi.ac.uk/interpro/signature/TIGR00621) |
|  |  |  |  |  | [PTHR10302:SF0 (PANTHER)](http://www.ebi.ac.uk/interpro/signature/PTHR10302) |
|  |  |  |  |  | MF_00984 (HAMAP) |
|  | [IPR012340](http://www.ebi.ac.uk/interpro/entry/IPR012340) | HOMOLOGOUS_SUPERFAMILY | Nucleic acid-binding, OB-fold |  | [SSF50249 (SUPERFAMILY)](http://www.ebi.ac.uk/interpro/signature/SSF50249) |
|  | no IPR | FAMILY | Primosome PriB/single-strand DNA-binding |  | SignalP-TM (SIGNALP_GRAM_POSITIVE) |
|  | no IPR | FAMILY | Single-stranded DNA-binding protein |  | PIRSF002070 (PIRSF) |
|  |  |  |  |  | SIGNAL_PEPTIDE_N_REGION (PHOBIUS) |
|  |  |  |  |  | NON_CYTOPLASMIC_DOMAIN (PHOBIUS) |
|  |  |  |  |  | SIGNAL_PEPTIDE_H_REGION (PHOBIUS) |
|  |  |  |  |  | SIGNAL_PEPTIDE_C_REGION (PHOBIUS) |
|  |  |  |  |  | SIGNAL_PEPTIDE (PHOBIUS) |
|  | no IPR |  |  |  | [G3DSA:2.40.50.140 (GENE3D)](http://www.ebi.ac.uk/interpro/signature/G3DSA:2.40.50.140) |
| *RsSSRP1* | [IPR000969](http://www.ebi.ac.uk/interpro/entry/IPR000969) | FAMILY | Structure-specific recognition protein | GO:0005634, GO:0003677 | [PR00887 (PRINTS)](http://www.ebi.ac.uk/interpro/signature/PR00887) |
|  | [IPR009071](http://www.ebi.ac.uk/interpro/entry/IPR009071) | DOMAIN | High mobility group box domain |  | [SM00398 (SMART)](http://www.ebi.ac.uk/interpro/signature/SM00398) |
|  |  |  |  |  | [PF00505 (PFAM)](http://www.ebi.ac.uk/interpro/signature/PF00505) |
|  |  |  |  |  | [PS50118(PROSITE_PROFILES)](http://www.ebi.ac.uk/interpro/signature/PS50118) |
|  | [IPR011993](http://www.ebi.ac.uk/interpro/entry/IPR011993) | HOMOLOGOUS_SUPERFAMILY | PH-like domain superfamily |  | [G3DSA:2.30.29.30(GENE3D)](http://www.ebi.ac.uk/interpro/signature/G3DSA:2.30.29.30) |
|  |  |  |  |  | [G3DSA:2.30.29.30(GENE3D)](http://www.ebi.ac.uk/interpro/signature/G3DSA:2.30.29.30) |
|  | [IPR013719](http://www.ebi.ac.uk/interpro/entry/IPR013719) | DOMAIN | Domain of unknown function DUF1747 |  | [SM01287 (SMART)](http://www.ebi.ac.uk/interpro/signature/SM01287) |
|  |  |  |  |  | [PF08512 (PFAM)](http://www.ebi.ac.uk/interpro/signature/PF08512) |
|  | [IPR024954](http://www.ebi.ac.uk/interpro/entry/IPR024954) | DOMAIN | SSRP1 domain |  | [PF03531 (PFAM)](http://www.ebi.ac.uk/interpro/signature/PF03531) |
|  | [IPR035417](http://www.ebi.ac.uk/interpro/entry/IPR035417) | DOMAIN | FACT complex subunit POB3-like, N-terminal PH domain |  | [PF17292 (PFAM)](http://www.ebi.ac.uk/interpro/signature/PF17292) |
|  | [IPR036910](http://www.ebi.ac.uk/interpro/entry/IPR036910) | HOMOLOGOUS_SUPERFAMILY | High mobility group box domain superfamily |  | [G3DSA:1.10.30.10(GENE3D)](http://www.ebi.ac.uk/interpro/signature/G3DSA:1.10.30.10) |
|  |  |  |  |  | [SSF47095(SUPERFAMILY)](http://www.ebi.ac.uk/interpro/signature/SSF47095) |
|  | [IPR038167](http://www.ebi.ac.uk/interpro/entry/IPR038167) | HOMOLOGOUS_SUPERFAMILY | SSRP1 domain superfamily |  | [G3DSA:2.30.29.220(GENE3D)](http://www.ebi.ac.uk/interpro/signature/G3DSA:2.30.29.220) |
|  | no IPR | DOMAIN | High mobility group box domain |  | cd13230 (CDD) |
|  |  |  |  |  | cd01390 (CDD) |
|  |  |  |  |  | cd13231 (CDD) |
|  | no IPR | HOMOLOGOUS_SUPERFAMILY | High mobility group box domain superfamily |  | [SSF50729(SUPERFAMILY)](http://www.ebi.ac.uk/interpro/signature/SSF50729) |
|  | no IPR | HOMOLOGOUS_SUPERFAMILY | PH-like domain superfamily |  | [G3DSA:2.30.29.150(GENE3D)](http://www.ebi.ac.uk/interpro/signature/G3DSA:2.30.29.150) |
|  | no IPR | DOMAIN | SSRP1 domain |  | mobidb-lite (MOBIDB_LITE) |
|  |  |  |  |  | mobidb-lite (MOBIDB_LITE) |
|  |  |  |  |  | [PTHR13711:SF204 (PANTHER)](http://www.ebi.ac.uk/interpro/signature/PTHR13711) |
|  |  |  |  |  | [PTHR13711 (PANTHER)](http://www.ebi.ac.uk/interpro/signature/PTHR13711) |
|  | no IPR |  |  |  | Coil (COILS) |
| *RsTAOK1* | [IPR000719](http://www.ebi.ac.uk/interpro/entry/IPR000719) | DOMAIN | Protein kinase domain | GO:0004672, GO:0005524, GO:0006468 | [PF00069 (PFAM)](http://www.ebi.ac.uk/interpro/signature/PF00069) |
|  |  |  |  |  | [PS50011(PROSITE_PROFILES)](http://www.ebi.ac.uk/interpro/signature/PS50011) |
|  | [IPR011009](http://www.ebi.ac.uk/interpro/entry/IPR011009) | HOMOLOGOUS_SUPERFAMILY | Protein kinase-like domain superfamily |  | [SSF56112(SUPERFAMILY)](http://www.ebi.ac.uk/interpro/signature/SSF56112) |
|  | no IPR | DOMAIN | Protein kinase domain |  | [G3DSA:1.10.510.10(GENE3D)](http://www.ebi.ac.uk/interpro/signature/G3DSA:1.10.510.10) |
|  |  |  |  |  | mobidb-lite (MOBIDB_LITE) |
|  |  |  |  |  | mobidb-lite (MOBIDB_LITE) |
|  |  |  |  |  | mobidb-lite (MOBIDB_LITE) |
|  |  |  |  |  | mobidb-lite (MOBIDB_LITE) |
|  |  |  |  |  | [PTHR24361 (PANTHER)](http://www.ebi.ac.uk/interpro/signature/PTHR24361) |
|  |  |  |  |  | [PTHR24361:SF189 (PANTHER)](http://www.ebi.ac.uk/interpro/signature/PTHR24361) |
|  | no IPR |  |  |  | Coil (COILS) |
|  |  |  |  |  | Coil (COILS) |
|  |  |  |  |  | Coil (COILS) |
|  |  |  |  |  | Coil (COILS) |
| *RsTIM* | [IPR006906](http://www.ebi.ac.uk/interpro/entry/IPR006906) | DOMAIN | Timeless protein |  | [PF04821 (PFAM)](http://www.ebi.ac.uk/interpro/signature/PF04821) |
|  | no IPR | DOMAIN | Timeless protein |  | mobidb-lite (MOBIDB_LITE) |
|  |  |  |  |  | [PTHR22940:SF4 (PANTHER)](http://www.ebi.ac.uk/interpro/signature/PTHR22940) |
|  |  |  |  |  | [PTHR22940 (PANTHER)](http://www.ebi.ac.uk/interpro/signature/PTHR22940) |
| *RsTOPBP1* | [IPR001357](http://www.ebi.ac.uk/interpro/entry/IPR001357) | DOMAIN | BRCT domain |  | [SM00292 (SMART)](http://www.ebi.ac.uk/interpro/signature/SM00292) |
|  |  |  |  |  | [PF00533 (PFAM)](http://www.ebi.ac.uk/interpro/signature/PF00533) |
|  |  |  |  |  | [PF16589 (PFAM)](http://www.ebi.ac.uk/interpro/signature/PF16589) |
|  |  |  |  |  | [PF12738 (PFAM)](http://www.ebi.ac.uk/interpro/signature/PF12738) |
|  |  |  |  |  | [PS50172 (PROSITE_PROFILES)](http://www.ebi.ac.uk/interpro/signature/PS50172) |
|  |  |  |  |  | [PS50172 (PROSITE_PROFILES)](http://www.ebi.ac.uk/interpro/signature/PS50172) |
|  |  |  |  |  | [PS50172 (PROSITE_PROFILES)](http://www.ebi.ac.uk/interpro/signature/PS50172) |
|  |  |  |  |  | [PS50172 (PROSITE_PROFILES)](http://www.ebi.ac.uk/interpro/signature/PS50172) |
|  |  |  |  |  | [PS50172 (PROSITE_PROFILES)](http://www.ebi.ac.uk/interpro/signature/PS50172) |
|  |  |  |  |  | [PS50172 (PROSITE_PROFILES)](http://www.ebi.ac.uk/interpro/signature/PS50172) |
|  |  |  |  |  | cd00027 (CDD) |
|  |  |  |  |  | cd00027 (CDD) |
|  |  |  |  |  | cd00027 (CDD) |
|  |  |  |  |  | cd00027 (CDD) |
|  |  |  |  |  | cd00027 (CDD) |
|  |  |  |  |  | cd00027 (CDD) |
|  | [IPR036420](http://www.ebi.ac.uk/interpro/entry/IPR036420) | HOMOLOGOUS_SUPERFAMILY | BRCT domain superfamily |  | [G3DSA:3.40.50.10190 (GENE3D)](http://www.ebi.ac.uk/interpro/signature/G3DSA:3.40.50.1019) |
|  |  |  |  |  | [G3DSA:3.40.50.10190 (GENE3D)](http://www.ebi.ac.uk/interpro/signature/G3DSA:3.40.50.1019) |
|  |  |  |  |  | [G3DSA:3.40.50.10190 (GENE3D)](http://www.ebi.ac.uk/interpro/signature/G3DSA:3.40.50.1019) |
|  |  |  |  |  | [G3DSA:3.40.50.10190 (GENE3D)](http://www.ebi.ac.uk/interpro/signature/G3DSA:3.40.50.1019) |
|  |  |  |  |  | [G3DSA:3.40.50.10190 (GENE3D)](http://www.ebi.ac.uk/interpro/signature/G3DSA:3.40.50.1019) |
|  |  |  |  |  | [G3DSA:3.40.50.10190 (GENE3D)](http://www.ebi.ac.uk/interpro/signature/G3DSA:3.40.50.1019) |
|  |  |  |  |  | [G3DSA:3.40.50.10190 (GENE3D)](http://www.ebi.ac.uk/interpro/signature/G3DSA:3.40.50.1019) |
|  |  |  |  |  | [G3DSA:3.40.50.10190 (GENE3D)](http://www.ebi.ac.uk/interpro/signature/G3DSA:3.40.50.1019) |
|  |  |  |  |  | [SSF52113 (SUPERFAMILY)](http://www.ebi.ac.uk/interpro/signature/SSF52113) |
|  |  |  |  |  | [SSF52113 (SUPERFAMILY)](http://www.ebi.ac.uk/interpro/signature/SSF52113) |
|  |  |  |  |  | [SSF52113 (SUPERFAMILY)](http://www.ebi.ac.uk/interpro/signature/SSF52113) |
|  |  |  |  |  | [SSF52113 (SUPERFAMILY)](http://www.ebi.ac.uk/interpro/signature/SSF52113) |
|  |  |  |  |  | [SSF52113 (SUPERFAMILY)](http://www.ebi.ac.uk/interpro/signature/SSF52113) |
|  |  |  |  |  | [SSF52113 (SUPERFAMILY)](http://www.ebi.ac.uk/interpro/signature/SSF52113) |
|  | no IPR | DOMAIN | BRCT domain |  | mobidb-lite (MOBIDB_LITE) |
|  |  |  |  |  | mobidb-lite (MOBIDB_LITE) |
|  |  |  |  |  | mobidb-lite (MOBIDB_LITE) |
|  |  |  |  |  | mobidb-lite (MOBIDB_LITE) |
|  |  |  |  |  | mobidb-lite (MOBIDB_LITE) |
|  |  |  |  |  | [PTHR13561 (PANTHER)](http://www.ebi.ac.uk/interpro/signature/PTHR13561) |
|  |  |  |  |  | [PTHR13561:SF20 (PANTHER)](http://www.ebi.ac.uk/interpro/signature/PTHR13561) |
|  |  |  |  |  | [PTHR13561 (PANTHER)](http://www.ebi.ac.uk/interpro/signature/PTHR13561) |
|  |  |  |  |  | [PTHR13561:SF20 (PANTHER)](http://www.ebi.ac.uk/interpro/signature/PTHR13561) |
| *RsTP53* | [IPR002117](http://www.ebi.ac.uk/interpro/entry/IPR002117) | FAMILY | p53 tumour suppressor family | GO:0006915, GO:0006355, GO:0003700, GO:0005634, GO:0003677 | [PR00386 (PRINTS)](http://www.ebi.ac.uk/interpro/signature/PR00386) |
|  |  |  |  |  | [PTHR11447(PANTHER)](http://www.ebi.ac.uk/interpro/signature/PTHR11447) |
|  | [IPR008967](http://www.ebi.ac.uk/interpro/entry/IPR008967) | HOMOLOGOUS_SUPERFAMILY | p53-like transcription factor, DNA-binding | GO:0003700, GO:0006355 | [SSF49417(SUPERFAMILY)](http://www.ebi.ac.uk/interpro/signature/SSF49417) |
|  | [IPR011615](http://www.ebi.ac.uk/interpro/entry/IPR011615) | DOMAIN | p53, DNA-binding domain | GO:0044212 | [PF00870 (PFAM)](http://www.ebi.ac.uk/interpro/signature/PF00870) |
|  |  |  |  |  | cd08367 (CDD) |
|  | [IPR012346](http://www.ebi.ac.uk/interpro/entry/IPR012346) | HOMOLOGOUS_SUPERFAMILY | p53/RUNT-type transcription factor, DNA-binding domain superfamily | GO:0006355, GO:0003700, GO:0005634, GO:0003677 | [G3DSA:2.60.40.720(GENE3D)](http://www.ebi.ac.uk/interpro/signature/G3DSA:2.60.40.720) |
|  | no IPR | DOMAIN | p53, DNA-binding domain |  | mobidb-lite (MOBIDB_LITE) |
|  |  |  |  |  | [PTHR11447:SF16 (PANTHER)](http://www.ebi.ac.uk/interpro/signature/PTHR11447) |
| *RsTP53BP1* | [IPR001357](http://www.ebi.ac.uk/interpro/entry/IPR001357) | DOMAIN | BRCT domain |  | [PS50172(PROSITE_PROFILES)](http://www.ebi.ac.uk/interpro/signature/PS50172) |
|  |  |  |  |  | [PS50172(PROSITE_PROFILES)](http://www.ebi.ac.uk/interpro/signature/PS50172) |
|  |  |  |  |  | cd00027 (CDD) |
|  | [IPR015125](http://www.ebi.ac.uk/interpro/entry/IPR015125) | DOMAIN | Tumour suppressor p53-binding protein-1 Tudor domain |  | [PF09038 (PFAM)](http://www.ebi.ac.uk/interpro/signature/PF09038) |
|  | [IPR036420](http://www.ebi.ac.uk/interpro/entry/IPR036420) | HOMOLOGOUS_SUPERFAMILY | BRCT domain superfamily |  | [G3DSA:3.40.50.10190 (GENE3D)](http://www.ebi.ac.uk/interpro/signature/G3DSA:3.40.50.1019) |
|  |  |  |  |  | [G3DSA:3.40.50.10190 (GENE3D)](http://www.ebi.ac.uk/interpro/signature/G3DSA:3.40.50.1019) |
|  |  |  |  |  | [SSF52113 (SUPERFAMILY)](http://www.ebi.ac.uk/interpro/signature/SSF52113) |
|  |  |  |  |  | [SSF52113 (SUPERFAMILY)](http://www.ebi.ac.uk/interpro/signature/SSF52113) |
|  | no IPR | DOMAIN | BRCT domain |  | cd04508 (CDD) |
|  | no IPR | HOMOLOGOUS_SUPERFAMILY | BRCT domain superfamily |  | [G3DSA:2.30.30.140 (GENE3D)](http://www.ebi.ac.uk/interpro/signature/G3DSA:2.30.30.140) |
|  |  |  |  |  | mobidb-lite (MOBIDB_LITE) |
|  |  |  |  |  | mobidb-lite (MOBIDB_LITE) |
|  |  |  |  |  | mobidb-lite (MOBIDB_LITE) |
|  |  |  |  |  | mobidb-lite (MOBIDB_LITE) |
|  |  |  |  |  | mobidb-lite (MOBIDB_LITE) |
|  |  |  |  |  | mobidb-lite (MOBIDB_LITE) |
|  |  |  |  |  | mobidb-lite (MOBIDB_LITE) |
|  |  |  |  |  | mobidb-lite (MOBIDB_LITE) |
|  |  |  |  |  | mobidb-lite (MOBIDB_LITE) |
|  |  |  |  |  | mobidb-lite (MOBIDB_LITE) |
|  |  |  |  |  | mobidb-lite (MOBIDB_LITE) |
|  |  |  |  |  | mobidb-lite (MOBIDB_LITE) |
|  |  |  |  |  | [PTHR15321 (PANTHER)](http://www.ebi.ac.uk/interpro/signature/PTHR15321) |
|  |  |  |  |  | [SSF63748 (SUPERFAMILY)](http://www.ebi.ac.uk/interpro/signature/SSF63748) |
| *RsUBC13* | [IPR000608](http://www.ebi.ac.uk/interpro/entry/IPR000608) | DOMAIN | Ubiquitin-conjugating enzyme E2 |  | [PF00179 (PFAM)](http://www.ebi.ac.uk/interpro/signature/PF00179) |
|  |  |  |  |  | [PS50127 (PROSITE_PROFILES)](http://www.ebi.ac.uk/interpro/signature/PS50127) |
|  |  |  |  |  | cd00195 (CDD) |
|  | [IPR016135](http://www.ebi.ac.uk/interpro/entry/IPR016135) | HOMOLOGOUS_SUPERFAMILY | Ubiquitin-conjugating enzyme/RWD-like |  | [G3DSA:3.10.110.10 (GENE3D)](http://www.ebi.ac.uk/interpro/signature/G3DSA:3.10.110.10) |
|  |  |  |  |  | [SSF54495 (SUPERFAMILY)](http://www.ebi.ac.uk/interpro/signature/SSF54495) |
|  | [IPR023313](http://www.ebi.ac.uk/interpro/entry/IPR023313) | ACTIVE_SITE | Ubiquitin-conjugating enzyme, active site |  | [PS00183 (PROSITE_PATTERNS)](http://www.ebi.ac.uk/interpro/signature/PS00183) |
|  | no IPR | DOMAIN | Ubiquitin-conjugating enzyme E2 |  | [PTHR43902 (PANTHER)](http://www.ebi.ac.uk/interpro/signature/PTHR43902) |
|  |  |  |  |  | [PTHR43902:SF5 (PANTHER)](http://www.ebi.ac.uk/interpro/signature/PTHR43902) |
|  | no IPR |  |  |  | [SM00212 (SMART)](http://www.ebi.ac.uk/interpro/signature/SM00212) |
| *RsUBC9* | [IPR000608](http://www.ebi.ac.uk/interpro/entry/IPR000608) | DOMAIN | Ubiquitin-conjugating enzyme E2 |  | [PF00179 (PFAM)](http://www.ebi.ac.uk/interpro/signature/PF00179) |
|  |  |  |  |  | [PS50127 (PROSITE_PROFILES)](http://www.ebi.ac.uk/interpro/signature/PS50127) |
|  |  |  |  |  | cd00195 (CDD) |
|  | [IPR016135](http://www.ebi.ac.uk/interpro/entry/IPR016135) | HOMOLOGOUS_SUPERFAMILY | Ubiquitin-conjugating enzyme/RWD-like |  | [G3DSA:3.10.110.10 (GENE3D)](http://www.ebi.ac.uk/interpro/signature/G3DSA:3.10.110.10) |
|  |  |  |  |  | [SSF54495 (SUPERFAMILY)](http://www.ebi.ac.uk/interpro/signature/SSF54495) |
|  | [IPR023313](http://www.ebi.ac.uk/interpro/entry/IPR023313) | ACTIVE_SITE | Ubiquitin-conjugating enzyme, active site |  | [PS00183 (PROSITE_PATTERNS)](http://www.ebi.ac.uk/interpro/signature/PS00183) |
|  | [IPR027230](http://www.ebi.ac.uk/interpro/entry/IPR027230) | FAMILY | SUMO-conjugating enzyme Ubc9 | GO:0019789 | [PTHR43927 (PANTHER)](http://www.ebi.ac.uk/interpro/signature/PTHR43927) |
|  | no IPR | DOMAIN | Ubiquitin-conjugating enzyme E2 |  | [PTHR43927:SF1 (PANTHER)](http://www.ebi.ac.uk/interpro/signature/PTHR43927) |
|  | no IPR |  |  |  | [SM00212 (SMART)](http://www.ebi.ac.uk/interpro/signature/SM00212) |
| *RsXPA* | [IPR000465](http://www.ebi.ac.uk/interpro/entry/IPR000465) | FAMILY | XPA | GO:0003684, GO:0005634, GO:0006289 | [TIGR00598 (TIGRFAM)](http://www.ebi.ac.uk/interpro/signature/TIGR00598) |
|  |  |  |  |  | [PTHR10142 (PANTHER)](http://www.ebi.ac.uk/interpro/signature/PTHR10142) |
|  | [IPR009061](http://www.ebi.ac.uk/interpro/entry/IPR009061) | HOMOLOGOUS_SUPERFAMILY | Putative DNA-binding domain superfamily |  | [SSF46955(SUPERFAMILY)](http://www.ebi.ac.uk/interpro/signature/SSF46955) |
|  | [IPR022652](http://www.ebi.ac.uk/interpro/entry/IPR022652) | CONSERVED_SITE | Zinc finger, XPA-type, conserved site |  | [PF01286 (PFAM)](http://www.ebi.ac.uk/interpro/signature/PF01286) |
|  |  |  |  |  | [PS00752(PROSITE_PATTERNS)](http://www.ebi.ac.uk/interpro/signature/PS00752) |
|  | [IPR022656](http://www.ebi.ac.uk/interpro/entry/IPR022656) | DOMAIN | XPA, C-terminal |  | [PF05181 (PFAM)](http://www.ebi.ac.uk/interpro/signature/PF05181) |
|  | [IPR037129](http://www.ebi.ac.uk/interpro/entry/IPR037129) | HOMOLOGOUS_SUPERFAMILY | XPA domain superfamily |  | [G3DSA:3.90.530.10(GENE3D)](http://www.ebi.ac.uk/interpro/signature/G3DSA:3.90.530.10) |
|  | no IPR | HOMOLOGOUS_SUPERFAMILY | Putative DNA-binding domain superfamily |  | [SSF57716(SUPERFAMILY)](http://www.ebi.ac.uk/interpro/signature/SSF57716) |
|  | no IPR | HOMOLOGOUS_SUPERFAMILY | XPA domain superfamily |  | mobidb-lite (MOBIDB_LITE) |
|  |  |  |  |  | mobidb-lite (MOBIDB_LITE) |
|  | no IPR |  |  |  | Coil (COILS) |
| *RsXPC* | [IPR004583](http://www.ebi.ac.uk/interpro/entry/IPR004583) | FAMILY | DNA repair protein Rad4 | GO:0006289, GO:0005634, GO:0003684 | [PTHR12135 (PANTHER)](http://www.ebi.ac.uk/interpro/signature/PTHR12135) |
|  |  |  |  |  | [PTHR12135 (PANTHER)](http://www.ebi.ac.uk/interpro/signature/PTHR12135) |
|  | [IPR018325](http://www.ebi.ac.uk/interpro/entry/IPR018325) | DOMAIN | Rad4/PNGase transglutaminase-like fold |  | [PF03835 (PFAM)](http://www.ebi.ac.uk/interpro/signature/PF03835) |
|  | [IPR018326](http://www.ebi.ac.uk/interpro/entry/IPR018326) | DOMAIN | Rad4 beta-hairpin domain 1 | GO:0003677 | [SM01030 (SMART)](http://www.ebi.ac.uk/interpro/signature/SM01030) |
|  |  |  |  |  | [PF10403 (PFAM)](http://www.ebi.ac.uk/interpro/signature/PF10403) |
|  | [IPR018327](http://www.ebi.ac.uk/interpro/entry/IPR018327) | DOMAIN | Rad4 beta-hairpin domain 2 | GO:0003677 | [SM01031 (SMART)](http://www.ebi.ac.uk/interpro/signature/SM01031) |
|  | [IPR018328](http://www.ebi.ac.uk/interpro/entry/IPR018328) | DOMAIN | Rad4 beta-hairpin domain 3 | GO:0003677 | [SM01032 (SMART)](http://www.ebi.ac.uk/interpro/signature/SM01032) |
|  |  |  |  |  | [PF10405 (PFAM)](http://www.ebi.ac.uk/interpro/signature/PF10405) |
|  | [IPR036985](http://www.ebi.ac.uk/interpro/entry/IPR036985) | HOMOLOGOUS_SUPERFAMILY | Transglutaminase-like superfamily |  | [G3DSA:3.90.260.10 (GENE3D)](http://www.ebi.ac.uk/interpro/signature/G3DSA:3.90.260.10) |
|  | no IPR | FAMILY | DNA repair protein Rad4 |  | [PTHR12135:SF0 (PANTHER)](http://www.ebi.ac.uk/interpro/signature/PTHR12135) |
|  |  |  |  |  | [PTHR12135:SF0 (PANTHER)](http://www.ebi.ac.uk/interpro/signature/PTHR12135) |
|  |  |  |  |  | TRANSMEMBRANE (PHOBIUS) |
|  |  |  |  |  | CYTOPLASMIC_DOMAIN (PHOBIUS) |
|  |  |  |  |  | NON_CYTOPLASMIC_DOMAIN (PHOBIUS) |
|  |  |  |  |  | SignalP-TM (SIGNALP_GRAM_POSITIVE) |
|  |  |  |  |  | [SSF54001 (SUPERFAMILY)](http://www.ebi.ac.uk/interpro/signature/SSF54001) |
|  | no IPR | DOMAIN | Rad4/PNGase transglutaminase-like fold |  | mobidb-lite (MOBIDB_LITE) |
|  |  |  |  |  | mobidb-lite (MOBIDB_LITE) |
|  |  |  |  |  | mobidb-lite (MOBIDB_LITE) |
|  |  |  |  |  | mobidb-lite (MOBIDB_LITE) |
|  |  |  |  |  | mobidb-lite (MOBIDB_LITE) |
|  | no IPR | HOMOLOGOUS_SUPERFAMILY | Transglutaminase-like superfamily |  | [G3DSA:3.30.70.2460 (GENE3D)](http://www.ebi.ac.uk/interpro/signature/G3DSA:3.30.70.2460) |
|  | no IPR |  |  |  | Coil (COILS) |
|  |  |  |  |  | Coil (COILS) |
| *RsXPF* | [IPR006166](http://www.ebi.ac.uk/interpro/entry/IPR006166) | DOMAIN | ERCC4 domain | GO:0003677, GO:0004518 | [SM00891 (SMART)](http://www.ebi.ac.uk/interpro/signature/SM00891) |
|  |  |  |  |  | [PF02732 (PFAM)](http://www.ebi.ac.uk/interpro/signature/PF02732) |
|  | [IPR010994](http://www.ebi.ac.uk/interpro/entry/IPR010994) | HOMOLOGOUS_SUPERFAMILY | RuvA domain 2-like |  | [SSF47781 (SUPERFAMILY)](http://www.ebi.ac.uk/interpro/signature/SSF47781) |
|  | [IPR011335](http://www.ebi.ac.uk/interpro/entry/IPR011335) | HOMOLOGOUS_SUPERFAMILY | Restriction endonuclease type II-like |  | [SSF52980 (SUPERFAMILY)](http://www.ebi.ac.uk/interpro/signature/SSF52980) |
|  | no IPR | DOMAIN | ERCC4 domain |  | [G3DSA:3.40.50.10130 (GENE3D)](http://www.ebi.ac.uk/interpro/signature/G3DSA:3.40.50.1013) |
|  |  |  |  |  | [G3DSA:1.10.150.20 (GENE3D)](http://www.ebi.ac.uk/interpro/signature/G3DSA:1.10.150.20) |
|  |  |  |  |  | mobidb-lite (MOBIDB_LITE) |
|  |  |  |  |  | mobidb-lite (MOBIDB_LITE) |
|  |  |  |  |  | mobidb-lite (MOBIDB_LITE) |
|  |  |  |  |  | [PTHR10150 (PANTHER)](http://www.ebi.ac.uk/interpro/signature/PTHR10150) |
| *RsXRCC1* | [IPR002706](http://www.ebi.ac.uk/interpro/entry/IPR002706) | DOMAIN | DNA-repair protein Xrcc1, N-terminal | GO:0000012, GO:0005634, GO:0003684 | [PF01834 (PFAM)](http://www.ebi.ac.uk/interpro/signature/PF01834) |
|  | [IPR008979](http://www.ebi.ac.uk/interpro/entry/IPR008979) | HOMOLOGOUS_SUPERFAMILY | Galactose-binding-like domain superfamily |  | [G3DSA:2.60.120.260(GENE3D)](http://www.ebi.ac.uk/interpro/signature/G3DSA:2.60.120.260) |
|  |  |  |  |  | [SSF49785(SUPERFAMILY)](http://www.ebi.ac.uk/interpro/signature/SSF49785) |
|  | no IPR | HOMOLOGOUS_SUPERFAMILY | Galactose-binding-like domain superfamily |  | [PTHR11370 (PANTHER)](http://www.ebi.ac.uk/interpro/signature/PTHR11370) |
|  | no IPR |  |  |  | Coil (COILS) |
| *RsXRCC2* | [IPR013632](http://www.ebi.ac.uk/interpro/entry/IPR013632) | DOMAIN | DNA recombination and repair protein Rad51-like, C-terminal |  | [PF08423 (PFAM)](http://www.ebi.ac.uk/interpro/signature/PF08423) |
|  | [IPR020588](http://www.ebi.ac.uk/interpro/entry/IPR020588) | DOMAIN | DNA recombination and repair protein RecA-like, ATP-binding domain | GO:0008094, GO:0006281, GO:0005524, GO:0003677 | [PS50162(PROSITE_PROFILES)](http://www.ebi.ac.uk/interpro/signature/PS50162) |
|  | [IPR027417](http://www.ebi.ac.uk/interpro/entry/IPR027417) | HOMOLOGOUS_SUPERFAMILY | P-loop containing nucleoside triphosphate hydrolase |  | [SSF52540(SUPERFAMILY)](http://www.ebi.ac.uk/interpro/signature/SSF52540) |
|  | [IPR030547](http://www.ebi.ac.uk/interpro/entry/IPR030547) | FAMILY | DNA repair protein XRCC2 | GO:0033063, GO:0005657, GO:0000724, GO:0006281 | [PTHR22942:SF44 (PANTHER)](http://www.ebi.ac.uk/interpro/signature/PTHR22942) |
|  | no IPR | DOMAIN | DNA recombination and repair protein Rad51-like, C-terminal |  | [PTHR22942(PANTHER)](http://www.ebi.ac.uk/interpro/signature/PTHR22942) |
|  | no IPR |  |  |  | [G3DSA:3.40.50.300(GENE3D)](http://www.ebi.ac.uk/interpro/signature/G3DSA:3.40.50.300) |
| *RsXRCC3* | [IPR013632](http://www.ebi.ac.uk/interpro/entry/IPR013632) | DOMAIN | DNA recombination and repair protein Rad51-like, C-terminal |  | [PF08423 (PFAM)](http://www.ebi.ac.uk/interpro/signature/PF08423) |
|  | [IPR016467](http://www.ebi.ac.uk/interpro/entry/IPR016467) | FAMILY | DNA recombination and repair protein, RecA-like |  | PIRSF005856 (PIRSF) |
|  | [IPR020588](http://www.ebi.ac.uk/interpro/entry/IPR020588) | DOMAIN | DNA recombination and repair protein RecA-like, ATP-binding domain | GO:0006281, GO:0003677, GO:0005524, GO:0008094 | [PS50162(PROSITE_PROFILES)](http://www.ebi.ac.uk/interpro/signature/PS50162) |
|  | [IPR027417](http://www.ebi.ac.uk/interpro/entry/IPR027417) | HOMOLOGOUS_SUPERFAMILY | P-loop containing nucleoside triphosphate hydrolase |  | [SSF52540(SUPERFAMILY)](http://www.ebi.ac.uk/interpro/signature/SSF52540) |
|  | [IPR033925](http://www.ebi.ac.uk/interpro/entry/IPR033925) | DOMAIN | Rad51/DMC1/RadA | GO:0003677 | cd01123 (CDD) |
|  | no IPR | DOMAIN | DNA recombination and repair protein Rad51-like, C-terminal |  | [PTHR22942:SF24 (PANTHER)](http://www.ebi.ac.uk/interpro/signature/PTHR22942) |
|  |  |  |  |  | [PTHR22942(PANTHER)](http://www.ebi.ac.uk/interpro/signature/PTHR22942) |
|  | no IPR | FAMILY | DNA recombination and repair protein, RecA-like |  | [G3DSA:3.40.50.300(GENE3D)](http://www.ebi.ac.uk/interpro/signature/G3DSA:3.40.50.300) |
| *RsXRCC4* | [IPR009089](http://www.ebi.ac.uk/interpro/entry/IPR009089) | HOMOLOGOUS_SUPERFAMILY | XRCC4, N-terminal domain superfamily | GO:0005634, GO:0006302, GO:0003677, GO:0006310 | [SSF50809(SUPERFAMILY)](http://www.ebi.ac.uk/interpro/signature/SSF50809) |
|  | [IPR010585](http://www.ebi.ac.uk/interpro/entry/IPR010585) | FAMILY | DNA repair protein XRCC4 | GO:0005634, GO:0003677, GO:0006310, GO:0006302 | [PF06632 (PFAM)](http://www.ebi.ac.uk/interpro/signature/PF06632) |
|  | no IPR | FAMILY | DNA repair protein XRCC4 |  | mobidb-lite (MOBIDB_LITE) |
|  |  |  |  |  | [PTHR28559(PANTHER)](http://www.ebi.ac.uk/interpro/signature/PTHR28559) |
|  |  |  |  |  | [SSF58022(SUPERFAMILY)](http://www.ebi.ac.uk/interpro/signature/SSF58022) |
|  | no IPR |  |  |  | Coil (COILS) |
|  |  |  |  |  | Coil (COILS) |
|  |  |  |  |  | [G3DSA:2.170.210.10(GENE3D)](http://www.ebi.ac.uk/interpro/signature/G3DSA:2.170.210.10) |
|  |  |  |  |  | [G3DSA:1.20.5.370(GENE3D)](http://www.ebi.ac.uk/interpro/signature/G3DSA:1.20.5.370) |
