# Supplementary material for: High expression of the breast cancer susceptibility gene BRCA1 in long-lived termite kings
Source: Aging (Albany NY). 2018 Oct 11;10(10):2668–83. doi: 10.18632/aging.101578 (PMC6224230; doi:10.18632/aging.101578)
Supplement: Supplementary Table S3 [file aging-10-101578-s004.docx]

**Table S3. Statistical analysis of differential expression among the termite castes.**

| Gene symbol | LR | *P*-value | FDR |
| --- | --- | --- | --- |
| UBC9 | 23.800 | <0.001 | **0.001 **** |
| RPA1 | 20.100 | <0.001 | **0.005 **** |
| RMI1 | 169.000 | <0.001 | **<0.001 ***** |
| PARP1 | 34.400 | <0.001 | **<0.001 ***** |
| CSNK2A | 49.300 | <0.001 | **<0.001 ***** |
| RFC3 | 20.000 | <0.001 | **0.005 **** |
| RRP1 | 49.500 | <0.001 | **<0.001 ***** |
| RAD23B | 16.300 | 0.003 | **0.024 *** |
| APLF | 16.800 | 0.002 | **0.019 *** |
| HUS1 | 44.400 | <0.001 | **<0.001 ***** |
| POLG1 | 37.700 | <0.001 | **<0.001 ***** |
| NBS1 | 74.000 | <0.001 | **<0.001 ***** |
| XRCC3 | 19.500 | <0.001 | **0.006 **** |
| SSBP | 17.100 | 0.002 | **0.017 *** |
| CHK1 | 30.000 | <0.001 | **<0.001 ***** |
| ALKBH1 | 31.800 | <0.001 | **<0.001 ***** |
| MERIT40 | 20.500 | <0.001 | **0.004 **** |
| MCPH1 | 75.700 | <0.001 | **<0.001 ***** |
| ALKBH7 | 15.000 | 0.005 | **0.039 *** |
| TP53 | 12.200 | 0.016 | 0.114 |
| ERCC8 | 56.600 | <0.001 | **<0.001 ***** |
| XRCC2 | 34.900 | <0.001 | **<0.001 ***** |
| APTX | 22.100 | <0.001 | **0.002 **** |
| MAPK14 | 53.100 | <0.001 | **<0.001 ***** |
| NTHL1 | 37.300 | <0.001 | **<0.001 ***** |
| SSRP1 | 16.500 | 0.002 | **0.021 *** |
| DCLRE1A | 34.000 | <0.001 | **<0.001 ***** |
| MDC1 | 4.890 | 0.298 | 1.000 |
| EN5 | 13.000 | 0.012 | 0.086 |
| RTEL1 | 127.000 | <0.001 | **<0.001 ***** |
| RAD1 | 32.900 | <0.001 | **<0.001 ***** |
| RAD9A | 40.400 | <0.001 | **<0.001 ***** |
| RAD50 | 31.400 | <0.001 | **<0.001 ***** |
| CDC6 | 76.700 | <0.001 | **<0.001 ***** |
| GTF2H2 | 37.100 | <0.001 | **<0.001 ***** |
| BRCA1 | 65.000 | <0.001 | **<0.001 ***** |
| ABRA1 | 4.950 | 0.293 | 1.000 |
| GEN1 | 113.000 | <0.001 | **<0.001 ***** |
| XRCC4 | 25.000 | <0.001 | **0.001 **** |
| CDK1 | 157.000 | <0.001 | **<0.001 ***** |
| ALKBH8 | 16.500 | 0.002 | **0.021 *** |
| DDB1 | 39.500 | <0.001 | **<0.001 ***** |
| DCLRE1C | 10.600 | 0.031 | 0.204 |
| TIM | 91.200 | <0.001 | **<0.001 ***** |
| MPG | 18.600 | 0.001 | **0.009 **** |
| POLI | 77.300 | <0.001 | **<0.001 ***** |
| RFC5 | 19.400 | 0.001 | **0.007 **** |
| BRE | 29.500 | <0.001 | **<0.001 ***** |
| SLX4 | 16.100 | 0.003 | **0.025 *** |
| RAD51C | 12.600 | 0.014 | **0.010 *** |
| MRE11 | 21.900 | <0.001 | **0.002 **** |
| RAD18 | 11.200 | 0.025 | 0.170 |
| PCNA | 46.000 | <0.001 | **<0.001 ***** |
| PIAS1 | 5.490 | 0.241 | 1.000 |
| TAOK1 | 8.300 | 0.081 | 0.463 |
| ALKBH4 | 33.500 | <0.001 | **<0.001 ***** |
| XPC | 12.000 | 0.017 | 0.124 |
| KU80 | 9.550 | 0.049 | 0.300 |
| RFC4 | 9.270 | 0.055 | 0.331 |
| RNF168 | 62.400 | <0.001 | **<0.001 ***** |
| CDK2 | 29.700 | <0.001 | **<0.001 ***** |
| RECQL4 | 40.600 | <0.001 | **<0.001 ***** |
| RAD51D | 3.540 | 0.472 | 1.000 |
| LIG4 | 25.200 | <0.001 | **0.001 **** |
| BLM | 53.900 | <0.001 | **<0.001 ***** |
| HELQ | 63.000 | <0.001 | **<0.001 ***** |
| TP53BP1 | 36.200 | <0.001 | **<0.001 ***** |
| SHPRH | 12.200 | 0.016 | 0.116 |
| ALKBH6 | 22.900 | <0.001 | **0.002 **** |
| PRIM2 | 165.000 | <0.001 | **<0.001 ***** |
| RAD52 | 7.270 | 0.122 | 0.645 |
| TOPBP1 | 30.800 | <0.001 | **<0.001 ***** |
| ERCC1 | 12.600 | 0.014 | 0.100 |
| RFC2 | 22.200 | <0.001 | **0.002 **** |
| ERCC5 | 22.100 | <0.001 | **0.002 **** |
| ERCC6 | 16.500 | 0.002 | **0.022 *** |
| DCLRE1B | 11.600 | 0.021 | 0.145 |
| RAD54-like | 32.800 | <0.001 | **<0.001 ***** |
| MLH1 | 16.200 | 0.003 | **0.024 *** |
| BRCA2 | 19.500 | 0.001 | **0.006 **** |
| CHK2 | 105.000 | <0.001 | **<0.001 ***** |
| BRCC3 | 3.970 | 0.4100 | 1.000 |
| SLX1 | 14.600 | 0.006 | **0.046 *** |
| PMS1 | 7.520 | 0.111 | 0.592 |
| ASCC3b | 11.600 | 0.021 | 0.144 |
| MSH4 | 32.700 | <0.001 | **<0.001 ***** |
| POLB | 20.700 | <0.001 | **0.004 **** |
| HERC2 | 21.200 | <0.001 | **0.003 **** |
| SMARCAL1 | 8.770 | 0.067 | 0.395 |
| ASCC3a | 18.000 | 0.001 | **0.012 *** |
| PNKP | 41.600 | <0.001 | **<0.001 ***** |
| RMI2 | 3.410 | 0.491 | 1.000 |
| ALKBH5 | 68.600 | <0.001 | **<0.001 ***** |
| XPF | 2.820 | 0.588 | 1.000 |
| CLASPIN | 95.700 | <0.001 | **<0.001 ***** |
| ERCC2 | 26.800 | <0.001 | **<0.001 ***** |
| MGMT | 13.100 | 0.011 | 0.081 |
| UBC13 | 31.900 | <0.001 | **<0.001 ***** |
| ATR | 51.400 | <0.001 | **<0.001 ***** |
| ATM | 16.200 | 0.003 | **0.024 *** |
| MSH6 | 13.000 | 0.012 | 0.086 |
| MSH3 | 13.100 | 0.011 | 0.080 |
| KU70 | 35.100 | <0.001 | **<0.001 ***** |
| RNF8 | 4.120 | 0.390 | 1.000 |
| H2AFY | 25.400 | <0.001 | **<0.001 ***** |
| ECT2 | 34.100 | <0.001 | **<0.001 ***** |
| ERCC3 | 12.500 | 0.014 | 0.101 |
| FANCM | 32.800 | <0.001 | **<0.001 ***** |
| CRY1 | 14.600 | 0.006 | **0.045 *** |
| PMS2 | 8.270 | 0.082 | 0.467 |
| DDX1 | 21.400 | <0.001 | **0.003 **** |
| XPA | 7.160 | 0.128 | 0.663 |
| RAD17 | 3.760 | 0.439 | 1.000 |
| RAD51A | 26.800 | <0.001 | **<0.001 ***** |
| REV1 | 25.200 | <0.001 | **<0.001 ***** |
| BRIP1 | 50.100 | <0.001 | **<0.001 ***** |
| NHEJ1 | 12.000 | 0.017 | 0.124 |
| MAPKAPK2 | 63.100 | <0.001 | **<0.001 ***** |
| RFC1 | 19.200 | 0.001 | **0.007 **** |
| XRCC1 | 7.480 | 0.113 | 0.602 |
| POLH | 20.800 | <0.001 | **0.004 **** |
| MUS81 | 45.400 | <0.001 | **<0.001 ***** |
| RAD54B | 10.500 | 0.033 | 0.217 |
| BARD1 | 19.000 | 0.001 | **0.008 **** |
| EME1 | 29.200 | <0.001 | **<0.001 ***** |
| MLH3 | 68.000 | <0.001 | **<0.001 ***** |
| PRKDC | 51.400 | <0.001 | **<0.001 ***** |

Comparison of normalized counts per million among castes was conducted using the edgeR package. Bold letters indicate significant differences (*FDR < 0.05, **FDR < 0.01, **FDR < 0.001). LR: likelihood ratio, FDR: false discovery rate.
