# Supplementary material for: High expression of the breast cancer susceptibility gene BRCA1 in long-lived termite kings
Source: Aging (Albany NY). 2018 Oct 11;10(10):2668–83. doi: 10.18632/aging.101578 (PMC6224230; doi:10.18632/aging.101578)
Supplement: Supplementary Table S4 [file aging-10-101578-s005.docx]

**Table S4. Factor loadings for each principal component.**

|  | PC1 | PC2 | PC3 | PC4 | PC5 | PC6 | PC7 | PC8 | PC9 | PC10 |
| --- | --- | --- | --- | --- | --- | --- | --- | --- | --- | --- |
| BRCA1 | 6.09 | 0.29 | -1.72 | -0.19 | 0.04 | 0.42 | -0.17 | -0.96 | 0.03 | -0.15 |
| CDK1 | 6 | 1.66 | -1.69 | -0.58 | -0.27 | -0.18 | 0.54 | -0.47 | 0.1 | -0.13 |
| MCPH1 | 5.53 | 0.7 | -1.67 | -0.64 | -0.06 | -0.27 | 0.01 | -0.58 | 0.04 | -0.09 |
| MSH4 | 4.84 | 0.5 | -2.05 | -0.64 | 0.13 | 0.26 | 0.6 | -0.2 | -0.01 | -0.19 |
| RAD54-like | 4.71 | 0.69 | -0.72 | 0.89 | -0.12 | -0.41 | -0.48 | -0.43 | 0.79 | 0.09 |
| ATR | 4.59 | -1.27 | -1.73 | -0.15 | 0.34 | 0.79 | 0.44 | -0.37 | -0.16 | -0.09 |
| RMI1 | 4.53 | 0.38 | 1.42 | 0.77 | -0.44 | -0.55 | -0.27 | -0.26 | -0.38 | -0.02 |
| HELQ | 4.26 | -1.89 | -1.54 | 0.49 | 0.32 | 0.07 | -0.06 | -0.44 | -0.1 | -0.25 |
| MGMT | 4.25 | 1.11 | -2.86 | -0.25 | 0.34 | 0.09 | 0.5 | -0.3 | 0.02 | -0.02 |
| GEN1 | 4.03 | 1.48 | 0.61 | 0.36 | -0.47 | -0.01 | -0.24 | -0.04 | -0.33 | -0.18 |
| CHK2 | 3.94 | 1.56 | 0.84 | 0.19 | 0.24 | 0.46 | 0.4 | 0.01 | 0.37 | 0.27 |
| PRIM2 | 3.58 | 2.18 | 3.25 | 0.27 | -1.11 | 0.21 | 1.08 | -0.1 | 0.21 | -0.23 |
| MLH1 | 3.26 | 0.75 | -1.63 | -1.27 | 0.14 | 0.05 | -0.08 | -0.04 | -0.13 | 0.13 |
| ATM | 2.95 | -0.64 | -1.44 | -1.48 | -0.58 | -0.43 | 0.31 | 0.17 | -0.14 | 0.37 |
| XRCC3 | 2.83 | 1.15 | -1.88 | 0.28 | 0.81 | 0.14 | 0.03 | -0.08 | 0.39 | 0.24 |
| TIM | 2.82 | 1.41 | 1.67 | -2.02 | -0.79 | -0.05 | -0.12 | 0.1 | -0.05 | -0.08 |
| ERCC1 | 2.33 | 0.6 | -1.16 | 0.01 | 0.69 | -0.49 | -0.4 | 0.03 | 0.05 | -0.09 |
| BLM | 2.15 | -1.53 | 1.1 | -0.17 | -0.4 | -0.76 | -0.24 | 0.14 | -0.07 | -0.01 |
| CLASPIN | 2.14 | 0.82 | 2.14 | 0.44 | -0.87 | 1.03 | -0.15 | -0.02 | -0.06 | -0.15 |
| TP53BP1 | 2.13 | -2.94 | -0.31 | 0.72 | -0.25 | 0.32 | -0.49 | -0.02 | 0.08 | -0.46 |
| SLX4 | 2.08 | -0.1 | -0.27 | -0.27 | -0.38 | 0.05 | -0.05 | 0.42 | 0.33 | 0.01 |
| RECQL4 | 2 | -2.31 | 1.57 | 1.27 | -0.02 | 0.07 | -0.4 | -0.28 | 0.19 | 0.43 |
| RAD50 | 1.82 | -0.99 | 0.3 | -0.55 | -0.2 | -0.47 | -0.31 | 0.17 | -0.02 | 0.28 |
| MLH3 | 1.75 | -4.18 | -0.44 | -0.12 | 0.33 | -0.17 | 0.85 | 0.14 | -0.36 | -0.2 |
| RFC3 | 1.62 | 0.74 | -1.37 | -0.35 | 0.42 | 0.54 | 0.05 | 0.24 | -0.18 | 0.31 |
| RNF168 | 1.53 | -1.21 | 1.9 | 0.58 | 0.27 | -0.2 | 0.06 | 0.07 | 0.19 | -0.14 |
| MAPK14 | 1.45 | 1.66 | 1.71 | -0.2 | 0.17 | -0.91 | 0.29 | 0.23 | 0.67 | 0.05 |
| CDC6 | 1.37 | 2.03 | 2.43 | 0.91 | -0.61 | 0.2 | 0.07 | -0.11 | 0.2 | -0.29 |
| TOPBP1 | 1.32 | 0.41 | -0.93 | -0.22 | -1.22 | 0.5 | -0.16 | 0.59 | -0.11 | -0.14 |
| NTHL1 | 1.14 | 0.8 | 0.3 | 0.84 | -0.58 | 0.08 | -0.28 | 0.38 | -0.29 | -0.11 |
| XRCC2 | 1.12 | 1.29 | -0.73 | 0.63 | 0.07 | 0.73 | -0.01 | 0.33 | -0.12 | 0.15 |
| APLF | 1.11 | 0.96 | -0.87 | 0.31 | 0.61 | -0.52 | 0 | 0.32 | -0.58 | -0.08 |
| BRCA2 | 1.1 | -2.09 | 0.24 | -0.54 | 0.01 | 0.29 | -0.95 | -0.24 | -0.06 | 0.26 |
| ASCC3a | 1.04 | -1.93 | -0.08 | -0.14 | -0.17 | -0.32 | 0.62 | 0.57 | -0.05 | 0.04 |
| ERCC5 | 1.03 | 0.07 | 1.09 | -0.62 | -0.62 | -0.21 | -0.38 | 0.26 | 0.51 | 0.06 |
| POLI | 1.01 | 1.75 | 2.22 | -0.19 | -0.11 | -0.49 | 0.17 | 0.03 | -0.29 | -0.17 |
| MSH6 | 0.98 | -2.48 | 0.69 | -0.05 | -0.29 | -0.07 | 0.24 | 0.29 | 0.33 | 0.18 |
| SMARCAL1 | 0.94 | 0.12 | -0.87 | 0.25 | 0.15 | -0.02 | -0.49 | 0.25 | -0.35 | -0.22 |
| CHK1 | 0.93 | 0.05 | 0.32 | 1.67 | -0.03 | 0.3 | -0.21 | 0.09 | -0.43 | 0.33 |
| NBS1 | 0.91 | 1.78 | 1.9 | -0.19 | -0.25 | -0.2 | 0.35 | 0.17 | -0.08 | 0.2 |
| FANCM | 0.8 | -4.15 | 0.25 | 0.76 | -0.34 | -0.66 | 0.32 | 0.15 | -0.04 | -0.28 |
| PRKDC | 0.78 | -0.43 | 1.34 | -1.26 | -0.58 | 0.71 | -0.07 | 0.18 | -0.11 | 0.18 |
| RFC4 | 0.76 | 0.55 | -0.77 | 0.64 | 0.29 | -0.67 | -0.09 | 0.41 | 0.25 | -0.07 |
| DCLRE1A | 0.76 | -1.4 | 1.13 | 0.68 | -0.16 | 0.23 | -0.95 | -0.14 | -0.17 | -0.16 |
| APTX | 0.71 | 1.19 | -0.38 | 0.6 | 0.1 | 0.1 | -0.24 | 0.35 | -0.16 | -0.16 |
| ALKBH5 | 0.58 | 0.86 | 1.78 | 1 | 0.64 | 0.98 | 0.4 | -0.13 | -0.47 | -0.07 |
| ERCC6 | 0.51 | -2.44 | -0.36 | 1.23 | 0.26 | -0.48 | -0.42 | 0.05 | 0.25 | -0.11 |
| RAD18 | 0.46 | 0.41 | -0.23 | 0.06 | -0.42 | 0.07 | -0.62 | 0.25 | -0.16 | 0.19 |
| KU70 | 0.45 | 1.34 | -0.3 | 0.18 | 0.64 | -0.03 | 0.15 | 0.42 | -0.1 | -0.18 |
| HERC2 | 0.4 | -3.84 | -0.23 | 0.73 | 0.19 | -1.1 | 0.37 | 0.12 | 0.34 | -0.1 |
| SHPRH | 0.39 | -2.36 | -0.24 | -0.5 | 0.1 | 0.86 | -0.42 | -0.12 | -0.21 | 0.49 |
| RPA1 | 0.17 | -0.42 | -0.09 | -0.09 | 0.71 | -0.31 | -0.15 | 0.17 | -0.52 | 0.13 |
| PARP1 | 0.15 | 0.67 | -0.37 | 1.16 | 0 | 0.49 | 0.46 | 0.41 | 0.04 | 0.66 |
| RAD52 | 0.14 | -1.07 | -0.68 | -1.13 | -0.38 | -0.08 | -0.36 | 0.4 | 0.64 | -0.58 |
| ECT2 | 0.09 | -1.98 | 1.26 | -0.6 | -0.01 | 0.41 | 0.19 | 0.05 | -0.22 | 0.24 |
| SSRP1 | 0.07 | 0.63 | -0.66 | 0.58 | -0.01 | 0.1 | -0.16 | 0.4 | 0.08 | 0.09 |
| RAD9A | 0.06 | 0.36 | 2.23 | -0.06 | -0.1 | -0.13 | 0.05 | -0.06 | 0.26 | -0.21 |
| MPG | 0.04 | 0.91 | -0.28 | -0.64 | 0.1 | -0.37 | -0.27 | 0.3 | -0.4 | 0.07 |
| POLG1 | -0.01 | 1.28 | -0.44 | 0.53 | -0.31 | 0.79 | 0.03 | 0.43 | 0.01 | 0.26 |
| PNKP | -0.07 | 0.69 | 1.81 | 1.54 | 0.07 | -0.47 | -0.18 | -0.19 | -0.06 | -0.2 |
| HUS1 | -0.08 | 0.22 | 2.33 | 1.58 | 0.5 | -0.18 | 0.05 | -0.54 | -0.15 | 0.09 |
| MUS81 | -0.1 | -2.75 | 0.87 | -1.11 | -0.01 | -1.02 | 0.53 | 0 | -0.77 | 0.16 |
| KU80 | -0.15 | 0.63 | -0.5 | 0.07 | -0.05 | -0.11 | -0.31 | 0.41 | -0.14 | -0.24 |
| ASCC3b | -0.31 | -2.91 | -0.31 | 1.33 | -0.37 | 0.72 | 0.05 | 0.23 | 0.03 | -0.19 |
| BRIP1 | -0.38 | 0.86 | 1.87 | -1.41 | -1.3 | -0.19 | -0.05 | 0.04 | -0.3 | -0.18 |
| ERCC8 | -0.38 | 1.72 | 2.27 | 0.58 | -0.2 | 0.07 | 0.09 | -0.46 | 0.01 | 0.32 |
| ABRA1 | -0.44 | -0.23 | -0.28 | 0.47 | -0.05 | 0.09 | -0.29 | 0.3 | 0.27 | 0.1 |
| PMS2 | -0.53 | -1.87 | 0.4 | 0.19 | -0.5 | -1.48 | -0.03 | 0.03 | -0.22 | 0.72 |
| RAD51D | -0.55 | -0.07 | -0.58 | 0.4 | 0.28 | -0.44 | -0.23 | 0.31 | -0.27 | -0.24 |
| ERCC2 | -0.61 | 0.36 | 0.13 | -1.62 | 0.17 | -0.36 | 0.12 | 0.25 | -0.3 | 0.11 |
| RAD1 | -0.7 | 1.16 | 0.03 | 0.37 | 0.37 | 0.17 | 0.14 | 0.21 | 0.12 | 0.47 |
| RFC1 | -0.86 | -2 | 0.51 | -1.5 | -0.33 | 0.44 | 0.1 | 0.07 | 0.03 | -0.1 |
| RAD54B | -0.96 | 0.21 | -0.44 | -0.26 | 0.3 | 0.26 | -0.24 | 0.18 | 0.31 | 0.23 |
| BARD1 | -0.97 | -1.02 | 0.46 | -0.4 | 0.45 | -0.1 | -0.83 | -0.26 | -0.76 | -0.41 |
| DDB1 | -1 | 1.57 | -0.94 | -0.55 | 0.44 | -0.05 | -0.19 | 0.21 | 0.29 | 0.19 |
| BRE | -1.04 | 1.32 | 0.65 | 0.4 | -0.13 | -0.16 | 0.08 | 0.18 | -0.09 | 0.09 |
| UBC9 | -1.07 | 1.12 | -1.11 | 0.2 | 0.1 | 0.1 | 0.14 | 0.37 | -0.25 | 0.31 |
| MRE11 | -1.08 | 1.21 | -0.27 | 0.75 | 0.34 | 0.14 | 0.02 | 0.28 | 0.08 | -0.26 |
| XPC | -1.1 | -2.41 | 0.92 | 0.99 | 0.08 | 0.72 | 0.03 | -0.16 | 0.11 | -0.03 |
| PIAS1 | -1.19 | -1.78 | 0.13 | 0.7 | 0.66 | -0.25 | 0.22 | -0.04 | 0.19 | 0.45 |
| RNF8 | -1.2 | -0.87 | -0.79 | -0.58 | -0.19 | 0.38 | 0.02 | 0.3 | -0.01 | 0.22 |
| ALKBH7 | -1.26 | 0.81 | -1.35 | -0.34 | 0.37 | 0.52 | -0.12 | 0.27 | -0.23 | -0.16 |
| BRCC3 | -1.31 | 0.3 | -0.66 | 0.38 | 0.05 | -0.55 | -0.37 | 0.23 | 0.25 | -0.17 |
| DCLRE1B | -1.37 | -0.75 | 0.61 | 1.02 | -0.37 | 0.44 | 0.38 | 0.16 | -0.34 | 0.23 |
| GTF2H2 | -1.38 | 1.81 | 0.46 | 0.33 | 0.25 | 0.2 | -0.12 | -0.02 | -0.14 | -0.18 |
| MDC1 | -1.39 | -1.8 | -0.8 | -0.07 | 0.27 | 0.12 | 0.05 | 0.1 | 0.59 | 0.29 |
| DDX1 | -1.41 | 0.79 | 0.73 | 0.7 | 0.21 | -0.48 | 0.1 | 0.01 | 0.22 | 0.02 |
| LIG4 | -1.41 | 0.91 | 1.54 | 0.2 | -0.09 | -0.26 | -0.18 | -0.38 | 0.2 | 0.22 |
| RFC2 | -1.48 | 0.41 | 0.74 | -0.22 | 0.52 | 0.1 | 0.05 | -0.11 | -0.13 | 0.14 |
| PCNA | -1.49 | 2.07 | 0.47 | -0.76 | -0.44 | -0.5 | -0.09 | 0.1 | -0.22 | -0.09 |
| POLH | -1.52 | -0.03 | 0.24 | -1.65 | -0.07 | 0.48 | 0.07 | 0.08 | 0.18 | 0.1 |
| ALKBH6 | -1.55 | 1.11 | 1.78 | -0.81 | -0.01 | -0.21 | -0.43 | -0.67 | 0.16 | 0.09 |
| H2AFY | -1.55 | 1.57 | 0.93 | -0.38 | -0.72 | 0.68 | 0.27 | 0.24 | 0.54 | -0.33 |
| RAD51C | -1.6 | 0.2 | -0.61 | -0.11 | -0.19 | 0.28 | 0.02 | 0.3 | 0.28 | 0.28 |
| ALKBH1 | -1.62 | 1.53 | -0.99 | 1.93 | 0.3 | -0.06 | -0.41 | -0.14 | 0.03 | -0.06 |
| ALKBH4 | -1.65 | 0.44 | 0.62 | -2.02 | 0.46 | -0.11 | -0.17 | -0.41 | -0.23 | 0.31 |
| EN5 | -1.67 | 0.19 | -0.26 | -0.95 | -0.59 | -0.39 | 0.19 | 0.5 | 0.4 | -0.45 |
| RFC5 | -1.69 | 1.09 | -1.3 | 0.84 | 1.19 | -0.56 | 0.22 | 0.03 | -0.12 | -0.1 |
| MSH3 | -1.72 | -0.18 | 0.05 | -0.56 | 0.88 | 0.99 | -0.38 | -0.42 | -0.17 | 0.41 |
| REV1 | -1.73 | 0.72 | 0.28 | -1.59 | 0.42 | -1.06 | 0.06 | -0.17 | -0.26 | -0.02 |
| ALKBH8 | -1.78 | 0.16 | 0.83 | 0.1 | 0.36 | 0.14 | 0.15 | -0.08 | 0.01 | -0.26 |
| TAOK1 | -1.81 | -2.08 | 0.66 | -0.88 | -0.6 | 0.05 | 0.5 | 0.04 | 0.36 | -0.07 |
| MERIT40 | -1.87 | 1.36 | -0.63 | -0.32 | -0.22 | -0.43 | -0.09 | 0.28 | -0.05 | -0.23 |
| XRCC1 | -1.89 | -2.99 | 0.55 | 0.16 | 0.13 | 0.13 | 0.04 | -0.42 | -0.14 | -0.02 |
| SLX1 | -1.9 | 0.17 | -1.05 | -0.6 | 0.71 | -0.05 | -0.15 | -0.02 | -0.31 | -0.07 |
| RAD51A | -1.94 | -0.36 | 0.19 | -1 | 1.94 | -0.65 | 0.33 | -0.43 | 0.39 | -0.46 |
| XPA | -2 | -1.37 | -0.18 | -0.08 | 0.73 | -0.28 | 0.04 | -0.24 | 0.35 | 0.33 |
| POLB | -2.01 | 0.7 | -0.68 | 0.04 | 0.66 | 0.68 | 0.23 | 0.04 | 0.14 | 0.23 |
| RAD17 | -2.05 | -0.86 | 0.46 | -0.27 | 0.01 | 0.65 | 0.05 | -0.05 | 0.51 | -0.18 |
| CSNK2A | -2.05 | 0.76 | 0.27 | -2.43 | -0.34 | -0.22 | -0.4 | -0.41 | 0.09 | 0.11 |
| PMS1 | -2.05 | -0.64 | -1.1 | 0.11 | 0.68 | 0.45 | -0.07 | 0.04 | 0.47 | -0.24 |
| RMI2 | -2.07 | -1.08 | -0.34 | 0.09 | 0.05 | 0.12 | 0.25 | 0.07 | -0.24 | 0.22 |
| XPF | -2.1 | -0.43 | -0.13 | 0.1 | 0.15 | -0.03 | -0.34 | -0.08 | 0.4 | -0.13 |
| ERCC3 | -2.2 | 0.35 | -0.62 | 0.55 | 0.28 | -0.42 | 0.09 | 0.11 | 0.39 | -0.05 |
| EME1 | -2.28 | 1.04 | -0.45 | -0.12 | 0.63 | 0 | 0.51 | 0.08 | -0.11 | 0 |
| CDK2 | -2.5 | -0.03 | -0.34 | -1.3 | 0.73 | 0.5 | -0.25 | -0.45 | 0.66 | 0.15 |
| SSBP | -2.54 | 0.72 | -0.3 | 0.67 | 0.87 | 0.03 | 0.18 | -0.31 | -0.27 | -0.06 |
| CRY1 | -2.63 | 0.63 | -0.26 | 0.54 | 0.75 | 0.23 | 0.15 | -0.37 | -0.14 | 0.17 |
| NHEJ1 | -2.65 | 1.1 | -0.54 | 0.81 | -0.06 | -0.13 | -0.11 | -0.09 | -0.04 | -0.16 |
| RAD23B | -2.68 | 1.52 | -0.97 | 1.24 | 0.7 | -0.66 | -0.18 | -0.33 | 0.09 | -0.4 |
| TP53 | -2.84 | -1.06 | 0.84 | -0.04 | -0.73 | 0.94 | 0.52 | -0.33 | -0.06 | -0.28 |
| RRP1 | -2.9 | 1.67 | -0.9 | -1.04 | 0.32 | -0.09 | -0.19 | -0.34 | -0.09 | 0.01 |
| UBC13 | -3.13 | 1.68 | -0.89 | 0.2 | -0.63 | 0.35 | 0.02 | -0.06 | -0.19 | -0.07 |
| XRCC4 | -3.29 | 0.96 | -1.68 | 1 | 0.04 | -0.19 | 0.63 | 0.02 | -0.38 | -0.22 |
| MAPKAPK2 | -3.3 | -1.97 | -1.21 | -0.81 | -2.06 | 0.96 | -0.19 | -0.09 | -0.31 | -0.83 |
| DCLRE1C | -3.33 | -0.54 | -0.06 | -0.13 | 1.28 | 0.12 | 0.78 | -0.75 | -0.19 | -0.33 |
| RTEL1 | -5.14 | 0.76 | -3.82 | 1.43 | -5.41 | -0.87 | 0.26 | -0.96 | 0.04 | 0.44 |

These genes were sorted by their degree of PC1 factor loadings.
