# Supplementary material for: High expression of the breast cancer susceptibility gene BRCA1 in long-lived termite kings
Source: Aging (Albany NY). 2018 Oct 11;10(10):2668–83. doi: 10.18632/aging.101578 (PMC6224230; doi:10.18632/aging.101578)
Supplement: Supplementary Table S5 [file aging-10-101578-s006.docx]

**Table S5. Statistical analysis of age-dependent expression changes in termite kings.**

| Gene symbol | LR | *P*-value | FDR |
| --- | --- | --- | --- |
| UBC9 | 11.520 | 0.003 | **<0.001 ***** |
| RPA1 | 13.684 | 0.001 | **<0.001 ***** |
| RMI1 | 67.722 | 0.000 | **<0.001 ***** |
| PARP1 | 6.802 | 0.033 | **<0.001 ***** |
| ^†^CSNK2A | 6.365 | 0.041 | <0.001 *** |
| RFC3 | 123.865 | 0.000 | **<0.001 ***** |
| ^†^RRP1 | 16.845 | 0.000 | <0.001 *** |
| RAD23B | 4.789 | 0.091 | **<0.001 ***** |
| APLF | 12.403 | 0.002 | **<0.001 ***** |
| HUS1 | 2.344 | 0.310 | **0.037 *** |
| POLG1 | 1.735 | 0.420 | **<0.001 ***** |
| NBS1 | 10.395 | 0.006 | **<0.001 ***** |
| XRCC3 | 9.567 | 0.008 | **<0.001 ***** |
| ^†^SSBP | 25.874 | 0.000 | 0.017 * |
| CHK1 | 1.457 | 0.483 | **<0.001 ***** |
| ALKBH1 | 1.569 | 0.456 | **<0.001 ***** |
| MERIT40 | 0.951 | 0.621 | **<0.001 ***** |
| MCPH1 | 9.709 | 0.008 | **<0.001 ***** |
| ALKBH7 | 24.288 | 0.000 | **<0.001 ***** |
| ^†^TP53 | 4.863 | 0.088 | <0.001 *** |
| ERCC8 | 61.980 | 0.000 | **<0.001 ***** |
| XRCC2 | 5.381 | 0.068 | **<0.001 ***** |
| APTX | 5.149 | 0.076 | **<0.001 ***** |
| MAPK14 | 5.910 | 0.052 | **<0.001 ***** |
| NTHL1 | 6.998 | 0.030 | **<0.001 ***** |
| SSRP1 | 57.543 | 0.000 | **<0.001 ***** |
| DCLRE1A | 3.560 | 0.169 | **<0.001 ***** |
| MDC1 | 34.649 | 0.000 | **0.013 *** |
| EN5 | 114.564 | 0.000 | **0.025 *** |
| ^†^RTEL1 | 93.255 | 0.000 | <0.001 *** |
| RAD1 | 18.431 | 0.000 | **<0.001 ***** |
| RAD9A | 125.513 | 0.000 | **0.005 **** |
| RAD50 | 31.563 | 0.000 | **<0.001 ***** |
| CDC6 | 232.748 | 0.000 | **<0.001 ***** |
| GTF2H2 | 21.009 | 0.000 | **<0.001 ***** |
| BRCA1 | 44.421 | 0.000 | **<0.001 ***** |
| ABRA1 | 46.829 | 0.000 | **<0.001 ***** |
| GEN1 | 3.812 | 0.149 | **<0.001 ***** |
| XRCC4 | 20.167 | 0.000 | 0.313 |
| CDK1 | 2.073 | 0.355 | **<0.001 ***** |
| ALKBH8 | 25.699 | 0.000 | 0.178 |
| DDB1 | 70.011 | 0.000 | **<0.001 ***** |
| ^†^DCLRE1C | 74.699 | 0.000 | <0.001 *** |
| TIM | 75.973 | 0.000 | **<0.001 ***** |
| MPG | 11.206 | 0.004 | **<0.001 ***** |
| POLI | 204.229 | 0.000 | **<0.001 ***** |
| RFC5 | 25.400 | 0.000 | **<0.001 ***** |
| BRE | 4.661 | 0.097 | **<0.001 ***** |
| SLX4 | 21.120 | 0.000 | **<0.001 ***** |
| RAD51C | 29.931 | 0.000 | **<0.001 ***** |
| MRE11 | 7.797 | 0.020 | **<0.001 ***** |
| RAD18 | 23.465 | 0.000 | **<0.001 ***** |
| PCNA | 153.995 | 0.000 | **<0.001 ***** |
| PIAS1 | 181.702 | 0.000 | **0.001 **** |
| TAOK1 | 287.130 | 0.000 | 0.557 |
| ^†^ALKBH4 | 0.911 | 0.634 | 0.001 ** |
| XPC | 57.744 | 0.000 | 0.349 |
| KU80 | 33.278 | 0.000 | **<0.001 ***** |
| RFC4 | 11.110 | 0.004 | **<0.001 ***** |
| RNF168 | 141.981 | 0.000 | **<0.001 ***** |
| ^†^CDK2 | 75.346 | 0.000 | 0.001 ** |
| RECQL4 | 19.311 | 0.000 | **<0.001 ***** |
| RAD51D | 113.362 | 0.000 | **<0.001 ***** |
| ^†^LIG4 | 30.489 | 0.000 | 0.026 * |
| BLM | 6.322 | 0.042 | **<0.001 ***** |
| HELQ | 78.811 | 0.000 | **<0.001 ***** |
| TP53BP1 | 166.229 | 0.000 | **<0.001 ***** |
| SHPRH | 77.425 | 0.000 | **0.042 *** |
| ^†^ALKBH6 | 7.311 | 0.026 | <0.001 *** |
| PRIM2 | 44.264 | 0.000 | **<0.001 ***** |
| RAD52 | 38.930 | 0.000 | **<0.001 ***** |
| TOPBP1 | 14.300 | 0.001 | **<0.001 ***** |
| ERCC1 | 43.971 | 0.000 | **<0.001 ***** |
| RFC2 | 110.170 | 0.000 | **0.004 **** |
| ERCC5 | 43.309 | 0.000 | **<0.001 ***** |
| ERCC6 | 2.106 | 0.349 | **<0.001 ***** |
| DCLRE1B | 14.073 | 0.001 | 0.634 |
| RAD54-like | 1.169 | 0.557 | **<0.001 ***** |
| MLH1 | 59.919 | 0.000 | **<0.001 ***** |
| BRCA2 | 60.365 | 0.000 | **<0.001 ***** |
| CHK2 | 2.175 | 0.337 | **<0.001 ***** |
| BRCC3 | 15.904 | 0.000 | **<0.001 ***** |
| SLX1 | 102.970 | 0.000 | **<0.001 ***** |
| PMS1 | 30.859 | 0.000 | 0.097 |
| ASCC3b | 21.055 | 0.000 | **<0.001 ***** |
| MSH4 | 64.312 | 0.000 | **<0.001 ***** |
| POLB | 48.574 | 0.000 | **0.004 **** |
| HERC2 | 84.046 | 0.000 | **<0.001 ***** |
| SMARCAL1 | 24.457 | 0.000 | **<0.001 ***** |
| ASCC3a | 53.525 | 0.000 | **<0.001 ***** |
| PNKP | 3.451 | 0.178 | **<0.001 ***** |
| RMI2 | 489.236 | 0.000 | 0.355 |
| ALKBH5 | 2.323 | 0.313 | **<0.001 ***** |
| XPF | 223.466 | 0.000 | 0.149 |
| CLASPIN | 184.827 | 0.000 | **<0.001 ***** |
| ERCC2 | 28.295 | 0.000 | **<0.001 ***** |
| MGMT | 74.780 | 0.000 | **<0.001 ***** |
| ^†^UBC13 | 79.869 | 0.000 | <0.001 *** |
| ATR | 10.684 | 0.005 | **<0.001 ***** |
| ATM | 27.389 | 0.000 | **<0.001 ***** |
| MSH6 | 8.727 | 0.013 | **<0.001 ***** |
| MSH3 | 18.502 | 0.000 | 0.169 |
| KU70 | 52.963 | 0.000 | **<0.001 ***** |
| RNF8 | 86.397 | 0.000 | **0.030 *** |
| H2AFY | 102.170 | 0.000 | 0.052 |
| ECT2 | 76.372 | 0.000 | 0.076 |
| ERCC3 | 78.133 | 0.000 | 0.068 |
| FANCM | 20.888 | 0.000 | **<0.001 ***** |
| CRY1 | 21.298 | 0.000 | 0.088 |
| PMS2 | 37.776 | 0.000 | **<0.001 ***** |
| DDX1 | 281.680 | 0.000 | **0.008 **** |
| XPA | 29.335 | 0.000 | 0.621 |
| RAD17 | 42.386 | 0.000 | 0.456 |
| RAD51A | 55.338 | 0.000 | 0.483 |
| ^†^REV1 | 8.898 | 0.012 | <0.001 *** |
| BRIP1 | 8.095 | 0.017 | **<0.001 ***** |
| NHEJ1 | 167.933 | 0.000 | **0.008 **** |
| ^†^MAPKAPK2 | 63.215 | 0.000 | 0.006 ** |
| RFC1 | 46.606 | 0.000 | 0.420 |
| XRCC1 | 6.567 | 0.037 | 0.310 |
| ^†^POLH | 113.077 | 0.000 | 0.002 ** |
| MUS81 | 15.923 | 0.000 | 0.091 |
| RAD54B | 55.686 | 0.000 | **<0.001 ***** |
| BARD1 | 48.277 | 0.000 | **0.041 *** |
| ^†^EME1 | 44.582 | 0.000 | 0.033 * |
| MLH3 | 239.105 | 0.000 | **<0.001 ***** |
| PRKDC | 36.703 | 0.000 | **0.001 **** |

Comparison of normalized counts per million among castes was conducted using the edgeR package. Bold letters indicate significant differences (*FDR < 0.05, **FDR < 0.01, **FDR < 0.001). LR: likelihood ratio, FDR: false discovery rate. The expression of several genes did not differ between young and mature primary kings (dagger footnote).
