# Supplementary material for: High expression of the breast cancer susceptibility gene BRCA1 in long-lived termite kings
Source: Aging (Albany NY). 2018 Oct 11;10(10):2668–83. doi: 10.18632/aging.101578 (PMC6224230; doi:10.18632/aging.101578)
Supplement: Supplementary Table S6 [file aging-10-101578-s007.docx]

**Table S6. Primer sequences.**

| Target gene | Sequence (5′–3′) | Amplicon (bp) |
| --- | --- | --- |
| *RsBRCA1* | Forward: GGATAGCTGGTTTTGCTGAAGAG | 130 |
|  | Reverse: AGTTGTCTGCTTGCTTTGCTTG |  |
| *RsMCPH* | Forward: ATGGGAAAATGCTGGAGGAA | 104 |
|  | Reverse: TCGTTGAGTATGAGGAGGATGG |  |
| *RsMLH1* | Forward: AACCCCACCAAACTCCACA | 92 |
|  | Reverse: CATCAACAGCATCCACTTCCA |  |
| *RsXRCC3* | Forward: TATATGGTGGCCTTAATGGAGGTG | 96 |
|  | Reverse: TGGGAGGGAAACAGCAGAA |  |
| *RsCDK1* | Forward: TGCCACCAAAGGCGAGT | 106 |
|  | Reverse: CAAATGCCCGACCCAGA |  |
| *RsMGMT* | Forward: AATGCAATGGCAGCAAACC | 114 |
|  | Reverse: CCAGTTCTTCACGCTGTTCTTTT |  |
| *ND5* | Forward: GCTGGGGGGGTTATTCATTCCAT | 125 |
|  | Reverse: GGCATACCACAAAGGGCAAAA |  |
| *RsG6PD* | Forward: GCACTTTGTTCGTTCTGATGAGTT | 144 |
|  | Reverse: TCACTACACACTTCATCTGCCTTCT |  |
